# The burden of disease and injury in the United States 1996

**DOI:** 10.1186/1478-7954-4-11

**Published:** 2006-10-18

**Authors:** Catherine M Michaud, Matthew T McKenna, Stephen Begg, Niels Tomijima, Meghna Majmudar, Maria T Bulzacchelli, Shahul Ebrahim, Majid Ezzati, Joshua A Salomon, Jessica Gaber Kreiser, Mollie Hogan, Christopher JL Murray

**Affiliations:** 1 Harvard Initiative for Global Health, Harvard University, 104 Mt Auburn Street, Cambridge, MA 02138, USA; 2 Centers for Disease Control and Prevention,1600 Clifton Road MS E-47, Atlanta, Georgia 30333, USA; 3 School of Population Health, The University of Queensland, Brisbane, Australia; 4 Columbia University in the City of New York, 2960 Broadway, New York, NY 10027-6902, USA; 5 Haas School of Business, 545 Student Services #1900, University of California, Berkeley, CA 94720-1900, USA; 6 Bloomberg School of Public Health, Johns Hopkins University, 615 N. Wolfe Street, Baltimore, MD 21205, USA

## Abstract

**Background:**

Burden of disease studies have been implemented in many countries using the Disability-Adjusted Life Year (DALY) to assess major health problems. Important objectives of the study were to quantify intra-country differentials in health outcomes and to place the United States situation in the international context.

**Methods:**

We applied methods developed for the Global Burden of Disease (GBD) to data specific to the United States to compute Disability-Adjusted Life Years. Estimates are provided by age and gender for the general population of the United States and for each of the four official race groups: White; Black; American Indian or Alaskan Native; and Asian or Pacific Islander. Several adjustments of GBD methods were made: the inclusion of race; a revised list of causes; and a revised algorithm to allocate cardiovascular disease garbage codes to ischaemic heart disease. We compared the results of this analysis to international estimates published by the World Health Organization for developed and developing regions of the world.

**Results:**

In the mid-1990s the leading sources of premature death and disability in the United States, as measured by DALYs, were: cardiovascular conditions, breast and lung cancers, depression, osteoarthritis, diabetes mellitus, and alcohol use and abuse. In addition, motor vehicle-related injuries and the HIV epidemic exacted a substantial toll on the health status of the US population, particularly among racial minorities. The major sources of death and disability in these latter populations were more similar to patterns of burden in developing rather than developed countries.

**Conclusion:**

Estimating DALYs specifically for the United States provides a comprehensive assessment of health problems for this country compared to what is available using mortality data alone.

## Background

This paper presents the results of a study conducted cooperatively by scientists from the Centers for Disease Control and Prevention (CDC) and the Harvard School of Public Health. The study essentially applied the methods used in the Global Burden of Disease analysis to data specific to the United States in order to calculate Disability-Adjusted Life Year (DALY) values for major health conditions and risk factors [1].

The genesis of the US Burden of Disease and Injury study (USBODI) was the release of the *1993 World Development Report: Investing in Health *published by the World Bank. This landmark report in international health policy introduced a new summary measure of population health – the Disability-Adjusted Life Year (DALY) [2]. In contrast to the traditional reliance on death counts and rates to assess the burden of health events, the DALY attempted to combine the impact of non-fatal health outcomes with mortality. Though originally developed for comparative analyses of disease burden in different regions of the world, this perspective seemed particularly appropriate to inform policy in a country such as the United States. In industrialized country settings, where death rates are low relative to developing countries, the majority of deaths occur after the age of 75 years. Yet too many deaths still occur at younger ages and many could be prevented. Therefore, information for health policy deliberations needs to emphasize the burden of premature mortality as well as disability. As stated in a recent Institute of Medicine report on summary health measures, "Mortality measures, although important, provide decision makers incomplete and insensitive information about overall population health." [3].

From the outset, this study had three major goals. The first goal was to incorporate non-fatal conditions into assessments of health status in the United States. So far most discussions about the relative importance of various health conditions centered on the number of deaths attributed to specific diseases, injuries or risk factors [4]. The focus on deaths has important implications for policy and great influence on resource allocation. As the average life expectancy continues to rise in economically developed countries, more and more deaths are attributed to chronic conditions that are recalcitrant to treatment and may have limited preventability [5]. Prioritization of research and health care expenditures based on such data tends to result in a focus on rescue-oriented, life-saving, and technologically advanced approaches rather than adequate consideration of interventions that promote healthy life-styles and improve overall physical and emotional function [6]. The DALY offers a rational methodology for weighing the relative importance of fatal and non-fatal health events. Hence, a much broader range of health conditions that are rarely identified as causes of death, such as mental health disorders and musculoskeletal diseases, can be introduced into data-based deliberations on health policy.

The second major goal was to develop a comprehensive set of internally consistent and scientifically credible epidemiological estimates for the major health conditions in the United States. This is greatly facilitated by a plethora of population-based surveys, registries and administrative data systems that attempt to capture information on a wide range of health events [7]. The major challenge is to impose a consistent and conceptually rigorous analytic approach so that the estimates are internally consistent. Reviews of cost-effectiveness ratios that depend on epidemiologic data and statistical modeling have demonstrated that it is very difficult to compare results from one study to the next because of major variations in methods, underlying assumptions, data sources and conceptual frameworks [8]. A major source of these inconsistencies is that most such models are developed on a case by case, disease by disease basis, with little attention to conceptual consistency and integration of data from multiple sources [9]. Estimates developed with an adherence to conceptual consistency for the United States can serve not only as a useful source of epidemiologic information, but can also stimulate further analyses and refinements by other investigators.

The third and final goal of the US Burden of Disease and Injury study was to provide a set of internationally comparable health statistics that place the United States public health situation in a global context. World population growth and technological developments over the last few decades in telecommunications, industrial pollution and transportation have effectively made the earth a much smaller planet [10]. This dynamic has major implications for the importation and exportation of health related vectors that include infectious diseases, manufactured products (e.g. energy rich foods, tobacco), and health system organization and practices [11-13]. Given the emergence of this global public health "village," and the growing importance of the DALY as a metric for assessing population health, it seems critical to provide an analysis of the public health situation in the United States that uses methods being adopted by international organizations and health ministries throughout the world.

The Global Burden of Disease (GBD) study developed health statistics for 8 large regions of the world. It includes the United States, which possesses substantial *intra-*national racial, ethnic and cultural variability. Generating a set of estimates specific to the United States not only provides an opportunity to frame the major health problems in this country in a global context, but also facilitates explication of *intra-*national disparities. For example, a previously published monograph that resulted from this project identified differences between race, sex and county-specific life expectancies that rivaled differences seen between the nations with the highest (Japan) and lowest (Sierra Leone) life expectancy values in the world [14].

The overall purpose of this study is to expand the understanding of the major determinants of ill and good health in the United States. The ultimate goal of such understanding should be policies and programs that decrease the overall impact and disparities in disease, disability and premature death.

## Methods

The study was patterned after the GBD and applied methods used in the GBD analysis to compute years of life lost due to premature mortality (YLL), years of life lost due to disability (YLD), and disability-adjusted life years (DALYs). The conceptual and computational details of how these parameters were estimated for individual conditions have been presented in the GBD. A summary overview of GBD methods is provided [see Additional file 1].

The detailed mortality data file for 1996 provided deaths by age, sex and race to compute YLL [15]. The National Health Interview Survey (NHIS), National Health and Nutrition Examination Survey (NHANES), National Longitudinal Alcohol Epidemiologic Survey (NLAES), the National Hospital Discharge Database, disease registers, and epidemiological studies provided the epidemiological parameters needed to compute YLD for 72 conditions that account for at least 90 per cent of the DALY total in the United States. If data on race and gender specific subgroups were too sparse to derive reasonable epidemiological parameters for particular conditions, YLD were estimated for these subgroups using YLD to YLL ratios for the overall population. For the remaining 26 conditions, YLD were calculated using YLD to YLL ratios from Established Market Economies (EME) countries in the GBD, applied to US specific estimates of YLL. A detailed presentation of analytic methods, data sources, and data sets used to develop estimates for major causes of diseases and injuries is provided [see Additional file 2].

Below we describe adjustments that were made to GBD methods in the context of the United States. These were 1) the inclusion of race; 2) a revised list of causes; and 3) a revised algorithm to allocate cardiovascular disease garbage codes to ischaemic heart disease (IHD).

### Selection of population groups

Estimates of the burden of disease and injury were done by gender and seven age groups (0–4; 5–14; 15–24; 25–44; 45–64; 65–74 and 75+) for the total US population, as well as for each of the four official race groups specified by the Office of Management and Budget (OMB): White; Black; American Indian or Alaskan Native; and Asian or Pacific Islander. Whites were the largest population group (82.8 per cent, or 219.7 million). Blacks represented 12.6 per cent of the population (33.5 million); American Indians – 0.9 per cent (2.3 million), and Asians – 3.7 per cent (9.7 million). Estimates by ethnicity were not included in this report because reliable estimates were only available for a subset of the Hispanic population.

The inclusion of race in the analysis posed particular challenges for minority populations because of race misclassification. Two independent data sets were combined to calculate death rates: the number of deaths in the numerator comes from the detailed mortality file, and population numbers in the denominator are from the census. There was no discrepancy in reporting of race in both data sets for Whites and Blacks, but race misclassification was found to be problematic for Asians and American Indians. Self-reporting of race in the census tended to be higher, particularly for American Indians, than was the attribution of race (by a third party) on death certificates – which will yield an underestimate of death rates [16]. However, we did not correct for race misclassification in American Indians and Asians because evaluations showed that discrepancies in race reporting varied from year to year and thus provided supportive evidence that there may be no systematic bias. Therefore race differentials expressed as rates may have been slightly overestimated. Death and DALY rates were age-standardized to the general population.

There were also important gaps in the available epidemiological data for Asians and American Indians. In order to fill information gaps, we assumed that ratios of YLL to YLD by cause, gender and age were similar to that of the total population. Such assumptions introduce a certain level of uncertainty in the estimates, and call for caution in the interpretation of rankings for causes that have small differences in the number of DALYs. For simplicity, American Indians or Alaskan Natives are referred to as "American Indians," and Asians or Pacific Islanders as "Asians" in the text, tables and figures below.

### USBODI cause list

Even though essentially all deaths in the United States are registered and medically certified, a detailed assessment of mortality data was conducted as part of the USBODI. This was done to further explore and refine the utility of the adjustment procedures for misclassification that were used in the GBD, and to provide a contrast to the overall results using the DALY.

The *International Statistical Classification of Disease and Related Health Problems*, Version 9, (ICD-9) [17] code listed as the underlying cause for each death recorded in the United States in 1996 was attributed to corresponding disease categories listed in the GBD. The GBD classification scheme was developed as a tool to better inform the health policy debate (Table 1). The list of causes selected for the USBODI was amended based on the distribution of causes of deaths in the United States. All ICD-9 reported codes accounting for more than 0.1 per cent of total deaths were examined. This process identified modifications that were needed from the GBD cause list. Several causes of little relevance to the United States were dropped, i.e. malaria and other tropical diseases. Other causes were added, i.e. Sudden Infant Death Syndrome (SIDS), and septicemia. For those codes accounting for more than 0.1 per cent of deaths that were not included in the GBD list the choice was made in consultation with CDC based on two major criteria. If the code represented a true cause of death with significance for health policy, it was added to the cause list. If the code more likely represented a "garbage category," then after consultation with experts in that disease and a review of published autopsy studies on this subject, a redistribution algorithm was proposed and applied. For example, careful consideration was given to the nearly 10.9 per cent of cancer deaths assigned to "unknown primary." There were another 4% of cancers that did not have a code that corresponded to the GBD classification system. The race and sex specific age distribution of cancers attributed to an ill-defined primary source were compared to all other cancer deaths. These distributions were generally similar. Therefore, cancers attributed to an ill-defined primary source were redistributed proportionally to all defined primary sources based on age, race and sex specific distributions. The detailed list of causes selected for the USBODI is provided [see Additional file 3].

**Table 1:** Global burden of disease classification system – main categories

**Communicable, maternal, perinatal and nutritional conditions (Group I)**

A. Infectious and parasitic diseases
B. Respiratory infections
C. Maternal conditions
D. Conditions arising during the perinatal period
E. Nutritional deficiencies

**Noncommunicable diseases (Group II)**

A. Malignant neoplasms
B. Other neoplasms
C. Diabetes mellitus
D. Endocrine disorders
E. Neuro-psychiatric conditions
F. Sense organ diseases
G. Cardiovascular diseases
H. Respiratory diseases
I. Digestive diseases
J. Genito-urinary diseases
K. Skin diseases
L. Musculo-skeletal diseases
M. Congenital anomalies
N. Oral conditions

**Injuries (Group III)**

A. Unintentional injuries
B. Intentional injuries


Source: Global Burden of Disease and Injury 1990

### Redistribution algorithm for cardiovascular garbage codes

The most problematic aspect of cause of death coding pertains to coding of ischaemic heart disease (IHD) (ICD-9 codes 410–414), which is one of the leading causes of premature mortality. The wide cross-national variations that exist in IHD reported mortality rates were explored in the context of the GBD and were convincingly attributed to variations across countries in coding practices. Physicians may use several ICD-9 codes that are actually due to IHD when they assign the cause of death. These include heart failure (428), ventricular dysrhythmias (427.1, 427.4, 427.5), general atherosclerosis (440.9), and ill-defined descriptions and complications of heart disease (429.0, 429.1, 429.2 and 429.9). IHD deaths may be assigned to these ill-defined cardiovascular codes, or "garbage codes" because of insufficient clinical information at the time of death, local medical diagnostic practices or simply by error. The statistical approach developed to correct for likely undercoding resulting from different coding practices in the GBD included a two-step procedure comprising an ordinary least squares (OLS) regression equation predicting the proportion of cardiovascular death for each age group assigned to ill-defined codes as a function of the proportion of deaths assigned to IHD, and the correction of proportions for each country within set constraints, based on the assumption that the cluster of countries where ill-defined coding was low defined the standard coding practices.

**Figure 1:**
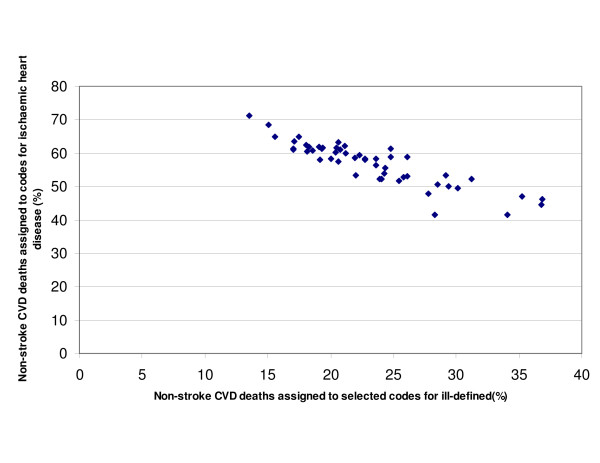
Proportion of cardiovascular disease deaths (excluding stroke) assigned to selected codes for ill-defined causes and directly assigned to ischemic heart disease in the United States.

An exploration of cardiovascular death coding in the United States showed important differences in coding practices between states. Indeed, the proportion of all cardiovascular deaths (minus stroke) coded to cardiovascular "garbage" codes ranged from 14% in New Mexico to 37% in Alabama and New Jersey (Table 2). Figure 1 illustrates the enormous variation across US states in coding practices with respect to these ill-defined cardiovascular codes. For each state, the fraction of cardiovascular deaths (excluding stroke) that are assigned to ICD-9 codes 410–414 is shown on the y-axis. On the x-axis the fraction of cardiovascular deaths (excluding stroke) that are assigned to the ill-defined cardiovascular codes is measured. The strong negative relation between IHD mortality and that from ill-defined cardiovascular codes supports the suggestion that the quality of IHD death certification varies substantially across states, as it does across countries in the world.

**Table 2:** Proportion of all cardiovascular deaths (except stroke) coded to cardiovascular "garbage codes" by state – United States 1996

**State name**	**% CV garbage**	**State name**	**% CV garbage**	**State name**	**% CV garbage**
Alabama	37	Kentucky	25	North Dakota	19
Alaska	22	Louisiana	23	Ohio	25
Arizona	22	Maine	24	Oklahoma	18
Arkansas	21	Maryland	24	Oregon	23
California	18	Massachusetts	22	Pennsylvania	15
Colorado	26	Michigan	19	Rhode Island	20
Connecticut	29	Minnesota	35	South Carolina	21
Delaware	29	Mississippi	17	South Dakota	19
DC	28	Missouri	29	Tennessee	21
Florida	16	Montana	31	Texas	24
Georgia	30	Nebraska	34	Utah	24
Hawaii	28	Nevada	20	Vermont	26
Idaho	17	New Hampshire	17	Virginia	24
Illinois	19	New Jersey	37	Washington	26
Indiana	18	New Mexico	14	West Virginia	17
Iowa	20	New York	19	Wisconsin	21
Kansas	24	North Carolina	21	Wyoming	19

This preliminary analysis confirmed the need to correct for under-registration of IHD in the US. To estimate the fraction of IHD deaths assigned to ill-defined cardiovasular codes, the regression equation applied in the GBD was revised. Age and sex specific lung cancer death rates were added to the model. Lung cancer mortality rates measure the cumulative effects of tobacco exposure as a risk factor for IHD [18].

The regression model for the US included age and sex specific lung cancer death rates, and ill-defined cardiovascular disease (CVD) rates for Blacks and Whites. These regression equations predicted the proportion of ill-defined CVD deaths by age and sex for Whites and Blacks. We applied results of regression equations for Whites to American Indians and Asians, which were not included in the regressions due their small population size.

The finding that the extent of miscoding increased in older age groups is consistent with GBD regression results: R-square increases with increasing age, which provides further evidence that ill-defined codes are indeed being used for IHD which is more common in older ages. Differences in coding practices observed by race as well as gender are not fully explained (Table 3). A recent study concluded that "the greater presence of medical knowledge at the time of death, reflected by place of death and cardiologist per capita, reduces the use of ill-defined cardiovascular clusters. Racial and gender effects on coronary heart disease (CHD) assignment may reflect disparities in access to care and quality of care." [19]

**Table 3:** R-squared values applied to the redistribution of cardiovascular garbage codes

**WHITES**					
Male	Lung Cancer		CVGarbageCodes		R-squared
	Coefficient	Pvalue	Coefficient	Pvalue	
15	-0.499	0.273	0.557	.000	26.17%
30	2.396	.000	-0.193	0.424	52.10%
45	1.495	.000	-0.741	.000	77.77%
65	1.235	.000	-0.833	.000	69.02%
75	2.73	.000	-1.122	.000	47.64%
					
Female					
15	0.348	0.0324	0.097	0.395	3.53%
30	0.727	.000	0.539	0.008	37.82%
45	0.869	.000	-0.315	0.283	31.41%
65	-0.459	0.206	0.752	0.018	11.47%
75	2.011	0.123	-0.679	0.021	12.25%

**BLACKS**					
Male	Lung Cancer		CV GarbageCodes		R-squared
	Coefficient	Pvalue	Coefficient	Pvalue	

15	0.592	0.314	-0.0393	0.792	4.13%
30	1.575	0.013	0.0735	0.785	23.40%
45	1.211	.000	-0.403	0.025	47.27%
65	0.435	0.082	-0.551	.000	40.73%
75	0.183	0.769	-0.7207	0.005	26.34%
					
Female					
15	-0.829	0.042	0.256	0.047	16.06%
30	-0.846	0.0127	0.532	0.03	12.85%
45	0.02	0.942	-0.11	0.634	8.30%
65	0.695	0.42	-1.369	0.059	13.60%
75	1.015	0.524	-0.772	0.013	16.67%

### International comparisons

Ten countries with comparable levels of development and a population greater than 10 million: Australia, Canada, France, Germany, Greece, Italy, Japan, Netherlands, Spain and the United Kingdom, were selected for international comparisons. YLL by cause were obtained directly from the World Health Organization (WHO). YLD and DALY estimates were only available at the regional level, with the exception of Australia, where a national burden of disease study applying the GBD methodology had been conducted [21].

International comparisons may address two sets of issues – the difference in the magnitude of YLL (expressed as YLL rates), or differences in the distribution of major causes of YLL. We examined differences in rankings of major causes of YLL and YLL rates between the United States and other comparable countries.

Rankings for the twenty leading causes of mortality burden in the United States were compared to rankings for these conditions in the ten selected countries. We made one change in the list of conditions adopted for the United States to ensure comparability among countries, which was to combine mortality burden due to lymphomas and multiple myelomas. These two conditions are different forms of reticuloendothelial malignancies. Estimates were not available for these conditions separately in several of the selected countries. This change slightly altered rankings in the United States for several conditions, as the two conditions combined had a higher mortality burden than lymphomas alone. Lymphomas and multiple myelomas ranked 14^th ^for males and 13^th ^for females, and the mortality burden for lymphomas ranked 19^th ^and 17^th ^respectively. We plotted the rankings for each of the twenty leading causes of mortality burden in the United States (horizontal bars) against the range of rankings observed for each of these conditions in the selected countries (vertical bars), for each sex. The lowest and highest rankings observed in the countries other than the US define the bounds of vertical bars for each condition. Rankings, from one to twenty, are inversely related to the magnitude of mortality burden. Thus, IHD, which caused the largest number of YLL in the United States, ranked 1^st^. We also compared YLL rankings for the twenty leading causes of YLL for each race and sex against the ranges observed in the ten selected countries.

## Results

Detailed tabulations of deaths, YLL, YLD and DALYs for the 73 causes included in the USBODI by age, gender and race are provided [see Additional file 4]. Epidemiological parameters (incidence, prevalence, age at onset, duration, remission rates) and disability weights for each condition are provided [see Additional file 5].

Below we report key findings for the burden of disease and injury (DALYs); the mortality burden due to premature deaths (YLL); and the disability burden due to non-fatal health outcomes (YLD).

### Burden of disease and injury

#### Leading causes of DALYs

**Figure 2:**
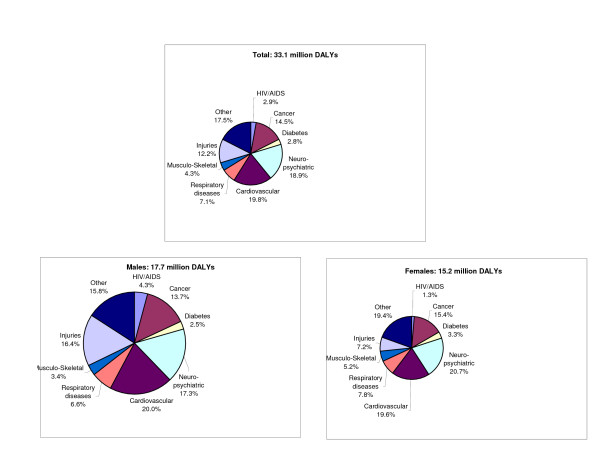
Burden of Disease (DALYs) by sex and major disease groups, US, 1996.

The burden of disease and injury resulting from premature deaths and disability was an estimated 33 million DALYs in 1996. Premature mortality contributed 55 per cent of the total (18 million YLL), and disability – 45 per cent (15 million YLD). Noncommunicable diseases (Group II) caused 80 per cent of total DALYs, the balance being almost equally divided between communicable diseases, maternal, perinatal and nutritional causes (Group I) and injuries (Group III). Cardiovascular diseases, neuropsychiatric conditions, cancers and injuries caused approximately two thirds of the total DALYs (Figure 2). Ischaemic heart disease (IHD) was the leading and single largest cause of deaths and DALYs causing almost 10 per cent of DALYs. The three other causes ranking in the top five-cerebrovascular diseases, motor vehicle accidents, unipolar major depression – contributed almost equally to the total burden, with shares ranging between 4.1 and 4.6 per cent (Table 6).

**Table 6:** Twenty leading causes of DALYs and deaths, US 1996

	**DALY**	**% of total**		**Deaths**	**% of total**
**Total DALYs**	**33,090,212**		**Total Deaths**	**2,314,689**	
					
Ischemic heart disease	3,134,732	9.5	Ischemic heart disease	536,314	23.17
Cerebrovascular Disease	1,510,287	4.6	Lung trachea or bronchial cancer	168,206	7.27
Motor vehicle accidents	1,393,278	4.2	Cerebrovascular Disease	161,678	6.98
Unipolar major depression	1,370,285	4.1	COPD	99,982	4.32
Lung trachea or bronchial cancer	1,362,712	4.1	Lower respiratory infections	84,319	3.64
COPD	1,253,491	3.8	Diabetes mellitus	62,452	2.70
Alcohol use	1,141,193	3.4	Cancer colon or rectum	61,189	2.64
HIV	956,418	2.9	Breast cancer	46,649	2.02
Diabetes mellitus	946,291	2.9	Motor vehicle accidents	43,735	1.89
Osteoarthritis	942,682	2.8	Dementia and other degenerative and hereditary CNS disorders	43,190	1.87
Dementia and other degenerative and hereditary CNS disorders	889,242	2.7	Hypertension and hypertensive heart disease	39,589	1.71
Congenital Abnomalities	761,951	2.3	Prostate cancer	36,667	1.58
Homicide and Violence	714,621	2.2	Self-inflicted	31,725	1.37
Self-inflicted	674,443	2.0	HIV	31,188	1.35
Asthma	665,103	2.0	Cancer pancreas	29,494	1.27
Drug use	543,841	1.6	Inflammatory Cardiac	29,066	1.26
Breast cancer	514,786	1.6	Lymphomas	26,443	1.14
Conditions arising during the perinatal period	493,958	1.5	Cirrhosis of the liver	25,488	1.10
Cancer colon or rectum	483,931	1.5	Nephritis or nephrosis	24,569	1.06
Cirrhosis of the liver	411,539	1.2	Homicide and Violence	22,351	0.01

#### Sex and age patterns

The total disease burden for males (17.9 million DALYs) exceeded that for females (15 million DALYs). The excess disease burden for males was mostly due to the much larger number of premature deaths in young adult males. IHD resulted in twice the number of DALYs for males as it did for females, and was equal to the combined disease burden due to the three major causes of injuries that took a high toll in young adult males – motor vehicle accidents, homicide and violence, and self-inflicted injuries. Unipolar major depression caused almost the same disease burden for females that did motor vehicle accidents for males (Table 7).

**Table 7:** Twenty leading causes of DALYs, by sex, US 1996

	**Males**		
	**Cause list**	**DALY**	**% total**
	**Total DALY**	**17,860,393**	
1	Ischaemic heart disease	1,958,184	11.0%
2	Motor vehicle accidents	933,798	5.2%
3	Lung trachea or bronchial cancer	812,804	4.6%
4	HIV	763,816	4.3%
5	Alcohol use	731,890	4.1%
6	Cerebrovascular Disease	673,928	3.8%
7	COPD	641,701	3.6%
8	Homicide and Violence	567,717	3.2%
9	Self-inflicted	541,399	3.0%
10	Unipolar major depression	469,929	2.6%
11	Diabetes mellitus	442,051	2.5%
12	Osteoarthritis	434,856	2.4%
13	Drug use	411,780	2.3%
14	Congenital Abnomalities	410,388	2.3%
15	Dementia and other degenerative and hereditary CNS disorders	382,392	2.1%
16	Asthma	303,088	1.7%
17	Cirrhosis of the liver	280,632	1.6%
18	Conditions arising during the perinatal period	273,577	1.5%
19	Cancer colon or rectum	249,462	1.4%
20	Prostate cancer	238,889	1.3%
			
	**sub-total**	**11,522,281**	**64.5%**
			

	**Females**		

	**Cause list**	**DALY**	**% total**
	**Total DALY**	**15,229,819**	
1	Ischaemic heart disease	1,176,548	7.7%
2	Unipolar major depression	900,356	5.9%
3	Cerebrovascular Disease	836,359	5.5%
4	COPD	611,790	4.0%
5	Lung trachea or bronchial cancer	549,908	3.6%
6	Breast cancer	514,786	3.4%
7	Osteoarthritis	507,826	3.3%
8	Dementia and other degenerative and hereditary CNS disorders	506,849	3.3%
9	Diabetes mellitus	504,240	3.3%
10	Motor vehicle accidents	459,480	3.0%
11	Alcohol use	409,303	2.7%
12	Asthma	362,015	2.4%
13	Congenital Abnomalities	351,563	2.3%
14	Cancer colon or rectum	234,469	1.5%
15	Conditions arising during the perinatal period	220,382	1.4%
16	Lower respiratory infections	195,448	1.3%
17	PTSD	193,533	1.3%
18	HIV	192,602	1.3%
19	Panic disorder	182,218	1.2%
20	Bipolar disorder	165,236	1.1%

Half of the total disease burden in the United States occurred in adults between the ages of 25 and 64 years, the other half being almost evenly split between younger and older age groups: 23 per cent under the age of 25 years, and 27 per cent for ages 65 years and above. In sharp contrast, the number of deaths gradually increased with age. More than half of all deaths occurred in adults aged 75 years and older (Table 8).

**Table 8:** Ten leading causes of DALYs by age, US 1996

**Rank**	**All ages**	**DALYs**	**% of total**	**0–4**	**DALYs**	**% of total**
	Total	33,090,212		Total	2,123,767	
1	Ischaemic heart disease	3,134,732	9.5	Congenital abnomalities	679,542	32.0
2	Cerebrovascular disease	1,510,287	4.6	Perinatal conditions	492,486	23.2
3	Motor vehicle accidents	1,393,278	4.2	Sudden infant death syndrome	102,255	4.8
4	Unipolar major depression	1,370,285	4.1	Asthma	77,323	3.6
5	Lung, trachea or bronchial cancer	1,362,712	4.1	Diarrhoeal diseases	60,438	2.8
6	COPD	1,253,491	3.8	Motor vehicle accidents	48,630	2.3
7	Alcohol use	1,141,193	3.4	Falls	41,289	1.9
8	HIV	956,418	2.9	Homicide and violence	35,055	1.7
9	Diabetes mellitus	946,291	2.9	Lower respiratory infections	30,640	1.4
10	Osteoarthritis	942,682	2.8	Fires	22,090	1.0
						

**Rank**	**5–14**	**DALYs**	**% of total**	**15–24**	**DALYs**	**% of total**

	Total	1,136,989		Total	3,884,235	
1	Asthma	236,494	20.8	Motor vehicle accidents	499,505	12.9
2	Motor vehicle accidents	128,357	11.3	Alcohol use	433,515	11.2
3	Unipolar major depression	61,622	5.4	Drug use	291,844	7.5
4	Epilepsy	42,461	3.7	Homicide and violence	282,746	7.3
5	Schizophrenia	41,254	3.6	Schizophrenia	237,967	6.1
6	Falls	39,886	3.5	Bipolar disorder	221,134	5.7
7	Homicide and violence	28,242	2.5	Unipolar major depression	197,309	5.1
8	Fires	19,514	1.7	Panic disorder	158,379	4.1
9	Congenital abnomalities	17,860	1.6	Asthma	157,997	4.1
10	Drowning	16,472	1.4	Self-inflicted	157,281	4.0
						

**Rank**	**25–44**	**DALYs**	**% of total**	**45–64**	**DALYs**	**% of total**

	Total	8,364,608		Total	8,478,954	
1	Unipolar major depression	823,548	9.8	Ischaemic heart disease	1,154,002	13.6
2	HIV	751,598	9.0	Lung, trachea or bronchial cancer	630,224	7.4
3	Alcohol use	549,949	6.6	COPD	504,418	5.9
4	Motor vehicle accidents	523,203	6.3	Cerebrovascular Disease	492,918	5.8
5	Self-inflicted	352,241	4.2	Diabetes mellitus	395,612	4.7
6	Homicide and violence	308,550	3.7	Osteoarthritis	361,774	4.3
7	Ischaemic heart disease	274,704	3.3	Breast cancer	250,963	3.0
8	Diabetes mellitus	238,472	2.9	Unipolar major depression	237,590	2.8
9	COPD	234,552	2.8	Cirrhosis of the liver	208,861	2.5
10	Drug use	222,535	2.7	Cancer colon or rectum	190,453	2.2
						

**Rank**	**65–74**	**DALYs**	**% of total**	**75+**	**DALYs**	**% of total**

	Total	4,710,335		Total	4,391,323	
1	Ischaemic heart disease	820,583	17.4	Ischaemic heart disease	876,239	20.0
2	Lung, trachea or bronchial cancer	448,452	9.5	Dementias	469,035	10.7
3	Cerebrovascular disease	373,629	7.9	Cerebrovascular disease	420,278	48.0
4	COPD	282,397	6.0	Lung, trachea or bronchial cancer	200,620	4.6
5	Osteoarthritis	266,685	5.7	COPD	186,379	4.2
6	Dementias	224,484	4.8	Osteoarthritis	161,077	3.7
7	Diabetes mellitus	168,605	3.6	Lower respiratory infections	146,631	3.3
8	Cancer colon or rectum	138,630	2.9	Cancer colon or rectum	106,111	2.4
9	Prostate cancer	97,033	2.1	Diabetes mellitus	106,061	2.4
10	Breast cancer	94,919	2.0	Prostate cancer	81,456	1.9

The share of total DALYs was very similar for both sexes up to the age of 14 years, but increased in adult males between 15 and 64 years. In older adults, the share of total DALYs for females exceeded that for males (Table 9). Differentials in DALY rates between males and females were greatest between 25 and 44 years, when motor vehicle accidents, alcohol use and abuse, HIV/AIDS and major unipolar depression took the highest toll.

**Table 9:** Distribution of burden of disease (DALYs) by age group and sex, US, 1996

**Total**			**Males**		**Females**	
**Age Group**	**DALYs**	**% of total**	**DALYs**	**% of total**	**DALYs**	**% of total**

0–4 years	2,123,767	6.4%	1,164,600	6.5%	959,167	6.3%
5–14 years	1,136,989	3.4%	623,416	3.5%	513,573	3.4%
15–24 years	3,884,235	11.7%	2,279,895	12.8%	1,604,340	10.5%
25–44 years	8,364,608	25.3%	4,800,710	26.9%	3,563,898	23.4%
45–64 years	8,478,954	25.6%	4,754,166	26.6%	3,724,788	24.5%
65–74 years	4,710,335	14.2%	2,455,407	13.7%	2,254,928	14.8%
75 years and over	4,391,323	13.3%	1,782,198	10.0%	2,609,125	17.1%
						
**Total**	33,090,212		17,860,393		15,229,819	

#### Patterns by race

**Figure 3:**
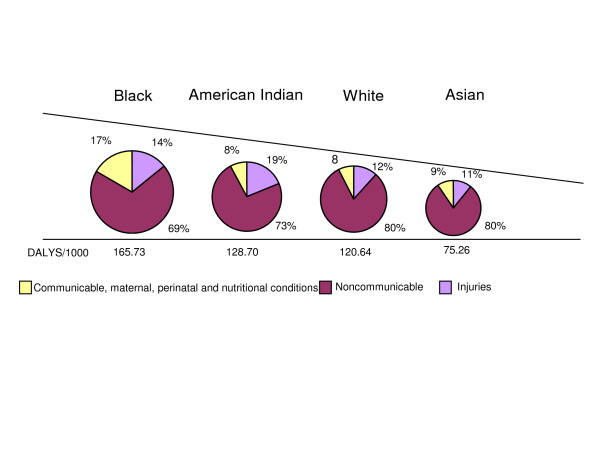
Groups I, II, and III as a percentage of total burden of disease (DALY) by race, US, 1996.

Blacks and American Indians suffered disproportionate shares of total burden relative to their population size: DALY rates per thousand were 165.7 for Blacks; 128.7 for American Indians; 120.6 for Whites, and 75.3 for Asians. The proportional distribution of Groups I, II, and III varied between races, pointing to important differences in prevailing patterns of burden of disease. Group I and III combined caused one fifth of total DALYs for Whites and Asians, and one third of total DALYs for Blacks and American Indians. The excess was due to Group I (17 per cent of total DALYs) for Blacks and Group III for American Indians (19 per cent of total DALYs) (Figure 3).

IHD was among the three leading causes of DALYs for all races. The two other causes were cerebrovascular diseases and lung cancer for Whites; HIV/AIDS and homicide and violence for Blacks; alcohol use and motor vehicle accidents for American Indians; and unipolar major depression and cerebrovascular diseases for Asians (Table 10).

**Table 10:** Ten leading causes of DALYs by race, US 1996

	**Whites**	**DALYs**	**% total**		**American Indians**	**DALYs**	**% total**
Rank	Total	26,510,011		Rank	Total	294,474	
1	Ischaemic heart disease	2,710,918	10.2%	1	Alcohol use	46,419	15.8%
2	Cerebrovascular Disease	1,201,246	4.5%	2	Motor vehicle accidents	23,112	7.8%
3	Lung trachea or bronchial cancer	1,170,492	4.4%	3	Ischaemic heart disease	14,598	5.0%
4	Motor vehicle accidents	1,148,293	4.3%	4	Unipolar major depression	11,815	4.0%
5	Unipolar major depression	1,127,045	4.3%	5	Cirrhosis of the liver	9,293	3.2%
6	COPD	1,111,489	4.2%	6	Diabetes mellitus	9,070	3.1%
7	Alcohol use	857,509	3.2%	7	Self-inflicted	8,336	2.8%
8	Osteoarthritis	820,284	3.1%	8	Cerebrovascular Disease	8,241	2.8%
9	Dementia and other degenerative and hereditary CNS disorders	791,780	3.0%	9	Homicide and Violence	7,754	2.6%
10	Diabetes mellitus	727,575	2.7%	10	Congenital Abnormalities	7,489	2.5%
	**Sub-total**	**11,666,630**	**44.0%**		**sub-total**	**146,128**	**49.6%**
							

	**Blacks**	**DALYs**	**% total**		**Asians**	**DALYs**	**% total**

Rank	Total	5,552,448		Rank	Total	733,279	
1	HIV/AIDS	429,383	7.7%	1	Unipolar major depression	54,264	7.4%
2	Ischaemic heart disease	370,170	6.7%	2	Ischaemic heart disease	39,046	5.3%
3	Homicide and Violence	336,215	6.1%	3	Cerebrovascular Disease	33,883	4.6%
4	Cerebrovascular Disease	266,918	4.8%	4	COPD	29,040	4.0%
5	Alcohol use	230,780	4.2%	5	Osteoarthritis	29,027	4.0%
6	Motor vehicle accidents	193,159	3.5%	6	Motor vehicle accidents	28,714	3.9%
7	Diabetes mellitus	189,656	3.4%	7	Congenital Abnormalities	28,238	3.9%
8	Unipolar major depression	177,162	3.2%	8	Asthma	26,137	3.6%
9	Conditions arising during the perinatal period	174,558	3.1%	9	Diabetes mellitus	19,989	2.7%
10	Lung trachea or bronchial cancer	172,425	3.1%	10	Dementia and other degenerative and hereditary CNS disorders	17,831	2.4%
	**sub-total**	**2,540,426**	**45.8%**		**sub-total**	**306,170**	**41.8%**

**Figure 4:**
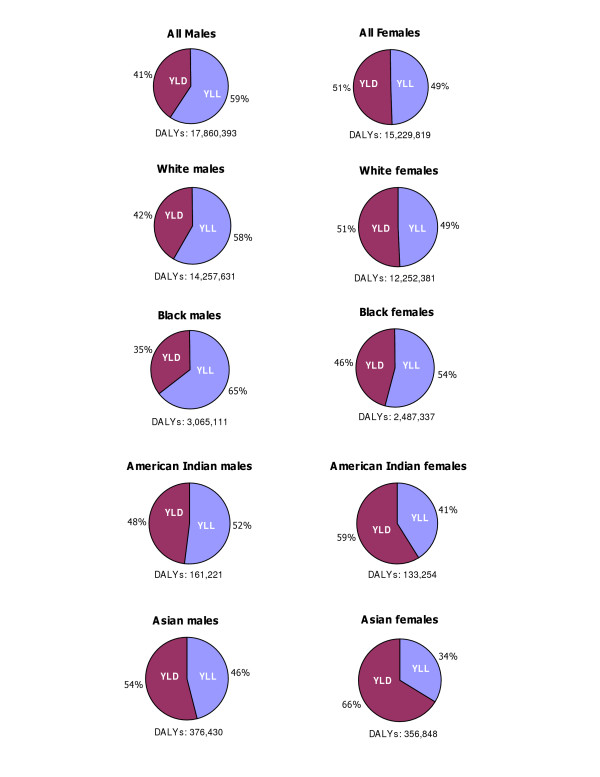
Distribution of YLL and YLD by sex and race, US, 1996.

Sex differentials in total burden by race increased with higher DALY rates. Male to female DALY ratios were 1.23 for Blacks, 1.21 for American Indians, 1.10 for Whites, 1.05 for Asians, and 1.17 overall. Although patterns of disease burden differed between races, leading causes were common to both sexes. Premature deaths contributed the largest share of total burden for males in all races, with the exception of Asian males. Non-fatal health outcomes contributed the largest share for females in all races, with the exception of Black females (Figure 4).

**Figure 5:**
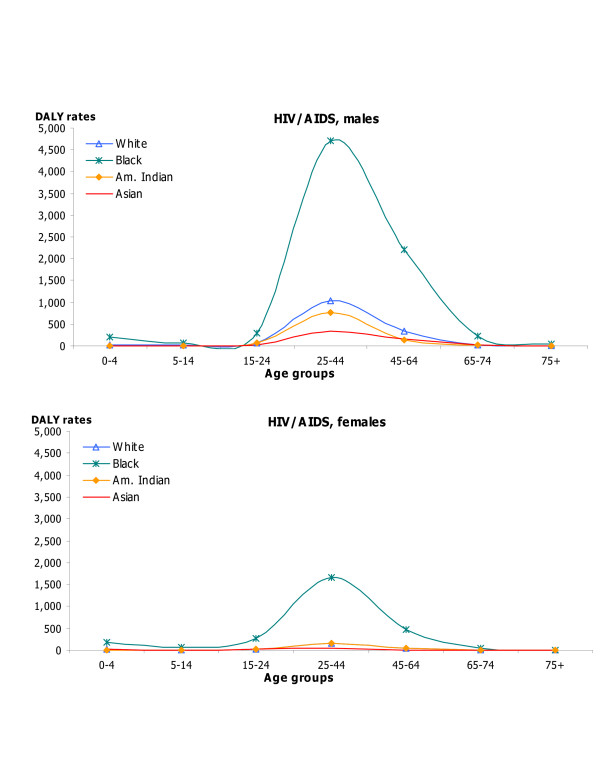
HIV/AIDS: distribution of DALY rates/100,000 by age, race and sex, US 1996.

**Figure 6:**
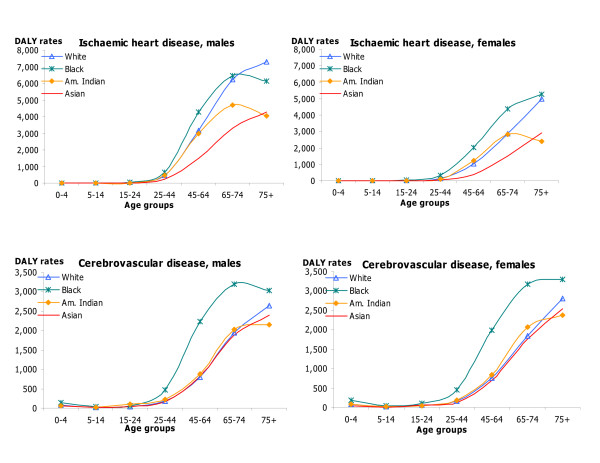
IHD and cerebrovascular diseases: distribution of DALY rates/100,000 by age, race and sex, US 1996.

**Figure 7:**
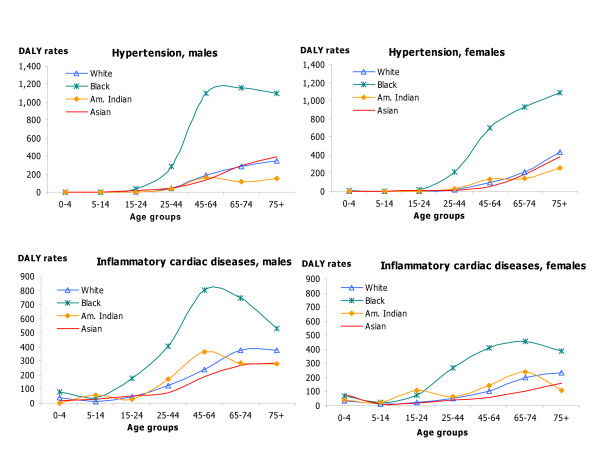
Hypertension and inflammatory cardiac diseases: distribution of DALY rates/100,000 by age, race and sex, US 1996.

**Figure 8:**
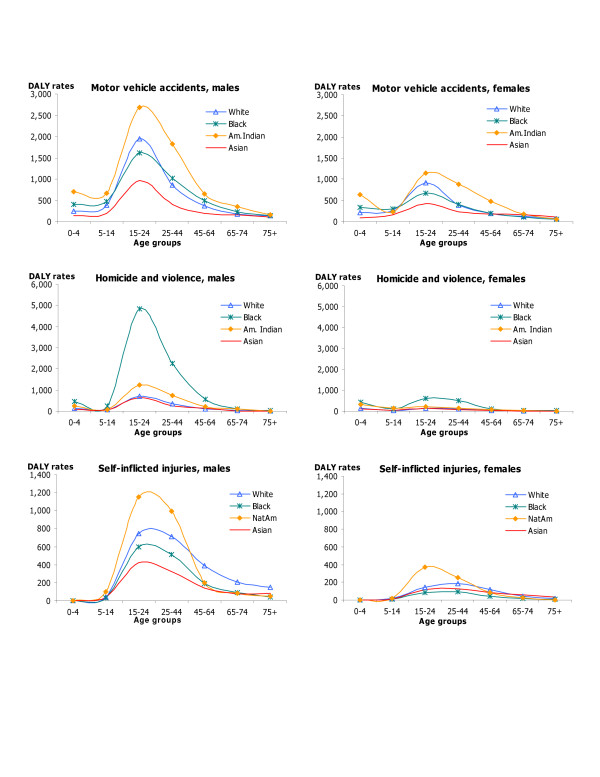
Major causes of injuries: distribution of DALY rates/100,000 by age, race and sex, US 1996.

DALY rates by age, sex, and race for HIV/AIDS, IHD and cerebrovascular diseases; hypertension and hypertensive heart disease, inflammatory cardiac diseases; and major causes of injuries capture changes in DALY rates over the lifespan as well as differences by race and sex in these important causes of disease burden (Figures 5, 6, 7, 8). DALY rates peaked in young adults for HIV/AIDS and injuries, and increased with age for cardiovascular diseases. DALY rates for Black males and females exceeded rates for the other race groups for HIV/AIDS, hypertension, cerebrovascular diseases, inflammatory cardiac diseases, motor vehicle accidents, homicide and violence, and self-inflicted injuries. Differentials between Blacks and the other races were always greater for males than for females, and were greatest in young adult males for HIV/AIDS, homicide and violence, hypertension and inflammatory cardiac diseases. Asian males and females had the lowest DALY rates for all major causes of burden. Differentials between races were least pronounced for IHD for both sexes.

### Mortality burden

#### Leading causes of YLL

**Figure 9:**
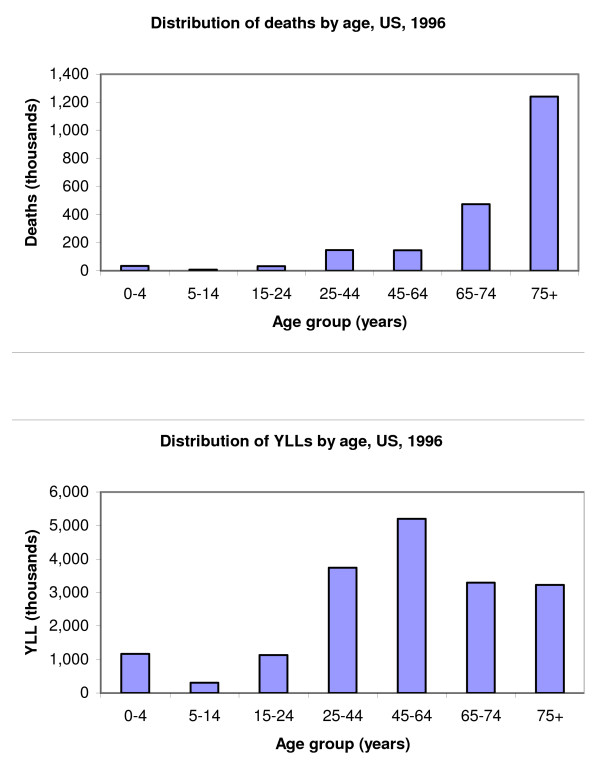
Distribution of deaths and YLL by age, US 1996.

**Figure 10:**
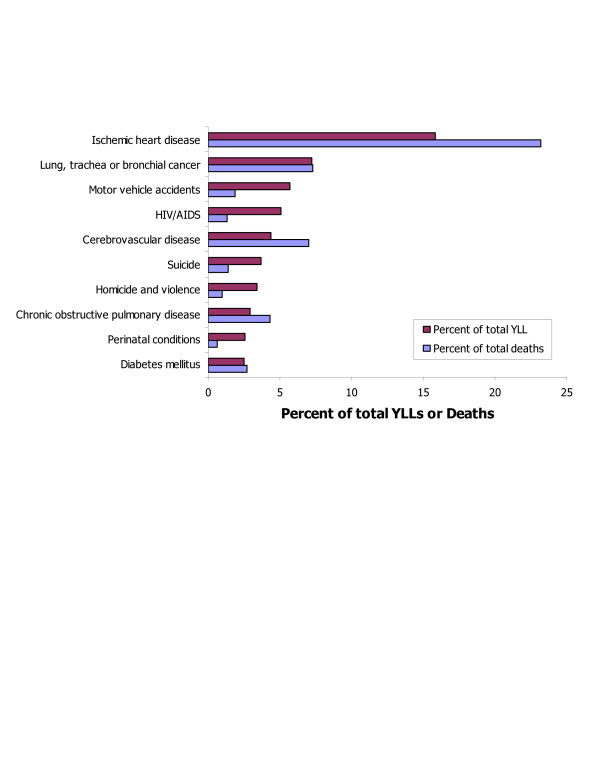
Ten leading causes of mortality burden and death, as per cent of total, both sexes, US 1996.

In 1996, 2.3 million people died in the United States, causing the loss of 18.1 million YLL (55 per cent of total DALYs). Age patterns of deaths and YLL differ: the number of deaths increased with age, the resulting number of YLL was greater for children and young adults than it was for older ages (Figure 9). The number of deaths and resulting number of YLL from any cause are not equivalent (Figure 10).

IHD was the unequivocal lead cause of death and YLL, causing almost one of every four deaths and 16 per cent of total YLL. The mortality burden due to IHD was more than double the mortality burden due to lung cancer, and almost three times that due to motor vehicle accidents (Table 11). The share of YLL exceeded that of YLD for cardiovascular diseases, cancers, injuries, respiratory infections, and conditions arising during the perinatal period.

**Table 11:** Leading causes of death and YLL, both sexes, all races combined

		**Deaths**	**%total**
	***Total Deaths***	**2,314,689**	
1	Ischaemic heart disease	536,314	23.2%
2	Lung trachea or bronchial cancer	168,206	7.3%
3	Cerebrovascular Disease	161,678	7.0%
4	COPD	99,982	4.3%
5	Lower respiratory infections	84,319	3.6%
6	Diabetes mellitus	62,452	2.7%
7	Cancer colon or rectum	61,189	2.6%
8	Breast cancer	46,649	2.0%
9	Motor vehicle accidents	43,735	1.9%
10	Dementia and other degenerative and hereditary CNS disorders	43,190	1.9%
11	Hypertension and hypertensive heart disease	39,589	1.7%
12	Prostate cancer	36,667	1.6%
13	Self-inflicted	31,725	1.4%
14	HIV	31,188	1.3%
15	Cancer pancreas	29,494	1.3%
16	Inflammatory Cardiac	29,066	1.3%
17	Lymphomas	26,443	1.1%
18	Cirrhosis of the liver	25,488	1.1%
19	Nephritis or nephrosis	24,569	1.1%
20	Homicide and Violence	22,351	1.0%
			
	**Sub-total**	**1,604,297**	**69.3%**
			

		**YLL**	**% total**

	**Total YLL**	**18,066,099**	
1	Ischaemic heart disease	2,858,744	15.8%
2	Lung trachea or bronchial cancer	1,301,182	7.2%
3	Motor vehicle accidents	1,027,005	5.7%
4	Cerebrovascular Disease	784,443	4.3%
5	HIV	718,975	4.0%
6	Self-inflicted	660,917	3.7%
7	Homicide and Violence	615,332	3.4%
8	COPD	526,219	2.9%
9	Conditions arising during the perinatal period	464,131	2.6%
10	Diabetes mellitus	450,913	2.5%
11	Breast cancer	450,327	2.5%
12	Cancer colon or rectum	409,534	2.3%
13	Lower respiratory infections	388,441	2.2%
14	Cirrhosis of the liver	321,588	1.8%
15	Congenital Abnomalities	318,948	1.8%
16	Inflammatory Cardiac	258,328	1.4%
17	Hypertension and hypertensive heart disease	241,073	1.3%
18	Lymphomas	233,048	1.3%
19	Poisoning	221,906	1.2%
20	Cancer pancreas	205,972	1.1%
			
	**Sub-total**	**12,457,024**	**69.0%**

#### Sex and age patterns

**Figure 11:**
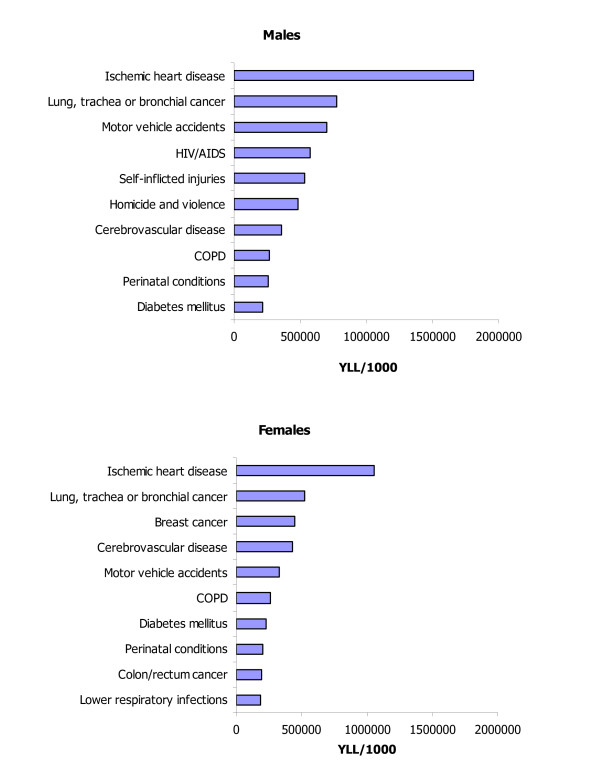
Leading causes of mortality burden (YLL) by sex, US, 1996.

The number of deaths and the age at death is driving differentials in mortality burden observed by age, sex and race. The mortality burden for males (10.5 million YLL) was 40 per cent greater than that for females (7.5 million YLL) (Table 12). The excess male mortality burden was largely due to the higher mortality burden resulting from IHD, injuries (motor vehicle accidents, homicide and violence, self-inflicted injuries), and HIV/AIDS. These causes combined resulted in 40 per cent of total YLL (4.1 million YLL) for males, but only in 24 per cent of total YLL for females (1.8 million YLL), and accounted for 80 per cent of the total sex differential. The female mortality burden exceeded that of males only for cerebrovascular diseases. Also noteworthy was the toll due to breast cancer (450 thousand YLL), which was almost equal to that of lung cancer (523 thousand YLL). YLL rates were higher for all leading causes for males than they were for females, with the exception of cerebrovascular diseases (Figure 11). The pattern of mortality burden shifted from a predominance of injuries between ages 5 and 44 years, to a gradual increase in chronic diseases (cancers and cardiovascular diseases) among older adults (Tables 13, 14, 15, 16, 17, 18).

**Table 12:** Leading causes of YLL, by sex, all races combined

	**Cause list**	**YLL**	**% total**
	**All males**	**10,529,540**	
1	Ischaemic heart disease	1,806,420	17.2%
2	Lung trachea or bronchial cancer	777,726	7.4%
3	Motor vehicle accidents	701,111	6.7%
4	HIV	575,297	5.5%
5	Self-inflicted	533,874	5.1%
6	Homicide and Violence	486,129	4.6%
7	Cerebrovascular Disease	356,563	3.4%
8	COPD	268,774	2.6%
9	Conditions arising during the perinatal period	259,581	2.5%
10	Diabetes mellitus	220,494	2.1%
11	Cirrhosis of the liver	219,876	2.1%
12	Cancer colon or rectum	212,958	2.0%
13	Lower respiratory infections	202,668	1.9%
14	Congenital Abnomalities	172,399	1.6%
15	Poisoning	168,131	1.6%
16	Inflammatory Cardiac	167,316	1.6%
17	Prostate cancer	160,019	1.5%
18	Lymphomas	134,145	1.3%
19	Hypertension and hypertensive heart disease	127,968	1.2%
20	Leukemias	114,710	1.1%
			
	**Sub-total**	**7,666,158**	**72.8%**
			

	**Cause list**	**YLL**	**% total**

	**All females**	**7,536,559**	
1	Ischaemic heart disease	1,052,325	14.0%
2	Lung trachea or bronchial cancer	523,456	6.9%
3	Breast cancer	450,327	6.0%
4	Cerebrovascular Disease	427,881	5.7%
5	Motor vehicle accidents	325,894	4.3%
6	COPD	257,445	3.4%
7	Diabetes mellitus	230,419	3.1%
8	Conditions arising during the perinatal period	204,550	2.7%
9	Cancer colon or rectum	196,575	2.6%
10	Lower respiratory infections	185,774	2.5%
11	Congenital Abnomalities	146,548	1.9%
12	HIV	143,678	1.9%
13	Homicide and Violence	129,202	1.7%
14	Self-inflicted	127,043	1.7%
15	Ovarian cancer	122,350	1.6%
16	Hypertension and hypertensive heart disease	113,105	1.5%
17	Cirrhosis of the liver	101,712	1.3%
18	Cancer pancreas	99,766	1.3%
19	Lymphomas	98,902	1.3%
20	Inflammatory Cardiac	91,012	1.2%
	**Sub-total**	5,027,963	66.7%

**Table 13:** Five leading causes of mortality burden (YLL) by sex and age, US, 1996

	**Males**					
**Rank**	**All ages**	**YLL**	**% of total**	**0–4**	**YLL**	**% of total**

	Total	10,529,540		Total	652,949	
1	Ischaemic heart disease	1,806,420	17.16%	Perinatal conditions	258,750	39.63%
2	Lung/Trachea/Bronchial cancer	777,726	7.39%	Congenital abnormalities	127,080	19.46%
3	Motor vehicle accidents	701,111	6.66%	Sudden infant death syndrome	61,101	9.36%
4	HIV/AIDS	575,297	5.46%	Motor vehicle accidents	17,505	2.68%
5	Suicide	1,028,947	9.77%	Lower respiratory infections	17,259	2.64%
						

**Rank**	**5–14**	**YLL**	**% of total**	**15–24**	**YLL**	**% of total**

	Total	185,702		Total	845,157	
1	Motor vehicle accidents	42,534	22.90%	Motor vehicle accidents	263,631	31.19%
2	Homicide and violence	15,729	8.47%	Homicide and violence	210,534	24.91%
3	Drowning	12,290	6.62%	Suicide	131,075	15.51%
4	Congenital abnormalities	10,634	5.73%	Drowning	20,807	2.46%
5	Suicide	8,483	4.57%	Poisoning	18,087	2.14%
						

**Rank**	**25–44**	**YLL**	**% of total**	**45–64**	**YLL**	**% of total**

	Total	2,548,913		Total	3,150,157	
1	HIV/AIDS	444,123	17.42%	Ischaemic heart disease	763,334	24.23%
2	Motor vehicle accidents	279,389	10.96%	Lung/Trachea/Bronchial cancer	372,984	11.84%
3	Suicide	277,467	10.89%	Cerebrovascular disease	115,851	3.68%
4	Homicide and violence	207,217	8.13%	Cirrhosis of the liver	113,739	3.61%
5	Ischaemic heart disease	171,437	6.73%	HIV/AIDS	111,874	3.55%
						

**Rank**	**65–74**	**YLL**	**% of total**	**75+**	**YLL**	**% of total**

	Total	1,786,501		Total	1,360,161	
1	Ischaemic heart disease	484,267	27.11%	Ischaemic heart disease	382,495	28.12%
2	Lung/Trachea/Bronchial cancer	253,281	14.18%	Lung/Trachea/Bronchial cancer	104,020	7.65%
3	Chronic obstructive pulmonary disease	103,012	5.77%	Cerebrovascular disease	99,208	7.29%
4	Cerebrovascular disease	84,904	4.75%	Chronic obstructive pulmonary disease	85,031	6.25%
5	Colon/Rectum cancer	62,395	3.49%	Prostate cancer	64,236	4.72%

**Table 14:** Five leading causes of mortality burden (YLL) by sex and age, US, 1996

	**Females**					
**Rank**	**All ages**	**YLL**	**% of total**	**0–4**	**YLL**	**% of total**

	Total	7,536,559		Total	512,861	
1	Ischaemic heart disease	1,052,325	13.96%	Perinatal conditions	203,909	39.76%
2	Lung/Trachea/Bronchial cancer	523,456	6.95%	Congenital abnormalities	109,458	21.34%
3	Breast cancer	450,327	5.98%	Sudden infant death syndrome	41,154	8.02%
4	Cerebrovascular disease	427,881	5.68%	Motor vehicle accidents	14,795	2.88%
5	Motor vehicle accidents	325,894	4.32%	Homicide and violence	13,612	2.65%
						

**Rank**	**5–14**	**YLL**	**% of total**	**15–24**	**YLL**	**% of total**

	Total	123,888		Total	285,089	
1	Motor vehicle accidents	28,952	23.37%	Motor vehicle accidents	117,878	41.35%
2	Homicide and violence	8,134	6.57%	Homicide and violence	32,799	11.50%
3	Congenital abnormalities	7,226	5.83%	Suicide	22,517	7.90%
4	Leukemias	6,120	4.94%	Leukemias	6,726	2.36%
5	Brain cancer	5,611	4.53%	HIV/AIDS	5,830	2.05%
						

**Rank**	**25–44**	**YLL**	**% of total**	**45–64**	**YLL**	**% of total**

	Total	1,192,947		Total	2,053,395	
1	Motor vehicle accidents	117,878	9.88%	Ischaemic heart disease	524,172	25.53%
2	HIV/AIDS	109,310	9.16%	Lung/Trachea/Bronchial cancer	234,241	11.41%
3	Breast cancer	95,818	8.03%	Breast cancer	220,350	10.73%
4	Suicide	67,035	5.62%	Cerebrovascular disease	99,565	4.85%
5	Homicide and violence	60,074	5.04%	Diabetes mellitus	81,366	3.96%
						

**Rank**	**65–74**	**YLL**	**% of total**	**75+**	**YLL**	**% of total**

	Total	1,503,549		Total	1,864,830	
1	Ischaemic heart disease	277,053	18.43%	Ischaemic heart disease	459,396	24.63%
2	Lung/Trachea/Bronchial cancer	172,116	11.45%	Cerebrovascular disease	186,468	10.00%
3	Chronic obstructive pulmonary disease	97,759	6.50%	Chronic obstructive pulmonary disease	88,838	4.76%
4	Cerebrovascular disease	90,698	6.03%	Lung/Trachea/Bronchial cancer	84,492	4.53%
5	Breast cancer	82,227	5.47%	Lower respiratory infections	83,467	4.48%

**Table 15:** Leading causes of YLL by sex and race – Whites

	**Cause list**	**YLL**	**% total**
	**Total White males**	**8,293,920**	
1	Ischaemic heart disease	1,584,087	19.1%
2	Lung trachea or bronchial cancer	656,850	7.9%
3	Road Traffic Accidents	573,953	6.9%
4	Self-inflicted	469,430	5.7%
5	HIV	334,425	4.0%
6	Cerebrovascular Disease	276,544	3.3%
7	COPD	244,401	2.9%
8	Homicide and Violence	221,000	2.7%
9	Cirrhosis of the liver	183,600	2.2%
10	Cancer colon or rectum	180,184	2.2%
11	Diabetes mellitus	173,405	2.1%
12	Conditions arising during the perinatal period	158,462	1.9%
13	Lower respiratory infections	158,231	1.9%
14	Congenital Abnomalities	135,970	1.6%
15	Poisoning	134,929	1.6%
16	Prostate cancer	128,058	1.5%
17	Inflammatory Cardiac	121,438	1.5%
18	Lymphomas	116,804	1.4%
19	Leukemias	99,137	1.2%
20	Cancer pancreas	89,780	1.1%
			
	**Sub-total**	**6,040,688**	**72.8%**
			

	**Cause list**	**YLL**	**% total**

	**Total White females**	**6,018,361**	
1	Ischaemic heart disease	895,819	14.9%
2	Lung trachea or bronchial cancer	460,651	7.7%
3	Breast cancer	370,855	6.2%
4	Cerebrovascular Disease	342,620	5.7%
5	Road Traffic Accidents	268,996	4.5%
6	COPD	240,803	4.0%
7	Diabetes mellitus	169,449	2.8%
8	Cancer colon or rectum	163,068	2.7%
9	Lower respiratory infections	153,465	2.5%
10	Conditions arising during the perinatal period	124,599	2.1%
11	Congenital Abnomalities	112,925	1.9%
12	Self-inflicted	112,621	1.9%
13	Ovarian cancer	108,262	1.8%
14	Lymphomas	87,677	1.5%
15	Cancer pancreas	83,238	1.4%
16	Cirrhosis of the liver	81,843	1.4%
17	Dementia and other degenerative and hereditary CNS disorders	76,867	1.3%
18	Leukemias	74,069	1.2%
19	Homicide and Violence	72,469	1.2%
20	Hypertension and hypertensive heart disease	72,258	1.2%
			
	**Sub-total**	**4,072,553**	**67.7%**

**Table 16:** Leading causes of YLL by sex and race – Blacks

	**Cause list**	**YLL**	**% total**
	**Total Black males**	**1,978,704**	
1	Homicide and Violence	250,257	12.6%
2	HIV	234,400	11.8%
3	Ischaemic heart disease	189,031	9.6%
4	Lung trachea or bronchial cancer	109,191	5.5%
5	Motor vehicle accidents	99,734	5.0%
6	Conditions arising during the perinatal period	92,854	4.7%
7	Cerebrovascular Disease	69,225	3.5%
8	Self-inflicted	47,940	2.4%
9	Hypertension and hypertensive heart disease	44,841	2.3%
10	Inflammatory Cardiac	41,409	2.1%
11	Diabetes mellitus	40,795	2.1%
12	Lower respiratory infections	38,675	2.0%
13	Congenital Abnomalities	30,182	1.5%
14	Cirrhosis of the liver	30,182	1.5%
15	Poisoning	30,101	1.5%
16	Prostate cancer	30,075	1.5%
17	Cancer colon or rectum	28,380	1.4%
18	COPD	21,382	1.1%
19	Sudden Infant Death Syndrome	19,017	1.0%
20	Nephritis or nephrosis	17,092	0.9%
			
	**Sub-total**	**1,464,762**	**74.0%**
			

	**Cause list**	**YLL**	**% total**

	**Total Black females**	**1,342,205**	
1	Ischaemic heart disease	141,305	10.5%
2	HIV	89,973	6.7%
3	Cerebrovascular Disease	74,323	5.5%
4	Conditions arising during the perinatal period	73,969	5.5%
5	Breast cancer	70,421	5.2%
6	Lung trachea or bronchial cancer	55,561	4.1%
7	Diabetes mellitus	54,669	4.1%
8	Homicide and Violence	52,280	3.9%
9	Motor vehicle accidents	42,499	3.2%
10	Hypertension and hypertensive heart disease	39,005	2.9%
11	Cancer colon or rectum	28,896	2.2%
12	Congenital Abnomalities	28,218	2.1%
13	Lower respiratory infections	28,108	2.1%
14	Inflammatory Cardiac	25,707	1.9%
15	Nephritis or nephrosis	15,811	1.2%
16	Cirrhosis of the liver	15,701	1.2%
17	COPD	14,858	1.1%
18	Cancer pancreas	14,132	1.1%
19	Sudden Infant Death Syndrome	13,841	1.0%
20	Cancer cervix	13,786	1.0%
			
	**Sub-total**	**893,064**	**66.5%**

**Table 17:** Leading causes of YLL by sex and race – American Indians

	**Cause list**	**YLL**	**% total**
	**Total American Indian males**	**83,713**	
1	Motor vehicle accidents	13,665	16.3%
2	Ischaemic heart disease	8,804	10.5%
3	Self-inflicted	6,472	7.7%
4	Homicide and Violence	5,287	6.3%
5	Cirrhosis of the liver	3,982	4.8%
6	Alcohol use	2,849	3.4%
7	Lung trachea or bronchial cancer	2,792	3.3%
8	Diabetes mellitus	2,610	3.1%
9	Lower respiratory infections	2,168	2.6%
10	Conditions arising during the perinatal period	2,078	2.5%
11	Poisoning	2,009	2.4%
12	HIV	1,952	2.3%
13	Cerebrovascular Disease	1,919	2.3%
14	Congenital Abnomalities	1,658	2.0%
15	Drowning	1,489	1.8%
16	Sudden Infant Death Syndrome	1,239	1.5%
17	Inflammatory Cardiac	1,193	1.4%
18	Cancer colon or rectum	816	1.0%
19	Falls	755	0.9%
20	COPD	719	0.9%
			
	**Sub-total**	**64,456**	**77.0%**
			

	**Cause list**	**YLL**	**% total**

	**Total Amerian Indian females**	**54,732**	
1	Motor vehicle accidents	6,429	11.7%
2	Ischaemic heart disease	4,247	7.8%
3	Cirrhosis of the liver	3,311	6.0%
4	Diabetes mellitus	2,970	5.4%
5	Cerebrovascular Disease	2,057	3.8%
6	Breast cancer	1,901	3.5%
7	Lung trachea or bronchial cancer	1,788	3.3%
8	Self-inflicted	1,786	3.3%
9	Homicide and Violence	1,534	2.8%
10	Conditions arising during the perinatal period	1,531	2.8%
11	Lower respiratory infections	1,523	2.8%
12	Congenital Abnomalities	1,182	2.2%
13	Alcohol use	1,156	2.1%
14	Sudden Infant Death Syndrome	907	1.7%
15	Cancer colon or rectum	851	1.6%
16	Poisoning	809	1.5%
17	COPD	731	1.3%
18	Ovarian cancer	678	1.2%
19	Inflammatory Cardiac	651	1.2%
20	Drowning	633	1.2%
			
	**Sub-total**	**36,675**	**67.0%**

**Table 18:** Leading causes of YLL by sex and race – Asians

	**Cause list**	**YLL**	**% total**
	**Total Asian males**	**173,201**	
1	Ischaemic heart disease	24,497	14.1%
2	Motor vehicle accidents	13,759	7.9%
3	Self-inflicted	10,032	5.8%
4	Homicide and Violence	9,584	5.5%
5	Lung trachea or bronchial cancer	8,892	5.1%
6	Cerebrovascular Disease	8,875	5.1%
7	Cancer liver	6,330	3.7%
8	Conditions arising during the perinatal period	6,188	3.6%
9	Congenital Abnomalities	4,590	2.7%
10	HIV	4,520	2.6%
11	Diabetes mellitus	3,684	2.1%
12	Lower respiratory infections	3,593	2.1%
13	Cancer colon or rectum	3,578	2.1%
14	Inflammatory Cardiac	3,276	1.9%
15	Drowning	3,256	1.9%
16	Cancer stomach	2,935	1.7%
17	Leukemias	2,601	1.5%
18	Hypertension and hypertensive heart disease	2,365	1.4%
19	COPD	2,272	1.3%
20	Cirrhosis of the liver	2,113	1.2%
			
	**Sub-total**	**126,941**	**73.3%**
			

	**Cause list**	**YLL**	**% total**

	**Total Asian females**	**121,261**	
1	Ischaemic heart disease	10,953	9.0%
2	Cerebrovascular Disease	8,881	7.3%
3	Road Traffic Accidents	7,970	6.6%
4	Breast cancer	7,150	5.9%
5	Lung trachea or bronchial cancer	5,455	4.5%
6	Conditions arising during the perinatal period	4,451	3.7%
7	Congenital Abnomalities	4,223	3.5%
8	Self-inflicted	4,112	3.4%
9	Cancer colon or rectum	3,761	3.1%
10	Diabetes mellitus	3,331	2.7%
11	Homicide and Violence	2,920	2.4%
12	Cancer stomach	2,873	2.4%
13	Ovarian cancer	2,776	2.3%
14	Lower respiratory infections	2,677	2.2%
15	Cancer liver	2,210	1.8%
16	Leukemias	2,126	1.8%
17	Cancer cervix	1,892	1.6%
18	Cancer pancreas	1,888	1.6%
19	Inflammatory Cardiac	1,602	1.3%
20	Lymphomas	1,479	1.2%
			
	**Sub-total**	**82,729**	**68.2%**

#### Patterns by race

The share of YLL due to communicable diseases (which include HIV/AIDS), maternal causes, perinatal and nutritional conditions was twofold larger for Blacks (20 per cent) than it was for any of the other races. Injuries predominated among American Indians, causing one third of the total mortality burden, and one fifth or less in the other races.

**Figure 12:**
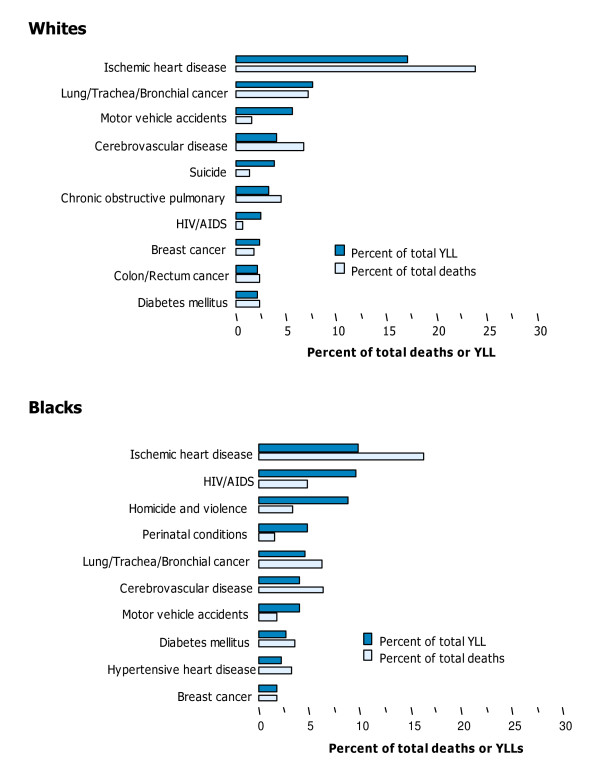
Ten leading causes of mortality burden (YLL) and death, as a per cent of total, by race, US, 1996.

**Figure 13:**
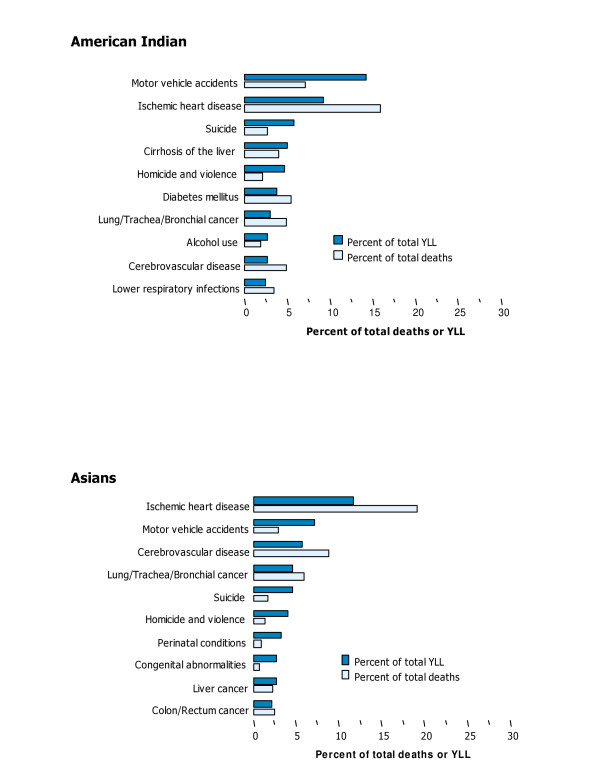
Ten leading causes of mortality burden (YLL) and death, as a per cent of total, by race, US, 1996.

The mortality burden was highest for Blacks and lowest for Asians, for both sexes and all ages. A few causes contributed about one third of total YLL in each race. These were IHD, lung cancer and motor vehicle accidents for Whites; IHD, HIV/AIDS and homicide and violence for Blacks; motor vehicle accidents, IHD and self-inflicted injuries for American Indians; and IHD, motor vehicle accidents, cerebrovascular diseases, and lung cancer for Asians (Tables 15, 16, 17, 18; Figures 12, 13).

#### Comparative rankings of mortality burden in the United States and selected industrialized countries

**Figure 14:**
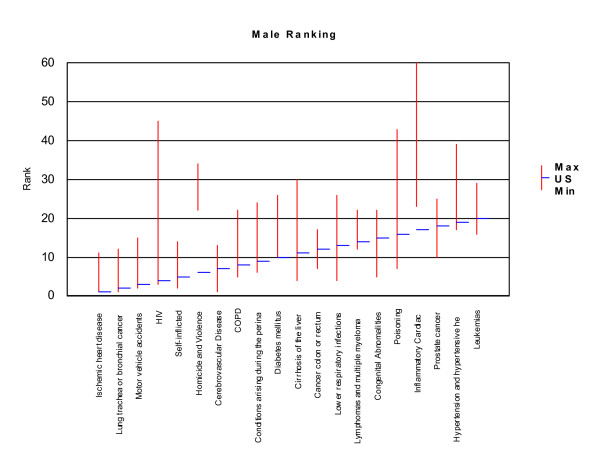
**Comparative rankings for the twenty leading causes of YLL for males, US compared with selected non-US OECD countries**. Note: Vertical red bars indicate the range between minimum and maximum rankings observed in the selected OECD countries (excluding the US). Blue horizontal lines indicate rankings for the US. The ten selected non-US OECD countries are: Australia, Canada, France, Germany, Greece, Italy, Japan, Netherlands, Spain and the United Kingdom.

**Figure 15:**
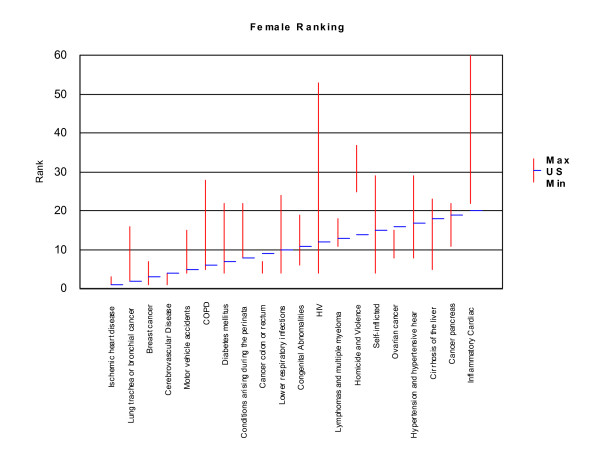
**Comparative rankings for the twenty leading causes of YLL for females, US compared with selected non-US OECD countries**. Note: Vertical red bars indicate the range between minimum and maximum rankings observed in the selected OECD countries (excluding the US). Blue horizontal lines indicate rankings for the US. The ten selected OECD countries are: Australia, Canada, France, Germany, Greece, Italy, Japan, Netherlands, Spain and the United Kingdom.

Relative YLL rankings observed in the United States and in ten selected industrialized countries (Australia, Canada, France, Germany, Greece, Italy, Japan, Netherlands, Spain and the United Kingdom) were similar for IHD, lung cancer and motor vehicle accidents for males, and IHD, breast cancer and cerebrovascular diseases for females, which ranked among the top five leading causes of YLL in all countries. In contrast, the range of rankings observed was widest for HIV/AIDS and inflammatory cardiac diseases (cardiomyopathy and endocarditis) for both sexes, appearing to cause a very high mortality burden in some countries, and a much lower mortality burden in others (Figures 14 and 15). The wide range observed for these two conditions may point to real differences in causes of death and their important risk factors, but may also indicate differences in cause of death reporting practices, particularly for inflammatory cardiac disease, which ultimately leads to congestive heart failure, and may not have been diagnosed as the underlying cause.

#### US YLL rankings by race compared to selected industrialized countries

**Figure 16:**
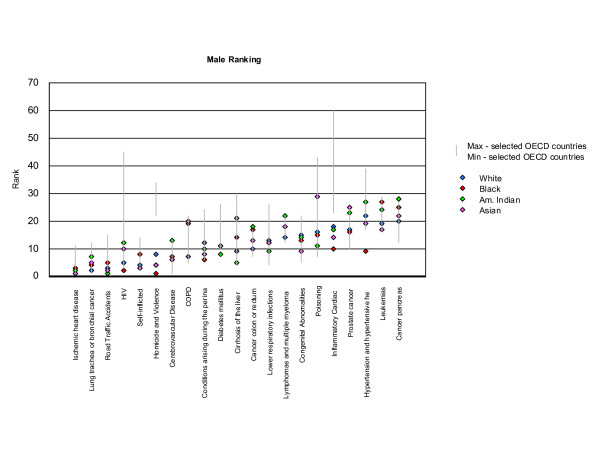
**YLL rankings by race in the US compared with selected non-US OECD countries, males**. Note: Vertical red bars indicate the range between minimum and maximum rankings observed in the selected OECD countries (excluding the US). Blue horizontal lines indicate rankings for the US. The ten selected OECD countries are: Australia, Canada, France, Germany, Greece, Italy, Japan, Netherlands, Spain and the United Kingdom

**Figure 17:**
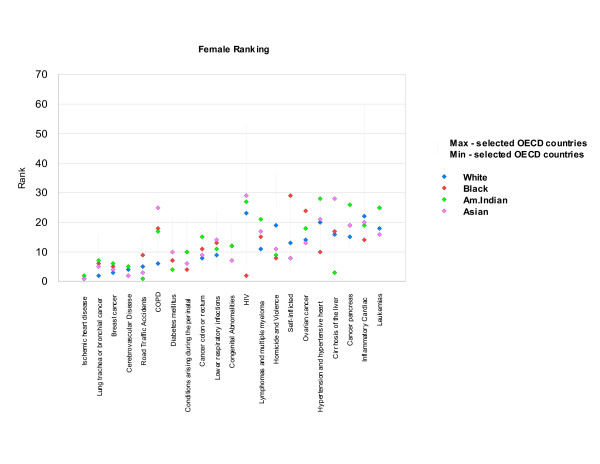
**YLL rankings by race in the US compared with non-US OECD countries, females**. Note: Vertical red bars indicate the range between minimum and maximum rankings observed in the selected OECD countries (excluding the US). Blue horizontal lines indicate rankings for the US. The ten selected OECD countries are: Australia, Canada, France, Germany, Greece, Italy, Japan, Netherlands, Spain and the United Kingdom

The higher share of YLL due to homicide and violence in the general population in the United States was observed in all races and both sexes (Figures 16 and 17). YLL rankings for all races and both sexes fell outside those observed in other countries for inflammatory cardiac diseases, pointing to a higher burden in the United States. Rankings for cerebrovascular diseases, on the other hand, pointed to a lower share of burden for males in all races. YLL rates exceeded rates for other countries for HIV/AIDS, homicide and violence, and inflammatory cardiac disease in both sexes, confirming findings based on the comparison of YLL rankings (Tables 19 and 20).

**Table 19:** Twenty leading causes of YLL in the United States: comparison of YLL rates per 100,000 between the United States and selected countries – Male

**Twenty leading causes of YLL in the United States**	**United States**	**Australia**	**Canada**	**France**	**Germany**	**Greece**	**Italy**	**Japan**	**Netherlands**	**Spain**	**United Kingdom**
IHD	1,392	269	950	578	1,249	1,337	974	504	942	826	2,154
Trachea, bronchus, lung cancer	599	105	469	666	611	756	683	387	595	638	714
MVA	547	106	292	515	325	690	438	242	233	550	350
HIV/AIDS	443	9	62	60	26	11	67	1	31	176	18
Self-inflicted	411	140	414	459	367	101	184	582	221	218	410
homicide and violence	375	15	57	21	19	37	38	14	38	35	55
Cerebroavascular disease	275	72	205	307	377	723	421	546	319	356	566
COPD	207	51	145	142	182	88	168	57	234	247	371
Perinatal conditions	200	43	115	120	87	115	90	39	116	78	215
Diabetes	170	33	146	97	125	54	144	76	120	93	99
Cirrhosis of the liver	169	25	104	281	372	85	228	161	85	195	258
Colon and rectum	164	68	187	213	267	160	225	231	220	235	338
Lower respiratory infections	156	19	66	134	99	49	80	281	152	97	524
Congenital anomalies	133	33	95	101	78	104	78	66	113	86	125
poisonings	130	51	87	12	28	104	11	12	30	71	111
Inflammatory heart disease	129	21	52	68	116	3	67	45	65	87	84
Prostate	123	41	118	145	142	119	110	50	151	121	241
Lymphoma and multiple myeloma	103	36	125	114	108	92	130	77	128	106	176
Hypertesive heart disease	99	7	21	51	77	48	117	15	24	38	54
Leukemia	88	25	82	91	87	105	100	70	78	86	115

**Table 20:** Twenty leading causes of YLL in the United States: comparison of YLL rates per 100,000 between the United States and selected countries – Female

**Twenty leading causes of YLL in the United States**	**United States**	**Australia**	**Canada**	**France**	**Germany**	**Greece**	**Italy**	**Japan**	**Netherlands**	**Spain**	**United Kingdom**
IHD	777	462	475	228	753	570	491	562	466	383	868
Trachea, bronchus, lung cancer	386	186	354	140	201	125	154	328	288	86	357
Breast cancer	332	285	326	389	404	313	363	396	457	288	492
Cerebroavascular disease	316	253	213	253	405	826	426	943	378	362	510
MVA	241	125	124	157	105	178	117	167	71	151	73
COPD	190	108	114	67	90	41	66	39	152	57	227
Diabetes	170	80	109	76	123	55	151	102	115	108	66
Perinatal conditions	151	108	87	89	64	75	70	70	94	63	115
Colon and rectum	145	164	145	155	217	153	171	381	189	174	201
Lower respiratory infections	137	50	56	91	79	34	61	418	147	71	412
Congenital anomalies	108	89	73	71	64	76	59	136	100	73	84
HIV/AIDS	106	2	13	16	6	2	17	0	8	43	4
homicide and violence	95	32	21	12	16	11	11	25	19	13	20
sel-inflicted	94	120	119	155	106	27	55	473	107	63	90
Ovarian cancer	90	69	79	90	114	82	88	148	99	74	144
Hypertesive heart disease	83	27	21	50	94	50	137	34	27	53	32
Cirrhosis of the liver	75	32	45	113	156	28	115	99	44	71	113
Cancer pancreas	74	56	67	66	94	73	85	187	78	62	84
Lymphoma and multiple myeloma	73	88	97	80	83	71	103	126	90	84	110
Inflammatory heart disease	67	31	29	24	44	3	30	53	42	45	32

The largest differentials between races for males pertained to COPD, cirrhosis of the liver, poisoning, hypertension and hypertensive heart disease. Rankings indicate a higher mortality burden due to COPD and cirrhosis of the liver in American Indians; hypertension and hypertensive heart disease in Blacks; and a lower mortality burden for poisoning in Asians. The largest rank differentials between races were observed in females for HIV/AIDS, self-inflicted injuries, COPD, hypertension and hypertensive heart disease, and cirrhosis of the liver. Rankings for Black females pointed to a higher mortality burden for HIV/AIDS, hypertension and hypertensive heart disease, and a lower mortality burden for self-inflicted injuries and ovarian cancer compared to White, American Indian and Asian females.

### Morbidity burden

#### Leading causes of YLD

**Figure 18:**
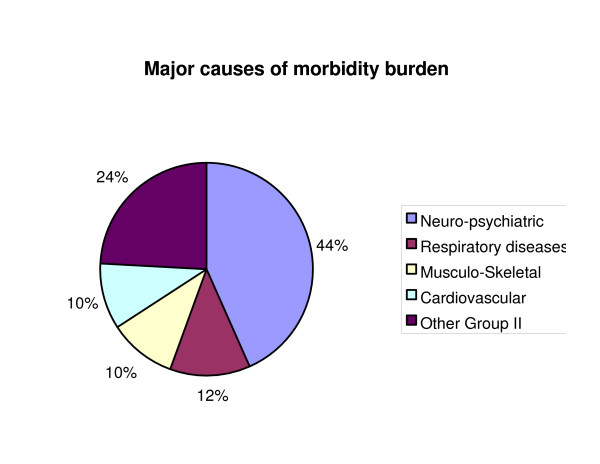
Distribution of YLD for non-communicable cause groupings.

**Figure 19:**
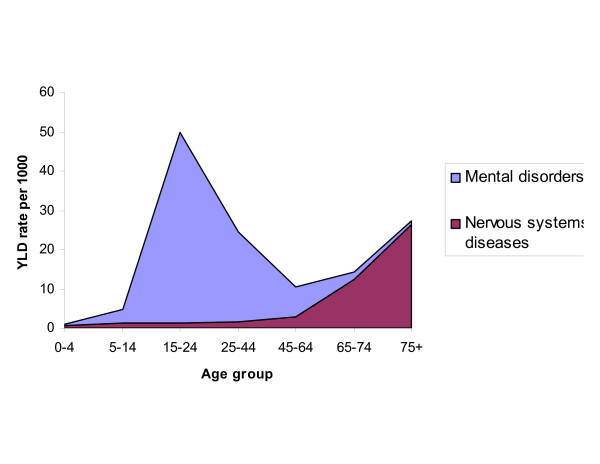
Age patterns of mental disorders and diseases of the nervous system.

Non-fatal health outcomes resulted in 15 million YLD, which was only slightly less than the mortality burden (17 million YLL). For neuropsychiatric conditions, musculoskeletal conditions, chronic respiratory diseases, YLD contributed more than YLL. Neuropsychiatric conditions were the predominant cause of disability, causing 44 per cent of total YLD, regardless of sex and race (Figure 18). They comprise a wide array of conditions, sub-divided into mental disorders and diseases of the nervous system (DSM IV). Mental disorders include mood disorders (unipolar major depression, bipolar disorders), schizophrenia, anxiety disorders (PTSD, obsessive compulsive disorders, and panic disorders) affecting mostly young adults, and substance related disorders (alcohol and drug use) that increase in older adults. Nervous system disorders include Alzheimer's disease and other degenerative and hereditary CNS disorders, Parkinson's disease, epilepsy, and multiple sclerosis (Figure 19).

Unipolar major depression, alcohol use, osteoarthritis, dementia and other degenerative disorders of the CNS and cerebrovascular diseases were the five leading causes of YLD (Table 21). Unipolar major depression and alcohol use combined (2.4 million YLD) caused 16 per cent of total YLD, which represented almost the same burden as IHD (2.9 million YLL).

**Table 21:** Twenty leading causes of YLD, both sexes and all races combined

**Rank**		**YLD**	**% of total**
	Total	15,024,113	
			
1	Unipolar major depression	1,370,070	9.1%
2	Alcohol use	1,037,529	6.9%
3	Osteoarthritis	940,612	6.3%
4	Dementia and other degenerative and hereditary CNS disorders	755,925	5.0%
5	COPD	727,272	4.8%
6	Cerebrovascular Disease	725,844	4.8%
7	Asthma	593,233	3.9%
8	Drug use	504,718	3.4%
9	Diabetes mellitus	495,377	3.3%
10	Congenital Abnomalities	443,004	2.9%
11	Motor vehicle accidents	366,273	2.4%
12	Bipolar disorder	363,298	2.4%
13	Schizophrenia	315,720	2.1%
14	Ischaemic heart disease	275,988	1.8%
15	PTSD	260,337	1.7%
16	Panic disorder	259,904	1.7%
17	HIV	237,443	1.6%
18	Falls	221,036	1.5%
19	Rheumatoid arthritis	189,421	1.3%
20	Obsessive-compulsive disorders	169,067	1.1%
			
	**Sub-total**	10,252,070	68.2%

#### Sex and age patterns

Sex differentials were much smaller for YLD than for YLL. The morbidity burden was slightly larger for females (7.7 million YLD) than for males (7.3 million YLD). The five leading causes of YLD were alcohol use, unipolar major depression, osteoarthritis, drug use, and chronic obstructive pulmonary disease (COPD) for males, and unipolar major depression, osteoarthritis, dementia and other degenerative and hereditary CNS disorders, and alcohol use for females (Table 22).

**Table 22:** Twenty leading causes of YLD by sex, US 1996

	**MALES**		
**Rank**		**YLD**	**% total YLD**
	**Total**	**7,330,853**	
1	Alcohol use	651,223	8.9%
2	Unipolar major depression	469,861	6.4%
3	Osteoarthritis	434,200	5.9%
4	Drug use	384,319	5.2%
5	COPD	372,927	5.1%
6	Dementia and other degenerative and hereditary CNS disorders	332,046	4.5%
7	Cerebrovascular Disease	317,366	4.3%
8	Asthma	272,898	3.7%
9	Congenital Abnomalities	237,988	3.2%
10	Motor vehicle accidents	232,687	3.2%
11	Diabetes mellitus	221,557	3.0%
12	Bipolar disorder	198,308	2.7%
13	HIV	188,519	2.6%
14	Schizophrenia	166,988	2.3%
15	Ischaemic heart disease	151,764	2.1%
16	Falls	132,485	1.8%
17	Obsessive-compulsive disorders	88,623	1.2%
18	Homicide and Violence	81,588	1.1%
19	Prostate cancer	78,870	1.1%
20	Panic disorder	77,701	1.1%
			
	**sub-total**	**5,091,918**	**69.5%**
			

	**FEMALES**		

**Rank**		**YLD**	**% total YLD**
	**Total**	**7,693,260**	
1	Unipolar major depression	900,209	11.7%
2	Osteoarthritis	506,412	6.6%
3	Dementia and other degenerative and hereditary CNS disorders	423,878	5.5%
4	Cerebrovascular Disease	408,478	5.3%
5	Alcohol use	386,306	5.0%
6	COPD	354,345	4.6%
7	Asthma	320,336	4.2%
8	Diabetes mellitus	273,821	3.6%
9	Congenital Abnomalities	205,015	2.7%
10	PTSD	193,533	2.5%
11	Panic disorder	182,203	2.4%
12	Bipolar disorder	164,990	2.1%
13	Schizophrenia	148,732	1.9%
14	Motor vehicle accidents	133,586	1.7%
15	Rheumatoid arthritis	131,758	1.7%
16	STD's excluding HIV	125,418	1.6%
17	Ischaemic heart disease	124,224	1.6%
18	Drug use	120,399	1.6%
19	Maternal Conditions	105,155	1.4%
20	Falls	88,551	1.2%
			
	**sub-total**	**5,297,349**	**68.9%**

The largest sex differentials pertained to the leading cause of YLD: alcohol use for males and unipolar major depression for females. The burden due to unipolar depression was almost double for females (900 thousand YLD) than it was for males (470 thousand YLD), whereas the burden due to alcohol was double for males (651 thousand YLD) than it was for females (386 thousand YLD). Together, alcohol use and unipolar depression caused 15 per cent of total YLD for males, and 17 per cent for females.

The share of total YLD due to neuropsychiatric conditions for males exceeded that for females. Morbidity due to substance abuse (alcohol and drug) in males was not entirely offset by the preponderance of mood and anxiety disorders in females. The slightly higher share of dementia and other degenerative and hereditary CNS disorders in females resulted from their higher life expectancy.

Major causes contributing to the morbidity burden changed with age. Mental disorders and injuries affected mostly young adults, whereas nervous system disorders, musculoskeletal conditions, cardiovascular diseases, and diabetes increased with age and were predominant among older adults. Chronic respiratory conditions affected all age groups. Congenital anomalies represented half of the non-fatal burden below age 5.

#### Patterns by race

Alcohol use was the leading cause of YLD for males in all races, with the exception of Asian males for whom unipolar depression was the leading cause. Unipolar major depression was the leading cause for females of all races, with the exception of American Indian females for whom alcohol use was the leading cause (Tables 23, 24, 25, 26).

**Table 23:** Leading causes of YLD – Whites

	**WHITE MALES**		
**Rank**	**Cause list**	**YLD**	**% total YLD**
	**Total YLD**	**5,963,710**	
1	Alcohol use	488,341	8.2%
2	Unipolar major depression	390,121	6.5%
3	Osteoarthritis	378,589	6.3%
4	Drug use	324,878	5.4%
5	COPD	319,049	5.3%
6	Dementia and other degenerative and hereditary CNS disorders	293,857	4.9%
7	Cerebrovascular Disease	258,909	4.3%
8	Asthma	216,831	3.6%
9	Motor vehicle accidents	195,590	3.3%
10	Congenital Abnomalities	189,083	3.2%
11	Diabetes mellitus	174,663	2.9%
12	Bipolar disorder	161,700	2.7%
13	Schizophrenia	134,190	2.3%
14	Ischaemic heart disease	130,502	2.2%
15	Falls	113,345	1.9%
16	HIV	111,533	1.9%
17	Obsessive-compulsive disorders	71,313	1.2%
18	Prostate cancer	68,023	1.1%
19	Panic disorder	63,735	1.1%
20	PTSD	55,968	0.9%
			
	**sub-total**	**4,140,220**	**69.4%**
			

	**WHITE FEMALES**		

**Rank**	**Cause list**	**YLD**	**% total YLD**
	**Total YLD**	**6,234,020**	
1	Unipolar major depression	736,746	11.8%
2	Osteoarthritis	439,876	7.1%
3	Dementia and other degenerative and hereditary CNS disorders	375,458	6.0%
4	Cerebrovascular Disease	323,173	5.2%
5	COPD	307,237	4.9%
6	Alcohol use	291,334	4.7%
7	Asthma	253,904	4.1%
8	Diabetes mellitus	210,058	3.4%
9	Congenital Abnomalities	162,162	2.6%
10	PTSD	159,120	2.6%
11	Panic disorder	145,888	2.3%
12	Bipolar disorder	131,835	2.1%
13	Schizophrenia	118,134	1.9%
14	Rheumatoid arthritis	110,674	1.8%
15	Motor vehicle accidents	109,754	1.8%
16	STD's excluding HIV	101,298	1.6%
17	Ischaemic heart disease	100,510	1.6%
18	Drug use	98,319	1.6%
19	Maternal Conditions	82,750	1.3%
20	Falls	76,404	1.2%
			
	**Sub-total**	**4,334,635**	**69.5%**

**Table 24:** Leading causes of YLD – Blacks

	**BLACK MALES**		
**Rank**	**Simple cause list**	**YLD**	**% total YLD**
	**Total YLD**	**1,086,407**	
1	Alcohol use	135,346	12.5%
2	HIV	73,292	6.7%
3	Unipolar major depression	57,653	5.3%
4	Cerebrovascular Disease	49,191	4.5%
5	Drug use	44,504	4.1%
6	Asthma	42,295	3.9%
7	Diabetes mellitus	39,783	3.7%
8	COPD	38,739	3.6%
9	Osteoarthritis	38,641	3.6%
10	Congenital Abnomalities	36,030	3.3%
11	Road Traffic Accidents	31,173	2.9%
12	Dementia and other degenerative and hereditary CNS disorders	28,292	2.6%
13	Homicide and Violence	26,846	2.5%
14	Bipolar disorder	26,799	2.5%
15	Schizophrenia	24,323	2.2%
16	Ischaemic heart disease	18,148	1.7%
17	Falls	15,894	1.5%
18	Diarrhoeal diseases	15,602	1.4%
19	Inflammatory Cardiac	13,178	1.2%
20	Obsessive-compulsive disorders	12,818	1.2%
			
	**sub-total**	**768,546**	**70.7%**
			

	**BLACK FEMALES**		

**Rank**	**Simple cause list**	**YLD**	**% total YLD**
	**Total YLD**	**1,145,131**	
1	Unipolar major depression	119,471	10.4%
2	Alcohol use	74,248	6.5%
3	Cerebrovascular Disease	74,179	6.5%
4	Diabetes mellitus	54,409	4.8%
5	Asthma	50,869	4.4%
6	Osteoarthritis	48,521	4.2%
7	Dementia and other degenerative and hereditary CNS disorders	37,519	3.3%
8	HIV	31,719	2.8%
9	Congenital Abnomalities	31,654	2.8%
10	COPD	31,132	2.7%
11	PTSD	27,508	2.4%
12	Panic disorder	26,916	2.4%
13	Bipolar disorder	24,649	2.2%
14	Schizophrenia	22,823	2.0%
15	Ischaemic heart disease	21,685	1.9%
16	Road Traffic Accidents	19,754	1.7%
17	STD's excluding HIV	17,688	1.5%
18	Drug use	17,686	1.5%
19	Maternal Conditions	16,770	1.5%
20	Rheumatoid arthritis	15,521	1.4%
			
	**sub-total**	**764,722**	**66.8%**

**Table 25:** Leading causes of YLD – American Indians

	**AMERICAN INDIAN MALES**		
**Rank**	**Simple cause list**	**YLD**	**% total YLD**
	**Total YLD**	**77,508**	
1	Alcohol use	22,997	29.7%
2	Unipolar major depression	4,160	5.4%
3	Drug use	3,042	3.9%
4	Osteoarthritis	2,940	3.8%
5	Asthma	2,745	3.5%
6	COPD	2,708	3.5%
7	Congenital Abnomalities	2,467	3.2%
8	Cerebrovascular Disease	2,014	2.6%
9	Bipolar disorder	1,948	2.5%
10	Road Traffic Accidents	1,902	2.5%
11	Schizophrenia	1,796	2.3%
12	Dementia and other degenerative and hereditary CNS disorders	1,775	2.3%
13	Diabetes mellitus	1,595	2.1%
14	HIV	1,190	1.5%
15	Cirrhosis of the liver	1,082	1.4%
16	Fires	1,027	1.3%
17	Obsessive-compulsive disorders	940	1.2%
18	Falls	921	1.2%
19	Ischaemic heart disease	881	1.1%
20	Panic disorder	743	1.0%
			
	**sub-total**	**58,874**	**76.0%**
			

	**AMERICAN INDIAN FEMALES**		

**Rank**	**Simple cause list**	**YLD**	**% total YLD**
	**Total YLD**	**78,522**	
1	Alcohol use	19,417	24.7%
2	Unipolar major depression	7,655	9.7%
3	Asthma	3,233	4.1%
4	Osteoarthritis	3,020	3.8%
5	COPD	2,692	3.4%
6	Cerebrovascular Disease	2,251	2.9%
7	Congenital Abnomalities	2,182	2.8%
8	Dementia and other degenerative and hereditary CNS disorders	2,006	2.6%
9	Diabetes mellitus	1,895	2.4%
10	Panic disorder	1,851	2.4%
11	Bipolar disorder	1,717	2.2%
12	Schizophrenia	1,625	2.1%
13	PTSD	1,251	1.6%
14	Maternal Conditions	1,178	1.5%
15	STD's excluding HIV	1,157	1.5%
16	Road Traffic Accidents	1,115	1.4%
17	Rheumatoid arthritis	958	1.2%
18	Drug use	925	1.2%
19	Cirrhosis of the liver	918	1.2%
20	Obsessive-compulsive disorders	865	1.1%
			
	**Sub-total**	**57,910**	**73.8%**

**Table 26:** Leading causes of YLD – Asians

	**ASIAN MALES**		
**Rank**	**Simple cause list**	**YLD**	**% total YLD**
	**Total YLD**	**203,229**	
1	Unipolar major depression	17,926	8.8%
2	Osteoarthritis	14,029	6.9%
3	COPD	12,431	6.1%
4	Drug use	11,895	5.9%
5	Asthma	11,027	5.4%
6	Congenital Abnomalities	10,408	5.1%
7	Dementia and other degenerative and hereditary CNS disorders	8,122	4.0%
8	Bipolar disorder	7,862	3.9%
9	Cerebrovascular Disease	7,251	3.6%
10	Schizophrenia	6,679	3.3%
11	Diabetes mellitus	5,516	2.7%
12	Alcohol use	4,539	2.2%
13	Road Traffic Accidents	4,022	2.0%
14	Obsessive-compulsive disorders	3,552	1.7%
15	Panic disorder	2,994	1.5%
16	HIV	2,505	1.2%
17	Epilepsy	2,470	1.2%
18	Falls	2,325	1.1%
19	Ischaemic heart disease	2,234	1.1%
20	Diarrhoeal diseases	2,057	1.0%
			
	**Sub-total**	**139,843**	**68.8%**
			

	**ASIAN FEMALES**		

**Rank**	**Simple cause list**	**YLD**	**% total YLD**
	**Total YLD**	**235,588**	
1	Unipolar major depression	36,337	15.4%
2	Osteoarthritis	14,995	6.4%
3	COPD	13,285	5.6%
4	Asthma	12,329	5.2%
5	Congenital Abnomalities	9,018	3.8%
6	Dementia and other degenerative and hereditary CNS disorders	8,896	3.8%
7	Cerebrovascular Disease	8,876	3.8%
8	Panic disorder	7,548	3.2%
9	Diabetes mellitus	7,458	3.2%
10	Bipolar disorder	6,788	2.9%
11	Schizophrenia	6,150	2.6%
12	PTSD	5,654	2.4%
13	STD's excluding HIV	5,275	2.2%
14	Rheumatoid arthritis	4,605	2.0%
15	Maternal Conditions	4,456	1.9%
16	Drug use	3,469	1.5%
17	Obsessive-compulsive disorders	3,347	1.4%
18	Road Traffic Accidents	2,963	1.3%
19	Epilepsy	2,222	0.9%
20	Diarrhoeal diseases	1,745	0.7%
			
	**Sub-total**	**165,417**	**70.2%**

Differentials in patterns of neuropsychiatric disorders by race were dominated by the large excess morbidity burden caused by substance abuse among American Indians, which accounted for half of total YLD, compared to approximately one third in the other race groups.

**Figure 20:**
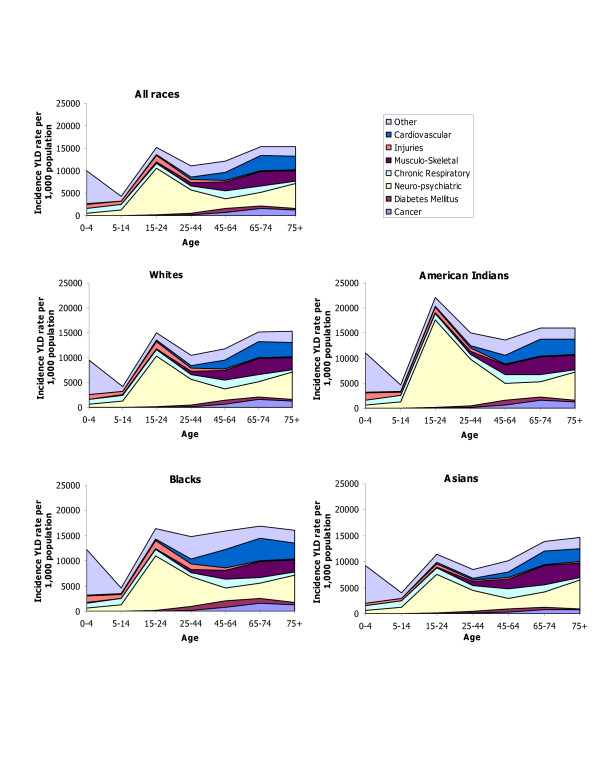
Patterns of YLD by age and race, US 1996.

The distribution of YLD rates for selected disease groupings by age further illustrate major differences that existed between races. These were particularly prominent for neuropsychiatric conditions in young adults between the ages 15 and 44 years (Figure 20).

## Discussion

Quantifying the burden of disease is not a morally neutral exercise. All summary measures of health include several value choices. A strength of the GBD was to make value choices incorporated in the calculation of DALYs transparent. These include a standard duration of life at each age, an age weighting function, and discounting for time preference. GBD values for these parameters were not changed to ensure the international comparability of the USBODI.

The validation of GBD disability weights in different national contexts is particularly important to enhance the confidence of decision-makers in key findings of national disease burden estimates. The instrument used to derive the disability weights is called the *Person Trade-Off *(PTO). In the GBD the full PTO was executed for a set of 22 indicator conditions.

In order to assess whether groups of people from the United States might value these indicator conditions substantially differently than the benchmark values developed for the GBD, 35 volunteers that included staff from CDC, state health departments, other US federal agencies such as the National Institutes of Mental Health, as well as members of non-profit groups such as the American Heart Association and the Arthritis Foundation, were recruited to execute a PTO (PTO1 and PTO2) exercise as part of the US study. These participants were placed in 4 small groups of 8–12 members according to the GBD PTO (PTO1 and PTO2) protocol. Consistent with results from other such groups coordinated in a variety of international settings by the Burden of Disease Unit at Harvard, there was evidence of substantial inter-*individual *variation between the participants for conditions associated with milder disability (Table 4). For example, the median value for vitiligo of the face was 0.00, and the average disability weight was 0.04. The standard deviation for the disability weight associated with this condition was larger than the actual point estimate. The coefficients of variation (C.V. = standard deviation/point estimate) were much smaller for conditions associated with more severe disability such as quadriplegia and severe dementia. However, despite variation between individuals within these groups, the correlation between groups for the disability weight values was very strong. Nineteen conditions were included in every exercise. For each group the median disability weight value for each of these conditions was calculated. The correlations between pairs of groups for the 19 disability weight values were very high (Table 5, Range of Pearson's correlation coefficients = 0.82–0.99).

**Table 4:** Disability weights from person trade-off exercise conducted in Atlanta compared to composite scores from other exercises conducted at various international sites

Indicator Condition	Atlanta PTO (N = 35)				Composite scores (N = 192)			
	Median	Mean	S.D.*	C.V.^+^	Median	Mean	S.D.*	C.V.^+^
	
Vitiligo on Face	0	0.04	0.11	2.75	0	0.04	0.1	2.5
Watery Diarrhea	0.02	0.06	0.08	1.33	0.05	0.1	0.16	1.60
Fracture of Radius	0.06	0.1	0.11	1.10	0.09	0.13	0.16	1.23
Infertility	0.03	0.11	0.16	1.45	0.09	0.16	0.19	1.19
Erectile Dysfunction	0.09	0.19	0.22	1.16	0.17	0.22	0.23	1.05
Severe Sore Throat	0.13	0.19	0.18	0.95	0.13	0.23	0.26	1.13
Rheumatoid Arthritis	0.17	0.26	0.24	0.92	0.29	0.32	0.22	0.69
Below Knee Amputation	0.29	0.32	0.20	0.63	0.29	0.34	0.22	0.65
Deafness	0.44	0.43	0.28	0.65	0.36	0.4	0.24	0.60
Recto-vaginal Fistula	0.29	0.38	0.33	0.87	0.41	0.44	0.28	0.64
Angina	0.38	0.39	0.23	0.59	0.43	0.46	0.26	0.57
Mental Retardation	0.64	0.55	0.28	0.51	0.5	0.5	0.25	0.50
Blindness	0.5	0.53	0.25	0.47	0.63	0.58	0.21	0.36
Paraplegia	0.67	0.6	0.25	0.42	0.71	0.68	0.2	0.29
Major Depression	0.89	0.79	0.23	0.29	0.81	0.75	0.2	0.27
Severe Migraine	0.96	0.89	0.18	0.20	0.88	0.8	0.2	0.25
Dementia	0.9	0.85	0.16	0.19	0.9	0.86	0.13	0.15
Active Psychosis	0.95	0.9	0.12	0.13	0.91	0.87	0.14	0.16
Quadriplegia	0.93	0.9	0.09	0.10	0.91	0.87	0.14	0.16

* S.D. = standard deviation.

^+ ^C.V. = Coefficient of Variation (standard deviation/mean)

**Table 5:** Pearson's correlation coefficients for median disability weights for each exercise based on 19 conditions common to all person trade-off exercises

Group									
International I	International I								
Netherlands	0.96	Netherlands							
Maghreb-8	0.94	0.95	Maghreb						
Japan	0.90	0.82	0.85	Japan					
GBD	0.97	0.95	0.97	0.88	GBD				
International II	0.99	0.97	0.94	0.89	0.97	International II			
CDC	0.97	0.98	0.92	0.84	0.95	0.98	CDC		
Brazil	0.90	0.91	0.87	0.83	0.87	0.90	0.90	Brazil	
Mexico	0.95	0.93	0.92	0.90	0.93	0.96	0.96	0.95	Mexico
Composite	0.99	0.98	0.96	0.89	0.98	0.99	0.99	0.94	0.97

Based on the above results it seemed reasonable to use the set of disability weights from the GBD study for the US evaluation. In a few instances more detailed data on health conditions were available in the US on the distribution of severity for certain health conditions such as depression [see Additional file 2]. Disability weights for severity-specific stages were developed for many of these conditions as part of a burden of disease and injury study implemented in the Netherlands [20]. Therefore, the Dutch weights were used when stage specific information on severity was available.

In the mid-1990s chronic diseases such as cardiovascular diseases, cancers, depression, osteoarthritis, diabetes mellitus, and alcohol use and abuse were the leading causes of death and disability in the United States. In addition, injuries from motor-vehicle accidents and the HIV epidemic exacted a substantial toll on the US population. These findings are consistent with other assessments of disease burden in developed and developing countries. However, the use of DALYs to enumerate the impact of health conditions is notably different from a simple listing of causes of death. This metric captures the importance of mental conditions, such as depression and degenerative musculoskeletal disease that cause major health problems but result in few deaths, as well as the importance of premature deaths among young adults (Tables 27, and 28).

**Table 27:** Top twenty leading causes of Disability Adjusted Life Years (DALY), Years Lost to Disability (YLD), Years of Life Lost (YLL) and Deaths for Males – US 1996*

	**Cause**	**DALY (%)**	**YLD (%)**	**YLL (%)**	**Death (%)**
1	Ischaemic heart disease	1,958 (11.0)	152 (2.1)	1,806(17.2)	287 (24.7)
2	Road traffic accidents	934 (5.2)	233 (3.2)	701 (6.7)	29 (2.5)
3	Lung trachea or bronchial cancer	813 (4.6)	35 (0.5)	778 (7.4)	102 (8.8)
4	HIV/AIDS	764 (4.3)	189 (2.6)	575 (5.5)	25 (2.2)
5	Alcohol use	732 (4.1)	651 (8.9)	81 (0.8)	5 (0.4)
6	Cerebrovascular Disease	674 (3.8)	317 (4.3)	357 (3.4)	63 (5.4)
7	COPD	642 (3.6)	373 (5.1)	269 (2.6)	52 (4.5)
8	Homicide and Violence	568 (3.2)	82 (1.1)	486 (4.6)	17 (1.5)
9	Self-inflicted injuries	541 (3.0)	8 (0.1)	534 (5.1)	26 (2.2)
10	Unipolar major depression	470 (2.6)	470 (6.4)	0 (0.0)	0 (0)
11	Diabetes mellitus	442 (2.5)	222 (3.0)	220 (2.1)	28 (2.4)
12	Osteoarthritis	435 (2.4)	434 (5.9)	1 (0.0)	0 (0)
13	Drug use	412 (2.3)	384 (5.2)	27 (0.3)	1 (0.1)
14	Congenital Abnormalities	410 (2.3)	238 (3.2)	172 (1.6)	6 (0.5)
15	Dementia and other degenerative and hereditary CNS disorders	382 (2.1)	332 (4.5)	50 (0.5)	14 (1.2)
16	Asthma	303 (1.7)	273 (3.7)	30 (0.3)	2 (0.2)
17	Cirrhosis of the liver	281 (1.6)	61 (0.8)	220 (2.1)	17 (1.4)
18	Conditions arising during the perinatal period	274 (1.5)	14 (0.2)	260 (2.5)	8 (0.7)
19	Cancer colon or rectum	249 (1.4)	37 (0.5)	213 (2.0)	30 (2.6)
20	Prostate cancer	239 (1.3)	79 (1.1)	160 (1.5)	37 (3.2)
-	Total number for each measure in the top 20 causes	11,523 (64.5)	4,584 (62.5)	6,940 (65.9)	749 (64.5)
-	Total number for each measure	17,861(100)	7,331(100)	10,530 (100)	1,164 (100)

*All counts for DALYs, Deaths, YLDs, and YLLs are in 1000's.

**Table 28:** Top twenty leading causes of Disability Adjusted Life Years (DALY), Years Lost to Disability (YLD), Years of Life Lost (YLL) and Deaths for Females – US 1996*

	Cause	DALY (%)	YLD (%)	YLL (%)	Death (%)
1	Ischaemic heart disease	1,177 (7.7)	124 (1.6)	1,052(14.0)	249 (21.7)
2	Unipolar major depression	900 (5.9)	900 (11.7)	0 (0.0)	0 (0.0)
3	Cerebrovascular disease	836 (5.5)	408 (5.3)	428 (5.7)	99 (8.6)
4	COPD	612 (4.0)	354 (4.6)	257 (3.4)	48 (4.1)
5	Lung trachea or bronchial cancer	550 (3.6)	26 (0.3)	523 (6.9)	66 (5.7)
6	Breast cancer	515 (3.4)	64 (0.8)	450 (6.0)	47 (4.1)
7	Osteoarthritis	508 (3.3)	506 (6.6)	1 (0.0)	1(0.0)
8	Dementia and other degenerative and hereditary CNS disorders	507 (3.3)	424 (5.5)	83 (1.1)	29 (2.5)
9	Diabetes mellitus	504 (3.3)	274 (3.6)	230 (3.1)	34 (3.0)
10	Road traffic accidents	459 (3.0)	134 (1.7)	326 (4.3)	15 (1.3)
11	Alcohol use	409 (2.7)	386 (5.0)	23 (0.3)	1 (0.1)
12	Asthma	362 (2.4)	320 (4.2)	42 (0.6)	4 (0.3)
13	Congenital abnormalities	352 (2.3)	205 (2.7)	147 (1.9)	6 (0.5)
14	Cancer colon or rectum	234 (1.5)	38 (0.5)	197 (2.6)	31 (2.7)
15	Conditions arising during the perinatal period	220 (1.4)	16 (0.2)	205 (2.7)	6 (0.5)
16	Lower respiratory infections	195 (1.3)	10 (0.1)	186 (2.5)	46 (4.0)
17	PTSD	194 (1.3)	194 (2.5)	0 (0.0)	0 (0.0)
18	HIV/AIDS	193 (1.3)	49 (0.6)	144 (1.9)	6 (0.5)
19	Panic disorder	182 (1.2)	182 (2.4)	0 (0.0)	0 (0.0)
20	Bipolar disorder	165 (1.1)	165 (2.1)	0 (0.0)	0 (0.0)
Total number for each measure in the top 20 causes		9,074 (59.6)	4,779 (62.1)	4,294 (57.0)	688 (59.7)
Total number for each measure		15,230 (100)	7,693 (100)	7,537 (100)	1,151 (100)

*All counts for DALYs, Deaths, YLDs, and YLLs are in 1000's

The juxtaposition of the twenty leading causes of death, YLL, YLD and DALYs illustrates the extent to which an assessment of the relative importance of various causes based simply on total number of deaths differs from the assessment of leading causes of YLL, YLD and DALYs. For example, the total number of years lived with a disability resulting from unipolar major depression (1.3 million YLD) was equal to the number of years lost due to premature death from lung cancer (1.3 million YLL); and the burden resulting from osteoarthritis and motor vehicle accidents were similar (940.6 million YLD and 1 billion YLL respectively).

Osteoarthritis of the hip and knee (OA) and rheumatoid arthritis (RA) were the two leading musculoskeletal disorders. OA is an important public health problem that affects mostly older adults causing great pain and disability, and is one of the most rapidly growing causes of disability. The estimated 40 million prevalent cases in 1996 is projected to increase to 60 million cases by 2020 [22].

Substantial differences were found in the relative impact of individual conditions by gender and race. HIV/AIDS, alcohol dependence, as well as violent and unintentional injuries accounted for most of the worse health outcomes observed among Black and American Indian populations compared to White and Asian populations. Blacks fared much worse than the other race groups with regard to pregnancy outcomes. Blacks were the only group for which perinatal conditions ranked among the top ten causes of DALYs. Relatively high perinatal mortality rates persist in this population due to the combined effect of premature delivery and poor perinatal care.

Conditions associated with social issues in younger ages were much more common among Blacks and American Indians. For instance, YLL rates for HIV/AIDS were fivefold larger for Blacks than they were for any of the other races. YLL rates for homicide and violence were seven times higher for American Indians and twice as high for Blacks than they were for Whites and Asians.

One important objective of the study was to place the United States public health situation in a global context. Non-communicable diseases are the leading causes of deaths in all industrialized countries, where child and adult mortality are low. In developing regions, where child and adult mortality are still high, Group I represents a much larger share of the total. The dominance of HIV/AIDS observed in Blacks in the United States was akin to that in developing regions of the world. It was the third leading cause among black women in the US and the fifth leading cause among females in developing regions. HIV/AIDS accounted for a much smaller proportion of DALYs in other races in the US and did not figure among the top ten causes of DALYs in developed countries. Alcohol use for males in most racial subgroups in the United States exacted a high burden. This condition also ranked among the top five in other developed regions. Only Asian males and people living in developing countries did not have a large number of DALYs attributed to alcohol use.

US rankings clearly fell outside of the range observed elsewhere for a few causes: homicide and violence, HIV/AIDS, and perinatal conditions stand out regardless of race and gender. The United States has not been as successful in reducing the mortality burden due to violent injuries and perinatal conditions as were other industrialized countries with comparable levels of development.

In spite of the extensive population-based data available in the United States, there were limitations particularly in estimating disease burden by race due to smaller populations – Asians and American Indians. The major methodological limitations pertained to the different methods used to assign race and ethnicity in the census compared to death certificates; and to the limited population-based information that was available for many conditions for Asians and American Indians. For these last two groups, ratios of YLL to YLD for the overall US population were generally used to estimate the burden due to non-fatal health conditions. Such assumptions introduce a certain level of uncertainty in the estimates and call for caution in the interpretation of small absolute differences in the number of DALYs between different causes. This study provides a benchmark against which to assess future trends in health differentials in the United States and underscores the importance of further research to improve methods, provide stronger empirical evidence and better understanding of major risk factors for poor health outcomes.

## Conclusion

This study provides a comprehensive picture of conditions that contribute most to poor health outcomes, and yields new evidence to the discussion of racial health inequalities in the United States. The existence of health inequalities is widely acknowledged and lies at the core of public health policy: reducing health inequalities is the major focus of Healthy People 2010 [23]. Previous studies have documented differentials in mortality by cause and have examined socio-economic determinants – income and education – of population health outcomes and health outcomes mediated through the health system: the utilization of health services, access, and quality of care [24-26].

The main policy message emerging from this study is that cost-effective public health interventions are available to reduce the burden of the three conditions that contributed most to racial inequalities. It adds new evidence that greater investments of public health interventions have a much greater potential to reduce large health inequalities in the United States than do technology driven curative interventions. The fact that other countries, which have lower expenditures per capita have achieved better health outcomes than the United States indicates that the major goal of reducing health disparities by 2010 can be achieved.

## Competing interests

The author(s) declare that they have no competing interests.

## Authors' contributions

CJL conceived of the study, participated in its design, and helped to draft the manuscript. CMM, MTM participated in the study's design and coordination, data analysis and helped to draft the manuscript. SB, NT, MM, MTB, EME, JS, JGK, MH contributed to data analysis. ME helped to draft the manuscript. All authors read and approved the final manuscript.

## Supplementary Material

Additional File 1 Global burden of disease methodology: summary overview. A summary overview of GBD methods. Click here for file

Additional File 2 Data sources and methods for developing estimates for the incidence, mortality, prevalence, and duration of selected conditions for estimation of YLD in the United States. A detailed presentation of analytic methods, data sources, and data sets used to develop estimates for major causes of diseases and injuries. Click here for file

Additional File 3 US burden of disease study classification system for diseases and injuries. The detailed list of causes selected for the USBODI. Click here for file

Additional File 4 US burden of disease – Detailed tabulations of deaths, YLL, YLD and DALYs. Detailed tabulations of deaths, YLL, YLD and DALYs for the 73 causes included in the USBODI by age, gender and race. Click here for file

Additional File 5 Epidemiological estimates. Detailed incidence by age, sex and race by disease and sequelae, included in the USBODI. Click here for file
