# Supplementary material for: The burden of disease and injury in the United States 1996
Source: Popul Health Metr. 2006 Oct 18;4:11. doi: 10.1186/1478-7954-4-11 (PMC1635736; doi:10.1186/1478-7954-4-11)
Supplement: Additional File 1 — Global burden of disease methodology: summary overview. A summary overview of GBD methods. [file 1478-7954-4-11-S1.doc]

##### *A. Summary Measures of Population Health*

The US Burden of Disease and Injury study (USBODI) was patterned after the Global Burden of Disease study (GBD). The metric used in this analysis to assess the burden associated with specific diseases and injuries is called the Disability Adjusted Life Year (DALY). The DALY is one member of a family of approaches to quantifying influences on population health known as *Summary Health Measures.* Summary measures of health incorporate the impact of mortality and morbidity into a single metric. The history, conceptual framework, methodological composition, technical complexities and ethical implications of these measures are extensive and have been reviewed in detail by other authors ([1] Field, 1998; [2] Murray, 1996; [3] Murray, 2000; [4] Preston, 1991; [5] Molla, 2001). The quantification of BODI includes estimation of years of life lost to premature death (YLL), computed based on actual number of deaths, and years of life lost to disability from non-fatal conditions (YLD), calculated based on a set of disease specific epidemiological parameters developed for each condition.

A.1. Disability-Adjusted Life Years – Conceptual Framework

The computation of summary measures like the DALY requires that life years lived in a less than prefect health be weighed with values ranging between 0 reflecting an absence of disability, and 1 which is equivalent to death ([6] Gold, 1996; [7] Nord, 1999; [8] Sullivan, 1971). The GBD developed disability weights to quantify the severity of disability due to different diseases and injuries, which range between 0 and 1. The validation of GBD disability weights in different national contexts is particularly important to enhance the confidence of decision makers in key findings of national disease burden estimates.

The instrument used to derive the disability weights is called the *Person Trade-Off* (PTO) exercise. In the *GBD* the full PTO was executed for a set of 22 indicator conditions. The participants were a group of experts from around the world who met in Geneva at WHO headquarters. These participants were asked to develop these disability weights for the purposes of international comparisons. After the disability weights were developed a modified Delphi approach was used by this group to provide over 400 other health states using these indicator conditions as benchmarks [9] (Murray, 1996).

All summary measures of health include several value choices. Strength of the GBD was to make value choices incorporated in the calculation of DALYs transparent. These include a standard duration of life at each age, an age weighting function, and discounting for time preference. GBD values for these parameters were not changed to ensure the international comparability of the USBODI.

***B. Estimation of Years of Life Lost (YLL)***

The number of years of life lost following premature death (YLL) represents the mortality component of DALYs.

B.1. Classification of Causes of Deaths

Mortality data have traditionally been the major data source for health policy. Therefore, this information provides a benchmark against which health data analyses using other methods, such as the DALY, can be compared.

All deaths occurring in the United Stated each year are reported to the National Center for Health Statistics (NCHS). Causes of deaths are coded following an internationally agreed set of rules – the International Classification of Causes of Death (ICD). The first ICD was established in 1900, and has since been reviewed periodically. In 1996 the ninth revision of the ICD (1976) was in use (ICD-9). It includes several thousands of possible codes to describe the underlying cause of death. This large number of coding options provides a very rich source of information on individual causes of death, but does not lend itself to inform public health policy.

The GBD classification scheme was developed to overcome this limitation, as a tool to better inform the health policy debate. It follows a ‘tree-structure’. At the first level of disaggregation, overall mortality is divided into three broad groups of causes, which are both exhaustive and mutually exclusive:

1. Group I – Communicable, maternal, perinatal and nutritional conditions
2. Group II – Noncommunicable diseases
3. Group III – Injuries

Each group has been further divided into several major sub-categories of disease and injury that are mutually exclusive and exhaustive At the second level of disaggregation, Group I has been divided into infectious and parasitic causes, respiratory infections, maternal conditions, conditions arising during the perinatal period and nutritional deficiencies; Group II has been divided into 14 categories which include cardio-vascular diseases, cancers, diabetes, and neuro-psychiatric disorders; and Group III has been divided into two major sub-categories – intentional and non-intentional injuries. A third level of disaggregation is used to identify more specific causes of deaths within each of the second level categories. The specific GBD cause list includes 107 specific causes and clusters of causes. The overall GBD classification scheme was applied for the analysis of causes of deaths in the United States.

B.2. Redistribution of Ill-defined Causes and Cardiovascular “Garbage Codes”

The ICD-9 includes several codes that capture ill defined conditions not classified elsewhere. Several algorithms developed to redistribute these ill-defined conditions in the GBD were applied to the US.

(1) Chapter XVI of the ICD‑9 (Symptoms, Signs and 111‑Defined Conditions).

The redistribution algorithm for Chapter XVI codes was age‑specific:

(1) All deaths under age five were proportionately redistributed to all Group I causes and all deaths age five and above were proportionately redistributed to all Group 11 causes.

(2) Injuries undetermined whether accidentally or purposefully inflicted (E980‑989); Other and unspecified environmental and accidental causes which includes E 929.9; and Unspecified accidents stated as accidentally inflicted but not otherwise specified, were redistributed proportionately for each age group to intentional and unintentional injuries.

(3) Ill-defined cancers. These were proportionately redistributed to cancers for each age group.

(4) Selected cardiovascular codes. The algorithm to redistribute selected cardiovascular codes was further developed for the United States.

***C. Estimation of YLD***

C.1. Developing Internally Consistent Epidemiological Estimates for Non-fatal Conditions

Estimating years lived with a disability (YLD) is the most difficult component of the BODI study. While cause of death information is reported for each death and entered into a single database by the National Center for Health Statistics (NCHS), the estimation of YLD is much more complex. It requires a wide range of different epidemiological parameters specific to each disease or condition included in the study. These are not compiled in a single national database. A good knowledge of the diseases and judgment are thus essential to make sound decisions on what the most plausible sources of information are and which parameters best describe the disability caused by each disease. Art and science are needed to reconcile inconsistencies, deal with uncertainty, and fill gaps in the available data. These exist even in the United States, where epidemiological parameters are available for most conditions.

A disease process can be described by a number of variables, like incidence, prevalence, remission, case-fatality, duration, and mortality. These variables differ in nature: for example incidence, remission, and case-fatality express transitions from one state to another, while prevalence is a proportion. All of these variables can, in principle, be observed, but with a different degree of difficulty. Observing prevalence and incidence is usually much harder than mortality. Data collection, when done, is often limited in time and geographical area. Problems of case definition abound. Data are frequently incomplete, and when present, their validity may be in doubt. For instance, when incident cases are greatly underreported, the observed incidence will be too small to be plausible in relation to the reported mortality. Given the different nature of the disease variables and the differences in the way the data are collected it is inevitable that the observations are internally inconsistent.

The number of YLD resulting from a given condition is the product of the number of new cases (incident cases) of a given disability multiplied by its duration, weighted by the severity of the disability (or disability weight) The first step in the computation of YLD is the development of valid epidemiological parameters, based on sound understanding of the epidemiology of the disease. Epidemiological parameters required to estimate YLD are disability incidence, disability duration, age of onset, and distribution by severity class, by race and sex. These in turn require estimates of incidence, remission, case-fatality rates or relative risks, by age and sex. For many diseases or conditions, information on incidence and duration is not available. Most commonly, the prevalence of a disease or of its sequelae is known.

A disease model that explicitly describes the causal pathways allows inferring missing data if existing data is sufficient. For example, incidence can be calculated using such a disease model, if prevalence, case-fatality and remission rates are known. For those conditions with insufficient data on the above mentioned epidemiological parameters, the software program DISMOD is helpful in finding consistent matches between estimates of prevalence, incidence, duration, remission and mortality.

Interview surveys such as the National Health Interview Survey provide self-reported information on disabilities in key domains such as self-care, occupation, or recreation. While good information on the prevalence of disability by age in the community would be very helpful, survey data are usually uninformative for estimating YLD. This is because of the difficulty in attributing impairment to the underlying causes, and because of the differences between the disease concept the general public and the medically defined disease category for which information is intended to be collected.

Surveys using standard methods and definitions of visual, or hearing impairment, cognitive function, motor function, and psychosis and anxiety can be very useful for estimating the total burden of each condition or sequalae of other diseases. Surveys of blood tests that may be helpful include: hemoglobin (for anemia), serology for HIV, Hepatitis B, RPR (for syphilis), glucose tolerance test (for diabetes). For a variety of other diseases, disease specific measurements may be helpful in estimating burden such as surveys of goitre, epilepsy screening questions, alcohol abuse questions, *etc.* laboratory tests used in surveys that provide substantial information for disease burden studies include tuberculin skin tests (for TB), sputum examinations (for TB), chest X-rays (for TB), pulmonary function tests (for COPD), and EKGs (for ischemic heart disease). Data from obtained during clinical examination may be used to estimate burden for some diseases such amputations.

Estimating the burden of certain conditions requires combining information on the epidemiology of the disease with current intervention coverage in order to determine how much of the current burden is alleviated through treatment, either by reduction of the severity of disease or a shortening of the disability (for example, both may be the case in the treatment of tuberculosis). Cohort Studies can often provide insights into the age-pattern of incidence, remission and case-fatality that are useful in estimating the burden of a disease. Such studies are rare. In the majority of cases, routine data on consultations by diagnosis or even reported morbidity will not be very helpful in estimating burden. Facility based data (unless the coverage of the health system is near complete) will always be based on biased samples of the disability present in the community. Examples of conditions that can be estimated from hospital data if there is good coverage and data are available, include: perinatal and maternal conditions, meningitis, stroke, myocardial infarction, surgical conditions and the more serious injuries. There is an important distinction between surveillance needs and burden estimation. For surveillance and health service management purposes, routine reporting of consultations and admissions by diagnosis may be more helpful than it is for estimating burden.

C.2. DISMOD: A tool for Internally Consistent Epidemiological Estimates

Once all available data on a disease have been collated, the first step is to assess whether the observations are all internally consistent. For the GBD, the computer software named DisMod was developed for the purpose of supplementing observations with expert knowledge and producing internally consistent estimates of disease epidemiology. The updated version , DisMod II, has a number of additional features to enhance usability. DisMod II has been developed to provide a full graphical interface, database storage capabilities and substantially enhanced features and options. Version 1.0 of DISMOD II is now publicly available for downloading from the WHO website.

DISMOD allows the user to check if a set of assumptions on incidence, remission and case-fatality rates are consistent with prevalence and mortality numbers. Usually, not all available data will be consistent with each other. Judgment is then required to choose which data source is more likely to represent the reality in a particular community. When *ad hoc* adjustments must be made, the logic underlying these adjustments should be presented, justified, and subjected to consultation with experts. Estimates of disability incidence, duration, age of onset, and severity class must be plausible. Plausible estimates are estimates that are reasonably consistent with the known epidemiology of the disease and consistent with independent observations of the same phenomenon. For example, estimates of infertility due to septic abortions, obstructed labor or sexually transmitted diseases should not exceed the total observed secondary infertility rate.

DisMod is based on a simple disease model, which describes the disease by an illness-death process with only one diseased state. There are four states: susceptibles (or non-diseased); cases (or diseased); cause specific deaths; and deaths from other causes. Susceptibles become cases at an instantaneous rate *i*; cases go back to being susceptible at remission rate *r*; both susceptibles and cases are subject to a general instantaneous mortality rate ***m***; and the cases are subject to the instantaneous case-fatality rate ***f***. The set of differential equations implied in the Figure can be solved explicitly or computationally. DisMod takes a user-defined set of incidence, remission and case-fatality rates combined with either user-defined or regional general mortality rates to follow a cohort over time. The inputs to DisMod I are instantaneous rates and the outputs are prevalence rates, population incidence rates, durations, ages of onset and cause-specific mortality rates

DisMod implements an exact solution to the underlying differential equations as well as calculating solutions when the 3 hazard rates are provided as inputs. DisMod allows other combinations of inputs such as prevalence, remission and case-fatality. In these cases, DisMod uses the down hill simplex method to fit hazards such that the model reproduces the available input variables. DisMod has a range of features including sensitivity analysis, uncertainty analysis, the ability to give different weights to the various inputs, and the ability to smooth inputs and specify age patterns for outputs. New epidemiological estimates, based on US specific data, were developed for most conditions. A detailed account of data sources and methods applied to develop epidemiological estimates for major causes of disease burden are provided in Annex 2.

**references**

[1] Field, MJ and Gold MR, Summarizing Population Health: Directions for the development and application of population metrics. Washington, D.C.: National Academy Press; 1998.

[6] Gold M, Patrick D, Torrance G, Fryback D, Hadorn D, Kamlet M et al. Identifying and valuing outcomes. In: Gold MR, Siegel J, Russell L, Weinstein M, editors. Cost-effectiveness in health and medicine. New York: Oxford Universtiy Press, 1996: 82-123.

[5] Molla M, Wagener D, Madans J. Summary measures of population health: Methods for calculating healthy life expectancy. Healthy People 2000 Statistical Notes 2001;(21):1-11.

[9] Murray CJL, Lopez A. The Global Burden of Disease: a comprehensive assessment of mortality and disability from diseases, injuries, and risk factors in 1990 and projected to 2020. 1 ed. Cambridge: Harvard University Press; 1996.

[3] Murray C, Salomon J, Mathers C. A critical examination of summary measures of population health. Bull WHO 2000; 78(8):981-994.

[2] Murray CJL, Lopez A. Evidence-Based Health Policy - Lessons From the Global Burden of Disease Study. Science 1996;274:740-3.

[7] Nord E. Cost-value analysis in health care. United Kingdom: Cambridge University Press, 1999.

[4] Preston S. Health indices as a guide to health sector planning: A demographic critique. In: Gribble J, Preston S, editors. The epidemiological transition: Policy planning implications for developing countries. Washington, D.C.: National Academy Press, 1991: 126-144.

[8] Sullivan DF. Disability components for an index of health. HSMHA 2. 1971.
