# Supplementary material for: The burden of disease and injury in the United States 1996
Source: Popul Health Metr. 2006 Oct 18;4:11. doi: 10.1186/1478-7954-4-11 (PMC1635736; doi:10.1186/1478-7954-4-11)
Supplement: Additional File 2 — Data sources and methods for developing estimates for the incidence, mortality, prevalence, and duration of selected conditions for estimation of YLD in the United States. A detailed presentation of analytic methods, data sources, and data sets used to develop estimates for major causes of diseases and injuries. [file 1478-7954-4-11-S2.doc]

1. AIDS and non-AIDS associated HIV infection

2. Selected invasive cancers

1. Diabetes mellitus and selected sequelae

4. Ischemic heart disease

1. Stroke
2. Non-fatal injuries
3. Congenital anomalies
4. Unipolar major depression and substance abuse

**1**. **AIDS and Non-AIDS Associated HIV Infection**

AIDS

###### *Data Sources*

Data on the incidence, prevalence and mortality associated with AIDS and AIDS related conditions were obtained from the HIV and AIDS surveillance systems maintained at the Centers for Disease Control and Prevention (CDC). Estimates are based on data reported to this data system through June 1999. The 1993 case definition was used to identify AIDS cases [1]. This definition classifies HIV-infected persons with CD4 T-lymphocyte counts <200/µL or CD4 percentage of lymphocytes <14% as AIDS cases even if they have not experienced an AIDS-defining opportunistic infection. The case counts were inflated by a factor of 10% to adjust for underreporting of AIDS cases [2]. In addition to adjusting for underreporting, the counts were adjusted for the reporting delays. This adjustment uses a maximum likelihood procedure that provides extra weight to recently reported cases and deaths as an estimate of the numbers that would eventually be reported. This procedure assumes that the reporting delay distribution does not vary over the 6 prior to most recent year for which reports are available [3].

Active surveillance of deaths among AIDS cases and death certificate data from the National Center for Health Statistics were used to generate the number of AIDS deaths. The counts from these data sources do not completely coincide because some AIDS cases die of other causes (e.g. injury), and some persons designated as dying of HIV may not have technically met the AIDS case definition prior to their death. Therefore, they would not have been reported to that surveillance system. However, if they truly died of HIV infection, it is highly likely that their CD4 T-lymphocyte count, if known, would have been low enough to meet the AIDS case definition. Therefore, for estimation of deaths due to AIDS, it was assumed that all death certificates with HIV infection listed as the underlying cause pertained to persons who died of AIDS. Also, death certificates with any mention of HIV infection were included if the certificate also identified an AIDS-defining illness, an illness likely to be due to underlying HIV infection (cardiomyopathy, aspergillosis, or nocardiasis) or cardiac arrest [4].

*Estimation Procedures*

The mortality, and to a lesser extent incidence, of AIDS began to decline rapidly in 1996. There is good evidence that the initial decline that began in 1995 and continued in 1996 was primarily the result of treatment of patients who had advanced immune suppression with multiple drug therapy that includes a Protease Inhibitor [5]. These regimens are known as highly active anti-retroviral therapies (HAART). Therefore, since the software used in the BODI methods assumes a steady state relationship between incidence, prevalence and case-fatality it is not possible to derive a plausible set of "DISMOD-consistent" estimates using available data on all these measures for AIDS from 1996. Hence the perspective adopted was to try and calculate an *expected* average duration of disease based on the information available in 1996 that was least influenced by the rapidly changing clinical situation that was emerging during 1996. Since HAART therapy at that time was primarily directed to very ill patients with AIDS, it was assumed that the death rates would decline faster than the incidence rate [6]. Therefore, age specific AIDS incidence and prevalence were entered in DISMOD to estimate average durations of AIDS while ignoring the resulting mortality figures. It was assumed that no patient experienced a remission from AIDS. In general the mortality rates in the resulting DISMOD models were higher than the observed values since case fatality rates were precipitously dropping during 1996. Therefore, the measured mortality rates have been entered in the epidemiologic estimation tables along with the "synthetic" average durations derived from the measured incidence and prevalence values. These probably represent reasonable estimates that would have been expected by most practitioners at that time when the impact of treatment was not completely appreciated. The "true" expected, average, duration will not be fully understood until more data are garnered from patients who have been under treatment since 1996.

#### Non-AIDS Related HIV-Infection

*Data Sources and Estimation Procedures*

Data on the prevalence of non-AIDS HIV-infections were obtained from CDC. They are based on a number of sentinel survey systems maintained by the CDC. The incidence values for AIDS were then entered in DISMOD as mortality figures since the onset of AIDS was the endpoint of interest in this model. Age specific average durations from infection until the onset of AIDS were obtained from two sources. The durations entered into DISMOD for persons ages 5-65 years were based on data published in the Collaborative Group on AIDS Incubation and HIV Survival Study [7]. Estimates of duration from infection to AIDS for children 0-5 years of age were derived from a published analysis of data from the Pediatric Spectrum of Disease Project [8]. Both of these analyses provided median time intervals for the durations of interest. Since multiple studies have suggested that the incubation periods for HIV infection follow a Weibull rather than a simple exponential distribution, the median values were used as the starting estimates of the average durations in these models [9]. However, since both of the studies that provided the incubation periods did not include the CD4 criteria in the AIDS case definitions, the assumption proffered by Holtgrave et al.was adopted and one year was subtracted from each of the age-specific duration values [10].

In order to obtain incidence numbers commensurate with the observed AIDS case rates and estimated prevalence numbers adjustments were made to the final durations (see table). None of these adjustments involved values greater than one year for any age group. The final number of total incident cases estimated from DISMOD was between 55,000 and 60,000 which approximates Rosenberg's estimate for the number of cases in the early 1990. The prevalence figures from this model closely approximated the numbers provided by CDC. All of these estimates seemed well within the 20% coefficient of variation observed in the published results of the back-calculation studies used to anchor these DISMOD models [11].

| **Original and final duration values from the literature used to model HIV and AIDS estimates.** | | | | | |
| --- | --- | --- | --- | --- | --- |
| Age Groups in Published Materials | Median time to Clinical AIDS (years) | Median time to AIDS adjusted for T-cell | DISMOD Age Groups | Time to AIDS Entered in DISMOD | Adjustments after DISMOD Modeling with rounding |
| 0-4 | 5.0 | 4.0 | 0-4 | 4.0 | 4.0 |
| 5-14 | 11.0 | 10.0 | 5-14 | 10.0 | 10.0 |
| 15-24 | 11.0 | 10.0 | 15-24 | 10.0 | 10.0 |
| 25-34 | 9.8 | 8.8 | 25-44 | 8.2 | 9.0 |
| 35-44 | 8.6 | 7.6 | 45-64 | 5.5 | 6.0 |
| 45-54 | 7.7 | 6.7 | 65-74 | 4.8 | 5.0 |
| 55-64 | 5.3 | 4.3 | >75 | 3.4 | 4.0 |
| 65-74 | 4.0 | 3.0 |  |  |  |
| > 75 | 3.0 | 2.0 |  |  |  |

**2. Selected Invasive Cancers**

Cancers Other Than Non-Melanoma Skin Cancer

*Data Sources*

The data sources for these estimates were the same as those used by most organizations that attempt to develop estimates for the descriptive epidemiology of cancer in the United States [12]. The estimated numbers of cases as well as five year survival proportions were calculated using cancer incidence rates from the regions of the United States included in the National Cancer Institute’s (NCI) Surveillance, Epidemiology, and End Results (SEER) program and population data collected by the US Bureau of the Census [13]. Mortality counts and rates were obtained from the Underlying Cause of Death data compiled by the National Center for Health Statistics [14].

*Estimation Procedures*

The software program SEER*Stat 2.0 that is provided with data from the NCI was used to generate the incidence and survival estimates used in this analysis [15].^4^ All estimates were obtained using the codes for invasive, malignant tumors from the 9 regions that have participated in the SEER program from 1973-1996. The anatomical coding system of the SEER*Stat 2.0 program was also used since it corresponds to the ICD-9 specifications used in the Global Burden of Disease (GBD) study [16]. Incidence rates were calculated using cancers diagnosed during the period 1992-1996.

The SEER data are sufficiently voluminous to provide cancer incidence rates for whites, blacks and the total population. However, there are insufficient numbers of persons of Asian race in these data to compute rates of satisfactory precision. Therefore, the ratio of the mortality rate in Asians to the mortality rate in the total population was multiplied by the incidence rate in the total population to provide an incidence estimate for Asians.

Survival curves computed using yearly intervals were generated using data from cancers diagnosed during the 10 year period of 1987-1996. Consistent with the methodology in the Global Burden of Disease Study it was assumed that patients surviving five years or longer after a cancer diagnosis were “cured”. Therefore, the average duration of disease entered into the DISMOD software was based on survival curves. In this analysis the average duration was estimated by applying standard life-table methods to annual, observed, survival proportions for the five years after diagnosis.

As noted above there are insufficient numbers of Asians in the SEER data to estimate survival rates or incidence rates. Since the procedure used to calculate incidence rates among Asians generally assumes that the relation between incidence and mortality is equivalent to that observed in the overall population, the duration of disease calculated for the all patients with cancer was used as the value for this variable in this population subgroup.

Mortality counts and rates were derived using the ICD-9 codes from the GBD. This coding system was applied to the underlying causes of death as recorded in the mortality records compiled by NCHS.

*DISMOD*

The above calculations provided estimates of incidence, mortality and average duration that were considered invariate when running the DISMOD software. Remission and case fatality rates were then varied within DISMOD until values for the three invariate estimates were equivalent to the values computed from the above data sources.

Non-Melanoma Skin Cancer

*Data Sources and Estimation Procedures*

Estimates for the 1996 incidence of basal cell and squamous cell carcinomas in the United States were derived using the methods described by Miller *et al* [17]. These incidence rates were summed and submitted as the estimates for non-melanoma skin cancers. Briefly, data from the special National Cancer Institute (NCI) survey of basal cell and squamous cell cancer in eight regions of the United States conducted between June 1, 1977 and May 31, 1978 were used to obtain a reasonably representative set of baseline incidence rates for the United States [18]. The incidence rates from these surveys were then extrapolated to1996 using the average annual rate of increase observed in two long-term continuous population-based non-melanoma skin cancer registries that had been in operation in North America since 1977. These registries were located in the province of British Columbia and the Kaiser-Permanente Health Maintenance Organization in Portland, Oregon. The increases observed in the British Columbia registry were larger than those observed in Portland. Therefore, separate extrapolations using each of these estimated rates of increase from 1978 to 1996 were computed. The average of the two sets of rates for 1996 were used as the incidence estimates for persons of white race for the figures submitted to the Burden of Disease Unit.

Non-melanoma skin cancer is much less common in African-Americans than the rates observed in whites. Estimates for the incidence of this disease in Blacks in the United States were derived from the observation by Halder *et al* that these cancers are 68 times less frequent in African-Americans than in whites [19]. Therefore, we simply divided the incidence estimates for 1996 in whites by 68 to obtain estimates for Blacks. Incidence rates for Asians were assumed to be intermediate between those of whites and Blacks.

*DISMOD*

Since non-melanoma skin cancer is rarely fatal in the United States the assumption that a post diagnosis survival of five or more years represents cure was abandoned. Therefore, estimates of average duration and prevalence derived from DISMOD were computed by assuming that the incidence rates obtained from the above procedures, and mortality rates from the underlying cause of disease data, were invariate. It was also assumed that the remission rate for these cancers was at least 90%. With these assumptions as starting points, remission and mortality rates were varied until consistency was obtained between the incidence and mortality estimates. The resulting average durations were then assumed to represent reasonable estimates for this disease.

**3. Diabetes Mellitus and Selected Sequelae**

Data Sources

The data sources for these estimates were the National Health Interview Survey (NHIS), the National Health and Nutrition Examination Survey III (NHANES) and the NHANES I Epidemiologic Follow-up Survey (NHEFS). Estimates of self-reported diabetes prevalence were supplied by the Division of Diabetes Translation (DDT), CDC based on their analysis of data from the 1994-1996 NHIS as well as new analyses conducted for this study of the 1997 NHIS.

Provisional estimates of diabetes incidence were derived using answers to questions from the NHIS concerning whether the diabetes was diagnosed within 12 months of the date of the interview. Incidence rates for adolescents and children were derived using published estimates from population-based registries of insulin-dependent diabetes mellitus [20].

The self-reported estimates were compared to data from NHANES III to estimate the volume of undiagnosed diabetes. This survey contains questions similar to those in the NHIS on self-reported incidence and prevalence, as well as biochemical assays of fasting plasma glucose, and a 2 hour oral glucose tolerance test. To identify undiagnosed diabetics we used the American Diabetes Association criteria that only use fasting plasma glucose levels since the mortality associated with this approach is similar to that found with the more complex World Health Organization diagnostic standards [21].

Mortality data tapes in 1996 were used to identify the number of deaths attributed to ICD-9 code 250. The numbers and rates provided in the tables are based on these data. The number of deaths attributable (though not coded with diabetes as the underlying cause) to diabetes in DISMOD were calculated using published estimates on the age, sex and specific relative risk for total mortality associated with diabetes in adults [22]. These publications used NHEFS data. An independent analysis of this dataset with proportional hazard modeling was also conducted for this study. The predominant form of diabetes in children and adolescents is Type I, or insulin-dependent diabetes mellitus. Therefore relative-risks in children and adolescents (i.e < 24years of age) were based on published estimates from limited cohort studies which have demonstrated relative risk between 6-8, and the expert opinion of DDT staff [23]. These colleagues asserted that the relative risks increased with decreasing age. Incidence rates for sequelae were derived directly from the Global Burden of Disease (GBD) estimates.

Estimation Procedures

We estimated smoothed, age, race and sex-specific, self-reported prevalences for diabetes using quadratic logistic regression modeling of the NHIS and NHANES data among black and white racial groups. All the surveys indicated that the self-reported prevalence of diabetes decreased after the age of 65-74 years. However, experts in the DDT at the CDC indicated that these declines were probably the result of selection bias since household based surveys such as the NHIS do not include institutionalized persons. These experts felt that elderly persons with diabetes were more likely to be in nursing homes and other institutionalized settings. Therefore, they are not adequately represented in the NHIS and NHANES surveys. Therefore, we allowed the prevalence of diabetes to increase across all age groups, but held the incidence of disease in the age groups 65-74 and 75+ years relatively stable in the DISMOD models. Published results form the NHANES III data as well as analyses conducted by USBODI staff indicated that approximately 1/3 (33.8%) of diabetes is undiagnosed. Therefore, the self-reported prevalence of diabetes in adults was increased by a factor of 1.51 (i.e. self-reported prevalence/[1-0.338]). There were insufficient numbers of Asians and Native Americans in the national surveys to produce estimates for these groups. However, there were enough deaths attributed to diabetes in Asians to derive estimates for this group by assuming identical case-fatality relationships in this racial subgroup as there are in Whites, and adjusting the incidence rates to the registered death rates for this disease.

Age and sex specific rates for sequelae form the Established Market Economy (EME) countries in the GBD were applied to the United States after adjusting for the prevalence of diabetes in the U.S. [24,25]. This was done by dividing the age and sex specific prevalence estimates of diabetes for the corresponding age and sex specific groups, within racial categories, in the United States. These ratios were then multiplied by the incidence rates for each of the sequelae supplied for the EME in the GBD.

DISMOD

Iteratively derived incidence rates for all diabetes (reported and undiagnosed) in adults were calculated using the DISMOD. During these calculations the empirical prevalence rates and relative risks were held constant. For children and adolescents, the incidence rates from the registries were entered and the prevalence was derived for each age group using the relative-risk for total mortality as described above. The attributable deaths from diabetes (provided as supplementary column in tables) are calculated using the relative risks.

The prevalence and duration of sequelae were estimated using DISMOD by holding the prevalence adjusted incidence rates (see above) from the EME countries constant and entering the increased relative risks for total mortality associated with each sequela (see table).

**4. Ischemic Heart Disease**

### Introduction

The methods outlined below build on the IHD disease model described in the Australian burden of disease studies, which assumes IHD starts as either angina pectoris or an acute myocardial infarction (AMI) [26]. It is acknowledged that these two conditions relate to the same disease process, however there are insufficient data at this point to model them together. Angina pectoris has been modeled as recurring attacks over the rest of the person's life, with possible remission due to treatment. AMI is assumed to result in death, heart failure, or recovery with zero disability weight. Death is assumed to follow the AMI duration given in the GBD 1990 study for EME countries. Heart failure is assumed to follow immediately after the AMI. The disease model focuses on AMI events rather than on people experiencing AMI. The incidence data, therefore, refer to the number of new AMI incidents in a year rather than people experiencing AMI for the first time. No attempt was made to model the risk of second and subsequent AMIs in a person who has experienced a first AMI.

### Data Sources

All cases of AMI in the US were assumed to either die or receive hospital treatment. Those who die before reaching hospital were assumed to make no contribution to YLD, hence the relevant AMI incidence for YLD modeling is the incidence of people admitted to hospital with AMI. Hospitalizations were derived from the 1996 Healthcare Cost and Utilization Project (HCUP) Nationwide Inpatient Sample (NIS) and adjusted to reflect incorrect coding of AMI-related hospital episodes using unpublished data from the Atherosclerosis Risk in Communities (ARIC) study [27]. Because almost a fifth of NIS AMI records had no valid race code, race-specific estimates were calculated as a proportion of all records with a valid race code.

Both the National Health and Nutrition Examination Survey III (NHANES III) and the National Health Interview Survey (NHIS) provide estimates of angina prevalence, the former being derived using the Rose algorithm and the latter being based on response to the question ‘have you ever been told by a doctor you have angina?’ When the Rose-positive group was restricted to include only those taking anti-anginal medication, there was a reasonable degree of convergence between both sources. Age-specific estimates from pooled NHIS data for the period 1991 to 1996 were the most plausible, however, and were therefore used in preference to those from NHANES III.

### Estimation Procedures

The GBD study has both treated and untreated weights for AMI, and the treated weight was used to apply to the period in treatment during and immediately after an attack. Because there are no reliable data for directly estimating the population distribution of angina severity states, we used the Australian IHD model. It was assumed that most cases of angina will be treated and hence maintained in a mild state for the duration of the illness (unlike the Australia model which assumes a mild state until the final year during which a severe state was assumed). For heart failure, the Rochester Epidemiology Project presents the proportion of incident cases by New York Heart Association class I-II and III-IV [28]. These proportions were used to derive an average of the Dutch weights, which are also defined in terms of the New York Health Association (NYHA) classification. Equal proportions were assumed in classes III and IV since these were grouped in the published results.

### DISMOD

Estimates of angina incidence and duration were modeled in DisMod from NHIS prevalence figures. A long-term clinical outcome study of bypass angioplasty revascularization showed that more than 15% of revascularized patients still experience angina after five years and that a significant proportion require multiple episodes of surgery before successful remission [29]. A similar figure is reported by Sergeant at five years, with a figure of 37% at 10 years [30]. In the absence of further information on efficacy at a population level, remission from angina was derived by subtracting a conservative estimate of 25% from NIS derived estimates of number of revascularized cases and dividing by estimated prevalence. The Australian DisMod models use a relative risk (RR) of dying of 2 to obtain the 1996 incidence and duration corresponding with the self-report prevalence. This figure is similar to the 1.6 reported from a 15-year follow-up study in the UK [31]. Pending further information, the Australian model was replicated using the NHIS prevalence and remission estimates presented above and a RR of 2.

Results from the Framingham study suggests that 20 per cent of patients who survived AMI develop heart failure [32]. This figure is confirmed by a more recent unpublished figure of 17% from the ARIC study. The ARIC figure was used as the relevant proportion for all ages in our model. Average duration of heart failure was modeled in DisMod using prevalence figures from NHANES III [33] and incidence figures from the Rochester Epidemiology Project assuming zero remission. Data from the Framingham Heart Study show a distinct survival advantage in women, with a median duration of 3.2 years compared to 1.7 years for men for all ages [34]. This converts to a mean of 4.6 years and 2.4 years assuming an exponential distribution in the probability of survival. These figures for all ages were entered into DisMod thus over specifying the model so as to derive an age-specific duration estimates with the correct gender differentials.

**5. Stroke**

### Introduction

For estimating hospitalized stroke in the US, we adopted the approach described by Williams [35] in which first-ever and recurrent stroke hospitalizations are identified by ICD-9 codes 430-438 in any diagnosis field. Williams et al argue against stroke ascertainment from hospital discharge data using other strategies (i.e. restricting to codes 430-436 only in the primary position) because improvements in the rate of false positives achieved using these strategies come at the expense of complete ascertainment. Four studies are cited that report virtually complete ascertainment using 430-438 in any diagnosis field but suboptimal positive predictive values (PPVs) for each ranging from 0% to 100%. The consistency between these studies in reported PPVs for each code leads Williams to conclude that over-ascertainment can be corrected with adjustment factors derived by pooling study-specific PPVs.

### Data Sources

Estimates of hospitalized stroke were derived from the 1996 Nationwide Inpatient Sample (NIS). Discharge level records were selected on the basis of having a code 430-438 on any diagnosis field. For records with more than one code in this range, only the first code was identified. Records were weighted to derive national estimates. Williams et al do not account for the fact that hospital discharge data can contain multiple records for the one event (because of follow-up visits, transfers etc). It is not possible to determine the magnitude of this problem from the NIS as a unique personal ID field is not retained in this dataset. Discharge database for the state of California does retain a personal ID, however, and an analysis of this data shows that about 8% of people with an admission for non-fatal stroke have one or more additional admissions with 30 days of the first admission. This figure is used to deflate the non-fatal estimates above.

### Estimation Procedures

There is conflicting evidence on the proportion of total stroke events that fail to present to hospital. The Northern Manhattan Stroke Study suggests about 5% and the Rochester study suggests a figure of about 15%. These estimates are not nationally representative and both are less than estimates from other studies (up to 28%) [36]. Williams et al applied a simple mean of the Manhattan and Rochester figures (10%) across all ages as an estimate of total (i.e.fatal and non-fatal) out-of-hospital stroke[[1]](#footnote-2). In the absence of further information, a flat 10% for non-fatal strokes below 75 years and 15% above 75 years were assumed. The flat age distribution seems unlikely for fatal strokes, however, given the increasing occurrence of stroke deaths outside hospital with increasing age [37]. For fatal strokes, therefore, we modeled an exponential increase based on the proportions reported in this study.

The overall estimate of total strokes of 781,334 is slightly more than Williams et al's estimate of >750,000 (but within their upper bound of 784,000) and Broderick et al's figure of 731,000 [38]. Notwithstanding these marginal differences, there are now a number of analyses suggesting the envelope of total stroke burden (first-ever and recurrent) in the US is closer to three quarters of a million strokes annually, rather than the oft-quoted figure of 500,000 from the American Heart Association.

There is consensus within the literature that the proportion of all stroke events in the US that are recurrent is around 25% to 35% [39-41]. Williams et al attempt to validate the 25-35% range by comparing their all stroke rates with first-ever rates from Rochester. Their conclusion is that, on average, the range is probably correct, but that it is age-dependent, with negligible re-occurrence rates up to 65 years then rates of 33%, 50% and 66% in older age groups (65-74, 75-84 and 85+ respectively). Taking these findings into account an exponential increase from age 45 was assumed maintaining an average of 30%. This results in 552,699 first-ever strokes in 1996.

Stroke-related 28th-day case-fatality was derived from the previously estimated fatal and total stroke events. Because out-of-hospital fatal strokes were simply an extrapolation of hospitalized fatal stroke events, this analysis is built on the assumption that most in-hospital stroke deaths occur within 28 days of a stroke event. The age-adjusted case-fatality using this method, however, is in the same range as the age-adjusted 28-day case-fatality from ARIC and the age-specific figures increase from 55 onwards as in other studies. These figures were therefore regarded as a reasonable proxy for 28-day case-fatality at a national level. Secular trends in survival and the uneven geographic distribution of stroke mortality make comparisons between studies from different periods and places that used different racial compositions, age groups and case definitions inappropriate. ARIC is the most relevant in terms of time and is comprised of a biracial population, although the figures are limited to 45 to 64 year olds at baseline [42].

### DISMOD

The Australian burden studies modeled stroke survivors past the first 28 days based on an extrapolation of mortality in this group. For the US, estimates of self-reported current and chronic stroke from the National Health Interview Survey are available. Dismod II was used to model duration from incidence and prevalence assuming zero remission and a relative risk (RR) of 4.36 for males and 3.63 for females from Framingham. The overall proportion of estimated stroke-attributable deaths to coded stroke deaths was lower than that reported in the Perth Community Stroke Study [43]. Duration for 28-day deaths was estimated from average length of stay for those who die in hospital divided by total number of estimated of 28-day deaths. Because different PPV values for each ICD9 code in the range 430-438 were used to estimate in-hospital deaths, the calculation was weighted by the contribution of each code to total in-hospital deaths.

The National Health Interview Survey also provides self-report data on the ADL status of those for whom stroke is the main cause of disability. This is divided into the following categories: unable to perform personal care needs/major activity, limited ability to perform personal care needs/major activity, no limitation. There are three disease weights for stroke corresponding to different levels of permanent impairment (mild, moderate, severe) developed in the Netherlands study [44]. Assuming that the first ADL (activities of daily living) category corresponds to severe permanent impairments, the second to moderate permanent impairments, mild impairment prevalence can be calculated by subtracting the severe and moderate estimates from the total prevalence of survivors with permanent impairments. Bonita reports that men are more likely to make a complete recovery from stroke (50%) than women (37%) [45]. Based on this finding, it was assumed in the Australian studies that half the male incident cases and 37% of women experienced mild disability for 6 months then recovered, and the remainder experienced permanent impairments. The prevalence of survivors with permanent impairments was therefore the total prevalence of survivors multiplied by 50% for men and 63% for women. An average disability weight for the permanently impaired survivors was then calculated as the prevalence-weighted sum of the three disability weights for mild, moderate and severe impairments. This analysis was undertaken using pooled NHIS data for 1991-1996.

**6. Non-fatal Injuries**

Previous burden studies have relied on the following assumptions regarding non-fatal injuries:

1. The majority of ‘severe’ non-fatal injuries are admitted as inpatients to a hospital;
2. The majority of non-fatal injuries treated in emergency departments but not admitted for further treatment can be regarded as ‘less severe’ than those that subsequently get admitted; and
3. Non-fatal injuries treated outside the hospital system do not result in significant disability.

These assumptions underpin the methods outlined below.

# Data Sources

In the absence of a national hospital discharge data collection system or the national emergency department (ED) data collection system in the US, we developed national estimates of non-fatal injuries based on data collected in four states; California, Washington, South Carolina and New York. Four state-based data collection systems on non-fatal injuries have been identified by CDC as being relatively complete (personal communication, Annest). The remaining data collection systems do not cover the entire population or do not obtain adequate cause of injury information (ICD-9 'E-codes’) from participating hospitals [46]. Other important sources of information on hospitalizations include the National Hospital Discharge Survey and the HCUP Nationwide Inpatient Sample, although only about two-thirds of records in these surveys provide information on cause.

South Carolina has been identified as having the most established and complete of the state based ED collection systems (personal communication, Annest). The remainder are either in their infancy, do not cover the entire population or do not obtain adequate E coding of injury related records from participating hospitals. The other important source of information on ED presentations is the National Hospital Ambulatory Medical Care Survey (NHAMCS), although this survey is comprised of a relatively small sample and also has E code quality issues.

# Estimation Procedures

Given the extent of incompleteness in cause coding for injury hospitalizations at a national level, a feasible approach to obtaining the information required for a burden of injury analysis was to model cause of injury estimates from the state collection systems with reputably good quality E-coding. A preliminary analysis of unit record data for 1996 from California, Washington, South Carolina and New York state revealed a small degree of ‘lazy’ E-coding (use of place only E-codes, tendency to use ‘Other/Ill-defined’ E-codes), multiple E-coding (CA allows up to five E-codes per record making causal attribution difficult) and incomplete race coding (WA does not collect race information; approximately 6% missing in others states).

Based on these initial findings, a number of preparatory transformations were performed on the data. Given that estimates were required by race, the WA dataset was excluded because race information is not collected in this state. Native Americans were grouped with Whites in the remaining datasets because of small numbers. Unit records were grouped into discrete injury ‘events’ for each person based on the assumption that admissions for the same E-code or N-code within 90 days of each other were re-admissions/transfers for on-going treatment following the initial injury event. A single ‘cause of injury’ category was assigned to each event after cleaning multiple E-codes and proportionately re-distributing missing/ill-defined/intent undetermined E-codes across known categories. Events with missing/unknown race, age and county codes were also proportionately re-distributed. Out-of-state county codes were regarded as missing rather than excluded to account for cross border patient flows.

The data were aggregated by age category, sex, race, county and cause and separate Poisson regression models were fitted to event counts for each cause and sex combination using population as the exposure variable and age category, mortality, Gini coefficient, educational attainment, per capita income, and proportion living in urban areas as the predictor variables (the last four were derived at a county level from the 1990 Census). Extensive evaluation of these models was undertaken using goodness-of-fit statistics and predicted versus observed plots. The effects of clustering on county and interactions between predictors were also explored. From these models out-of-sample predictions by age, sex, race, and cause were made for each county across the US. The results of these predictions were aggregated to derive national estimates of injury incidence by age, sex, race and cause. Cause-specific predictions were scaled to all-cause predictions in order to maintain consistency between totals.

Data from the three states were also used to determine the distribution of ‘nature of injury’ categories (‘N-codes’ in ICD-9) across cause categories. The classification system used was adapted from the GBD study. Ill-defined and multiple injury N-codes were redistributed to other categories using the GBD algorithm. Following the Australia burden studies, only the most disabling N-code associated with each injury event was considered on the assumption that the disability associated with the less severe co-existing injury would be captured in the disability weight for the more severe injury. The first most severe ‘nature of injury’ category was identified for each event using a pre-determined hierarchy based on both severity and duration of disability. After making these transformations, observed cause-specific ‘nature of injury’ category distributions were applied to the cause-specific predictions for each age, sex, and race stratum.

The National Hospital Discharge Survey (NHDS) provides an alternative source on information on hospitalized injuries in the US. This survey was used to validate the modeling procedure described above. It was not possible to compare cause- or race-specific estimates because of high proportion of missing E-codes in the NHDS and its relatively small sample size, therefore only all-cause estimates by nature of injury were explored. To retain consistency between the two data sources, the same N-code algorithms used to transform the modeled estimates were also applied to those derived from the survey. In addition, the survey estimates were deflated for re-admissions using factors for each age and nature of injury stratum derived from the state level data. The results of this analysis showed a high degree of convergence between the two sources of all-cause estimates (Figure 1). In summary, multivariate modeling of the state based data produced a credible picture of the incidence of non-fatal hospitalized injuries at a national level in terms of both causes and consequences. No attempt was made to validate the accuracy of injury coding in these data.

Figure 1 Modeled and survey estimates of all-cause admissions by nature of injury

Note: the number of events represents the number of specific injuries (i.e # skull: number of injuries to the skull)

An attempt to derive non-admitted ED incidence from predicted admissions by establishing a relationship between observed admission and ED presentation rates for each county in South Carolina was unsuccessful. A feasible alternative, therefore, to apply the same modeling approach for ED presentations as described for hospitalizations above. NHAMCS was used to validate the all-cause predictions and a high degree of convergence between the two sources of estimates was found (Figure 2). Certain types of injuries (i.e. fractures of the skull, vertebra, pelvis, femur and patella, spinal cord lesions, internal injuries, all amputations, burns >60% and injured nerves) were assumed to all result in admission and were therefore removed from the ED estimates. For the remaining categories, hospitalization estimates were subtracted from the ED estimates so as to avoid doubling counting the ED cases that were subsequently admitted.

Figure 2 Modeled and survey estimates of all-cause ED presentations by nature of injury

Note: the number of events represents the number of specific injuries (i.e # skull: number of injuries to the skull)

DISMOD

The GBD disability weights were adopted with minor modifications. All injuries were assumed to be treated. The GBD assumptions regarding the proportion of cases resulting in long-term disability for each nature of injury stratum were retained after checking against expert advice at CDC and findings in the literature. Life long durations for each age, sex and race stratum were derived from DisMod. For injuries with no excess risk of mortality (eg. amputations), remission and case-fatality rates of zero were assumed. For those associated with an increased risk of mortality (eg. head injuries, spinal cord injuries, fractured femur/hip), zero remission and relevant relative risks from the literature were assumed [47-52]. People who visit emergency room for injuries but are not admitted are assumed to have short-term disability only. The short-term durations used in the GBD study were retained with only minor modifications.

**7. Congenital Anomalies**

The report includes detailed estimates of deaths, YLL, YLD and DALYs for nine categories of births defects: anencephaly, anorectal atresia; clefts (lip/palate); oesophageal atresia; renal agenesis; Down syndrome; congenital heart anomalies; spina bifida; and congenital rubella. These categories correspond to the GBD classification with minor modifications, of which the most important is the inclusion of congenital rubella. These nine categories account for about half of all congenital anomalies, and 80 percent of total DALYs. Although detailed estimates are not provided for the other congenital anomalies, all are included in the total estimates provided for congenital anomalies in the Annex tables.

Congental heart abnomalities in the GBD and in this report include the ICD-9 category “other congenital anomalies of the circulatory system”. Estimates for congenital rubella include only the disease burden due to premature deaths as no data were available for the prevalence of congenital rubella.

Data Sources

We used the Metropolitan Atlanta Congenital Defects Program (MACDP) database to determine the birth prevalence of selected congenital anomalies. The MACDP was created by the CDC to develop a case-registry for use in epidemiologic and genetic studies. In 1997, Georgia State officially made the CDC its agent for the collection of information on congenital anomalies. Birth prevalence rates obtained from this data and another widely used birth defects registry in the United States, The California Birth Defects Monitoring Program, are generally similar.

All births and still births to mothers resident in 5 counties of the metropolitan Atlanta area, Georgia are evaluated in the MACDP. Records of infants/children born after the 20^th^ week of gestation with major structural or genetic defect diagnosed before the 6^th^ birthday and whose mothers were residents of the catchment area are searched by trained data abstractors for the ICD-9-CM codes for congenital anomalies (740-759.9) and for 46 other ICD-9-CM diagnosis codes that may indicate a codable condition. In 1995, these counties had an estimated population of 2.5 million, and 40,259 live births. Among resident live births, 40% were recorded as African American race. We obtained the number of deaths for each condition from the underlying cause of death data, 1996.

Estimation Procedures

In the MACDP, we searched for any mention of the ICD codes of selected congenital anomalies and calculated the rates using the estimated population in the catchment area. We obtained average of values obtained for years 1993 through1998. The population distribution of metropolitan Atlanta does not reflect the population distribution of the United States. Therefore, we race adjusted the birth prevalence obtained from the MACDP using the race distribution of the US population in 1996, National Center for Health Statistics. Many children with congenital anomalies have multiple defects. To adjust for double counting, the California Birth Defects Monitoring Program (CBDMP) has developed a hierarchy based on severity of the congenital defects. The proportion of less severe conditions (e.g. cleft palate) that are found in children with more severe ones (e.g. Down syndrome) should be taken out. For example, if 20 percent of children with Down syndrome also have clefts than estimates for cleft palates should be reduced by 20 percent and attributed to Down syndrome. We applied this hierarchy to the MACDP data to adjust for double counting.

We used DISMOD to estimate epidemiologic parameters of nonfatal congenital conditions. We assumed that birth prevalence of congenital anomalies equals incidence, there is no incidence after age 0, and congenital anomalies have a remission rate of 0. We then entered the birth prevalence data in the incidence column, and adjusted the case-fatality rates to approximate the age-specific number of deaths in the output column to that obtained from the underlying cause of death mortality data. We compared our findings with projections or findings from other studies including the birth prevalence rates and survival estimates from the CBDMP, and from literature. We assumed that children with anencephaly, abdominal wall defects, and bilateral renal agenesis do not survive beyond the first few days of birth. After careful review of the underlying cause of death and other analyses based on CBDMP and the literature, we excluded unilateral renal agenesis from our estimates. The underlying cause of death database includes a considerable number of deaths due to renal conditions in young and middle-aged people, some of which may be due to unilateral renal agenesis or digenesis. Other analyses bases on CBDMP and the literature confirmed survival at older ages for conditions including unilateral renal agenesis. However, it is believed that there is a discrete point beyond which survivors have mortality rates resembling that of the general population.

It is important to note that numbers of deaths and YLL by age and sex for the nine selected birth defects categories were based on U.S. death certificates for 1996. We are aware that in spite of efforts made to improve the quality of data on death certificates, varying degrees of misclassification may occur during coding leading to underreporting of some conditions (e.g. cardiovascular defects). This problem may also apply to a number of conditions other than congenital diseases. However, the burden of disease framework did not allow for the redistribution of reported causes of deaths, other than ill-defined categories and cardiovascular garbage codes, to ensure that the total number of deaths attributed to each GBD category did not exceed the total number of reported deaths. The redistribution algorithm applied to so-called cardiovascular garbage codes was based on careful analysis of large datasets from all OECD countries (see methods section).

# 8. Unipolar major depression and alcohol use

Unipolar major depression and alcohol use were the two leading neuropsychiatric disorders, which include mental disorders and nervous system disorders. Mental disorders can be divided into i) mood disorders (unipolar major depression, dysthymia, bipolar disorder, panic disorder, post-traumatic stress disorder (PTSD), obsessive-compulsive disorder, schizophrenia), and ii) substance-related disorders (alcohol and drug use).

**UNIPOLAR MAJOR DEPRESSION**

#### Data Sources

#### The Epidemiologic Catchment Area (ECA) Program (1980-1985) and the National Comorbidity Survey (NCS) (1990-1992) are the two main sources of data on the prevalence of mental disorders in the United States. Findings reported by the two surveys are not directly comparable as they different in the choice of survey instruments, definitions, and study design. Prevalence estimates reported in the NCS were higher than those reported in the ECA for both sexes and all age groups.

The ECA conducted a baseline assessment of 12,538 respondents ages 15 years and older, at four different sites, using the NIMH Diagnositc Interview Schedule (DIS). A follow up one year later in each site provided incidence estimates. The Baltimore Catchment Area Follow up interviewed 1,920 respondents enrolled in the initial study approximately ten years later (1993-1996).

The National Comorbidity Survey (NCS) provided data to estimate point prevalence and duration of unipolar major depression, bipolar disorder, panic disorder, and post-traumatic stress disorder (PTSD). The survey is a cross-sectional study, using the University of Michigan variant of the Composite International Diagnostic Schedule Interview (UM-CIDI) to diagnose these illnesses in a nationally representative sample of 8,098 respondents, ages 15-54 years. The UM-CIDI generates diagnoses according to the definitions and criteria for both the DSM-III-R and ICD-10 classification systems. DSM-III-R criteria were used for this study.

#### Estimation Procedures

Unipolar major depression (UMD) is a chronic, often remitting and relapsing illness. The frequency, duration and severity of episodes is highly variable in the general population, which posed difficult challenges for the evaluation of each component needed to estimate the morbidity burden – incidence, duration and severity. Estimation procedures were developed in close collaboration with Ron Kessler and William Eaton (who respectively directed the NCS and ECA study), and were discussed with a broader group of experts from the National Institute of Mental Health (NIMH).

##### *Incidence*

The GBD study defined incidence as the point prevalence, or prevalence of illness during the shortest time possible. In this study, incidence estimates were based on one-month prevalence data, which is the shortest period of time reported in the NCS.

###### *Duration*

A special analysis of the NCS data was carried out for this study to estimate the overall mean duration of UMD episodes. The duration was estimated separately for two groups: (a) those with more than one episode (n=859) and (b) those who has only one episode in their life (n=372). The average duration of the former group is around 17 weeks, and for the latter, 173 weeks (calculated with durations in excess of 1000 weeks truncated to 1000 weeks). Combining he two groups, the overall mean duration episodes was 22.6 weeks (just under 6 months), computed as the weighted average of the mean duration of single and multiple episodes of UMD. It was applied regardless of age, sex or race. Although based on different estimation procedures, the average duration of episodes, derived from the Baltimore ECA follow up for our study, was very similar.

###### *Severity of UMD in the general population*

Based on DSM-III R diagnostic criteria**[[2]](#footnote-3)**, twenty percent of all respondents had mild UMD, 65 percent moderate UMD, and 15 percent sever UMD. However, the severity distribution of UMD based on DSM-III R diagnostic criteria does not provide a good measure of the severity of health outcomes. The NCS questionnaire nevertheless included measures of social role impairment, work loss and mental stress, which provided the basis to develop disability weights for UMD in the US population. Each of these domains was divided into three levels: mild, moderate, and severe [53]. Based on this breakdown, 85% of males and 74% of females diagnosed with MDE had mild disabling health outcomes; 8% males and 15% females had moderately disabling health outcomes; and 7% males and 8% females had severe health outcomes.

*Severity weights for UMD*

Two sources provided disability weights to quantify the severity of health outcomes caused by UMD, the GBD and the Disability Weights for Diseases in the Netherlands. The Dutch disability weights were formalized for five domains in the EuroQol classification (EuroQol 5D) – mobility, self-care, usual activities, pain/discomfort, anxiety/depression and cognition. Each domain was coded as 1 (no problem), 2 (some problems), and 3 (unable to perform the activities, or extreme pain) (Table 1).

Table 1. Disability weights for UMD

| **Study** | **Disease stages** | **EQ 5D+ description** | **Disability weight *** |
| --- | --- | --- | --- |
| GBD (1996) | Untreated | NA | 0.600 |
|  | Treated | NA | 0.302 |
|  |  |  |  |
| Disability Weights for Diseases in the Netherlands (1997) | Mild | 112121 | 0.14 |
|  | Moderate | 122122 | 0.35 |
|  | Severe | 223232 | 0.76 |
|  | With psychosis, e.g. with delusions and/or hallucinations | 223233 | 0.83 |

* The scale for Dutch disability weights ranged from 0 (worst imaginable health state) to 1 (best imaginable health state). This scale is the inverse of the GBD scale. Disability weights presented here are equal to 1-Dutch weight.

The severity of impairments reported in the NCS (measures of social role impairment, work loss and mental stress), was linked to EuroQol domains of usual activities. The severity of anxiety/depression in the NCS was coded on a scale ranging from 1-9. Two domains with mild values or one domain with a mild value and one domain with a moderate value were classified as mild (1-3). Two domains with moderate values or one domain with a mild value and one domain with a severe value were classified as moderate (4-6). Two domains with severe values or one domain with a moderate value and one domain with a severe value were classified as severe (7-9). The choice of cut-off points to define mild, moderate, and severe depression in the NCS inevitably has a degree of arbitrariness. However, they were defined in consultation with experts.

Severity weights applied to quantify YLD due to UMD represent the population distribution of mild, moderate, and severe, weighted using the Dutch disability weights in each age group (Table 2).

Table 2. Composite disability weights by age and sex, based on the NCS*

- The age of respondents in the NCS ranged between 15-54 years.
- Disability weights for ages below 15 and above 54 equal values for these ages.

*Comorbidity*

Comorbidity is common between anxiety and affective disorders. In Australia, the share of comorbid disorders were allocated equally among anxiety and affective disorders. For the US we applied DSM-III-R diagnostic hierarchies[[3]](#footnote-4) to avoid possible double counting.

For comorbidity between affective and anxiety disorders, comorbid states were attributed to the affective illness. As a result, estimates provided for other anxiety disorders do not include cases comorbid with depression or bipolar illness.

Alcohol and drug use

*Data sources*

Estimates for alcohol and drug abuse and dependence were based on the National Longitudinal Alcohol Epidemiologic Survey (NLAES) provided estimates of the prevalence of alcohol and drug use disorders. Wave 1 of this survey provides cross-sectional data, using the AUDADIS diagnostic schedule, on substance use in a nationally representative sample of 42,862 respondents, ages18-75+ years. The field survey was carried out in 1992. NLAES over-sampled minorities and the younger age groups. DSM-IV definitions are used for diagnosis of alcohol and drug use disorders. Prevalence data was available by age, sex, and race for lifetime, prior to the past year, prevalence.

Estimates of the duration of alcohol use disorders were also derived from the NLAES. The survey included two questions pertaining to the longest period for which symptoms of abuse and/or dependence lasted. We averaged the longest duration reported by alcohol abuse and/or dependence was 4-5 years for males and 3-4 years for females. Given the episodic nature of symptoms due to alcohol abuse and/or dependence, were based on answers provided to the question “How many times in the last year have you had five or more drinks at one time”, and were estimated to be equal to fifteen percent of the time.

###### *Severity distribution of alcohol and substance use disorders*

Following the recommendation from Bridget Grant who was the primary investigator for the study, the severity distribution of substance use disorders was based on symptom counts. The NLAES included 41 questions related to the impairment caused by alcohol and/or dependence. 1-14 symptoms indicated a mild drinking; 15-18 symptoms, moderate drinking; and 29 or more symptoms, severe drinking. Using this severity distribution, we constructed a weighted average, using the following Dutch disability weights: mild drinking (defined as some physical or social problems caused by excessive alcohol intake): 0.11; moderate drinking (between problem drinking and manifest alcoholism): 0.33; and manifest alcoholism (defined as severe social problems caused by

alcohol intake): 0.55.

The approach to assess the severity of drug use disorders was similar and was derived from answers provided to 28 questions used to diagnose drug abuse and/or dependence. Based on symptom counts, 1-10 symptoms were designated as mild; 11-20 symptoms as moderate; and 21 symptoms or more as severe. These were the basis to compute composite disability weights for drug use disorders.

UMD, alcohol use disorders and drug use disorders are highly comorbid conditions. Comorbidity between alcohol use disorders and drug use disorders was attributed to drug use disorders because of they caused a higher disability than did alcohol use disorders. The comorbidity between substance use disorders and depression were included in estimates of UMD.

### DISMOD

NLAES epidemiological estimates provided the basis to compute race and age specific estimates of alcohol dependence and/or abuse in the US population. Drug use disorders, although presented as a single final estimate by age, sex and race, were modeled separately in 5 categories – marijuana abuse and dependence; heroin/methadone abuse and dependence; cocaine and opiate abuse and dependence; and stimulant/hallocinogen abuse and dependence. The NLAES did not include questions about duration or remission for the different drug use disorders, so that remission rates were those provided in the Victoria burden of disease study, assuming that the average duration of drug abuse for each category were similar in Australia and the United States.

Reference List

1. Centers for Disease Control and Prevention: **1993 revised classification system for HIV infection and expanded surveillance case definition for AIDS among adolescents and adults.** *MMWR Recomm Rep* 1992, **41:** 1-19.

2. Klevens RM, Fleming PL, Li J, Gaines CG, Gallagher K, Schwarcz S *et al*.: **The completeness, validity, and timeliness of AIDS surveillance data.** *Ann Epidemiol* 2001, **11:** 443-449.

3. Wang MC: **Analysis of retrospectively ascertained data in the presence of reporting delays.** *Journal of the American Statistical Association* 1992, **87:** 397-406.

4. Chu SY, Buehler JW, Lieb L, Beckett G, Conti L, Costa S *et al*.: **Causes of death among persons reported with AIDS.** *Am J Public Health* 1993, **83:** 1429-1432.

5. Palella FJ, Jr., Delaney KM, Moorman AC, Loveless MO, Fuhrer J, Satten GA *et al*.: **Declining morbidity and mortality among patients with advanced human immunodeficiency virus infection. HIV Outpatient Study Investigators.** *N Engl J Med* 1998, **338:** 853-860.

6. Bozzette SA, Berry SH, Duan N, Frankel MR, Leibowitz AA, Lefkowitz D *et al*.: **The care of HIV-infected adults in the United States. HIV Cost and Services Utilization Study Consortium.** *N Engl J Med* 1998, **339:** 1897-1904.

7. **Time from HIV-1 seroconversion to AIDS and death before widespread use of highly-active antiretroviral therapy: a collaborative re-analysis. Collaborative Group on AIDS Incubation and HIV Survival including the CASCADE EU Concerted Action. Concerted Action on SeroConversion to AIDS and Death in Europe.** *Lancet* 2000, **355:** 1131-1137.

8. Barnhart HX, Caldwell MB, Thomas P, Mascola L, Ortiz I, Hsu HW *et al*.: **Natural history of human immunodeficiency virus disease in perinatally infected children: an analysis from the Pediatric Spectrum of Disease Project.** *Pediatrics* 1996, **97:** 710-716.

9. Rosenberg PS: **Backcalculation models of age-specific HIV incidence rates.** *Stat Med* 1994, **13:** 1975-1990.

10. Holtgrave DR, Pinkerton SD: **Updates of cost of illness and quality of life estimates for use in economic evaluations of HIV prevention programs.** *J Acquir Immune Defic Syndr Hum Retrovirol* 1997, **16:** 54-62.

11. Karon JM, Rosenberg PS, McQuillan G, Khare M, Gwinn M, Petersen LR: **Prevalence of HIV infection in the United States, 1984 to 1992.** *JAMA* 1996, **276:** 126-131.

12. Wingo PA, Landis S, Parker S, et al: **Using cancer registry and vital statistics data to estimate the number of new cancer cases and deaths in the United States for the upcoming year.** *J Reg Management* 1998, **25:** 43-51.

13. Ries LAG, Kosary CL, Hankey BF, Miller BA, Clegg L, Edwards BK: *SEER Cancer Statistics Review, 1973-1996*. Bethesda, MD: National Cancer Institute; 1999.

14. National Center for Health Statistics CfDCaP. Detailed Mortality File. 1996. Hyattsville, MD, US Department of Health and Human Services.
Ref Type: Data File

15. Cancer Statistics Branch. A Guide to SEER*Stat: Version 2.0. 1999. Bethesda, MD, National Cancer Institute.
Ref Type: Report

16. Murray CJL, Lopez A: *Global Health Statistics: A compendium of incidence, prevalence and mortality estimates for over 200 conditions*, 1 edn. Cambridge, MA: Harvard University Press; 1996.

17. Miller DL, Weinstock MA: **Nonmelanoma skin cancer in the United States: incidence.** *J Am Acad Dermatol* 1994, **30:** 774-778.

18. Scotto J, Fears TR, Fraumeni JF. Incidence of nonmelanoma skin cancer in the United States, NIH Publication No. 83-2433. 1983. Washington, DC, Public Health Service.
Ref Type: Report

19. Halder RM, Bridgeman-Shah S: **Skin cancer in African Americans.** *Cancer* 1995, **75:** 667-673.

20. Dokheel TM: **An epidemic of childhood diabetes in the United States? Evidence from Allegheny County, Pennsylvania.Pittsburgh Diabetes Epidemiology Research Group.** *Diabetes Care* 1993, **16:** 1606-1611.

21. de VF, Dekker JM, Stehouwer CD, Nijpels G, Bouter LM, Heine RJ: **Similar 9-year mortality risks and reproducibility for the World Health Organization and American Diabetes Association glucose tolerance categories: the Hoorn Study.** *Diabetes Care* 2000, **23:** 40-44.

22. Gu K, Cowie CC, Harris MI: **Mortality in adults with and without diabetes in a national cohort of the U.S. population, 1971-1993.** *Diabetes Care* 1998, **21:** 1138-1145.

23. Portuese E, Orchard T: **Mortality in insulin-dependent diabetes.** In *Diabetes in America, NIH Publication No. 95-1468*. Edited by National Diabetes Data Group. Bethesda, MD: National Institutes of Health, National Institute of Diabetes and Digestive and Kidney Diseases; 1995:221-232.

24. Klein R, Klein BEK: **Vision disorders in diabetes.** In *Diabetes in America, NIH Publication No. 95-1468*. Edited by National Diabetes Data Group. Bethesda, MD: National Institutes of Health, National Institute of Diabetes and Digestive and Kidney Diseases; 1995:293-338.

25. Reiber GE, Boyko EJ, Smith DG: **Lower extremity foot ulcers and amputations in diabetes.** In *Diabetes in America, NIH Publication No. 95-1468*. Edited by National Diabetes Data Group. Bethesda, MD: National Institutes of Health, National Institute of Diabetes and Digestive and Kidney Diseases; 1995:409-427.

26. Mathers CD, Vos ET, Stevenson CE, Begg SJ: **The burden of disease and injury in Australia.** *Bull World Health Organ* 2001, **79:** 1076-1084.

27. Rosamond WD, Chambless LE, Folsom AR, Cooper LS, Conwill DE, Clegg L *et al*.: **Trends in the incidence of myocardial infarction and in mortality due to coronary heart disease, 1987 to 1994.** *N Engl J Med* 1998, **339:** 861-867.

28. Senni M, Tribouilloy CM, Rodeheffer RJ, Jacobsen SJ, Evans JM, Bailey KR *et al*.: **Congestive heart failure in the community: trends in incidence and survival in a 10-year period.** *Arch Intern Med* 1999, **159:** 29-34.

29. Feit F, Brooks MM, Sopko G, Keller NM, Rosen A, Krone R *et al*.: **Long-term clinical outcome in the Bypass Angioplasty Revascularization Investigation Registry: comparison with the randomized trial. BARI Investigators.** *Circulation* 2000, **101:** 2795-2802.

30. Sergeant P, Lesaffre E, Flameng W, Suy R, Blackstone E: **The return of clinically evident ischemia after coronary artery bypass grafting.** *Eur J Cardiothorac Surg* 1991, **5:** 447-457.

31. Lampe FC, Whincup PH, Wannamethee SG, Shaper AG, Walker M, Ebrahim S: **The natural history of prevalent ischaemic heart disease in middle-aged men.** *Eur Heart J* 2000, **21:** 1052-1062.

32. Cowie MR, Mosterd A, Wood DA, Deckers JW, Poole-Wilson PA, Sutton GC *et al*.: **The epidemiology of heart failure.** *Eur Heart J* 1997, **18:** 208-225.

33. American Heart Association. 2000 Heart and Stroke Statistical Update. 1999. Dallas, TX, American Heart Association.
Ref Type: Report

34. Ho KK, Pinsky JL, Kannel WB, Levy D: **The epidemiology of heart failure: the Framingham Study.** *J Am Coll Cardiol* 1993, **22:** 6A-13A.

35. Williams A: **Advances in secondary prevention of stroke.** *Practitioner* 1999, **243:** 222-225.

36. Sacco RL, Boden-Albala B, Gan R, Chen X, Kargman DE, Shea S *et al*.: **Stroke incidence among white, black, and Hispanic residents of an urban community: the Northern Manhattan Stroke Study.** *Am J Epidemiol* 1998, **147:** 259-268.

37. Wein TH, Smith MA, Morgenstern LB: **Race/ethnicity and location of stroke mortality: implications for population-based studies.** *Stroke* 1999, **30:** 1501-1505.

38. Broderick J, Brott T, Kothari R, Miller R, Khoury J, Pancioli A *et al*.: **The Greater Cincinnati/Northern Kentucky Stroke Study: preliminary first-ever and total incidence rates of stroke among blacks.** *Stroke* 1998, **29:** 415-421.

39. Brown RD, Whisnant JP, Sicks JD, O'Fallon WM, Wiebers DO: **Stroke incidence, prevalence, and survival: secular trends in Rochester, Minnesota, through 1989.** *Stroke* 1996, **27:** 373-380.

40. Lackland DT, Bachman DL, Carter TD, Barker DL, Timms S, Kohli H: **The geographic variation in stroke incidence in two areas of the southeastern stroke belt: the Anderson and Pee Dee Stroke Study.** *Stroke* 1998, **29:** 2061-2068.

41. Terent A: **[Costs of stroke care. Lower costs in spite of increased incidence].** *Lakartidningen* 1993, **90:** 2758-2763.

42. Rosamond WD, Folsom AR, Chambless LE, Wang CH, McGovern PG, Howard G *et al*.: **Stroke incidence and survival among middle-aged adults: 9-year follow-up of the Atherosclerosis Risk in Communities (ARIC) cohort.** *Stroke* 1999, **30:** 736-743.

43. Anderson CS, Jamrozik KD, Broadhurst RJ, Stewart-Wynne EG: **Predicting survival for 1 year among different subtypes of stroke. Results from the Perth Community Stroke Study.** *Stroke* 1994, **25:** 1935-1944.

44. Stouthard M, Essink-Bot ML, Bonsel GJ, Barendregt JJ, Kramers P, van de Water H *et al*.: *Disability Weights for Diseases in the Netherlands*. Rotterdam, the Netherlands: Department of Public Health, Erasmus University; 1997.

45. Bonita R, Anderson CS, Broad JB, Jamrozik KD, Stewart-Wynne EG, Anderson NE: **Stroke incidence and case fatality in Australasia. A comparison of the Auckland and Perth population-based stroke registers.** *Stroke* 1994, **25:** 552-557.

46. Annest JL, Mercy JA, Gibson DR, Ryan GW: **National estimates of nonfatal firearm-related injuries. Beyond the tip of the iceberg.** *JAMA* 1995, **273:** 1749-1754.

47. Baguley I, Slewa-Younan S, Lazarus R, Green A: **Long-term mortality trends in patients with traumatic brain injury.** *Brain Inj* 2000, **14:** 505-512.

48. Cooper C: **The crippling consequences of fractures and their impact on quality of life.** *Am J Med* 1997, **103:** 12S-17S.

49. DeVivo MJ, Krause JS, Lammertse DP: **Recent trends in mortality and causes of death among persons with spinal cord injury.** *Arch Phys Med Rehabil* 1999, **80:** 1411-1419.

50. Division of Acute Care RRaDP. *Traumatic Brain Injury in the United States: A Report to Congress*. 1999. National Center for Injury Prevention and Control, Centers for Disease Control and Prevention, U.S. Department of Health and Human Services.
Ref Type: Report

51. Kraus JF, Black MA, Hessol N, Ley P, Rokaw W, Sullivan C *et al*.: **The incidence of acute brain injury and serious impairment in a defined population.** *Am J Epidemiol* 1984, **119:** 186-201.

52. Surkin J, Gilbert BJ, Harkey HL, III, Sniezek J, Currier M: **Spinal cord injury in Mississippi. Findings and evaluation, 1992-1994.** *Spine* 2000, **25:** 716-721.

53. Kessler RC, DuPont RL, Berglund P, Wittchen HU: **Impairment in pure and comorbid generalized anxiety disorder and major depression at 12 months in two national surveys.** *Am J Psychiatry* 1999, **156:** 1915-1923.

1. In fact, Williams et al simply inflate their hospital estimates by 1.1, resulting in an out-of-hospital to total proportion of 9.1%. [↑](#footnote-ref-2)
2. DSM-III R criteria:

   One of the symptoms must be

   (1) Depressed mood (feeling low, blue, moody)

   Or

   (2) Loss of interest/pleasure for a period of at least two weeks

   Other symptoms, “which must be present during the same two-week period and represent a change from previous functioning,” are:

   (3) Significant weight loss/gain, or increase/decrease in appetite nearly every day

   (4) Insomnia or hypersomnia nearly every day

   (5) Psychomotor agitation or retardation nearly every day (observable by others)

   (6) Fatigue or loss of energy nearly every day

   (7) Feelings of worthlessness or excessive/inappropriate guilt nearly everyday

   (8) Diminished ability to think or concentrate, or indecisiveness, nearly every day

   (9) Recurrent thoughts of death, suicidal ideation, or suicidal planning or attempt [↑](#footnote-ref-3)
3. The hierarchy rules: a) Organic mental disorders pre-empt diagnosis of other mental illness and b) When a pervasive disorder shares many symptoms with a less common disorder, the more pervasive disorder is diagnoses. From Introduction of DSM-III-R. [↑](#footnote-ref-4)
