# Supplementary material for: The burden of disease and injury in the United States 1996
Source: Popul Health Metr. 2006 Oct 18;4:11. doi: 10.1186/1478-7954-4-11 (PMC1635736; doi:10.1186/1478-7954-4-11)
Supplement: Additional File 3 — US burden of disease study classification system for diseases and injuries. The detailed list of causes selected for the USBODI. [file 1478-7954-4-11-S3.doc]

| **Title of USBODI cause** **ICD-9 Code** | ICD-9 Code |
| --- | --- |
| I. Communicable, maternal, perinatal and nutritional conditions | 001-139, 243, 260-269, 280-285, 320-322, 381-382, 460-465, 466, 480-487, 614-616, 630-676, 760-779 |
| A. Infectious and parasitic diseases | 001-139, 320-322,614-616 |
| 1. Tuberculosis | 010-018, 137 |
| 2. STDs excluding HIV | 090-099, 614-616 |
| a. Syphilis | 090-097, 614 |
| b. Chlamydia | 99.5 |
| c. Gonorrhoea | 98 |
| 3. HIV | 042-044 |
| 4. Diarrhoeal diseases | 001, 002, 004, 006-009 |
| 5. Vaccine Preventable Diseases | 032, 033, 037, 045, 050, 055, 056, 138 |
| a. Pertussis | 33 |
| b. Tetanus | 037, 771.3 |
| 6. Bacterial Meningitis and Meningococcemia | 036, 320-322 |
| 7. Hepatitis B and C | 070.2-070.9 |
| 8. Septicemia | 38 |
| 9. Intestinal Nematodes | 126-129 |
| B. Respiratory Infections | 460-466,480-487,381-382 |
| 1. ALRI | 466, 480-487 |
| 2. AURI | 460-465 |
| 3. Otitis Media | 381-382 |
| C. Maternal Conditions | 630-676 |
| 1. Hemmorrhage | 640, 641, 666 |
| 2. Sepsis | 670 |
| 3. Hypertensive disorders of pregnancy | 642 |
| 4. Abortion | 630-639 |
| D. Conditions arising during the perinatal period | 760-779 |
| 1. Low Birth Weight | 764-765 |
| 2. Birth Asphyxia and Birth Trauma | 767-770 |
| 3. Respiratory Distress Syndrome and other respiratory conditions |  |
| 4. Infections specific to the neonatal period |  |
| E. Nutritional Deficiencies | 260-269, 280-285 |
| 1. Protein-Energy Malnutrition | 260-263 |
| 2. Iron-Deficiency Anemia | 280 |

| **Title of USBODI cause** | **ICD-9 Code** |
| --- | --- |
| II. Noncommunicable diseases | 140-242, 244-259, 270-279, 286-319, 323-380,  383-459, 467-479, 488-613, 617-629, 680-759 |
| A. Malignant neoplasms | 140-209 |
| 1. Mouth and oropharynx cancer | 140-149 |
| 2. Esophagus cancer | 150 |
| 3. Stomach cancer | 151 |
| 4. Small Intestine cancer | 152 |
| 5. Colon/Rectum cancer | 153, 154 |
| 6. Liver cancer | 155 |
| 7. Pancreas cancer | 157 |
| 8. Trachea/Bronchus/Lung cancer | 162 |
| 9. Melanoma cancer | 172 |
| 10. Non-melanoma skin cancer | 173 |
| 11. Breast cancer | 174 |
| 12. Cervix cancer | 180 |
| 13. Corpus Uteri cancer | 179, 182 |
| 14. Ovary cancer | 183 |
| 15. Prostate cancer | 185 |
| 16. Bladder cancer | 188 |
| 17. Kidney and renal pelvis cancer | 189 |
| 18. Brain cancer | 191 |
| 19. Lymphomas | 200-202 |
| a. Hodgkin's disease | 201 |
| b. Non-Hodgkin's lymphoma | 200.0-200.8, 202.0-202.2, 202.8-202.9 |
| 20. Multiple Myeloma | 203.0, 203.2-203.8 |
| 21. Leukemias | 204-208 |
| a. Lymphocitic (acute, chronic, other) | 204 |
| b. Myeloid (acute, chronic, other) | 205 |
| c. Monocytic (acute, chronic, other) | 206 |
| B. Other Neoplasms | 210-239 |
| C. Diabetes mellitus | 250 |
| D. Nutritional/Endocrine | 240-242, 244-249, 251-259, 270-279, 281-289 |
| E. Neuro-psychiatric conditions | 290-319, 323-359 |
| 1. Unipolar Major Depression | 296.1-296.3 |
| 2. Bipolar Disorder | 296, 296.0, 296.4-296.9 |
| 3. Schizophrenia | 295 |
| 4. Epilepsy | 345 |
| 5. Alcohol use | 291, 303, 305.0 |
| 6. Alzheimer's and other dementia | 330, 331, 290 |
| 7. Parkinson's disease | 332 |
| 8. Multiple sclerosis | 340 |
| 9. Drug use | 304, 305.2-305.9 |

| **Title of USBODI cause** | **ICD-9 Code** |
| --- | --- |
|  |  |
| 10. PTSD | No ICD-9 code |
| 11. Obsessive-Compulsive Disorder | 300.3 |
| 12. Panic Disorder | 300.2 |
| F. Sense organ diseases | 360-380, 383-389 |
| 1. Glaucoma | 365 |
| 2. Cataracts | 366 |
| G. Cardiovascular diseases | 390-459 |
| 1. Rheumatic heart disease | 390-398 |
| 2. Ischemic heart disease | 410-414, Proportion of: 428, 427.1, 427.4, |
|  | 427.5, 440.9, 429.0-429.2, 429.9 |
| 3. Cerebrovascular disease | 430-438 |
| a. Intracerebral and intracranial hemorrhage | 430-432 |
| b. Cerebral infarction | 433-438 |
| 4. Inflammatory Cardiac | 394-397 |
| a. Acute and sub-acute endocarditis |  |
| b. Cardiomyopathy |  |
| 5. Valvular disorders | 424 |
| 6. Aortic aneurysm | 441 |
| 7. Hypertension and Hypertensive heart disease | 401-402 |
| 8. Pulmonary embolism | 415.1 |
| 9. Conduction Disorders and Other Drsrythmias |  |
| 10. Peripheral vascular disorders |  |
| H. Respiratory diseases | 470-478, 490-519 |
| 1. COPD | 490-492, 495-496 |
| 2. Asthma | 493 |
| I. Digestive diseases | 530-579 |
| 1.Oesophagus | 530 |
| 2. Peptic Ulcer Disease | 531-533 |
| 3. Cirrhosis of the Liver | 571 |
| a. alcoholic |  |
| b. non-alcoholic |  |
| 4. Appendicitis | 540-543 |
| 5. Gastro-intestinal haemorhage |  |
| J. Genito-urinary diseases | 580-611, 617-629 |
| 1. Nephritis/Nephrosis | 580-589 |
| 2. Benign Prostatic Hypertension | 600 |
| K. Skin diseases | 680-709 |
| L. Musculo-skeletal diseases | 710-739 |
| 1. Rheumatoid arthritis | 714 |
| 2. Osteoarthritis | 715 |

| **Title of USBODI cause** | **ICD-9 Code** |
| --- | --- |
| M. Congenital anomalies | 740-759 |
| 2. Anencephaly | 740 |
| 3. Anorectal atresia | 751.2 |
| 4. Cleft lip | 749.1 |
| 5. Cleft palate | 749 |
| 6. Oesophageal atresia | 750.3 |
| 7. Renal agenesis | 753 |
| 8. Down syndrome | 758 |
| 9. Congenital heart anomalies | 745-747 |
| 10. Spina bifida | 741 |
| 11. Congenital Rubella | 771 |
| N. Oral health | 520-529 |
| 1. Dental Caries | 521 |
| 2. Peridontal disease | 523 |
| O. Sudden Infant Death Syndrome |  |
|  |  |
| III. Injuries | E800-999 |
| A. Unintentional | E800-921, E923-949 |
| 1. Motor Vehicle Accidents | E810-819, E826-829, E928-929 |
| a. Occupants | xxx.0-xxx.6, xxx.8 |
| b. Pedestrians | xxx.7 |
| 2. Poisonings | E850-869 |
| 3. Falls | E880-888 |
| 4. Fires | E890-899 |
| 5. Drownings | E910 |
| 6. Accidental firearms | E922 |
| B. Intentional injuries | E922, 950-979, 990-999 |
| 1. Self-inflicted | E950-959 |
| a. by firearm | E955 |
| b. other | E950-954, E956-959 |
| 2. Violence | E922, E960-969, E965.0 |
| a. by firearm | E965.4, E960-964, E965.5 |
| b. other | E969, E970-978 |
| 3. War | E990-999 |
