# Supplementary material for: The burden of disease and injury in the United States 1996
Source: Popul Health Metr. 2006 Oct 18;4:11. doi: 10.1186/1478-7954-4-11 (PMC1635736; doi:10.1186/1478-7954-4-11)
Supplement: Additional File 4 — US burden of disease – Detailed tabulations of deaths, YLL, YLD and DALYs. Detailed tabulations of deaths, YLL, YLD and DALYs for the 73 causes included in the USBODI by age, gender and race. [file 1478-7954-4-11-S4.ppt]

## Slide 1
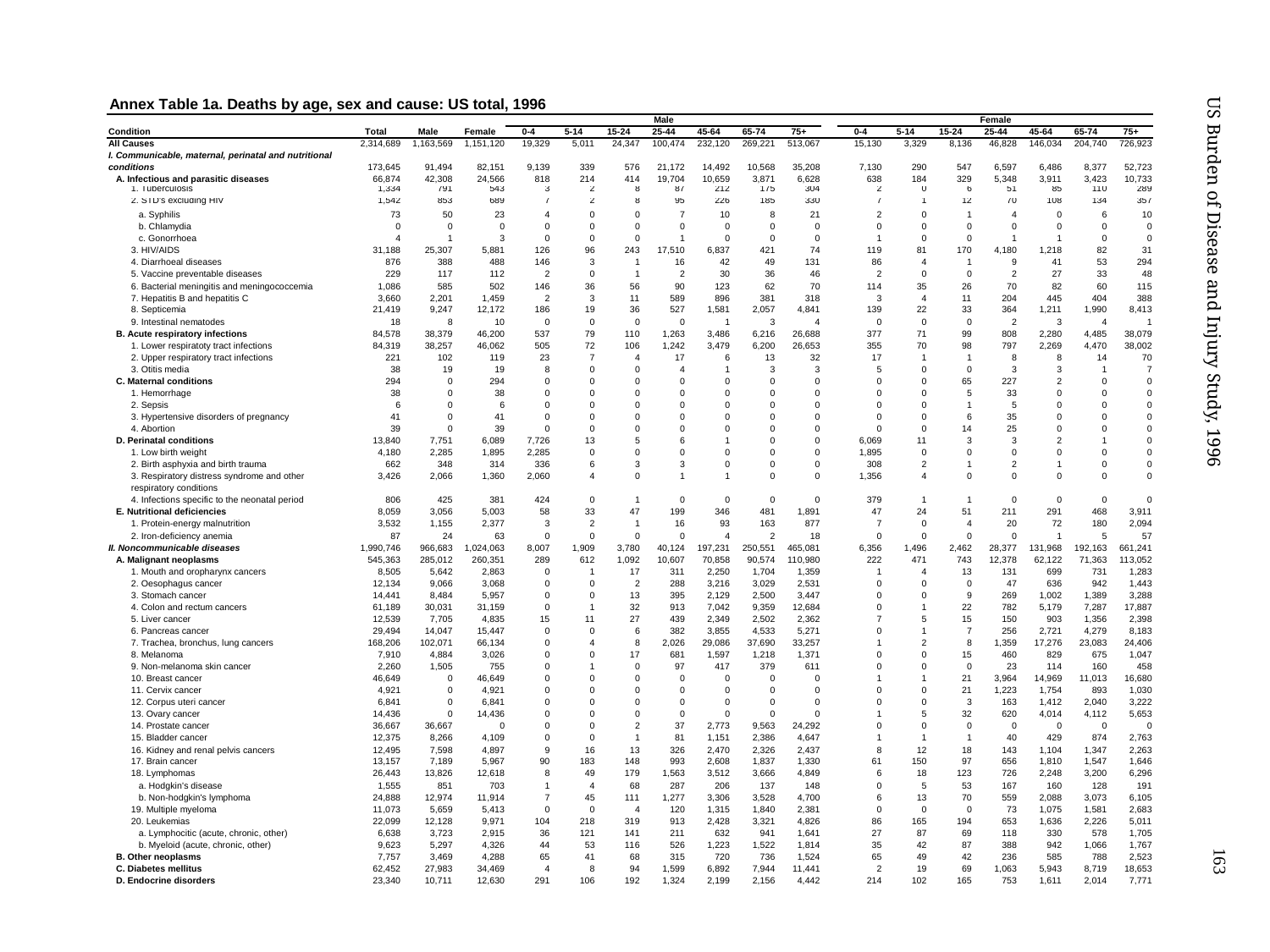

US Burden of Disease and Injury Study, 1996
163

## Slide 2
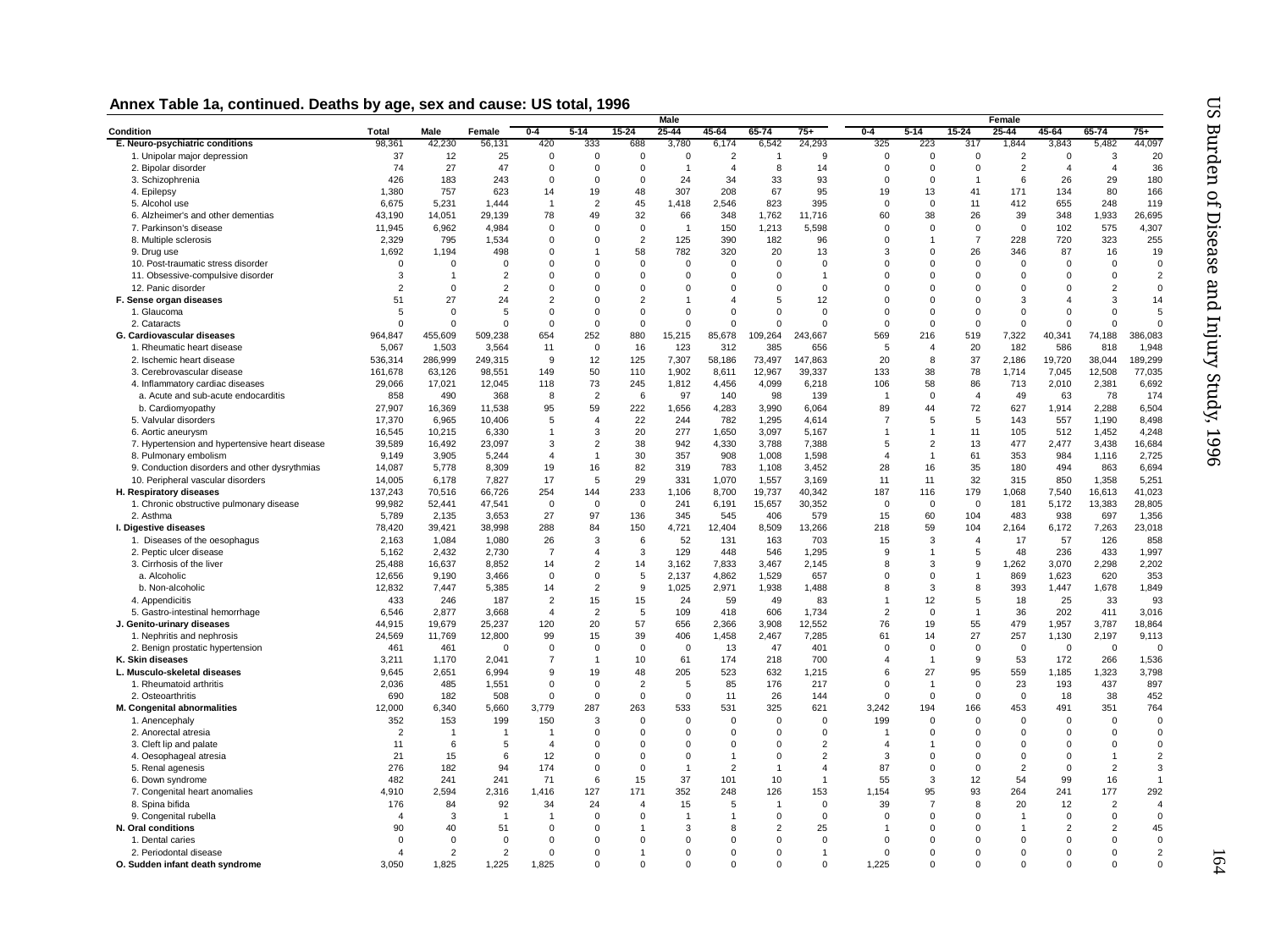

US Burden of Disease and Injury Study, 1996
164

## Slide 3
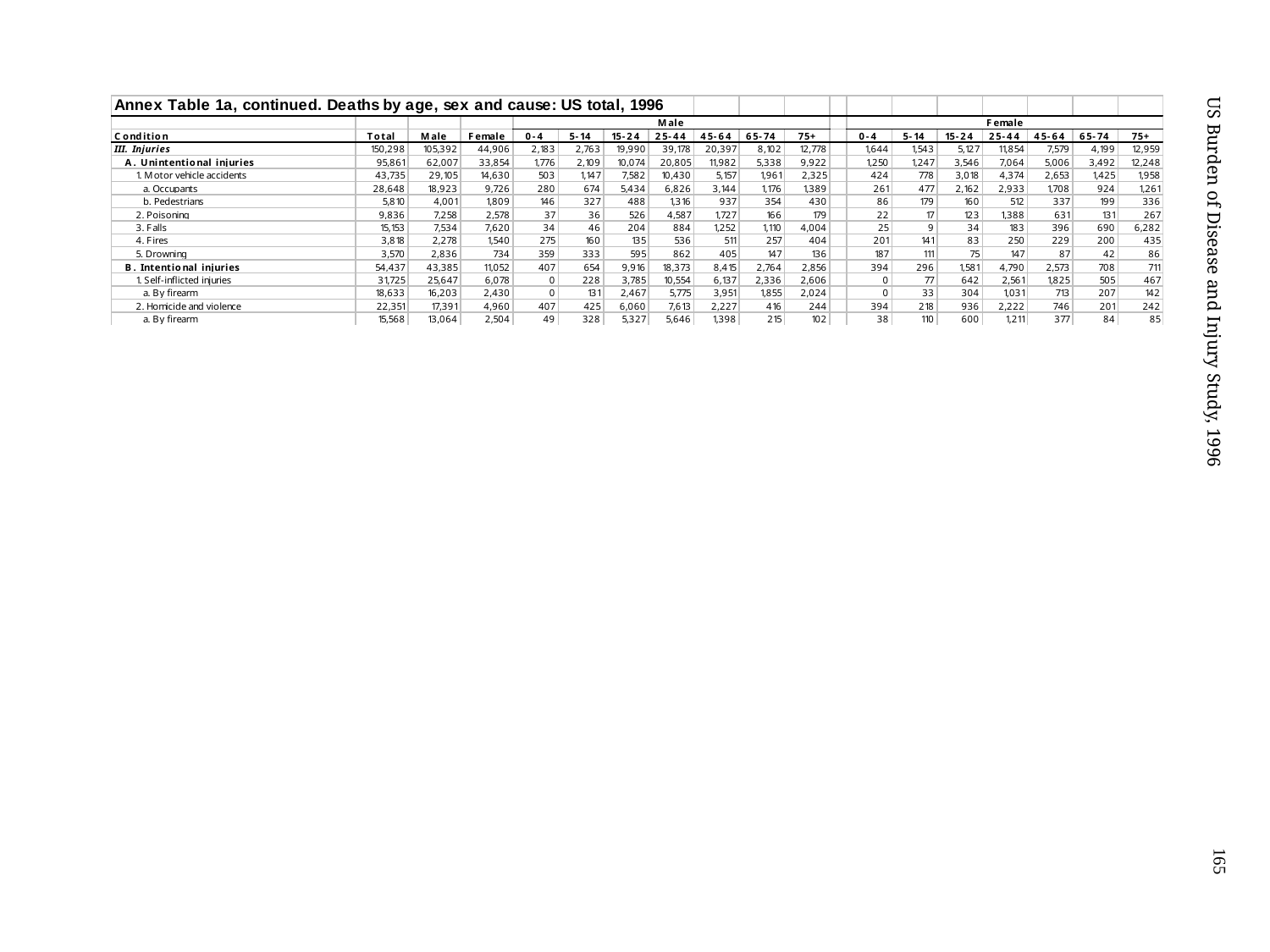

US Burden of Disease and Injury Study, 1996
165

## Slide 4
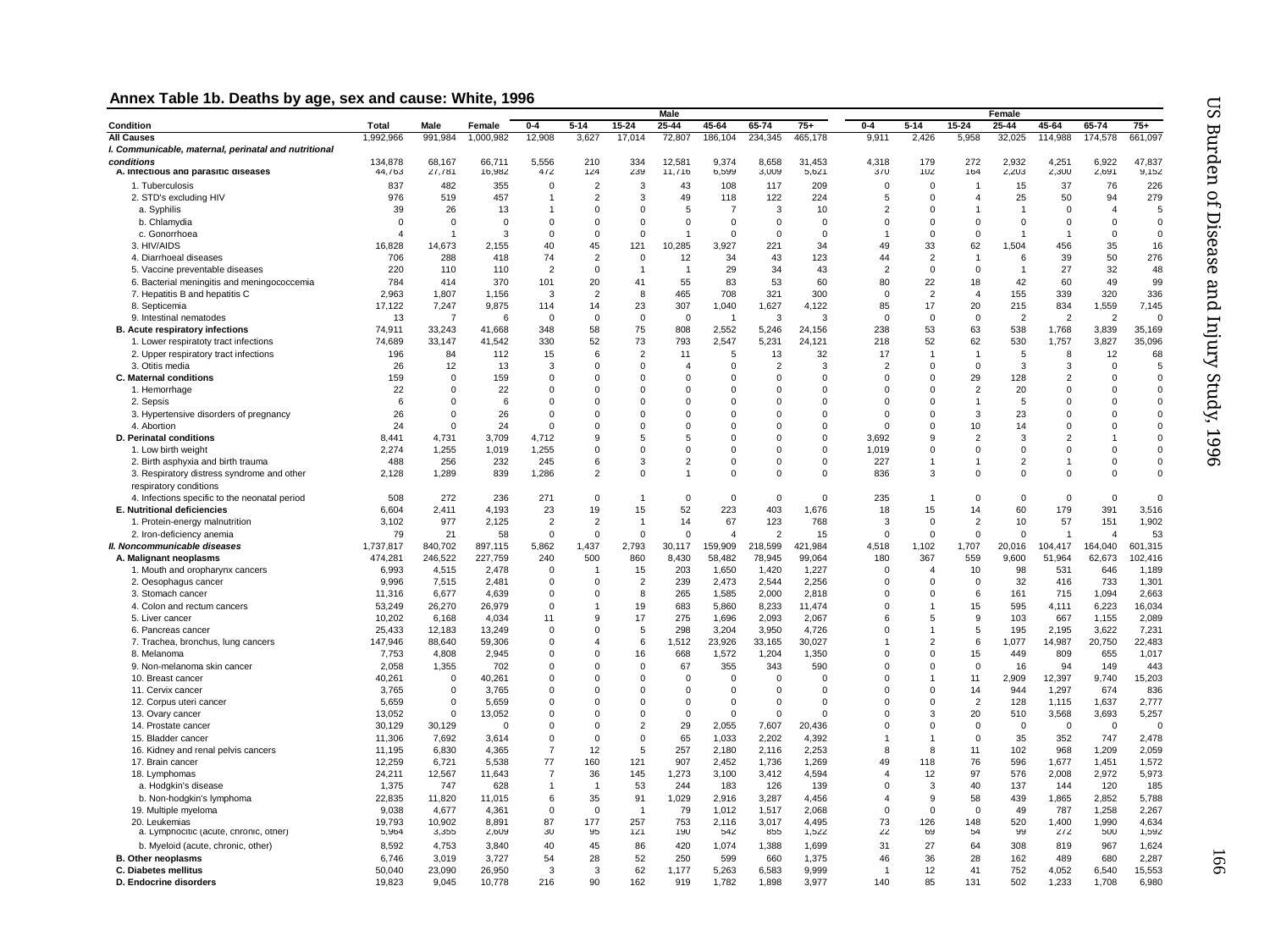

US Burden of Disease and Injury Study, 1996
166

## Slide 5
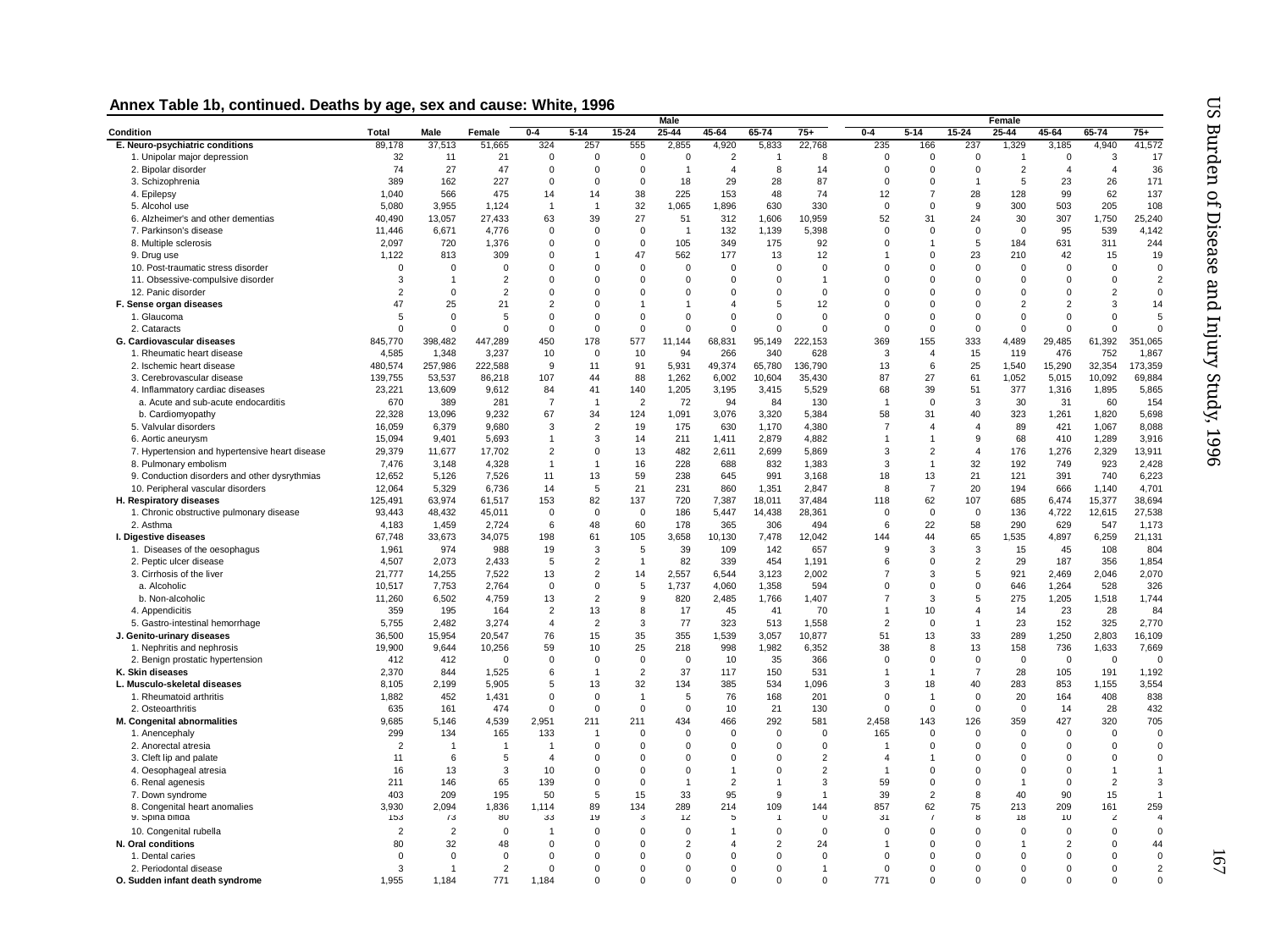

US Burden of Disease and Injury Study, 1996
167

## Slide 6
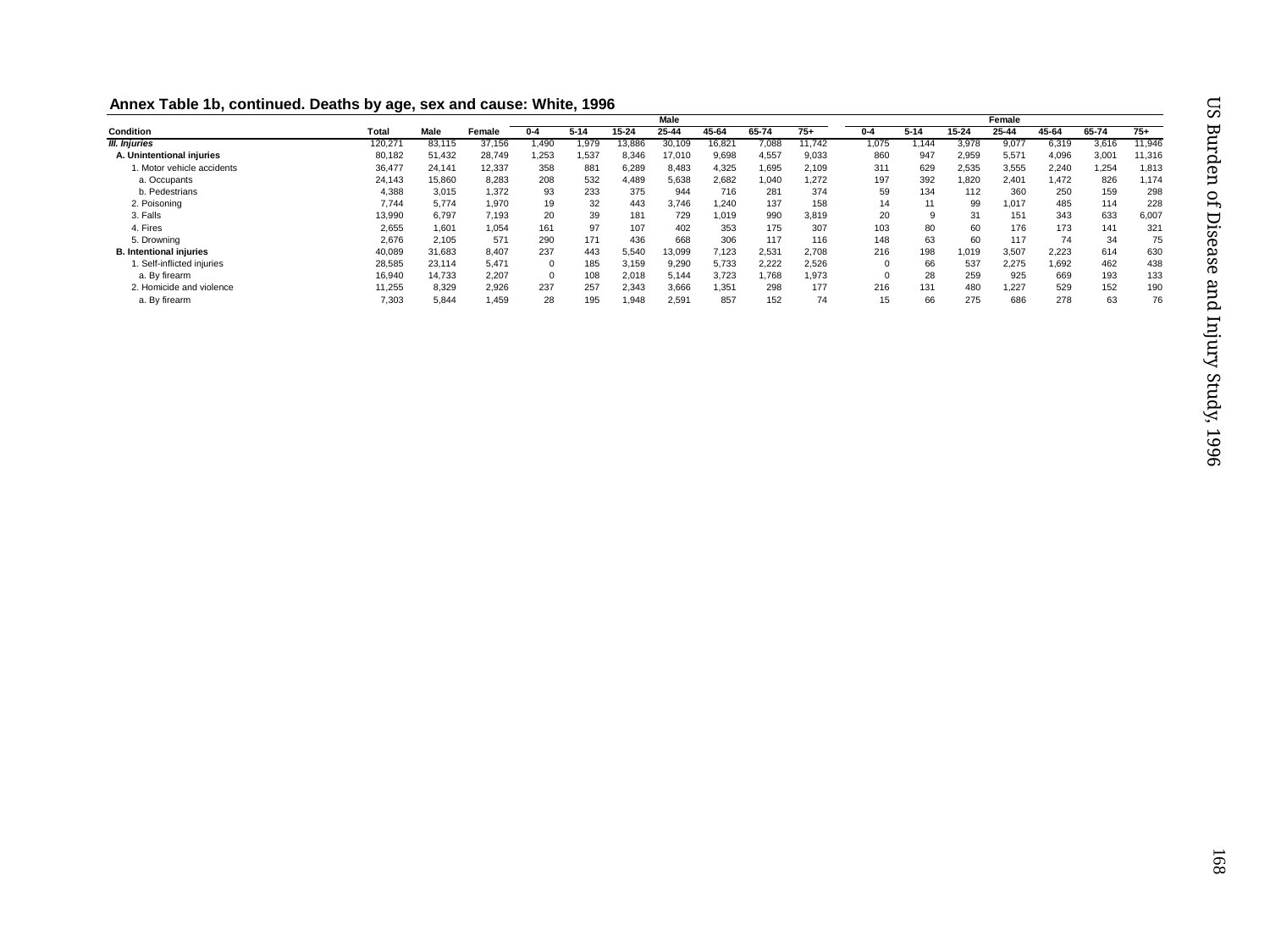

US Burden of Disease and Injury Study, 1996
168

## Slide 7
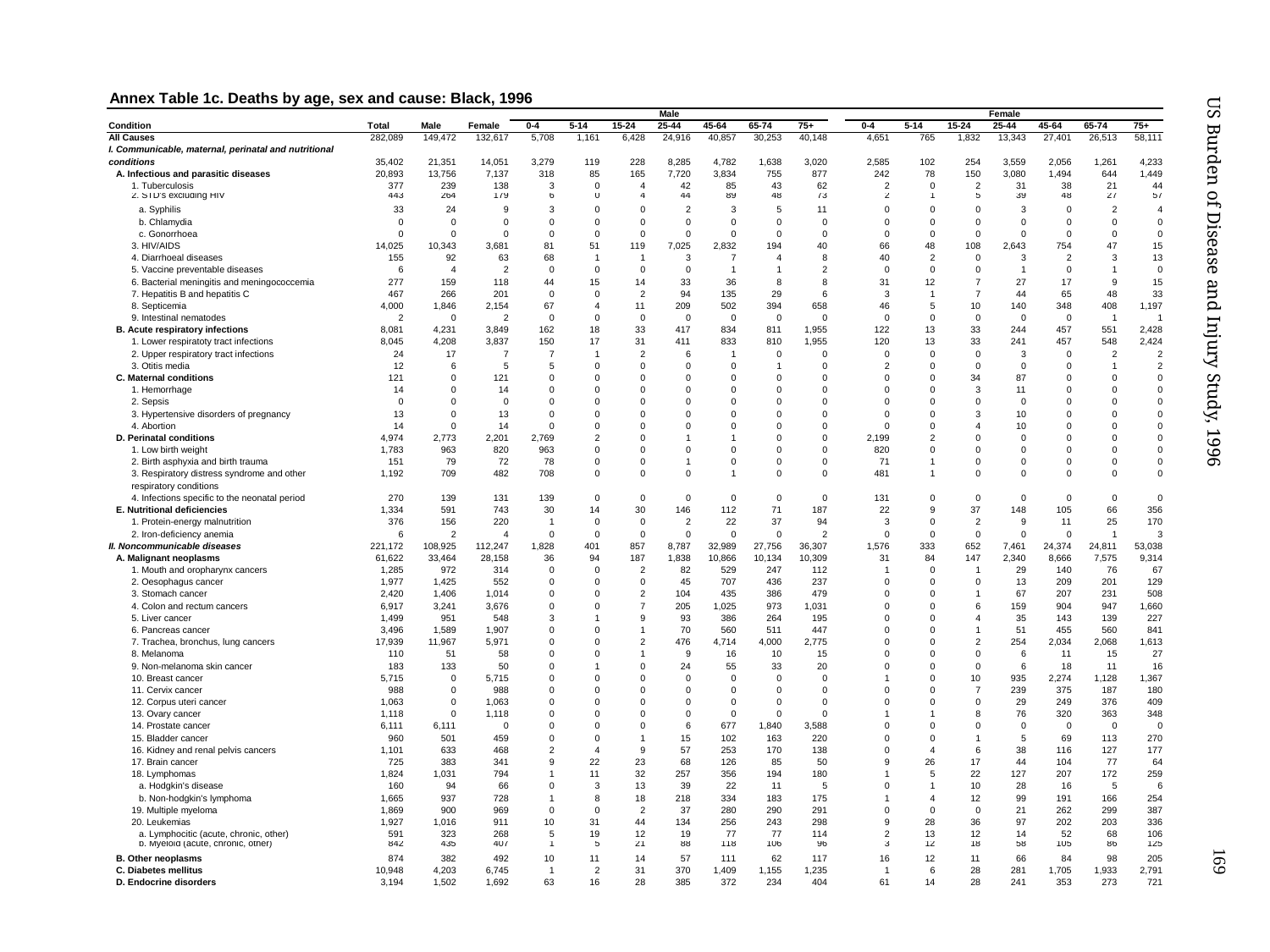

US Burden of Disease and Injury Study, 1996
169

## Slide 8
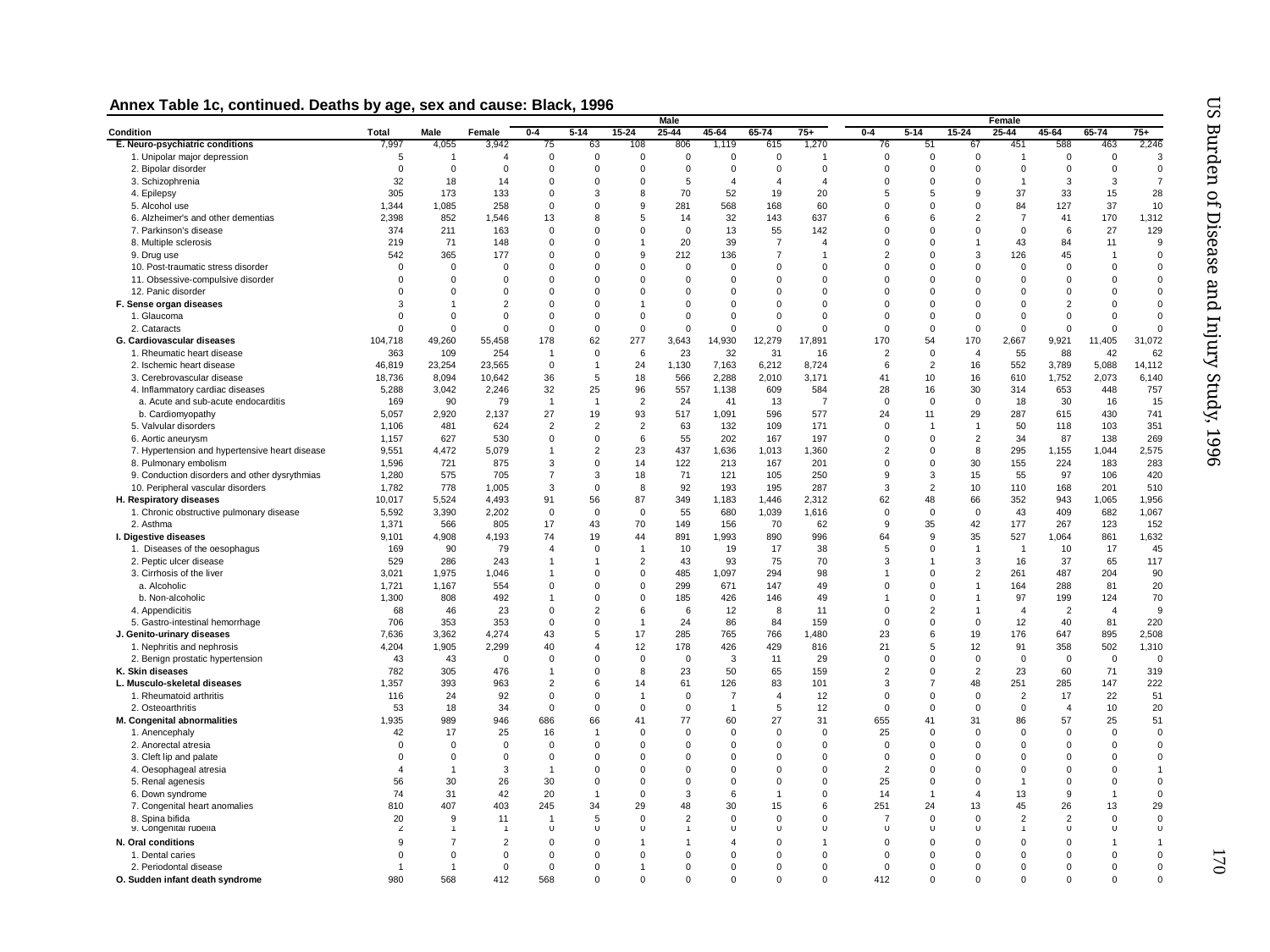

US Burden of Disease and Injury Study, 1996
170

## Slide 9
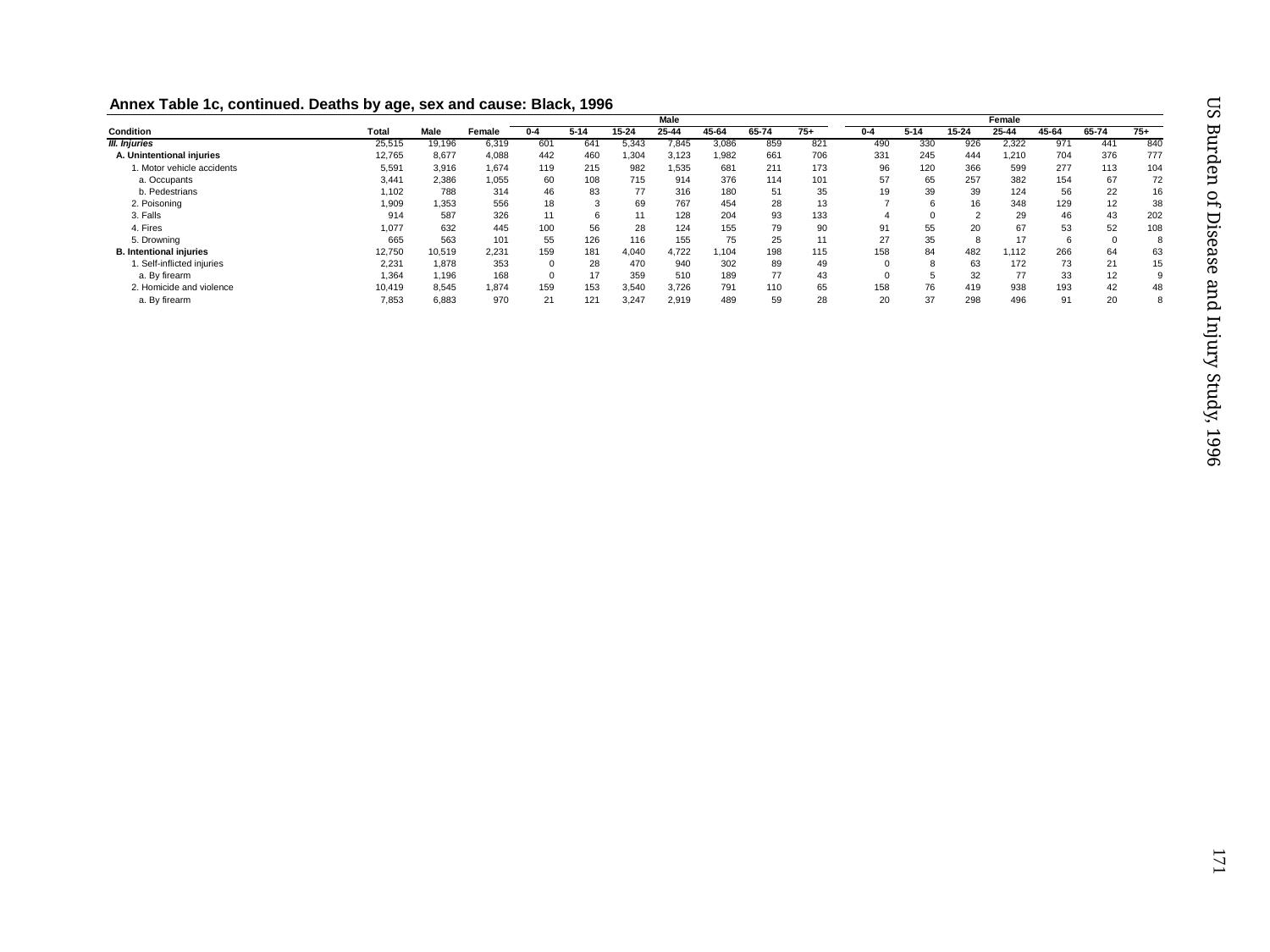

US Burden of Disease and Injury Study, 1996
171

## Slide 10
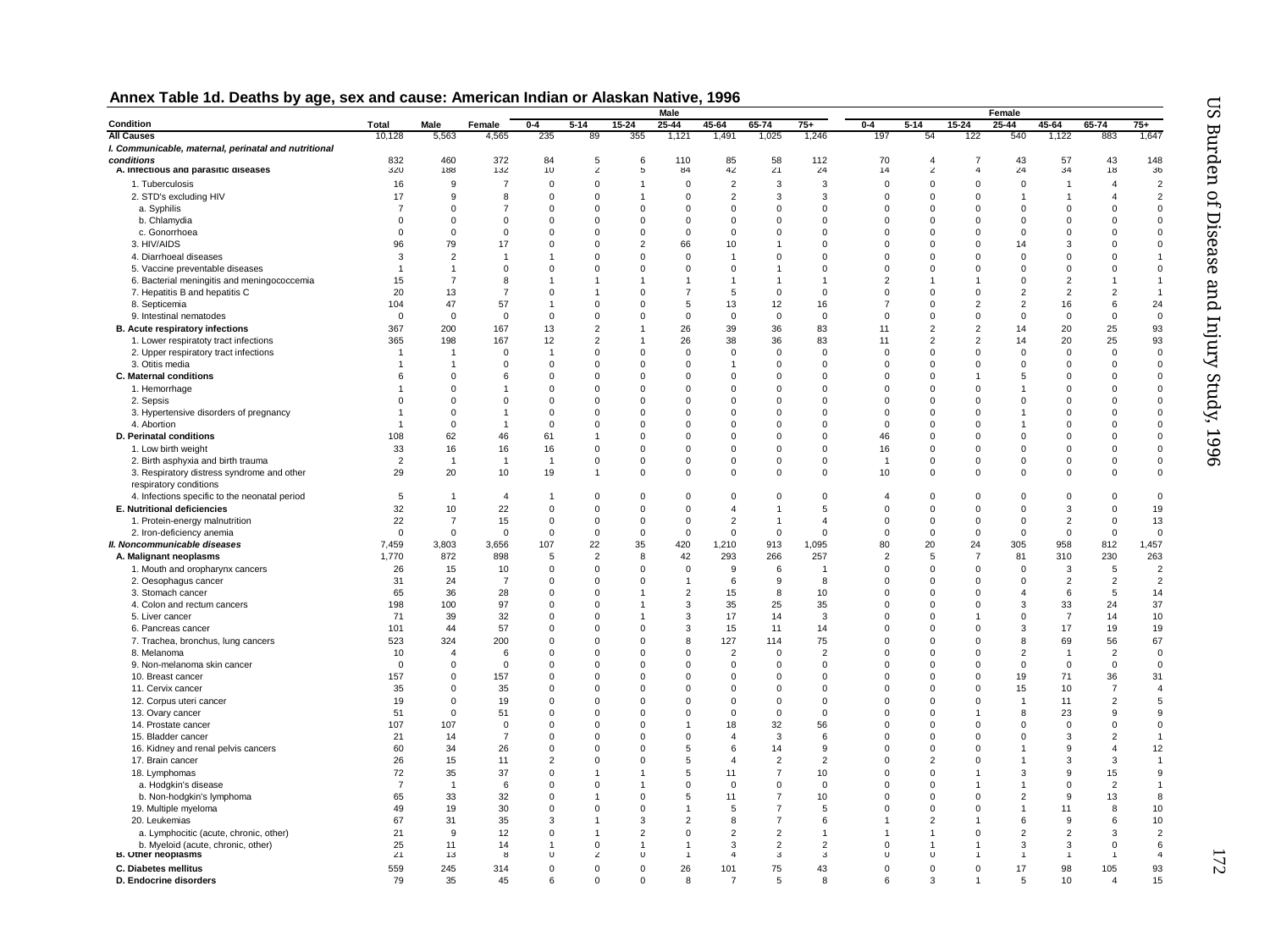

US Burden of Disease and Injury Study, 1996
172

## Slide 11
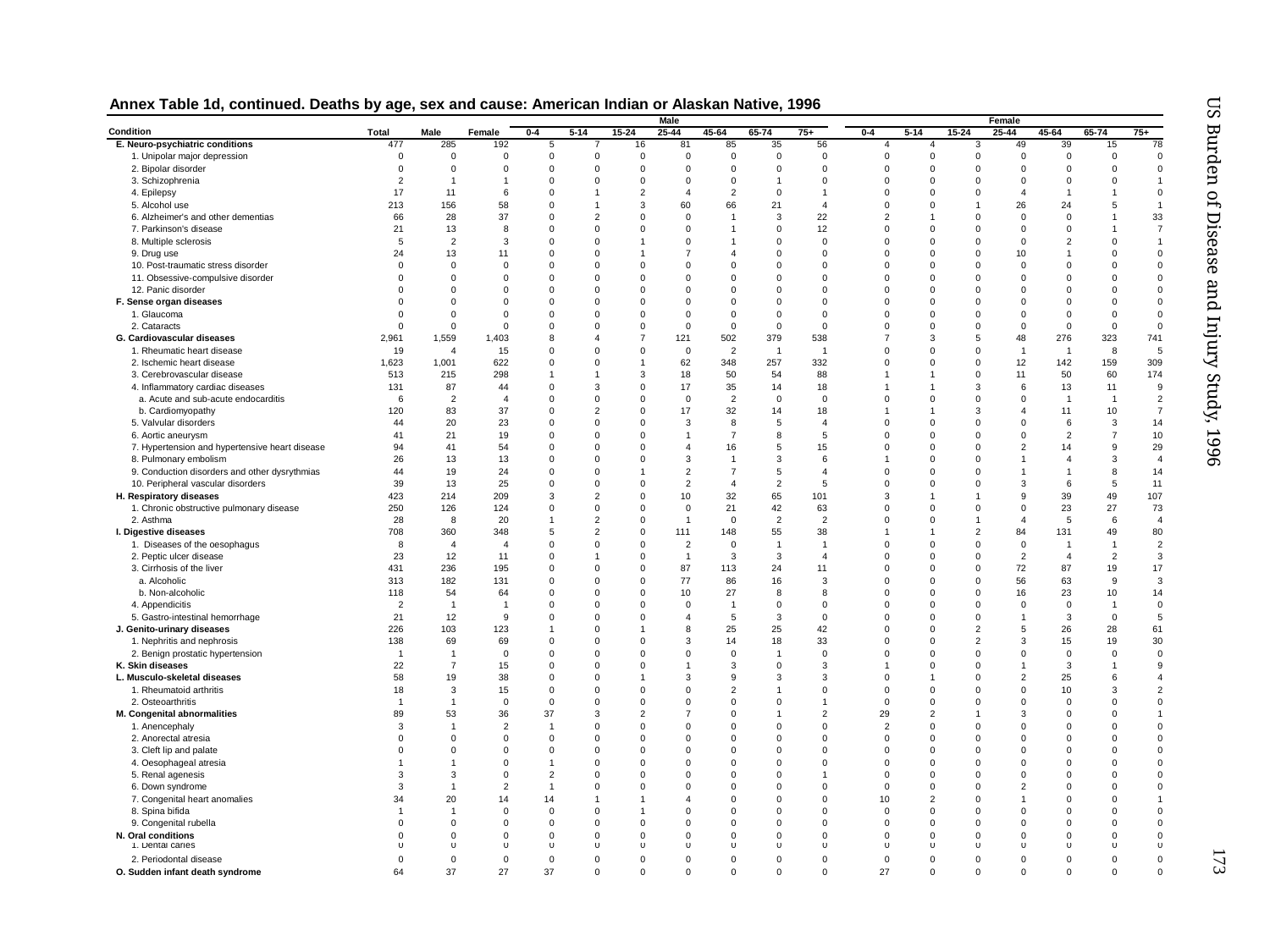

US Burden of Disease and Injury Study, 1996
173

## Slide 12
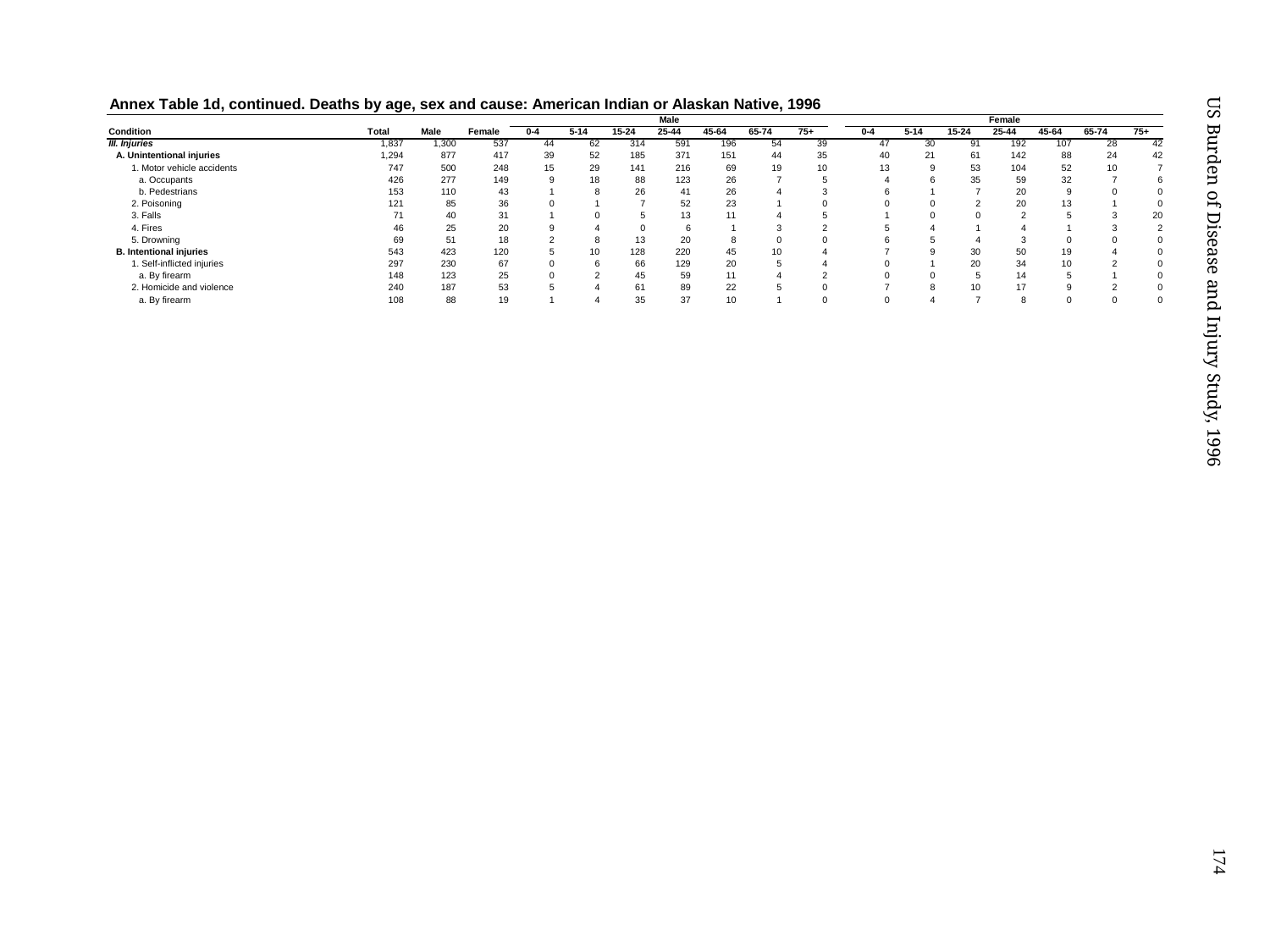

US Burden of Disease and Injury Study, 1996
174

## Slide 13
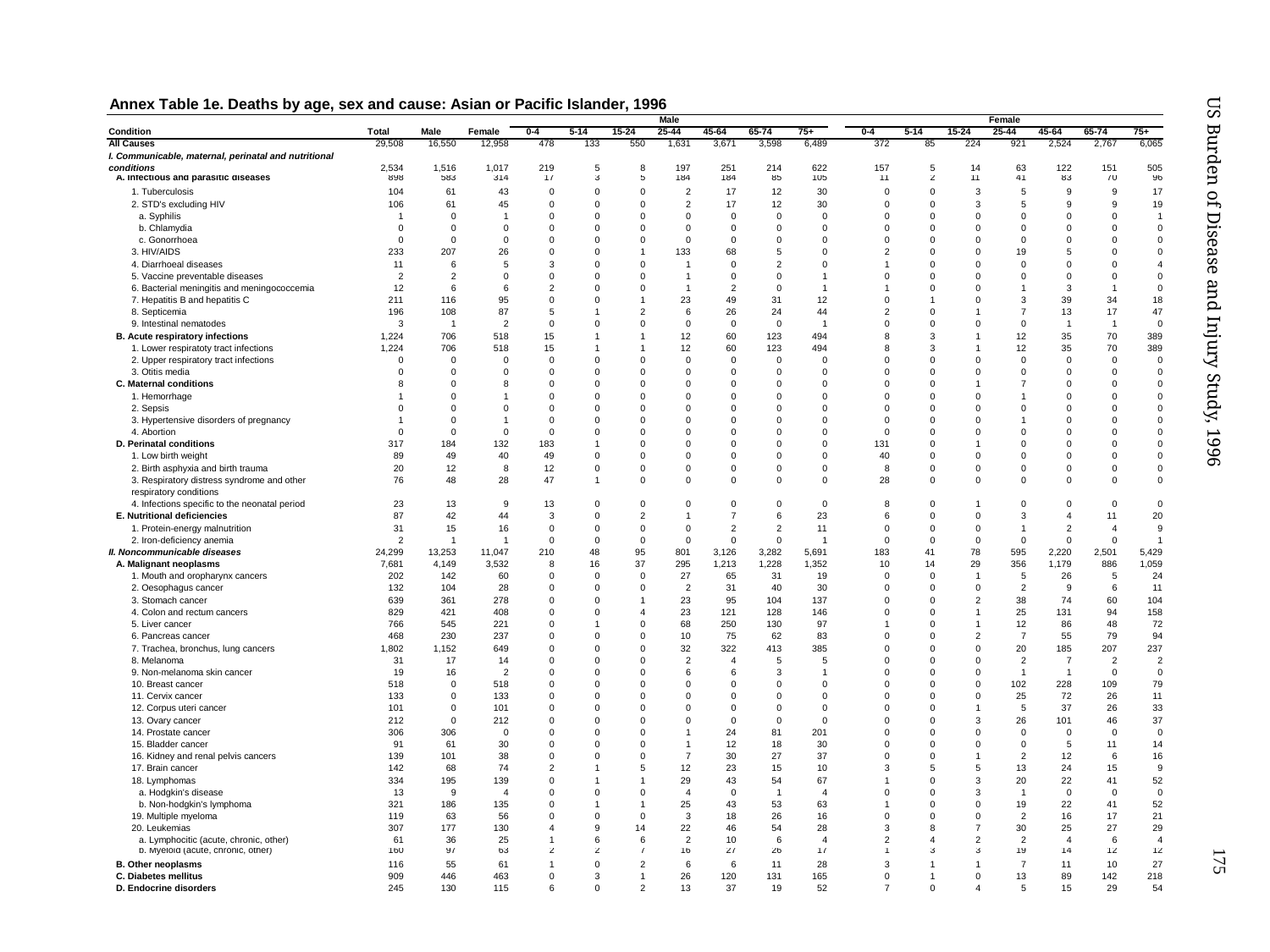

US Burden of Disease and Injury Study, 1996
175

## Slide 14
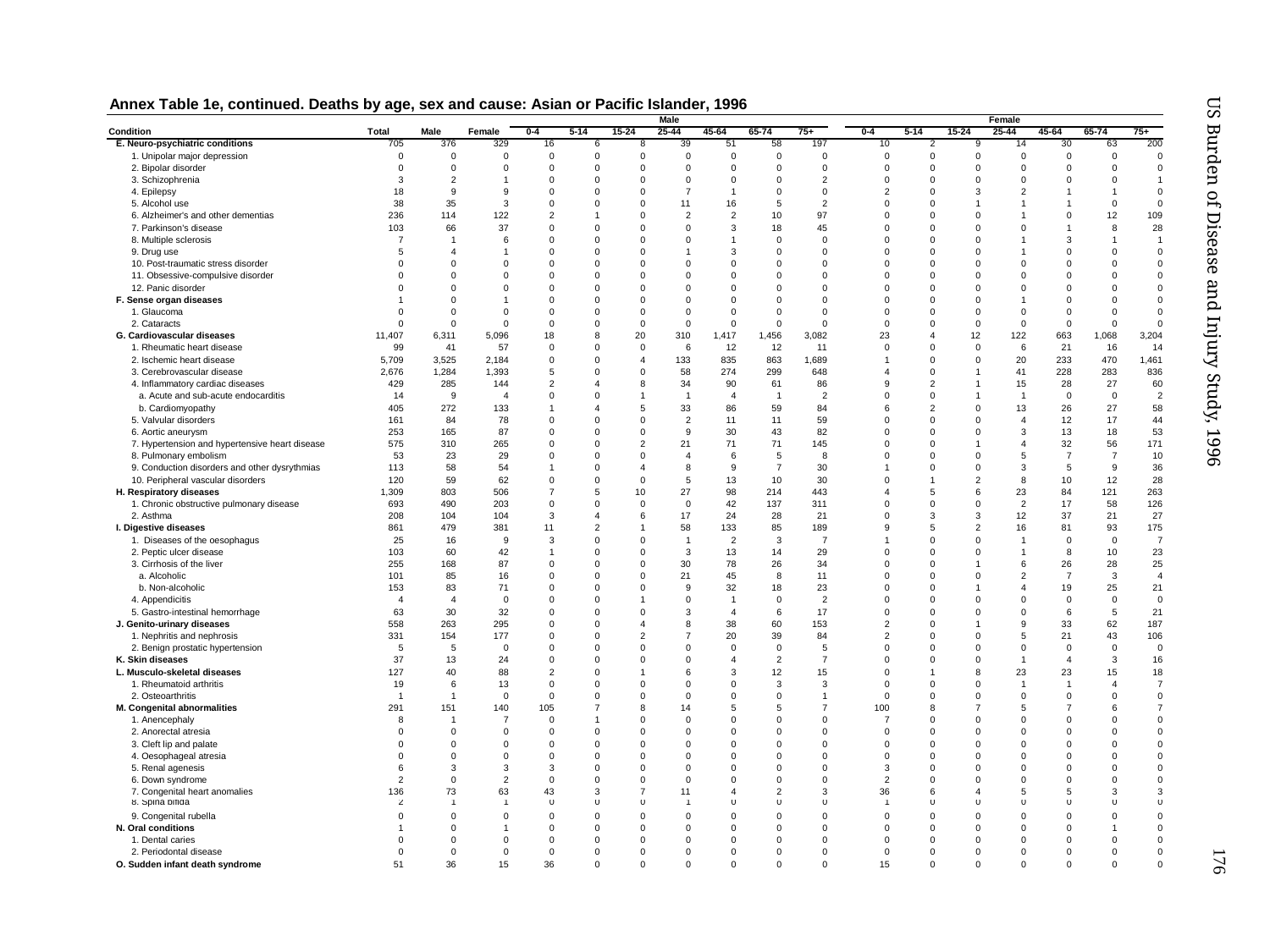

US Burden of Disease and Injury Study, 1996
176

## Slide 15
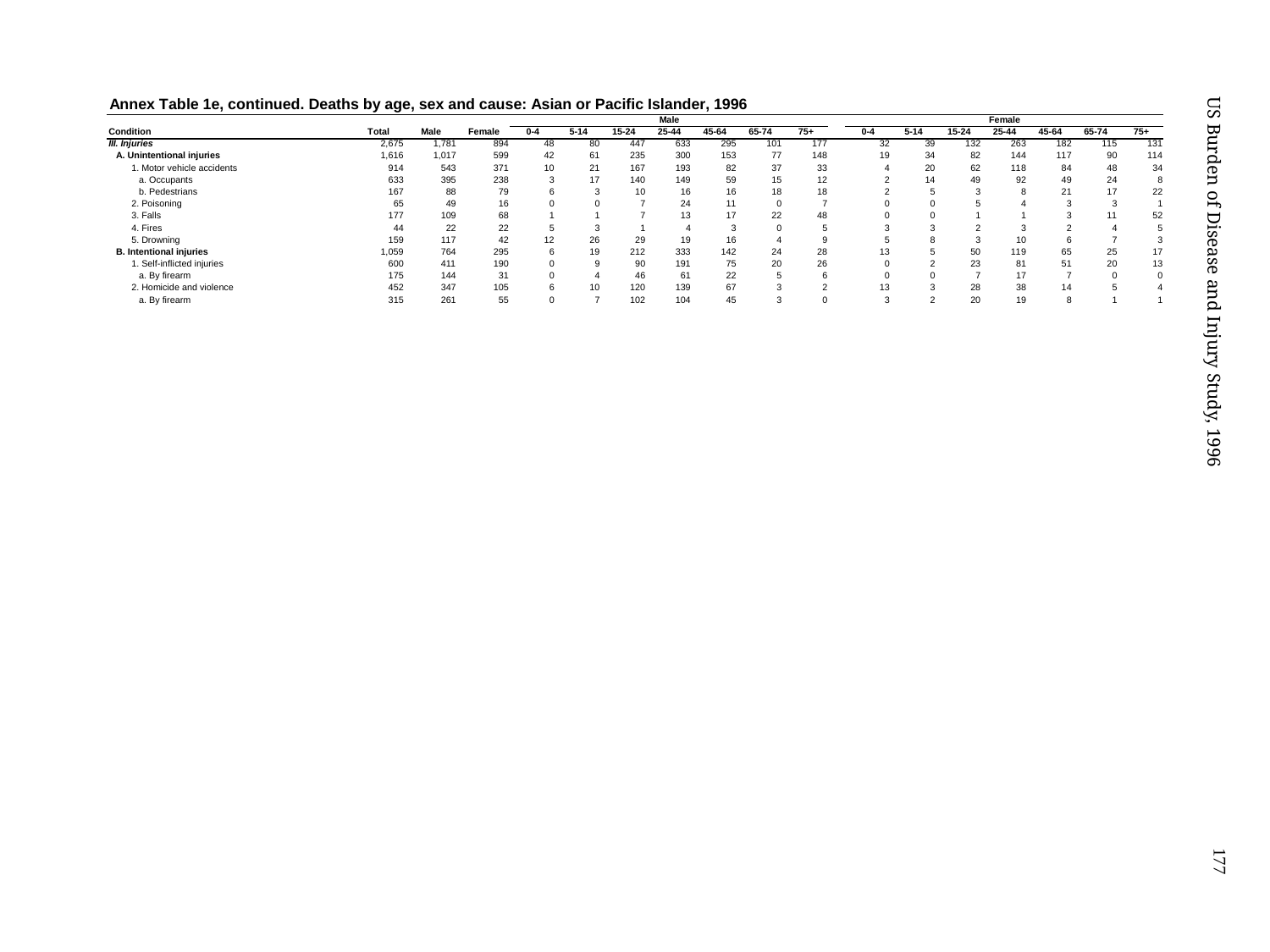

US Burden of Disease and Injury Study, 1996
177

## Slide 16
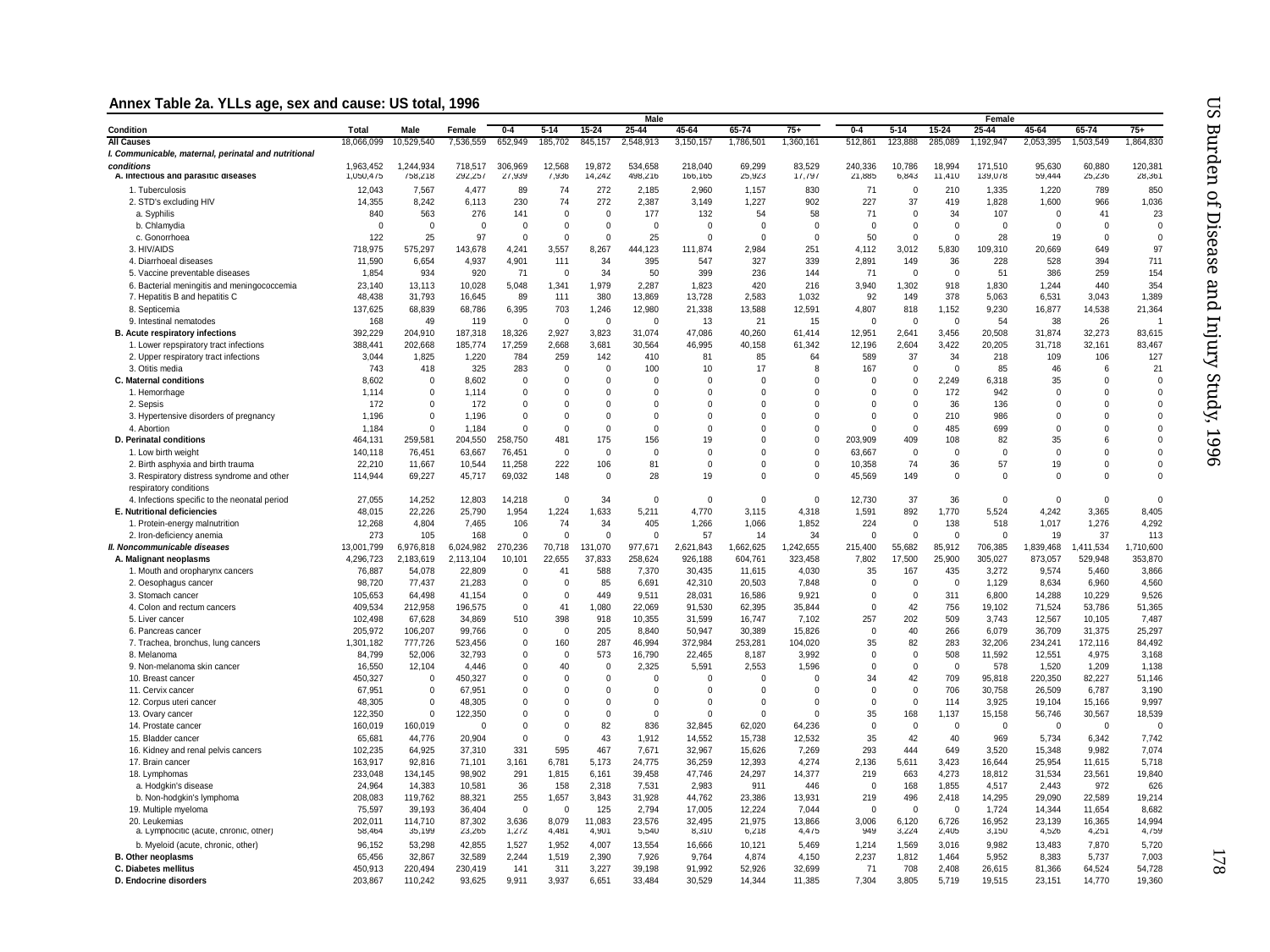

US Burden of Disease and Injury Study, 1996
178

## Slide 17
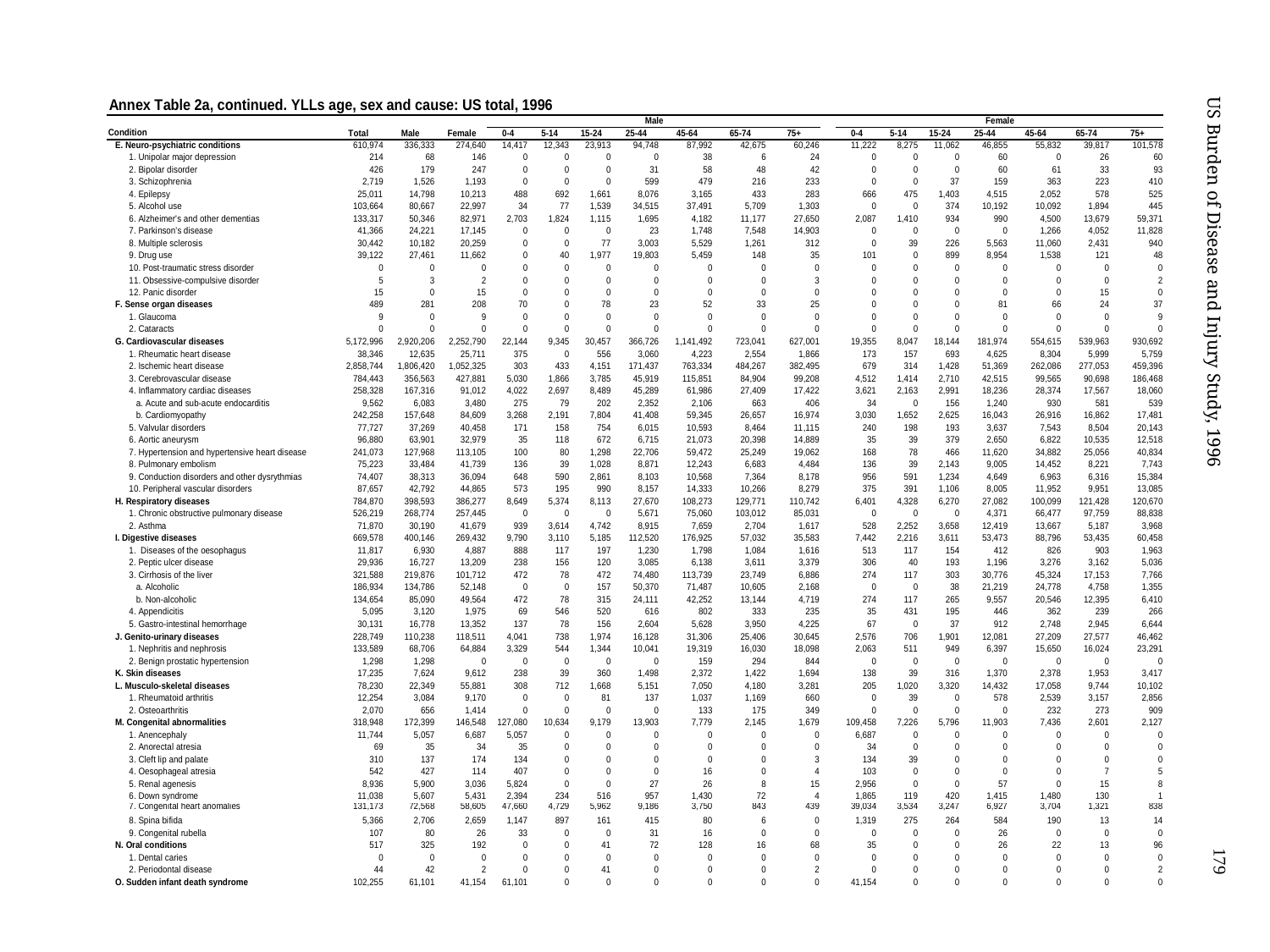

US Burden of Disease and Injury Study, 1996
179

## Slide 18
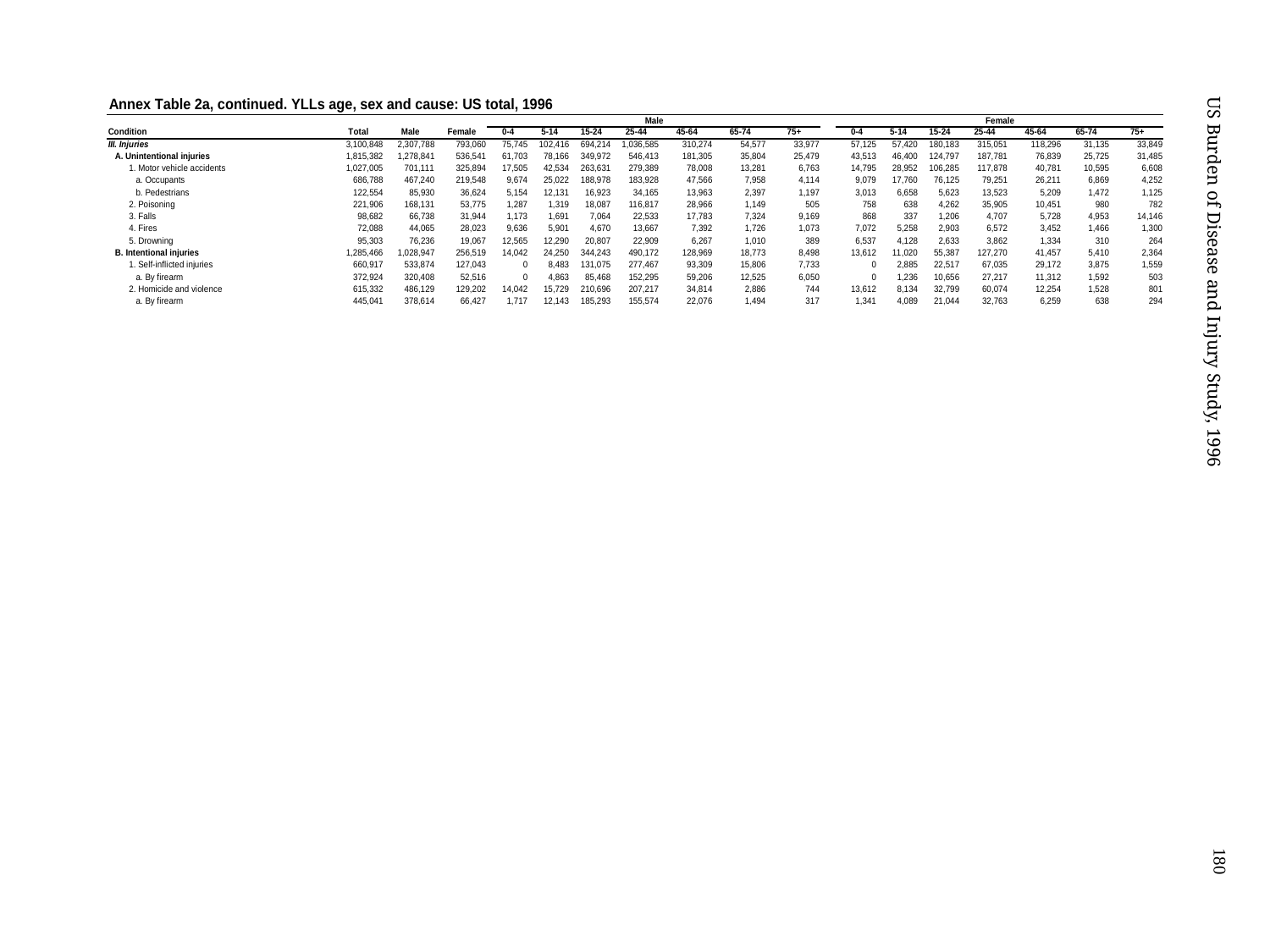

US Burden of Disease and Injury Study, 1996
180

## Slide 19
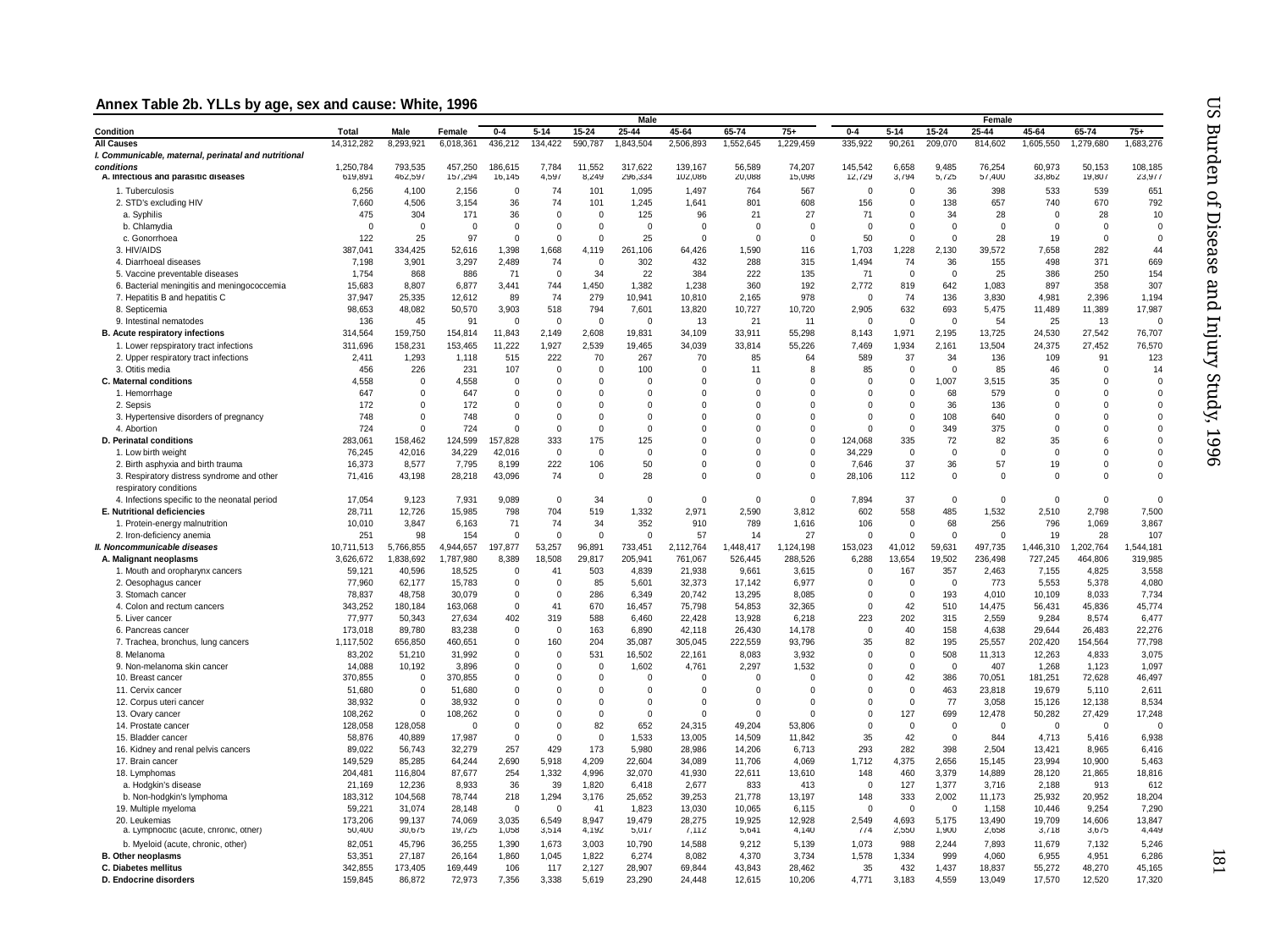

US Burden of Disease and Injury Study, 1996
181

## Slide 20
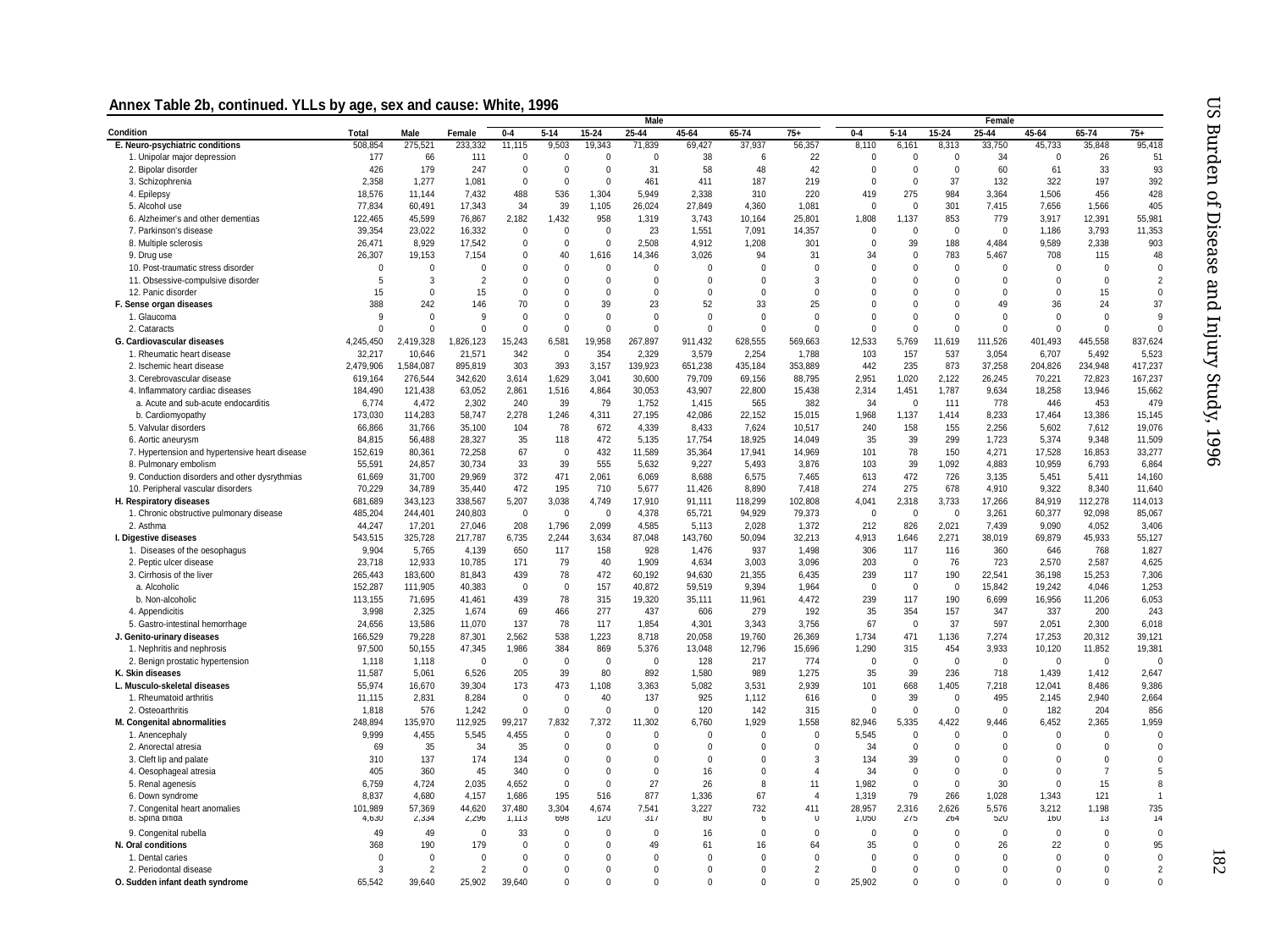

US Burden of Disease and Injury Study, 1996
182

## Slide 21
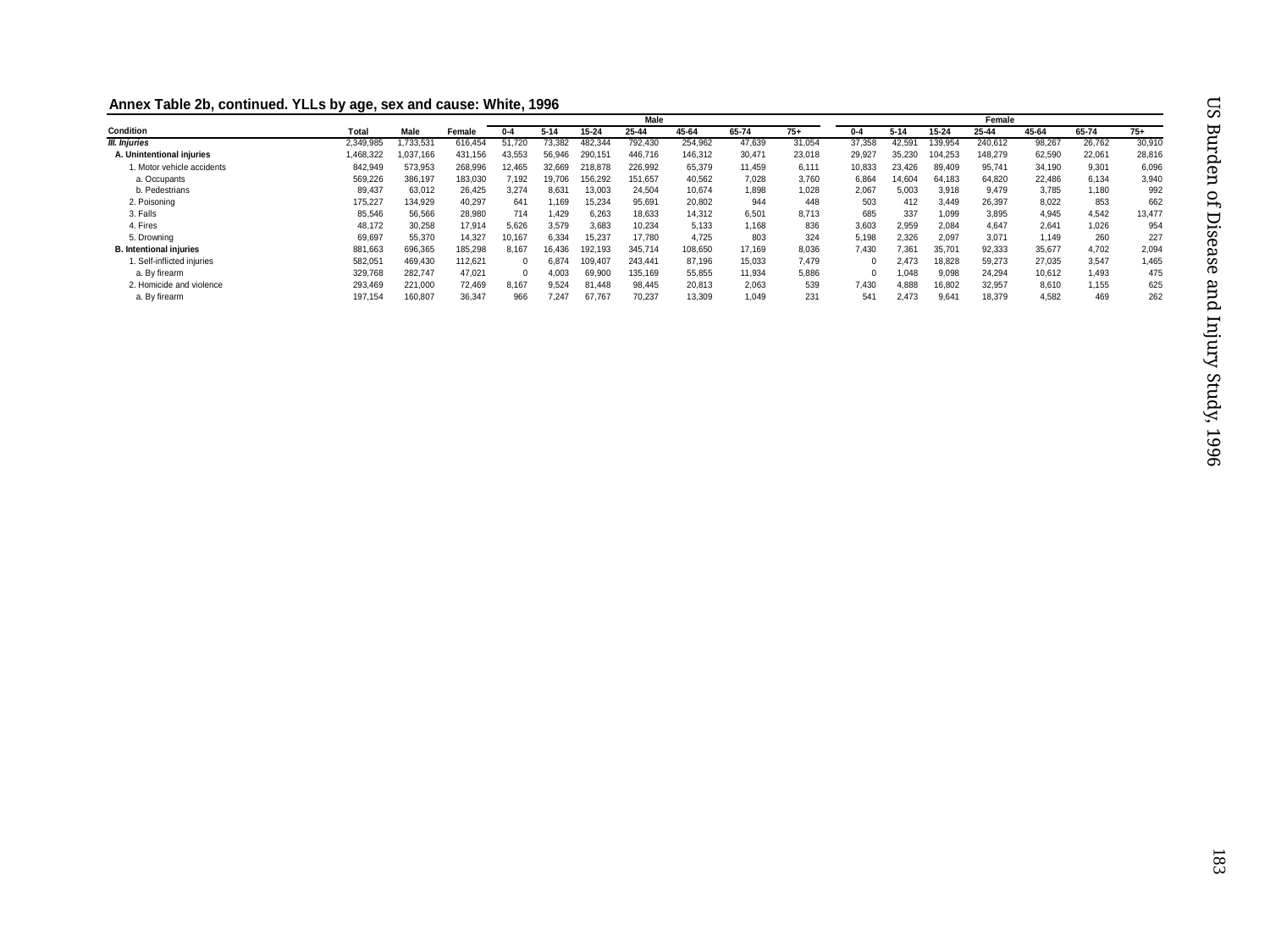

US Burden of Disease and Injury Study, 1996
183

## Slide 22
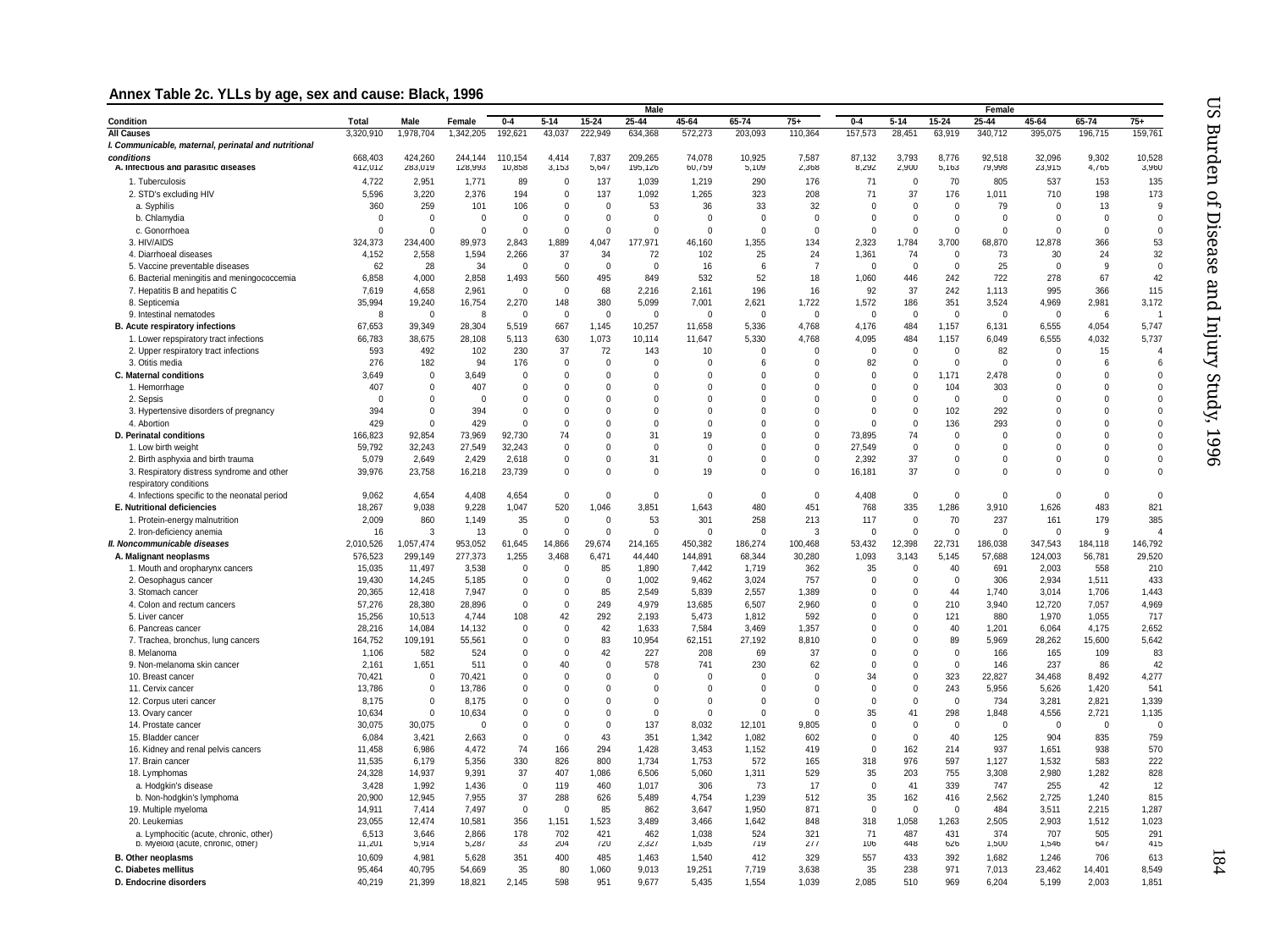

US Burden of Disease and Injury Study, 1996
184

## Slide 23
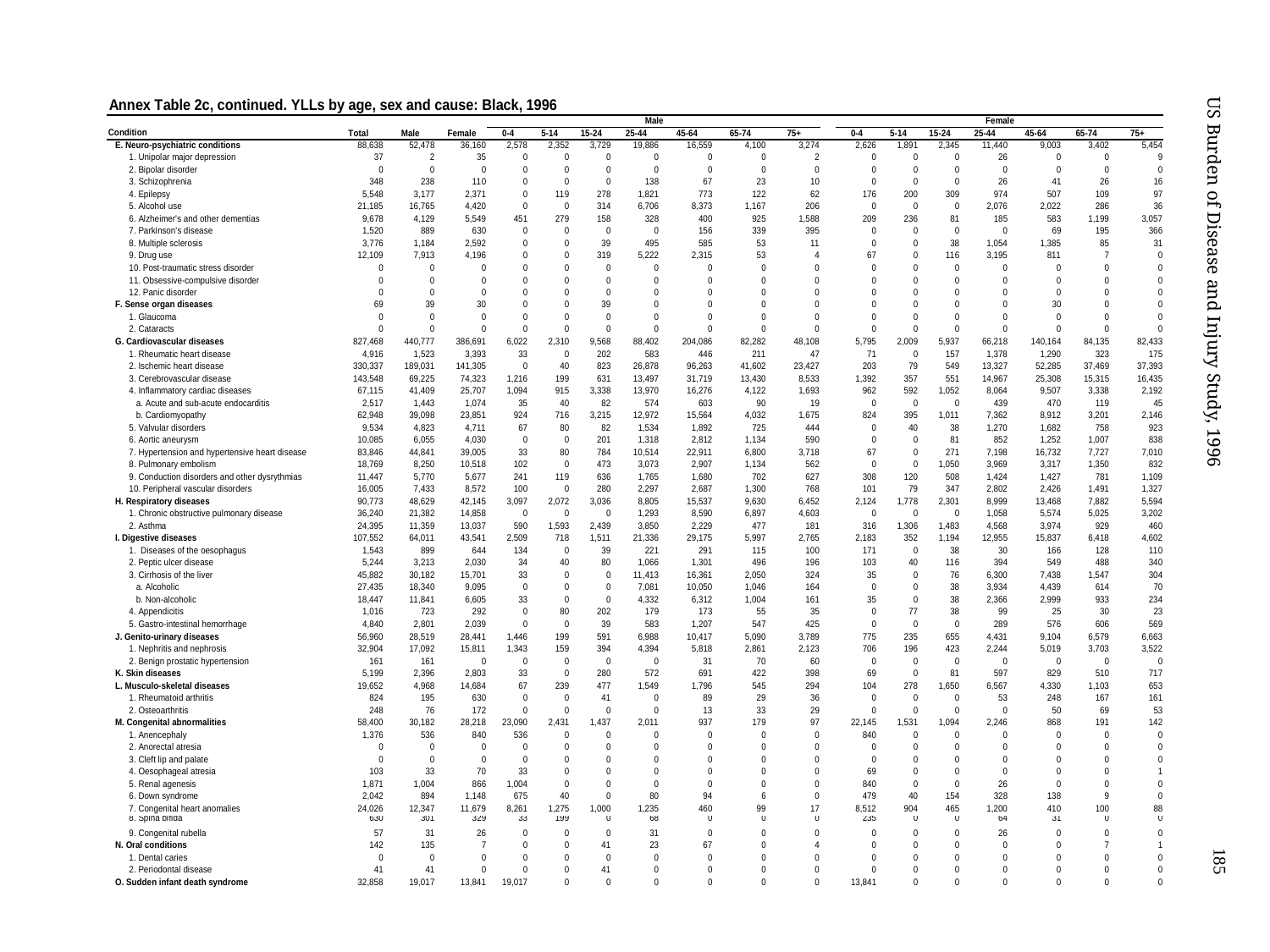

US Burden of Disease and Injury Study, 1996
185

## Slide 24
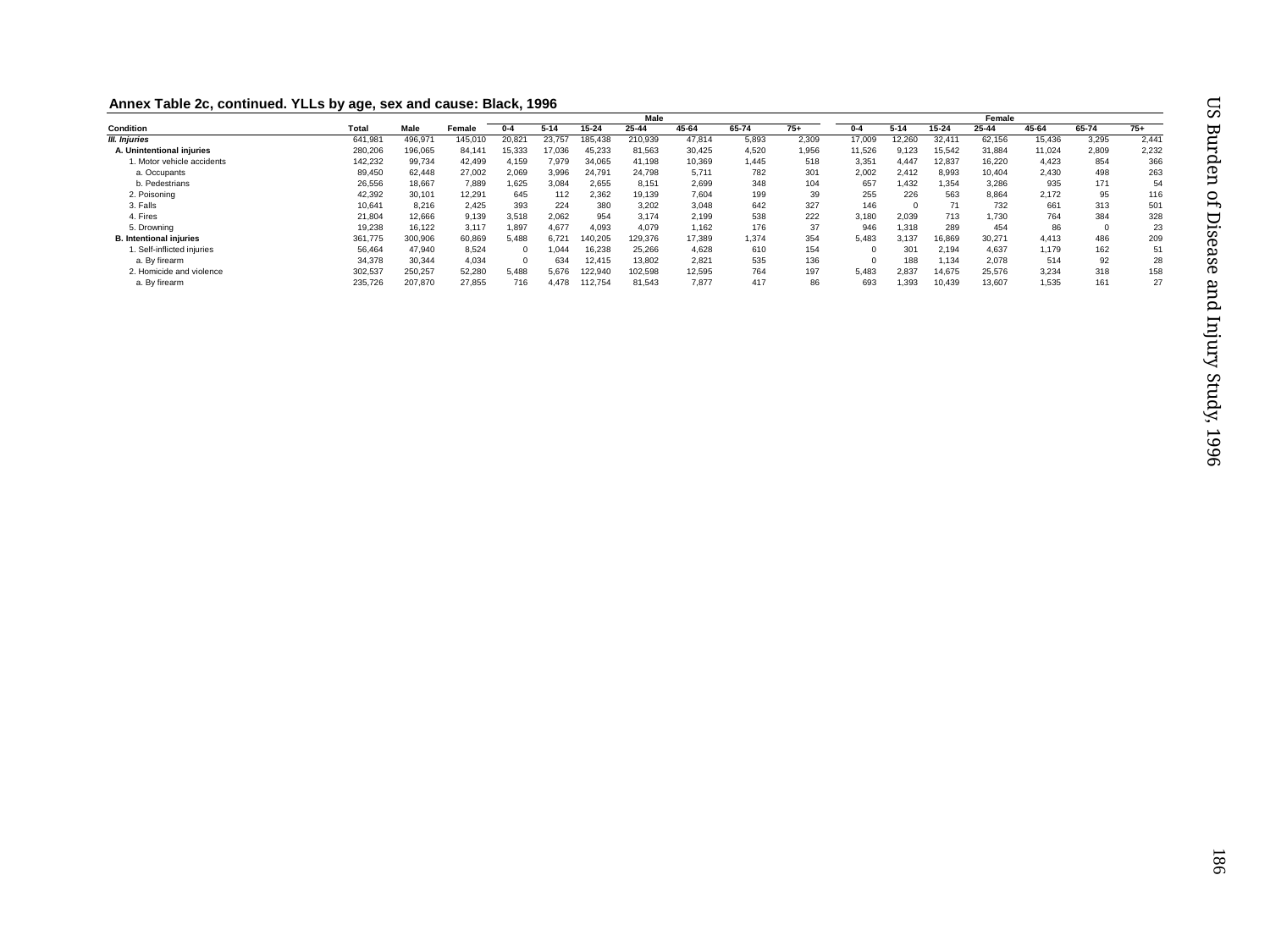

US Burden of Disease and Injury Study, 1996
186

## Slide 25
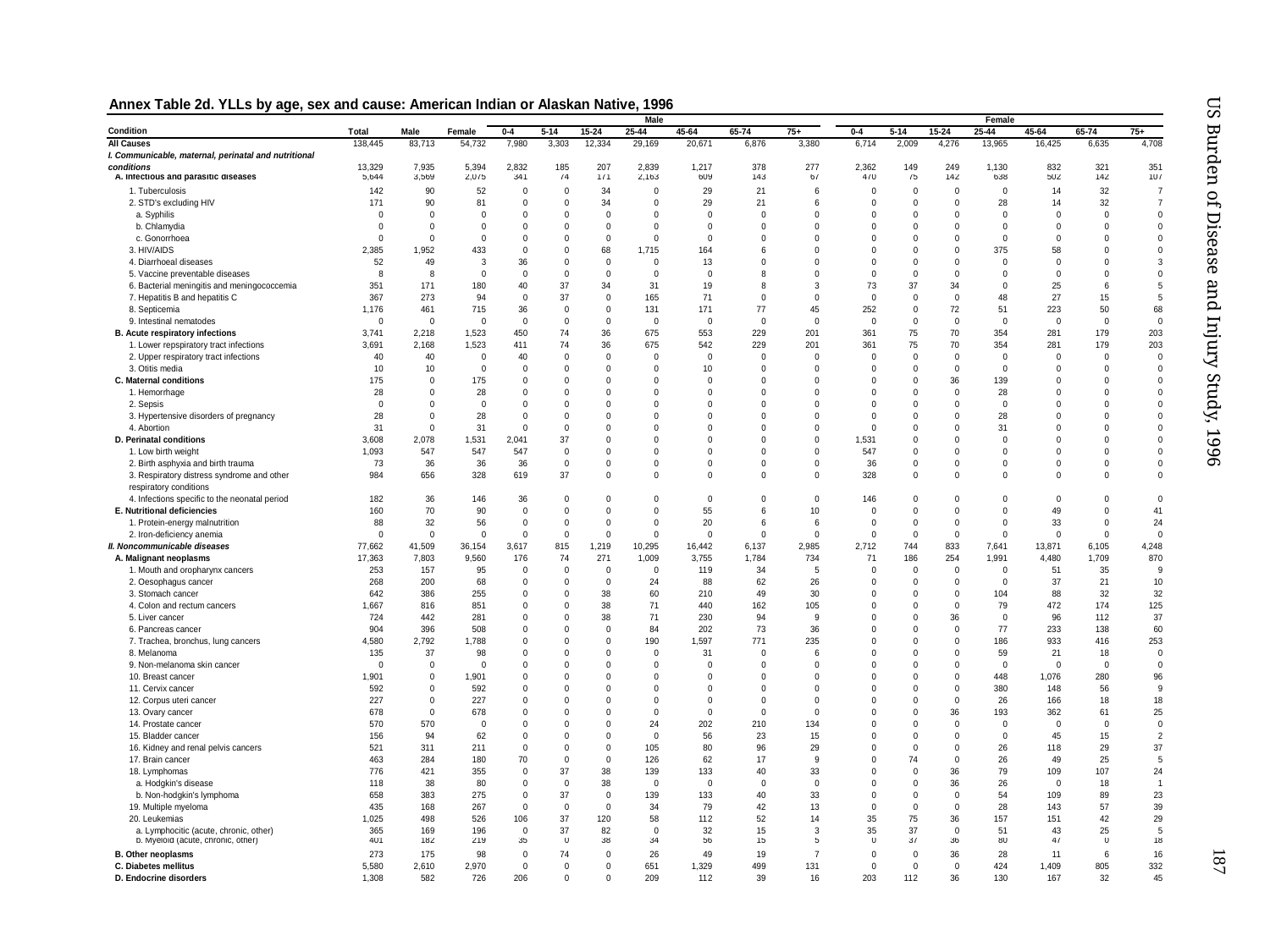

US Burden of Disease and Injury Study, 1996
187

## Slide 26
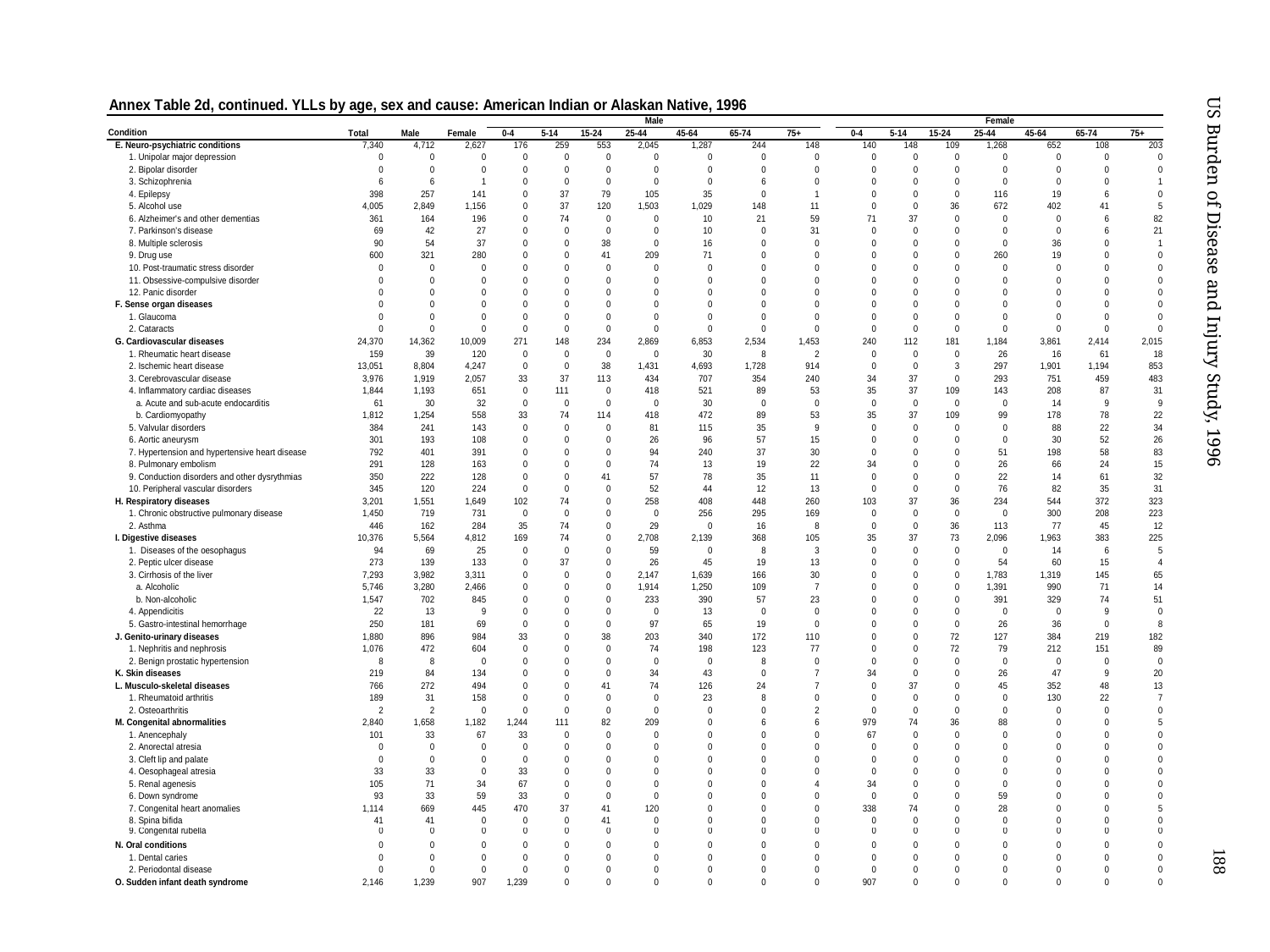

US Burden of Disease and Injury Study, 1996
188

## Slide 27
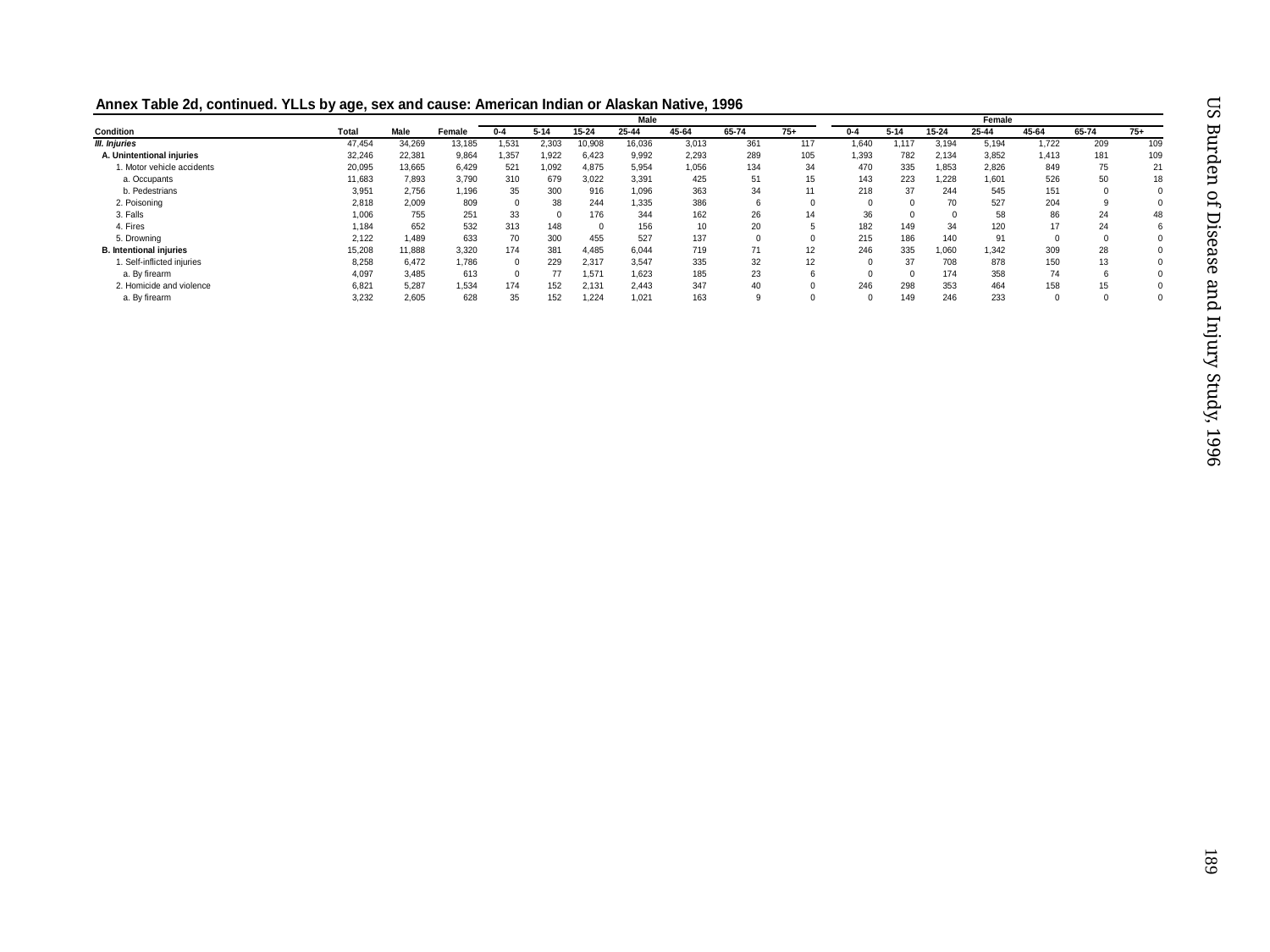

US Burden of Disease and Injury Study, 1996
189

## Slide 28
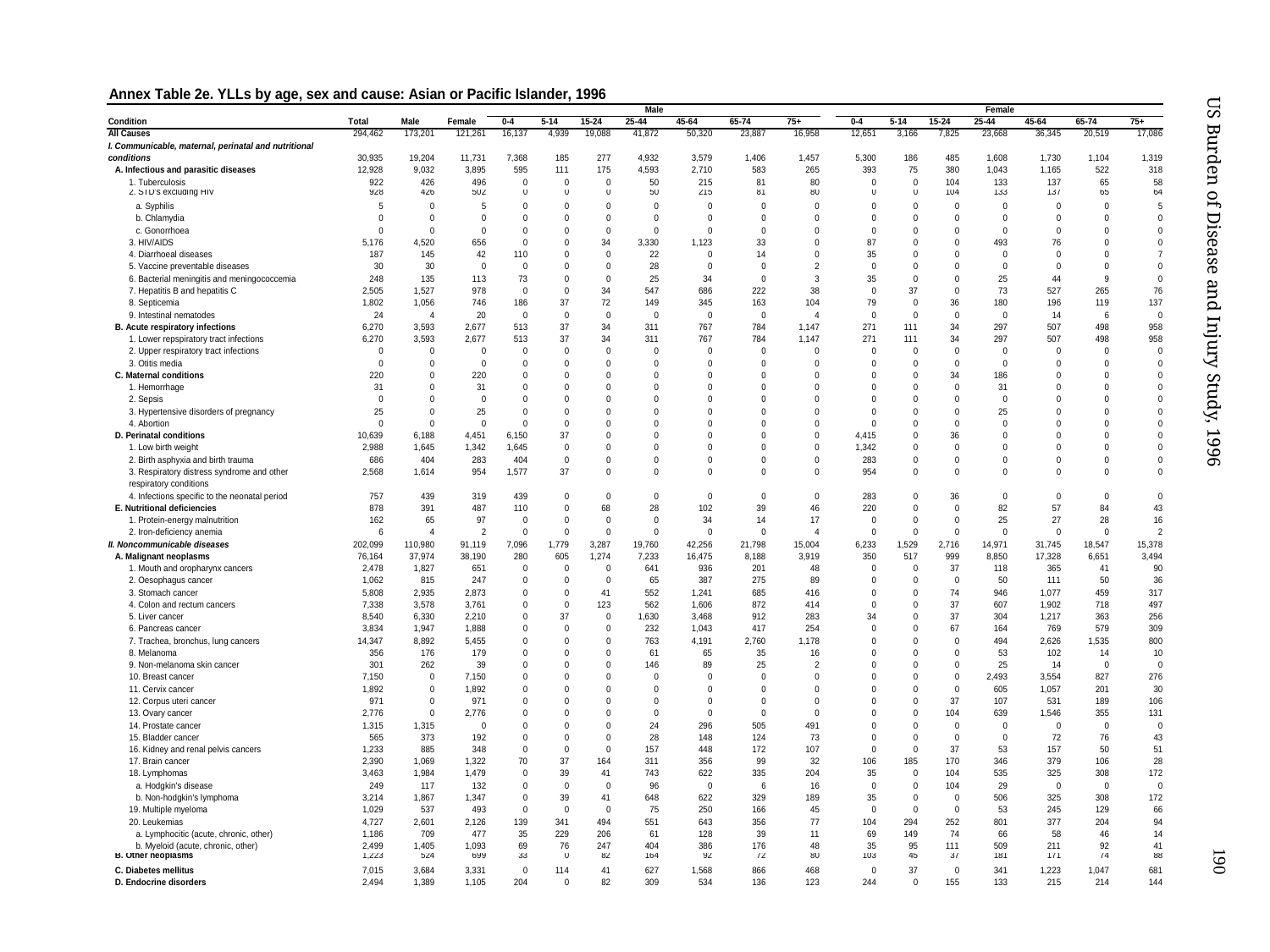

US Burden of Disease and Injury Study, 1996
190

## Slide 29
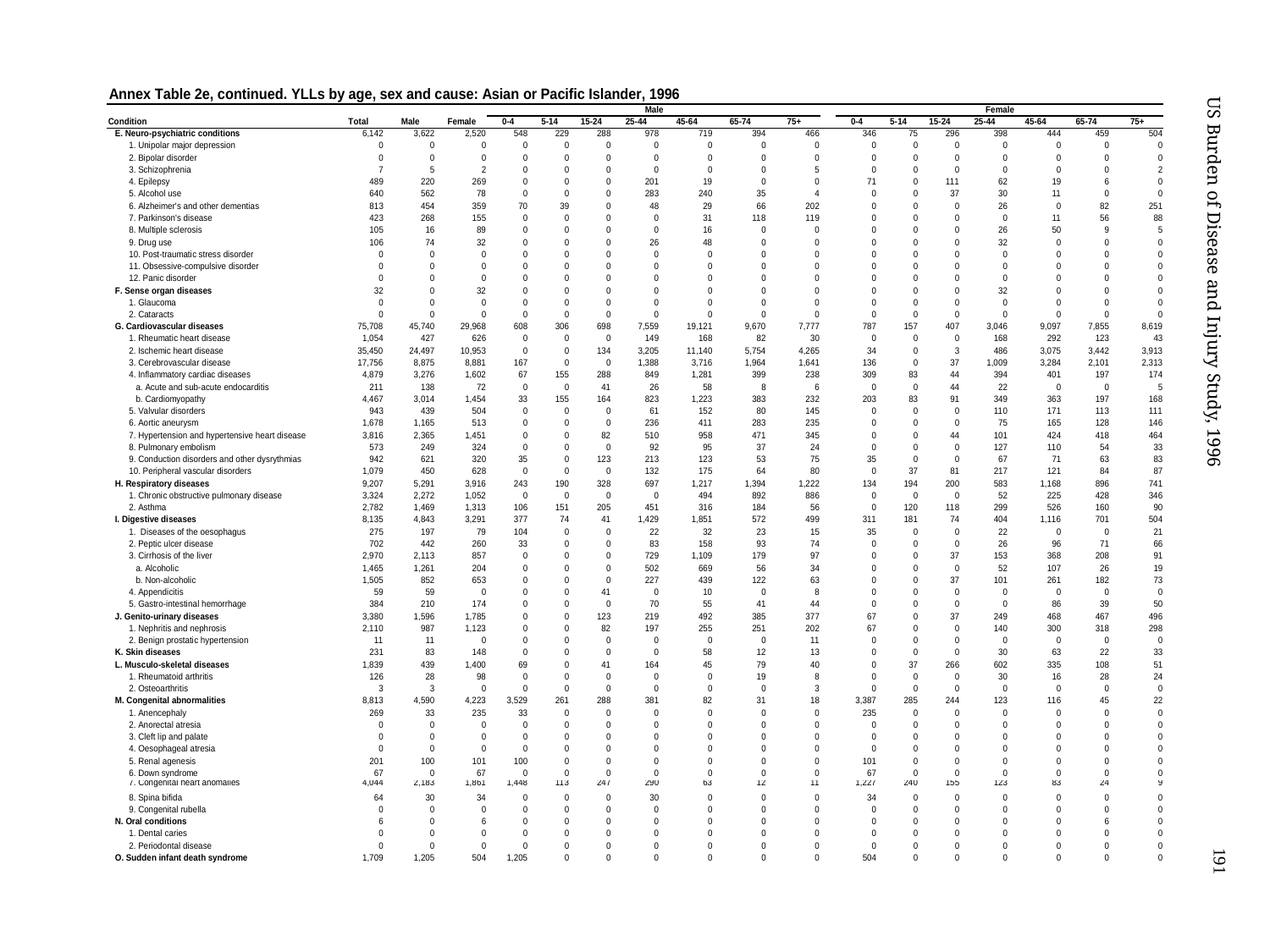

US Burden of Disease and Injury Study, 1996
191

## Slide 30
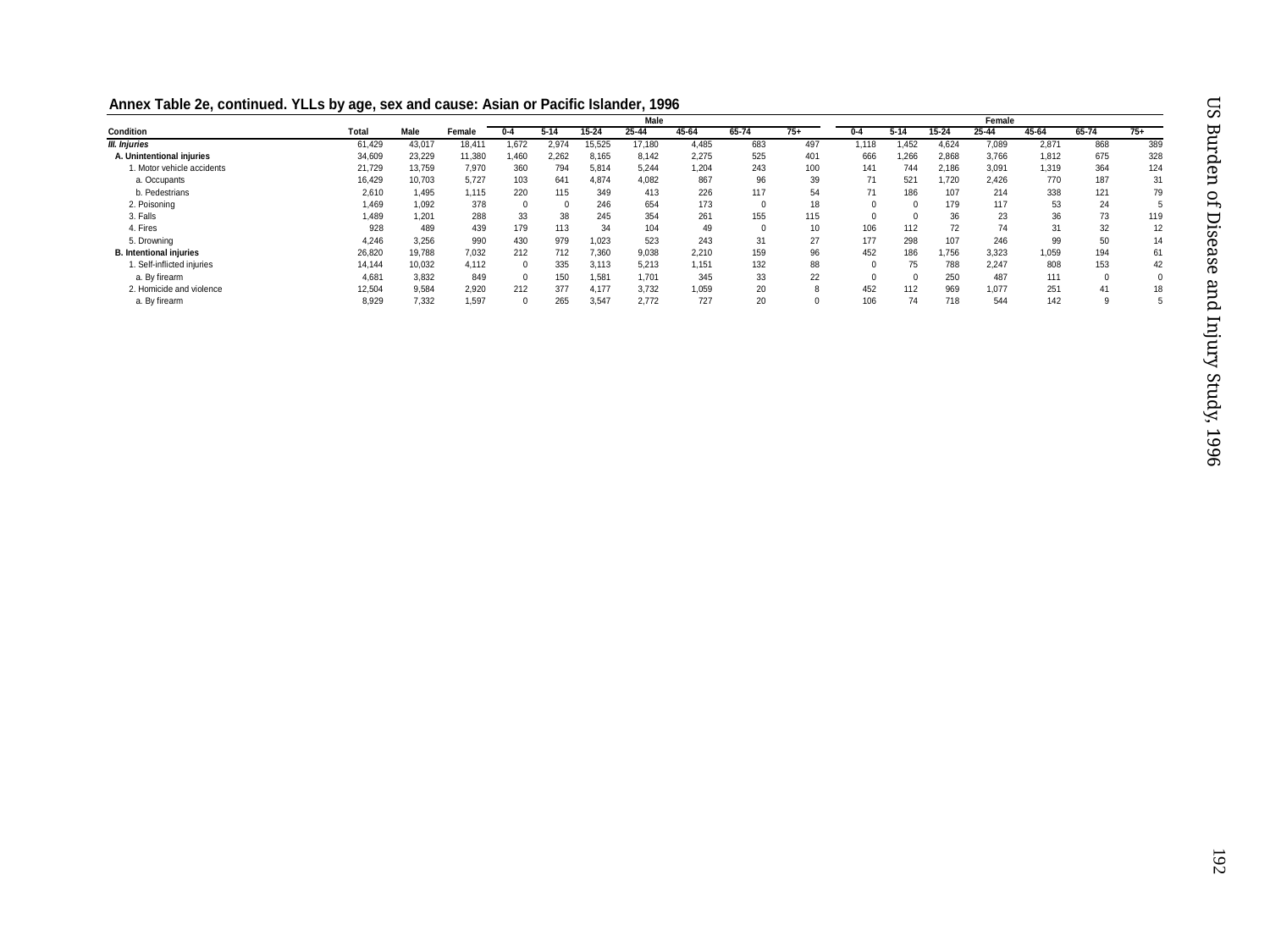

US Burden of Disease and Injury Study, 1996
192

## Slide 31
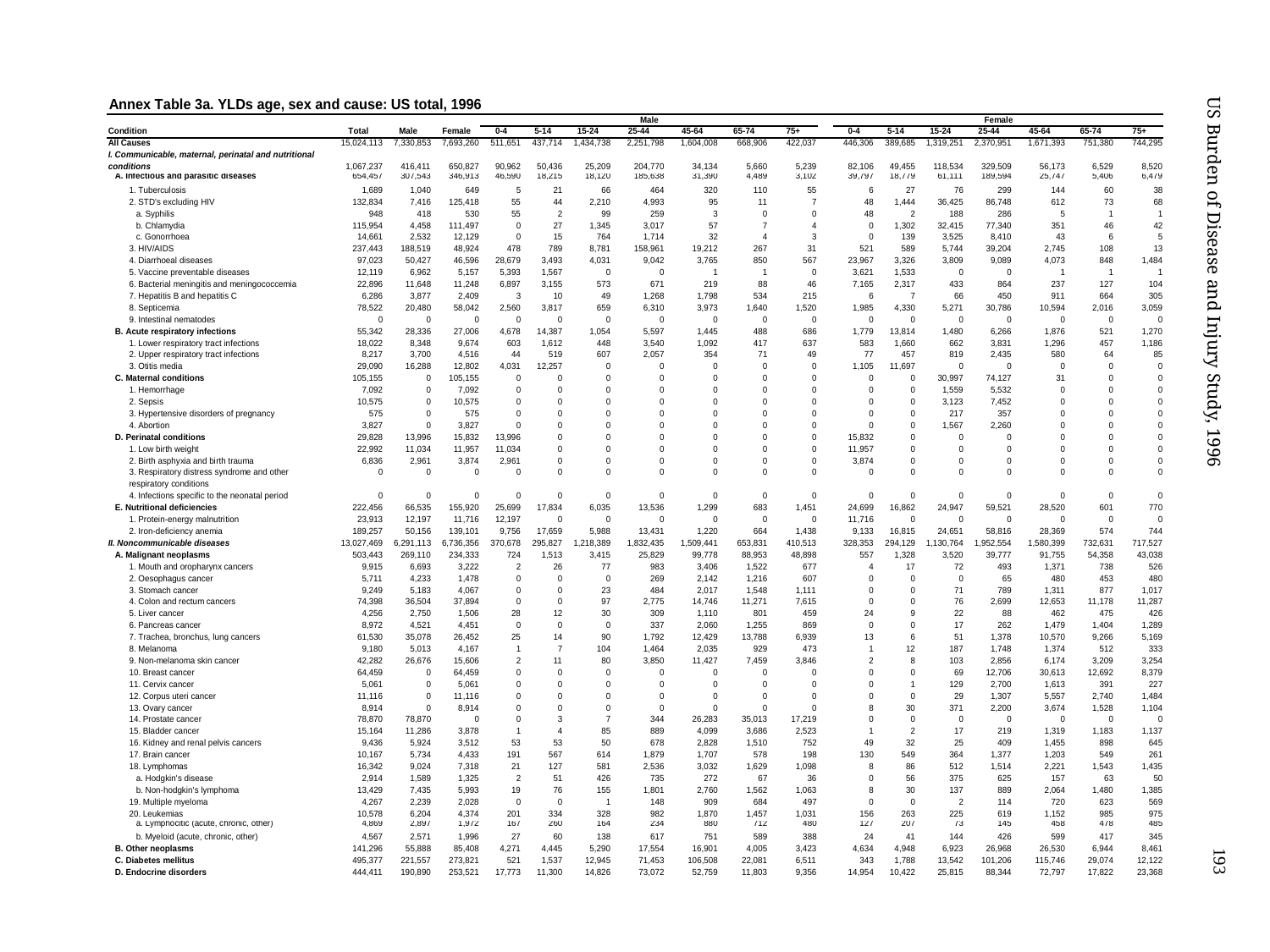

US Burden of Disease and Injury Study, 1996
193

## Slide 32
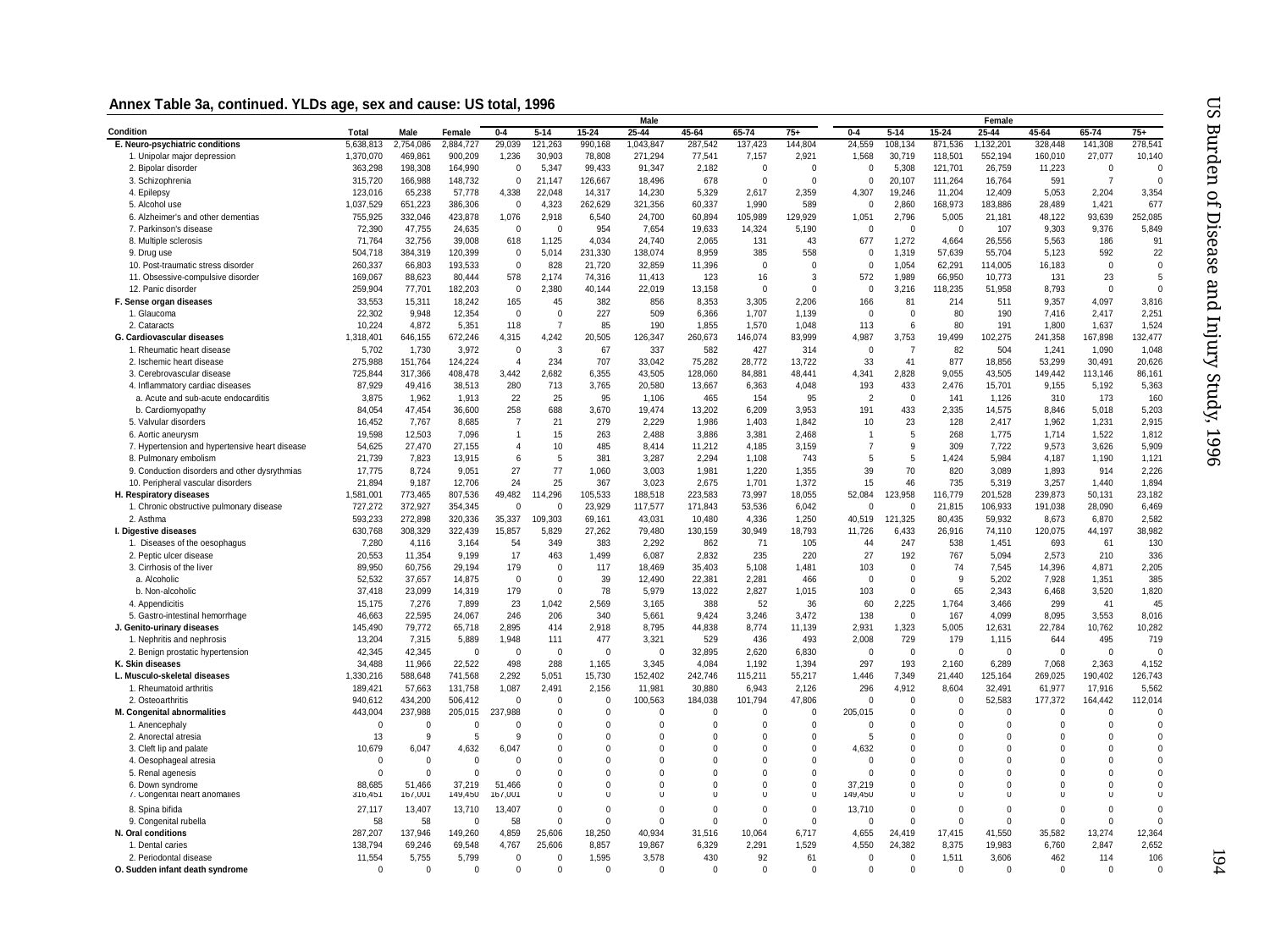

US Burden of Disease and Injury Study, 1996
194

## Slide 33
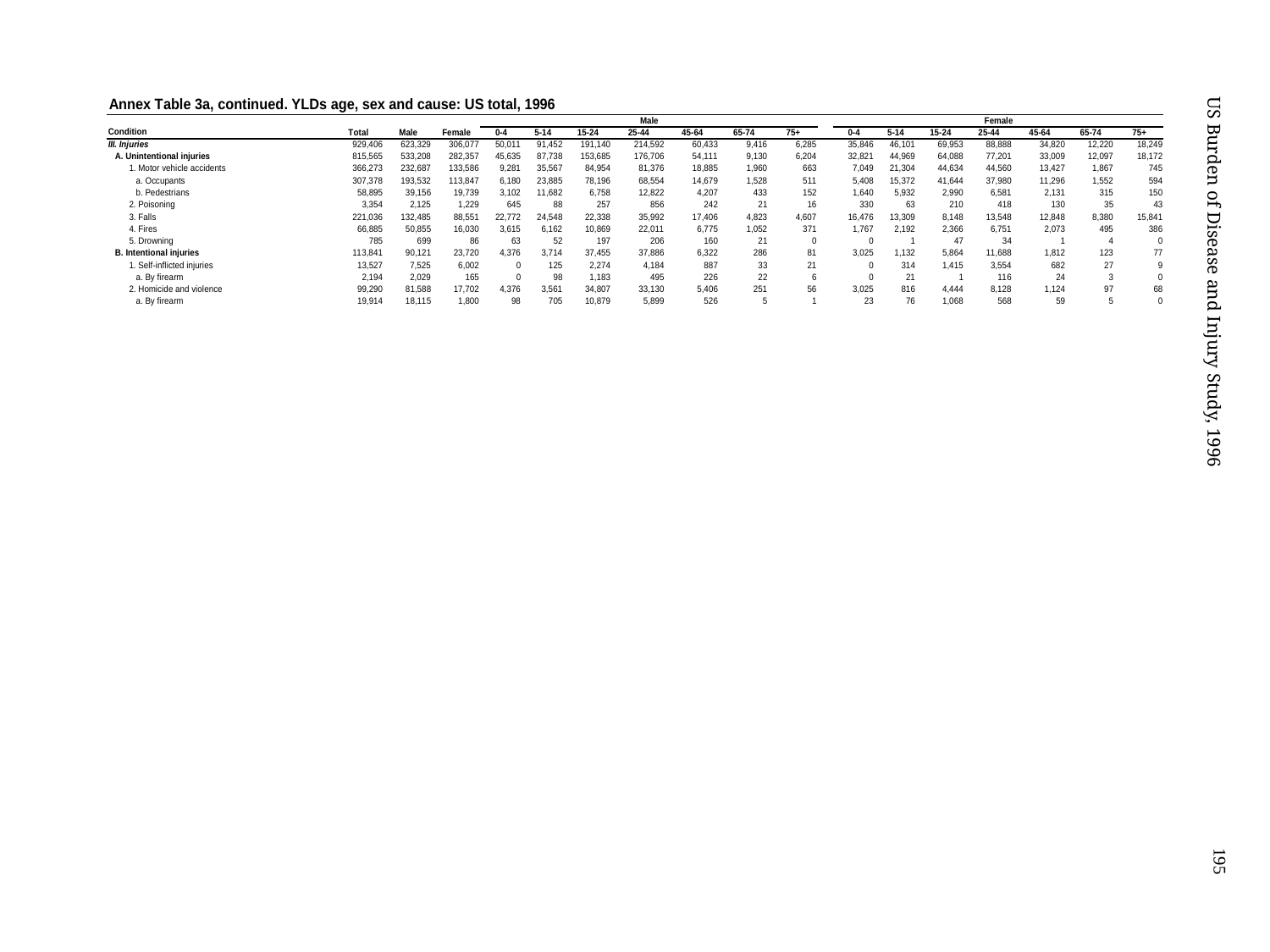

US Burden of Disease and Injury Study, 1996
195

## Slide 34
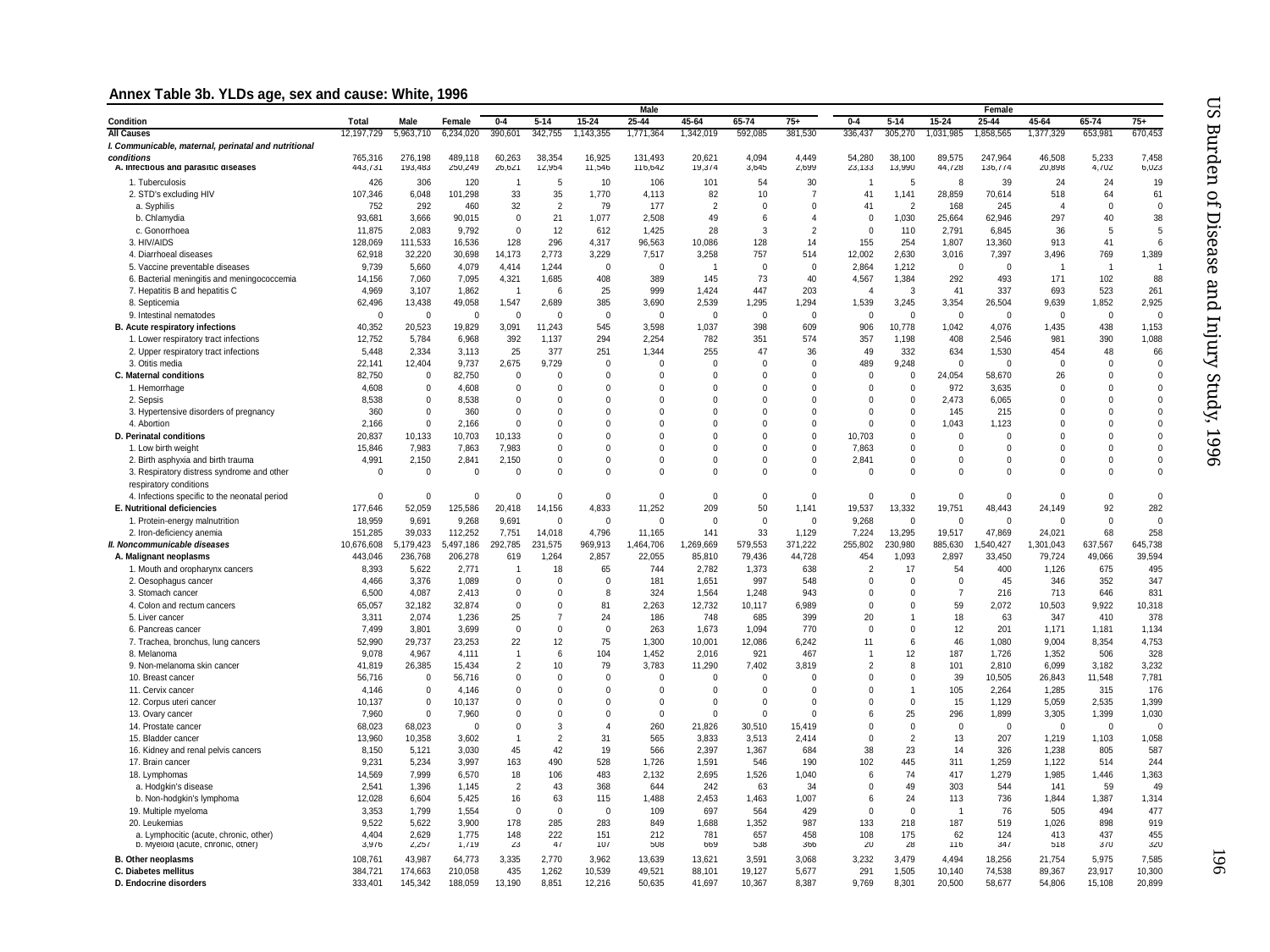

US Burden of Disease and Injury Study, 1996
196

## Slide 35
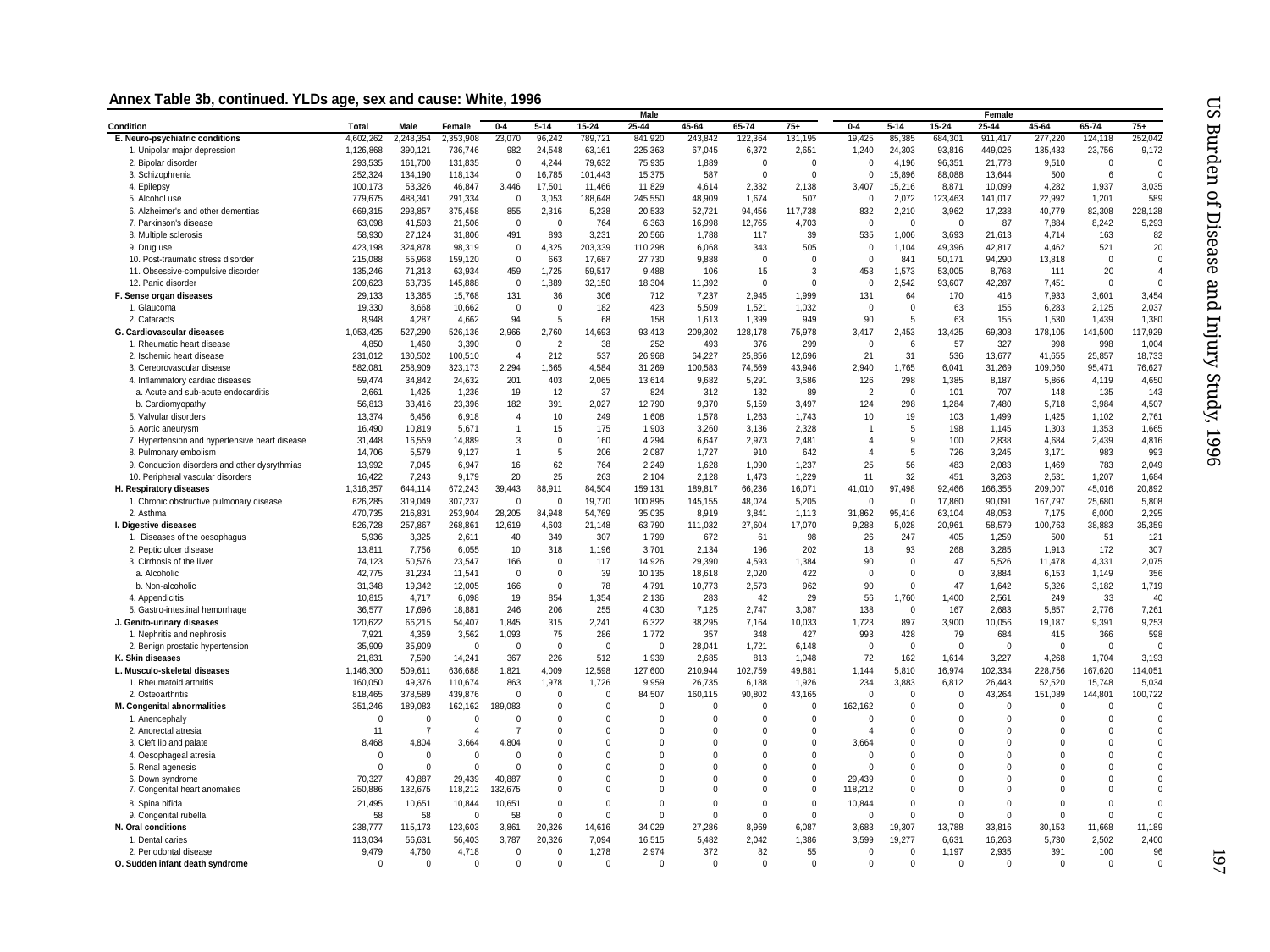

US Burden of Disease and Injury Study, 1996
197

## Slide 36
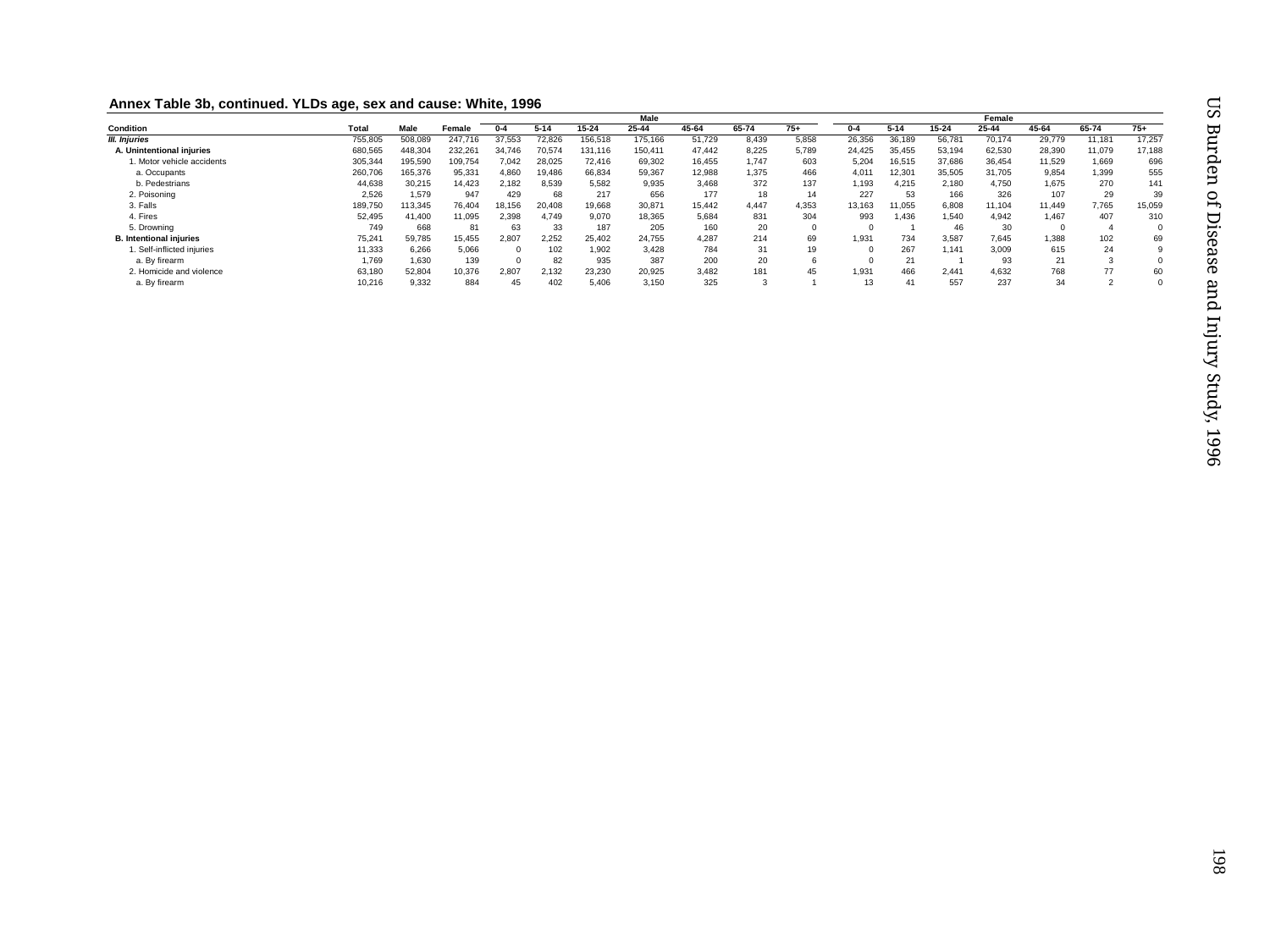

US Burden of Disease and Injury Study, 1996
198

## Slide 37
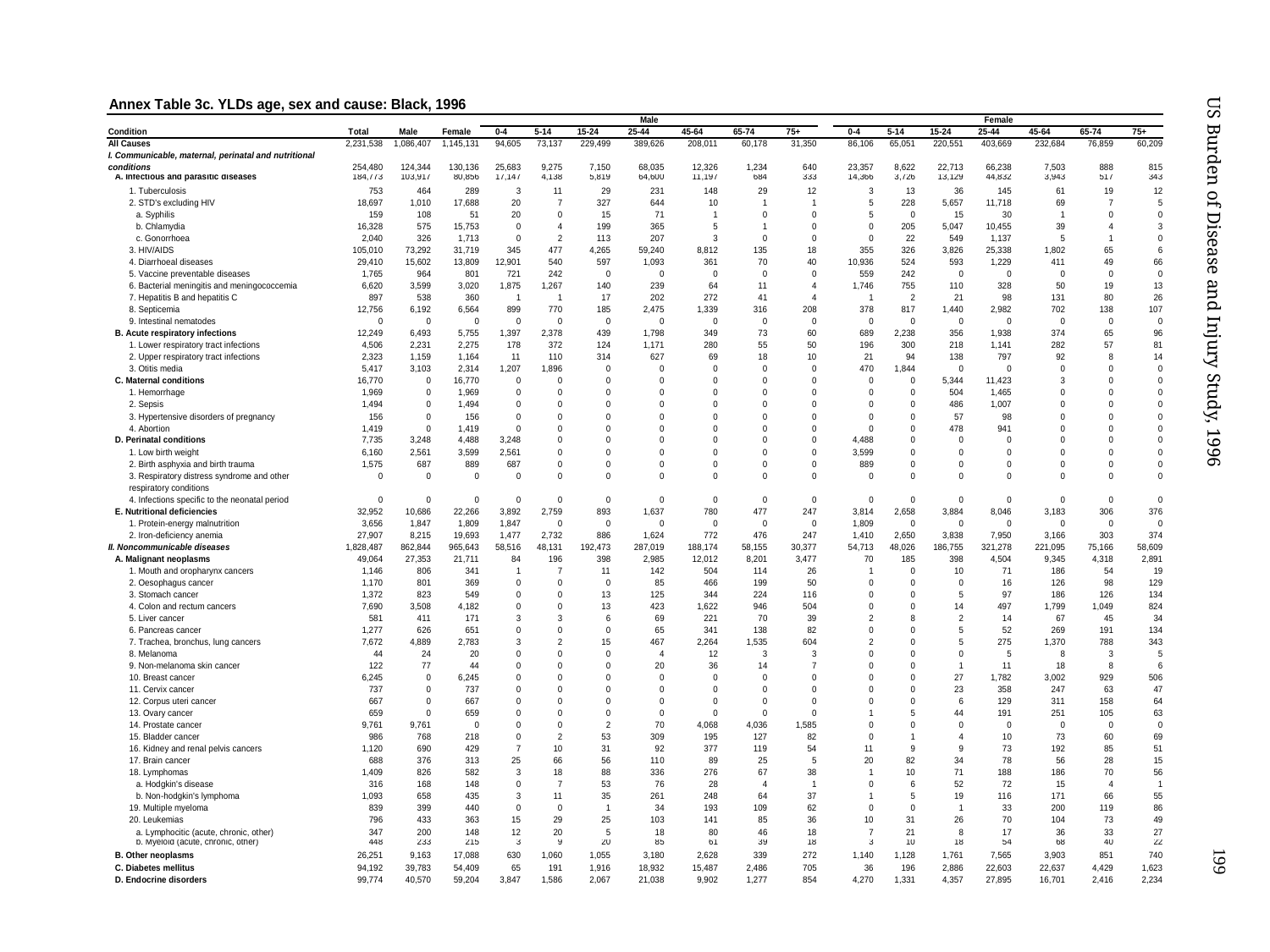

US Burden of Disease and Injury Study, 1996
199

## Slide 38
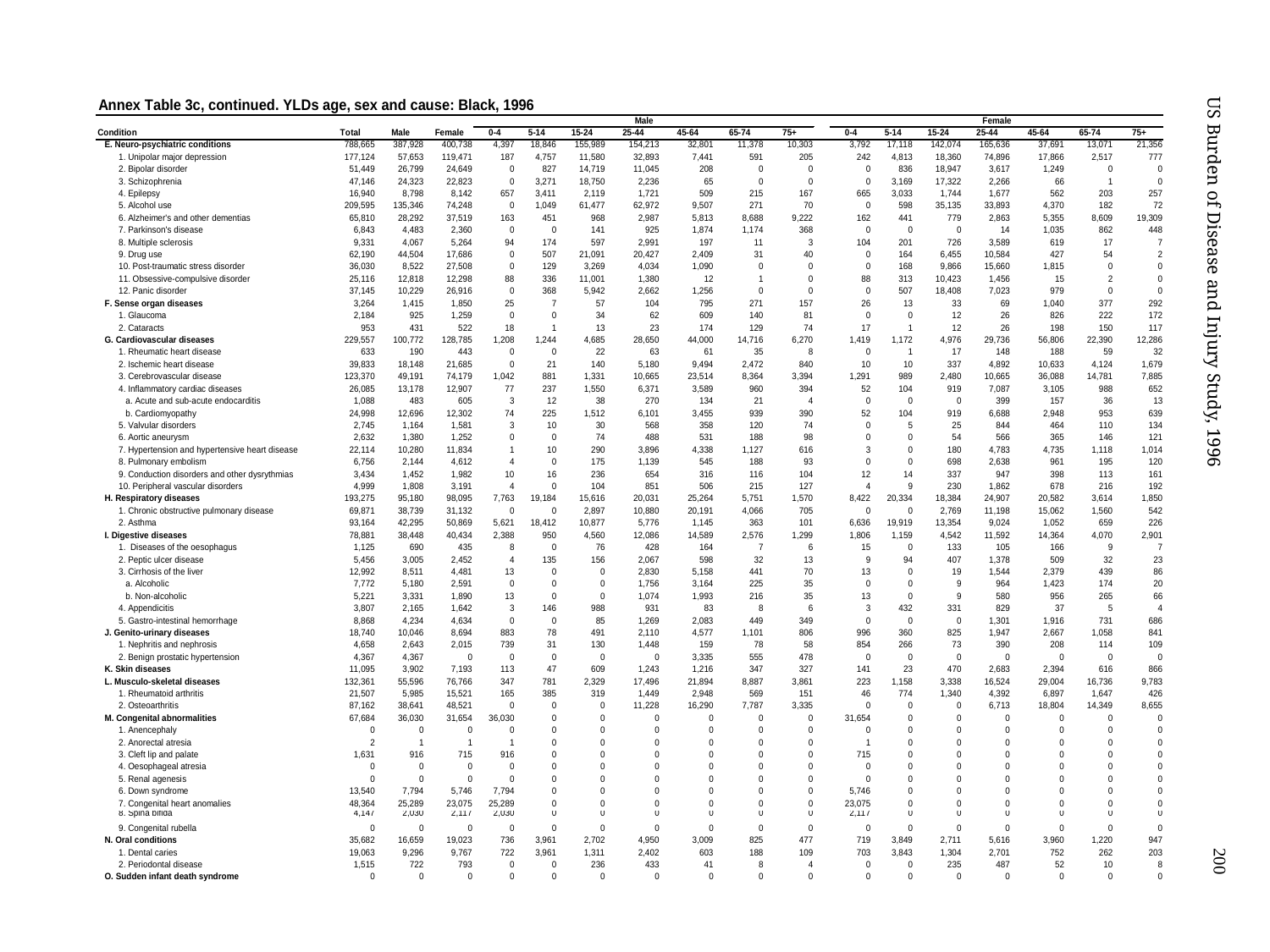

US Burden of Disease and Injury Study, 1996
200

## Slide 39
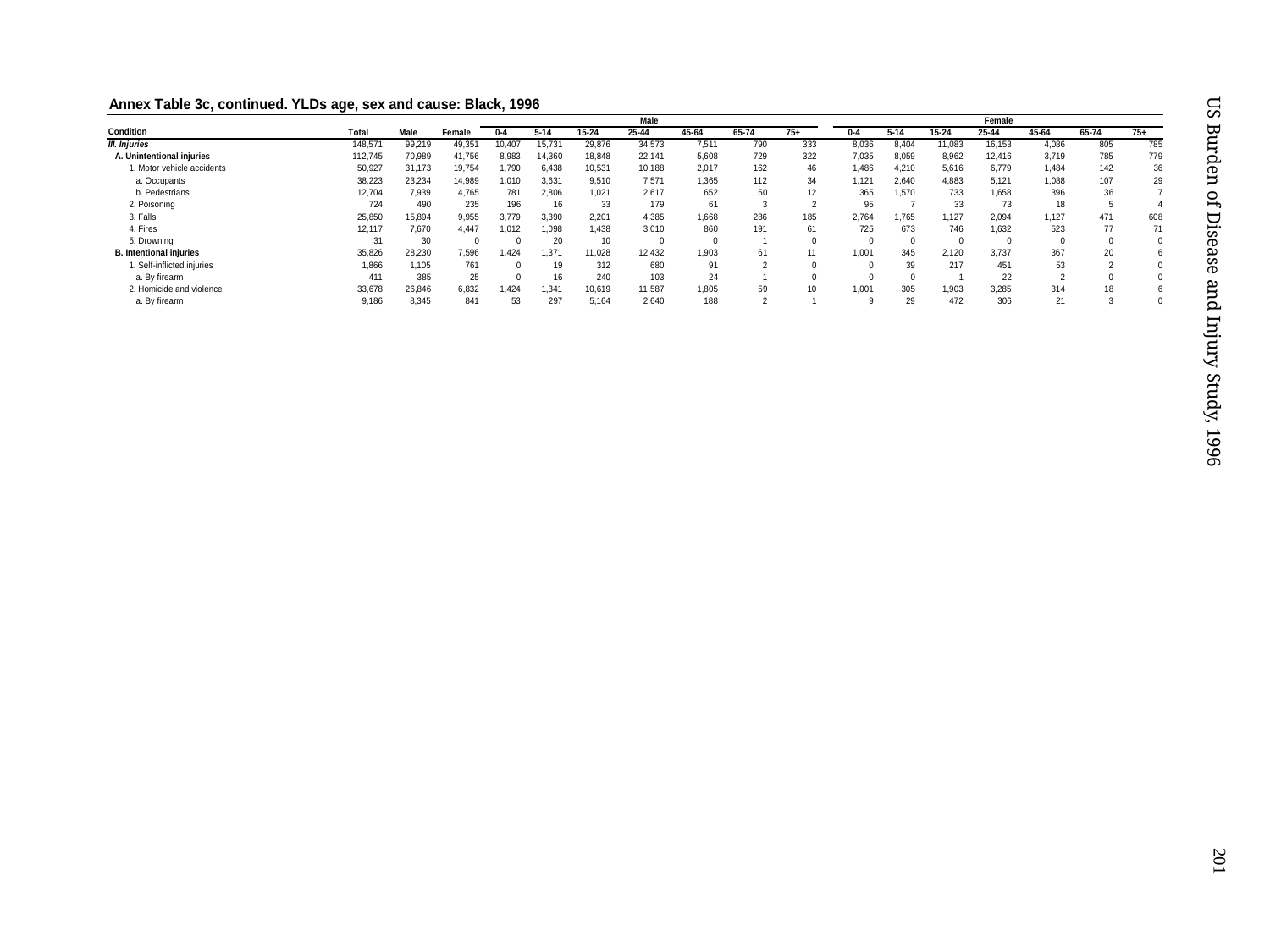

US Burden of Disease and Injury Study, 1996
201

## Slide 40
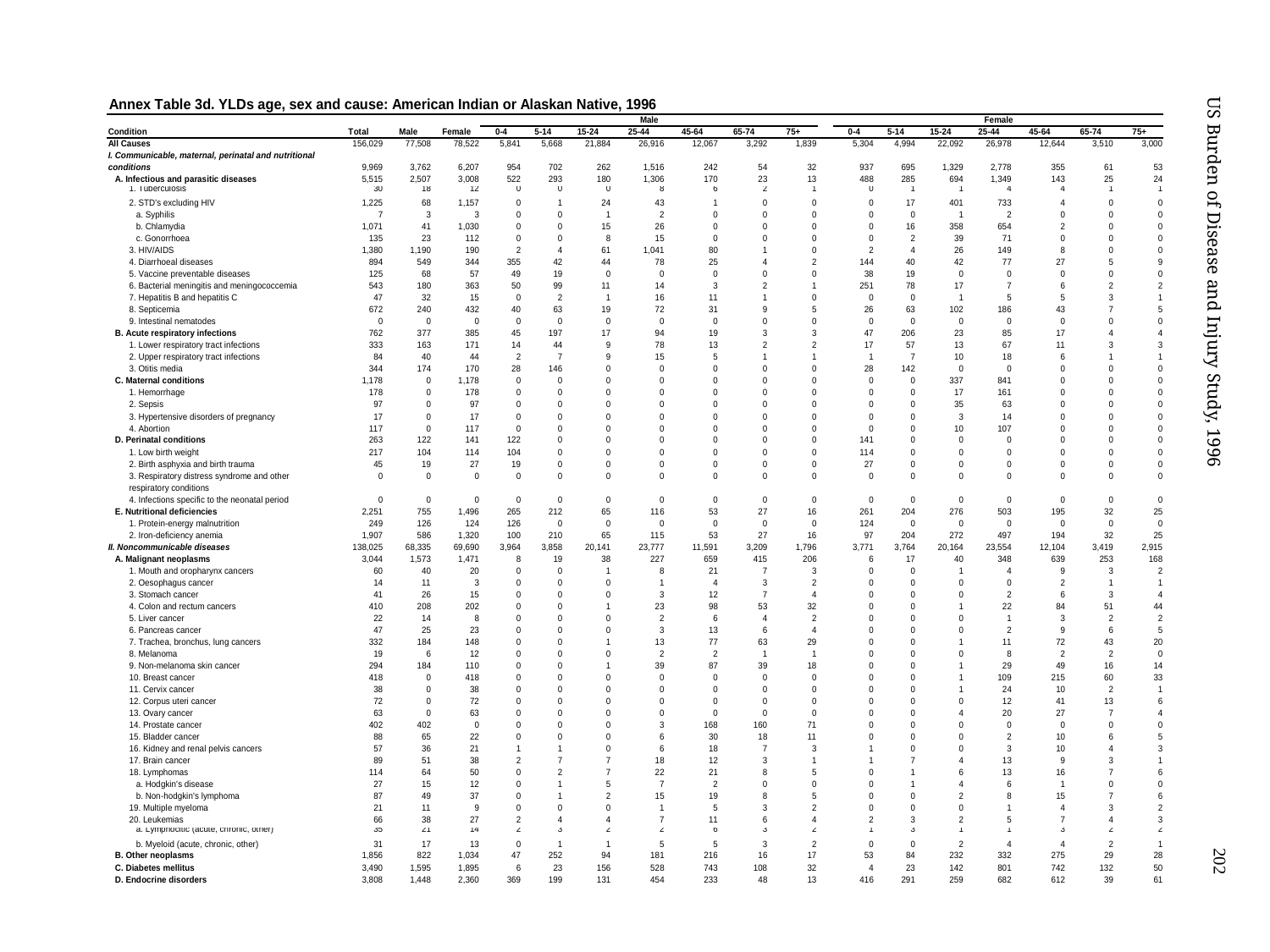

US Burden of Disease and Injury Study, 1996
202

## Slide 41
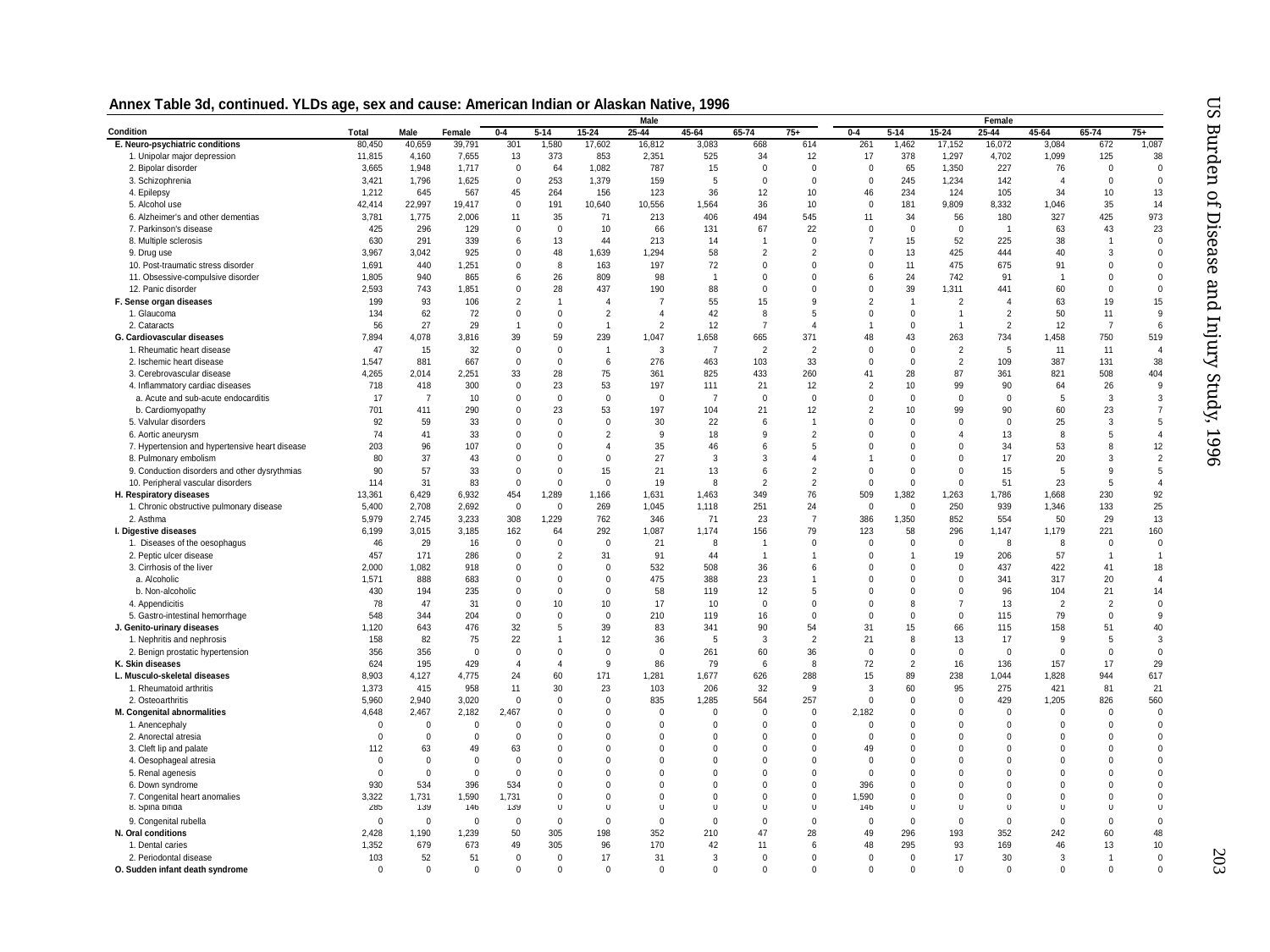

US Burden of Disease and Injury Study, 1996
203

## Slide 42
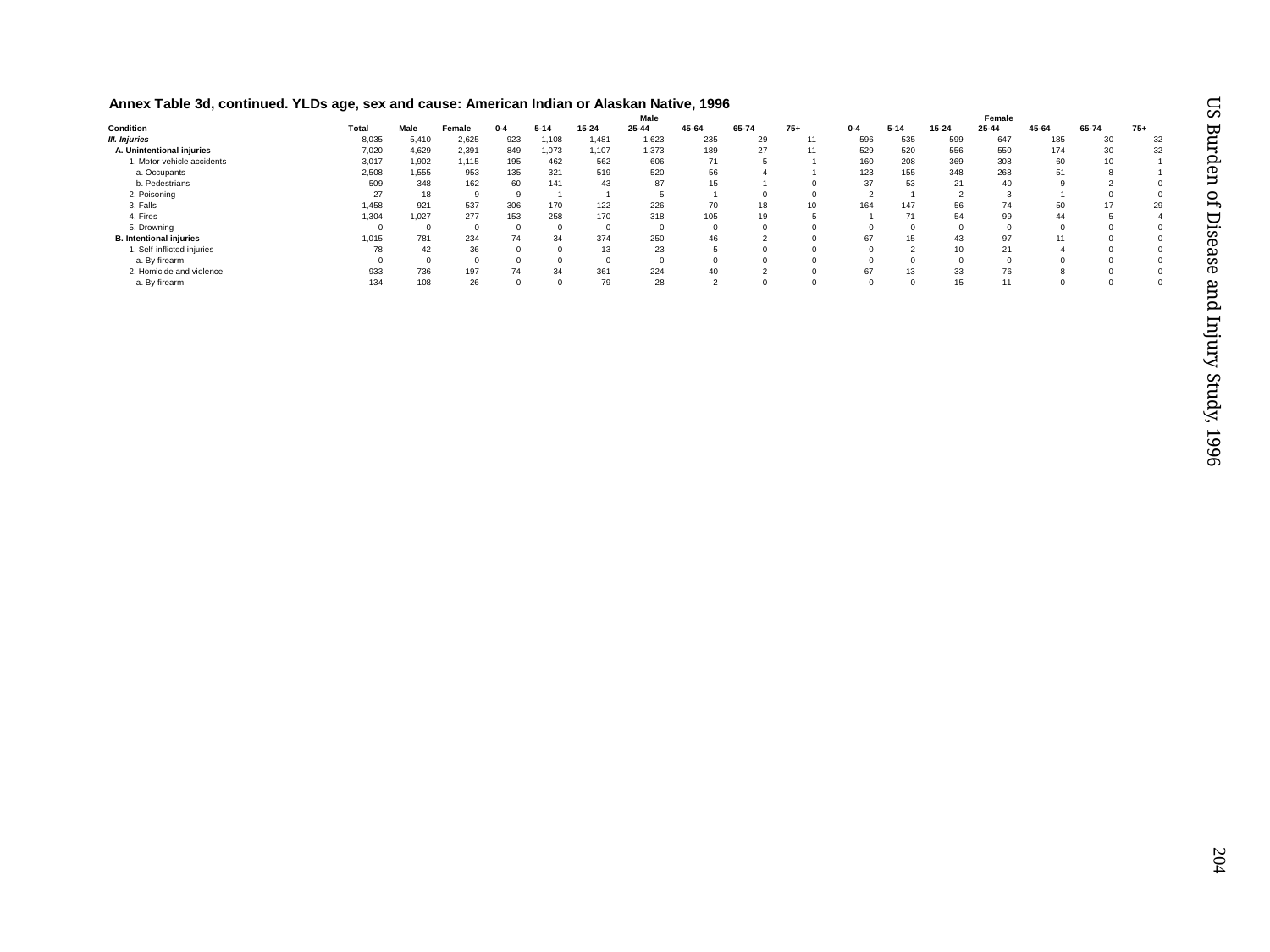

US Burden of Disease and Injury Study, 1996
204

## Slide 43
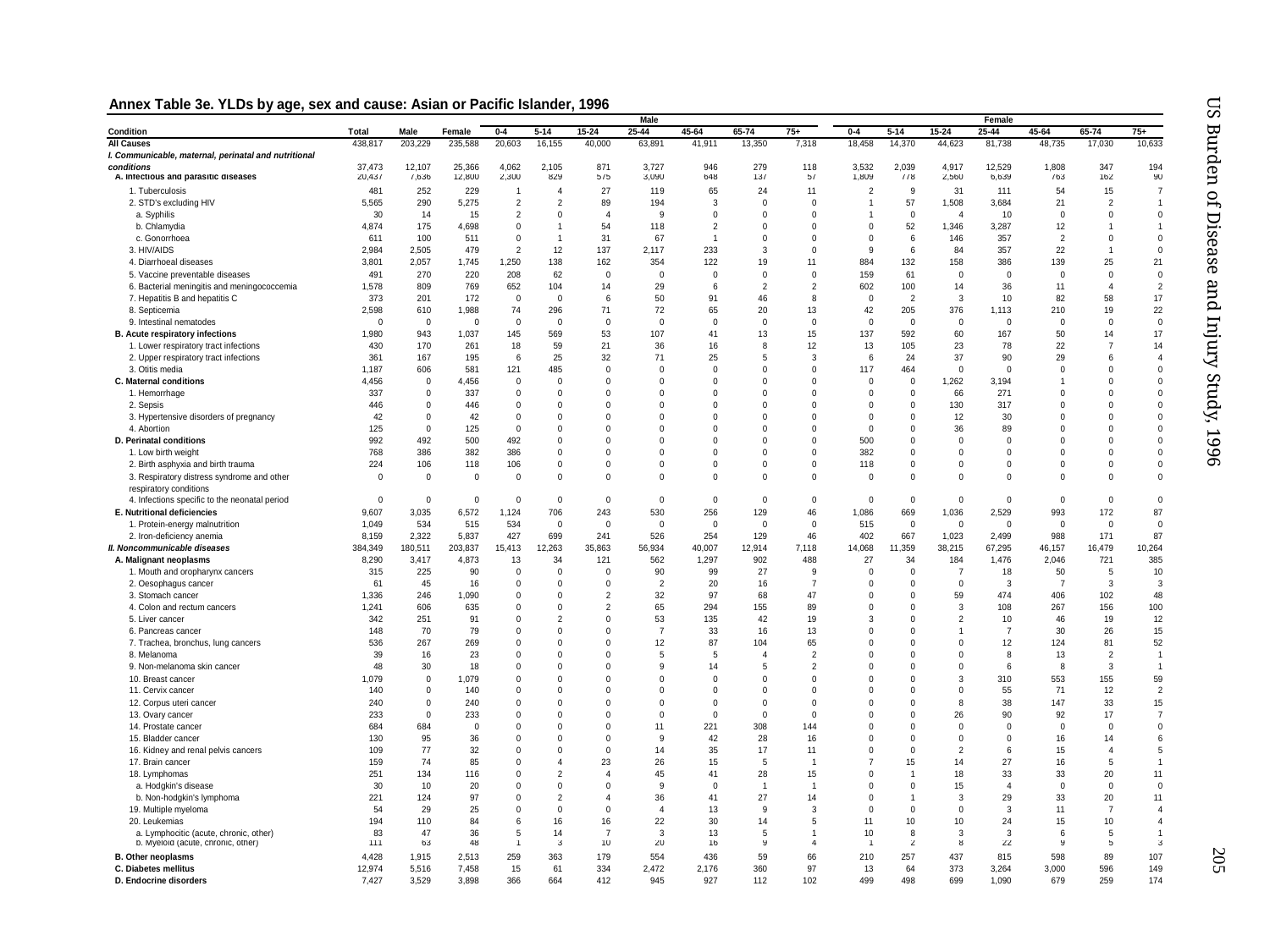

US Burden of Disease and Injury Study, 1996
205

## Slide 44
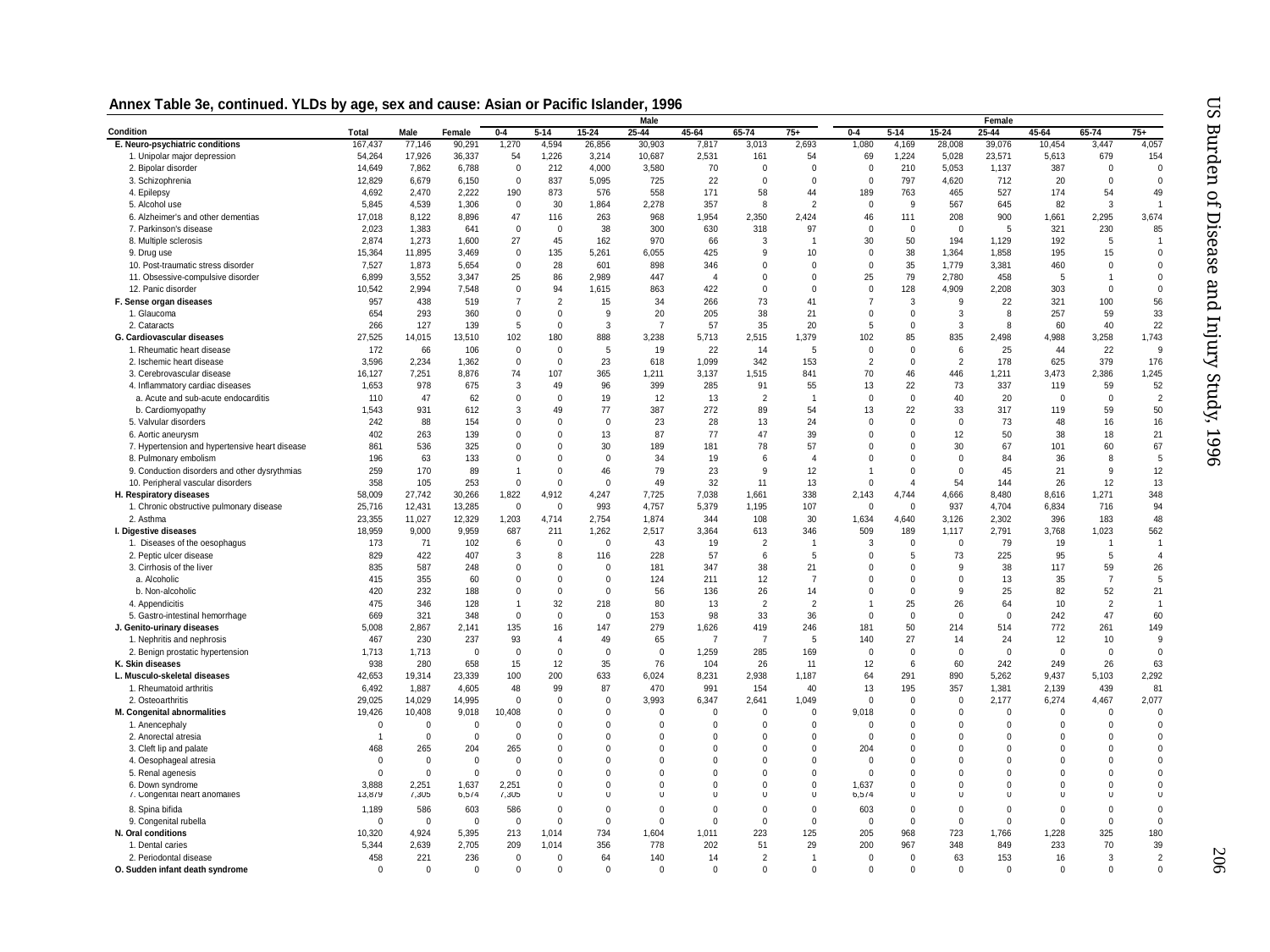

US Burden of Disease and Injury Study, 1996
206

## Slide 45
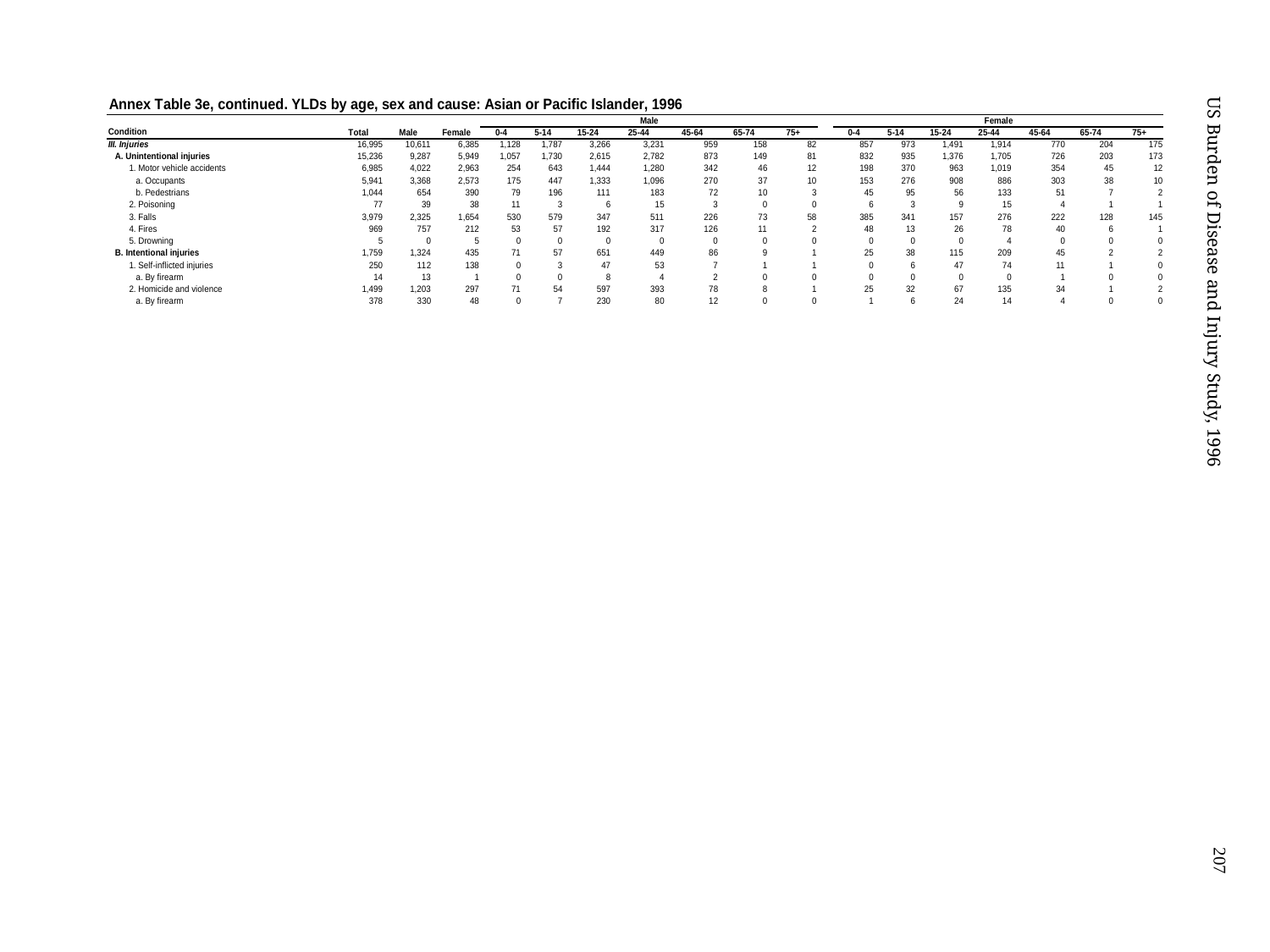

US Burden of Disease and Injury Study, 1996
207

## Slide 46
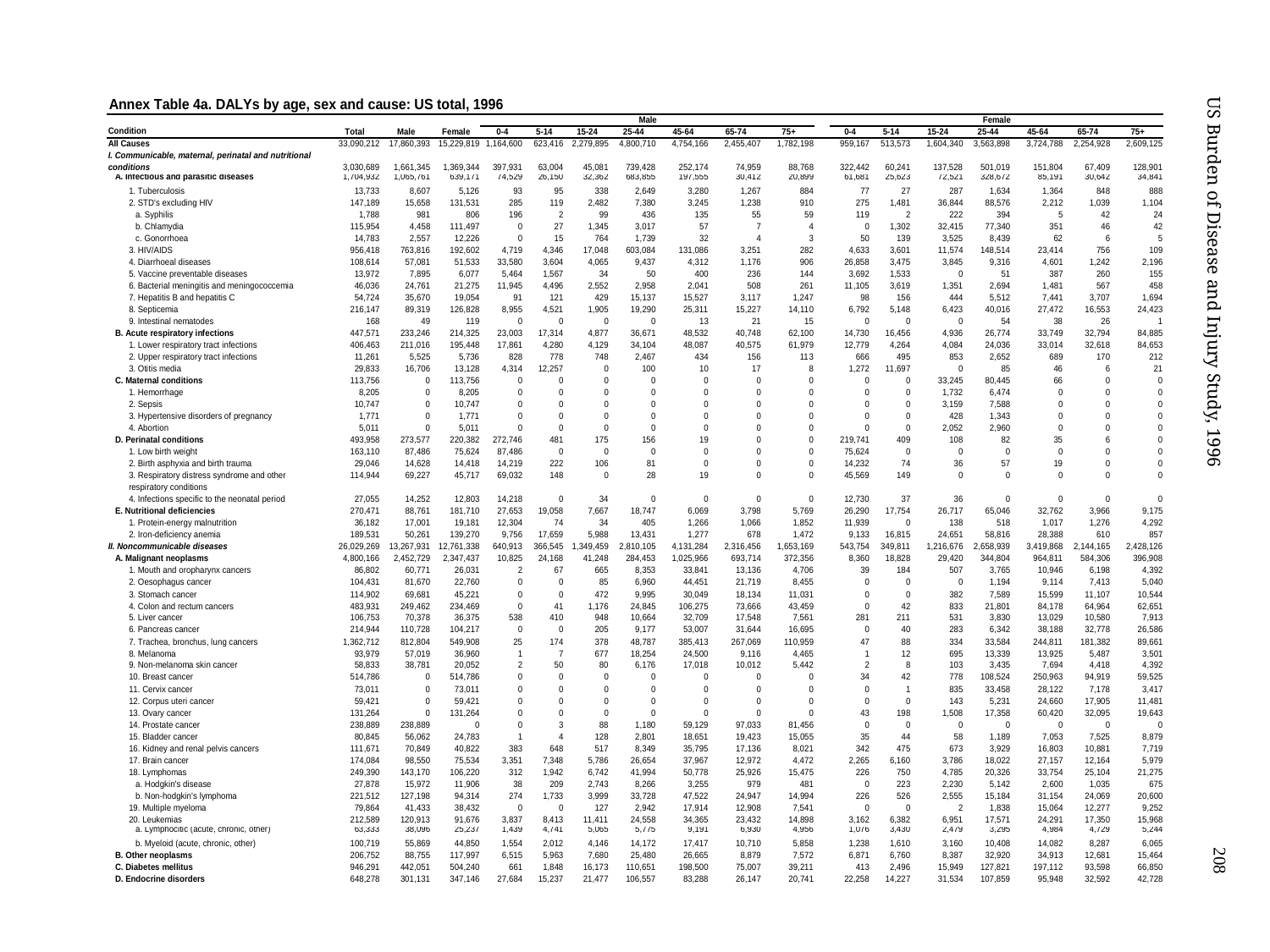

US Burden of Disease and Injury Study, 1996
208

## Slide 47
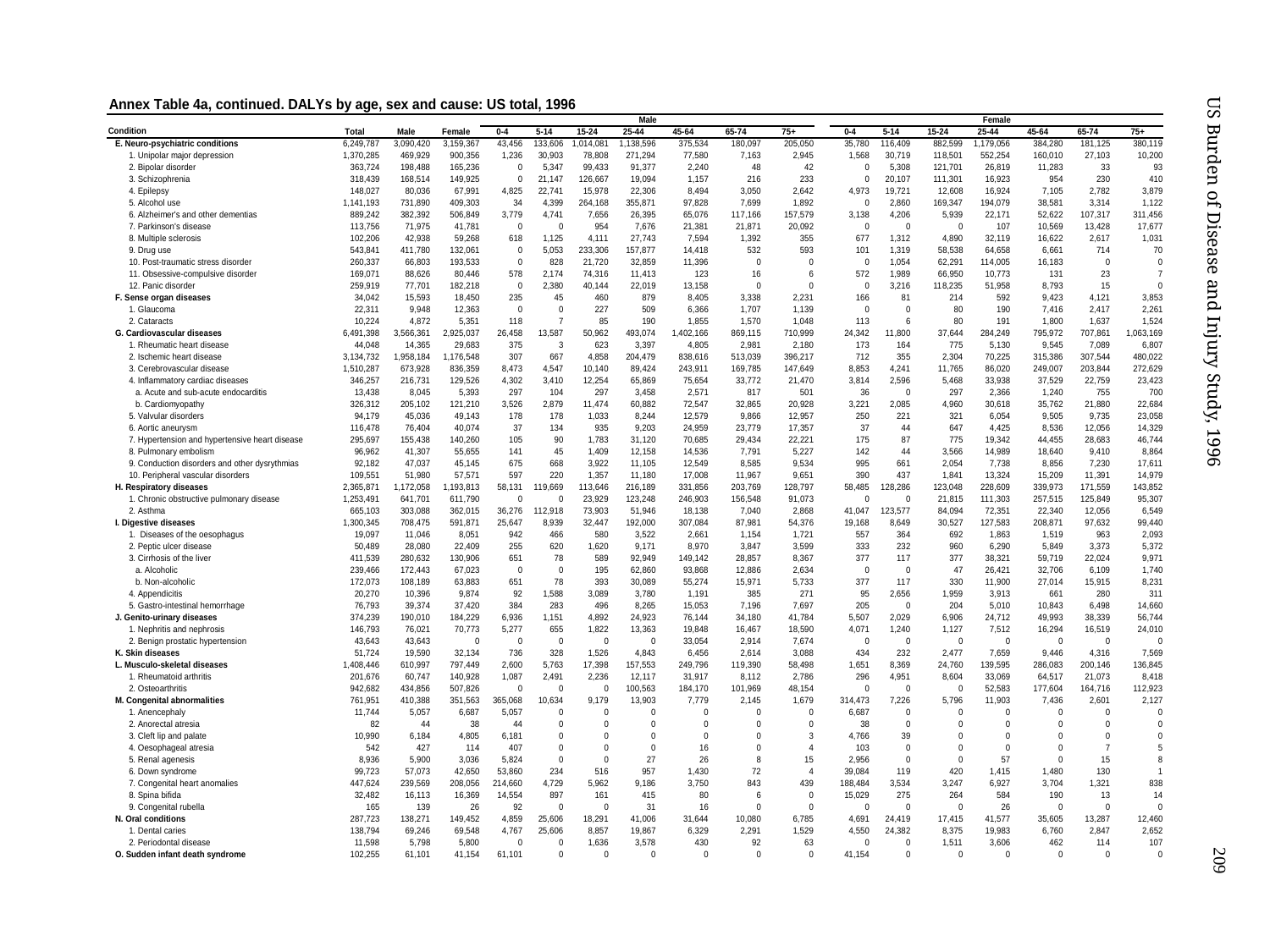

US Burden of Disease and Injury Study, 1996
209

## Slide 48
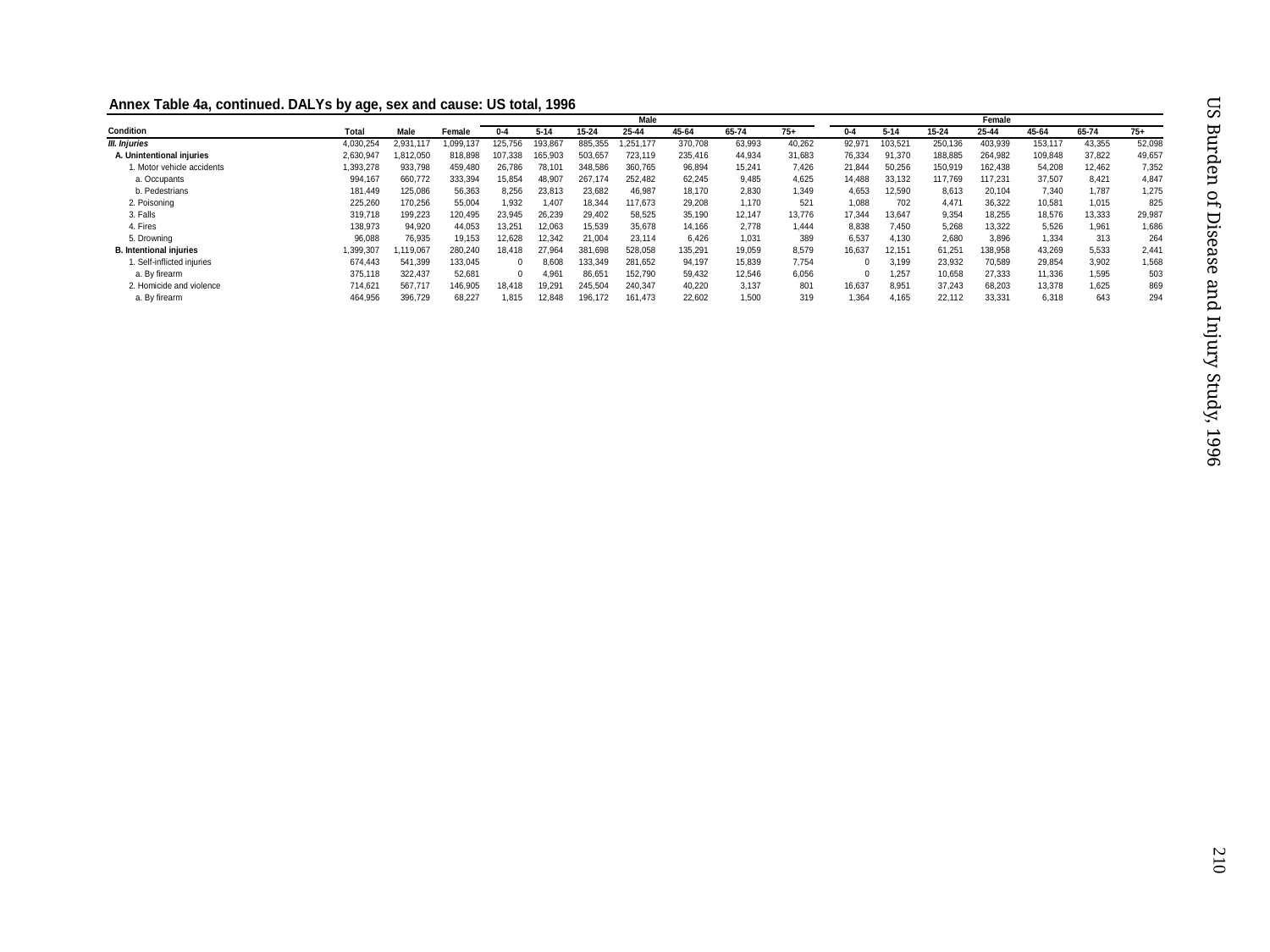

US Burden of Disease and Injury Study, 1996
210

## Slide 49
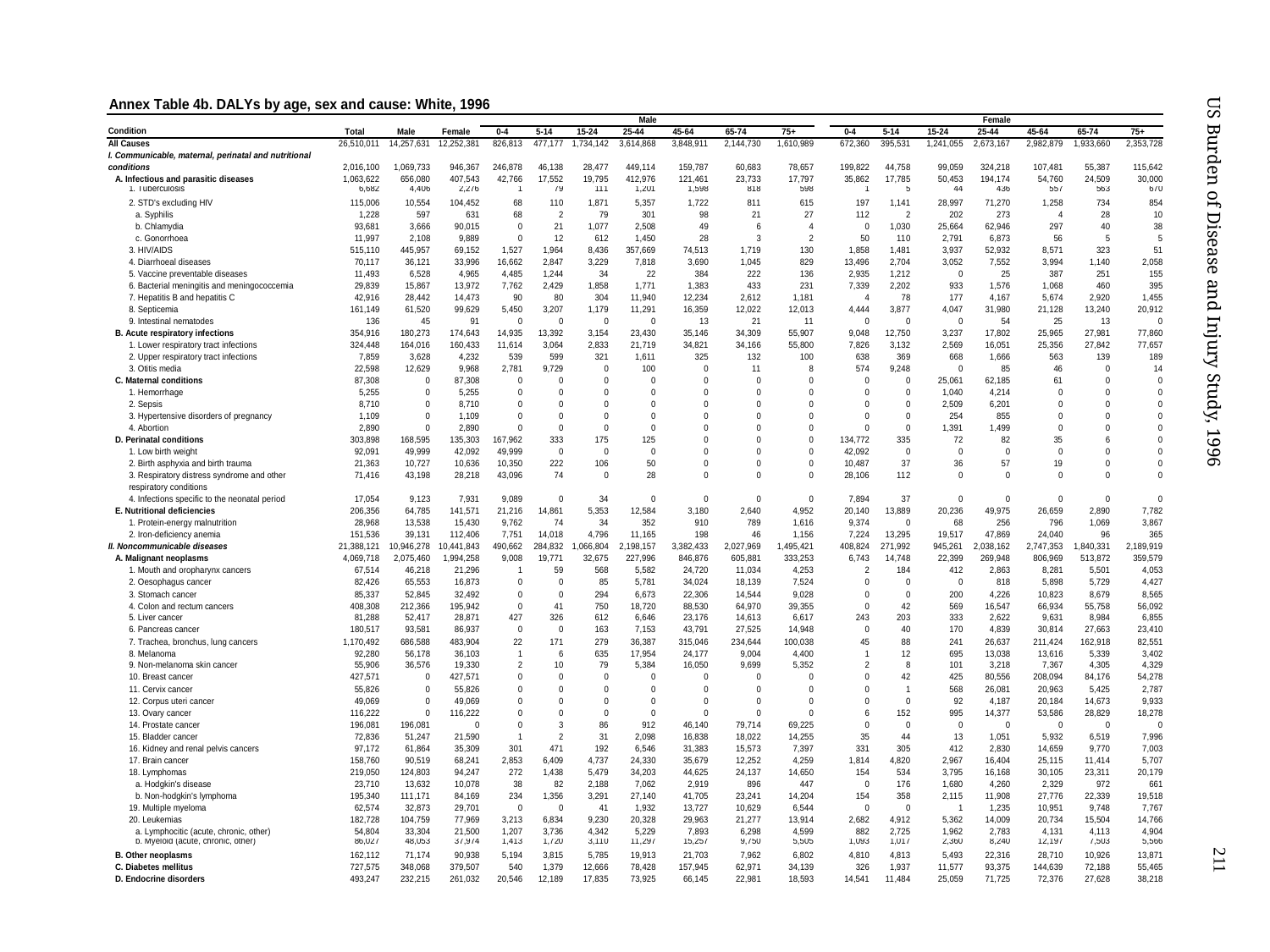

US Burden of Disease and Injury Study, 1996
211

## Slide 50
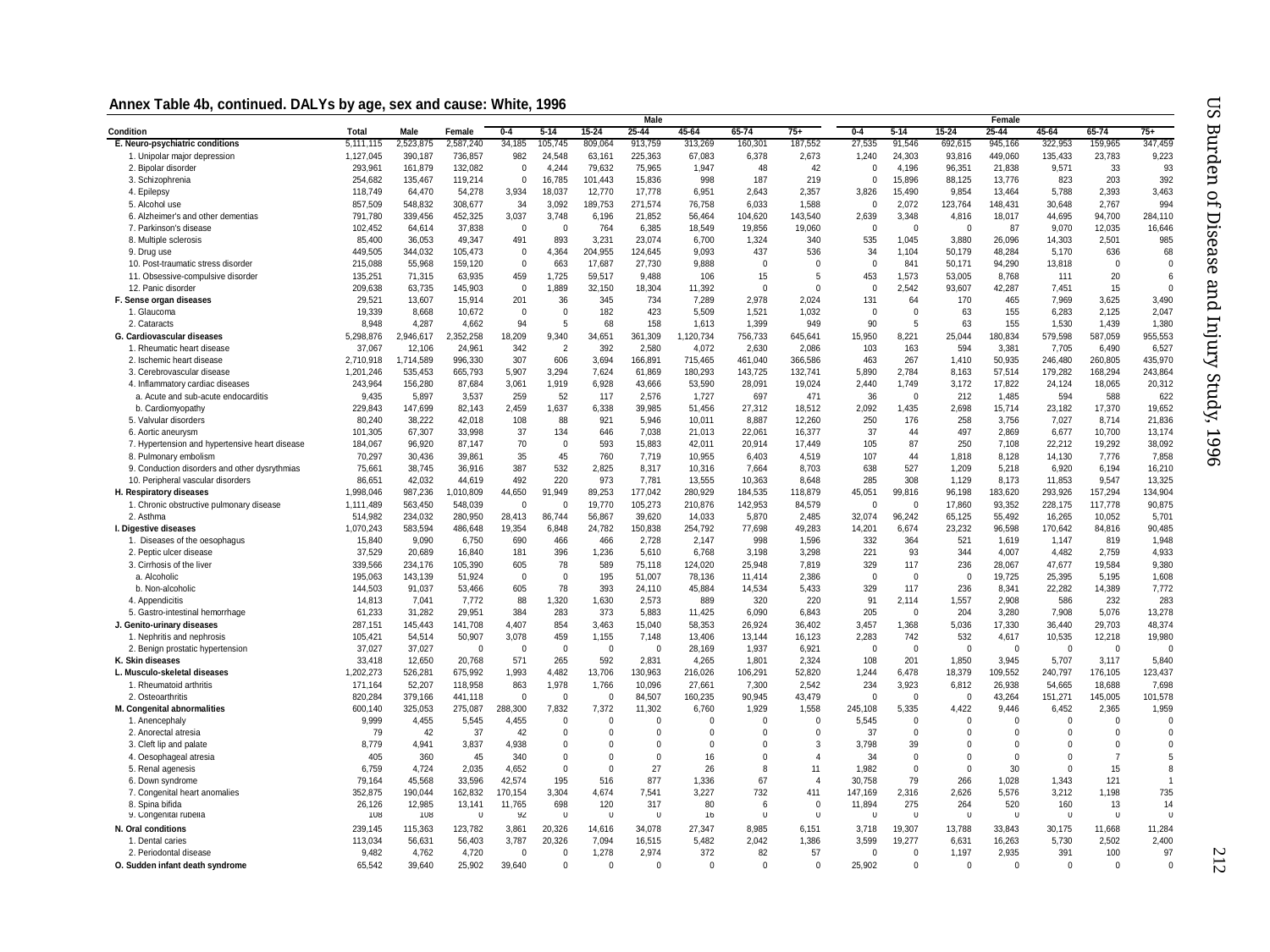

US Burden of Disease and Injury Study, 1996
212

## Slide 51
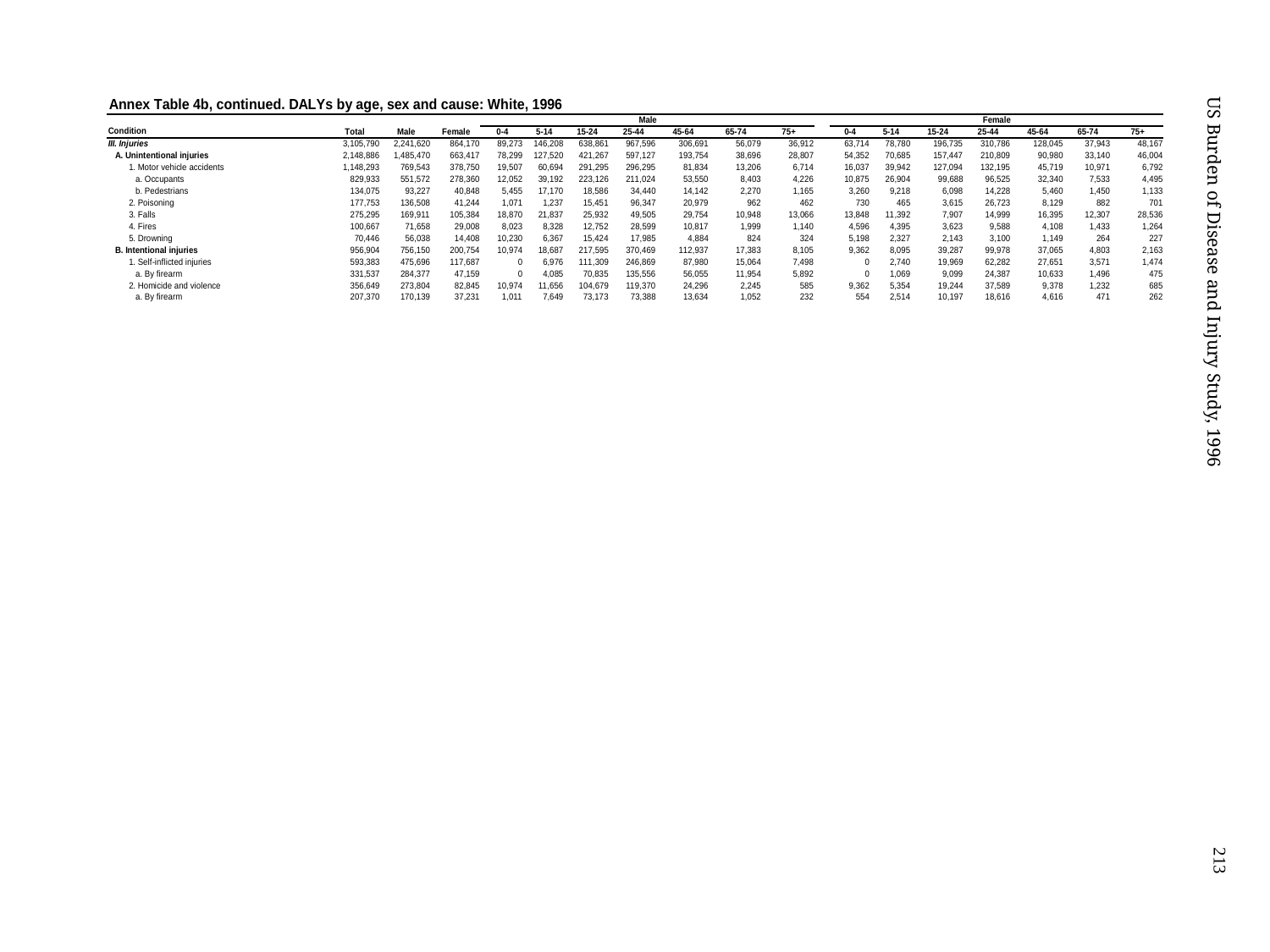

US Burden of Disease and Injury Study, 1996
213

## Slide 52
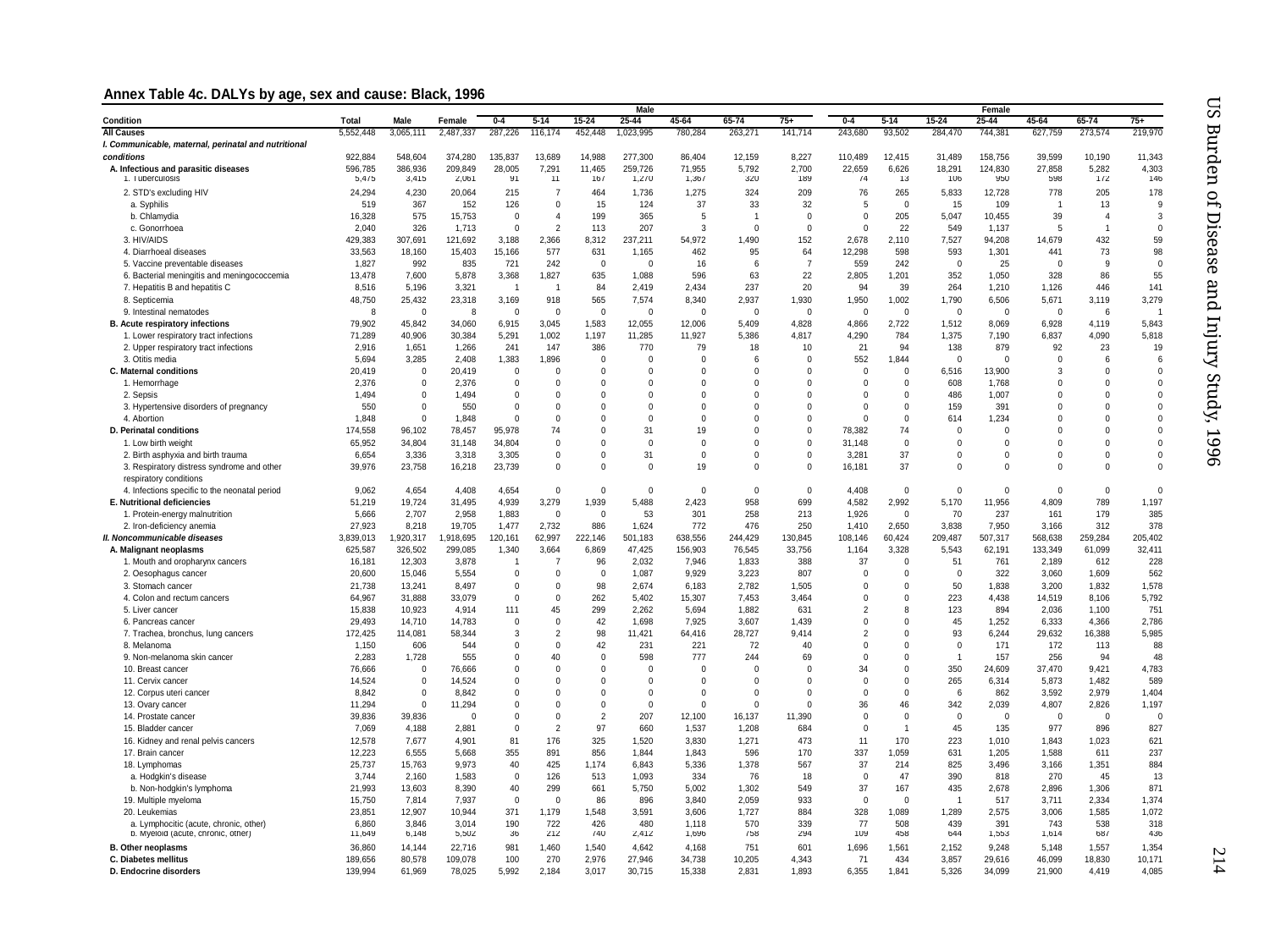

US Burden of Disease and Injury Study, 1996
214

## Slide 53
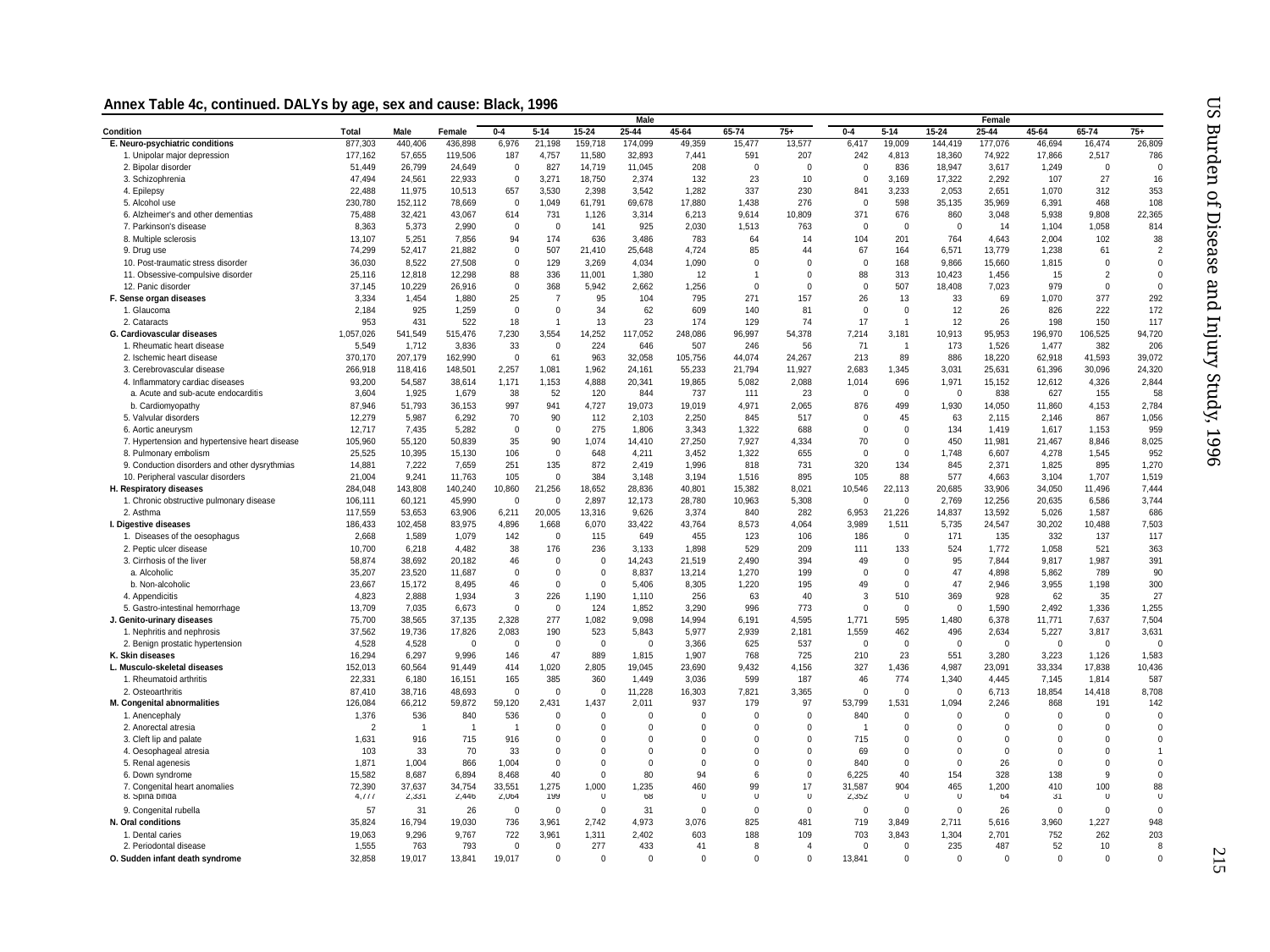

US Burden of Disease and Injury Study, 1996
215

## Slide 54
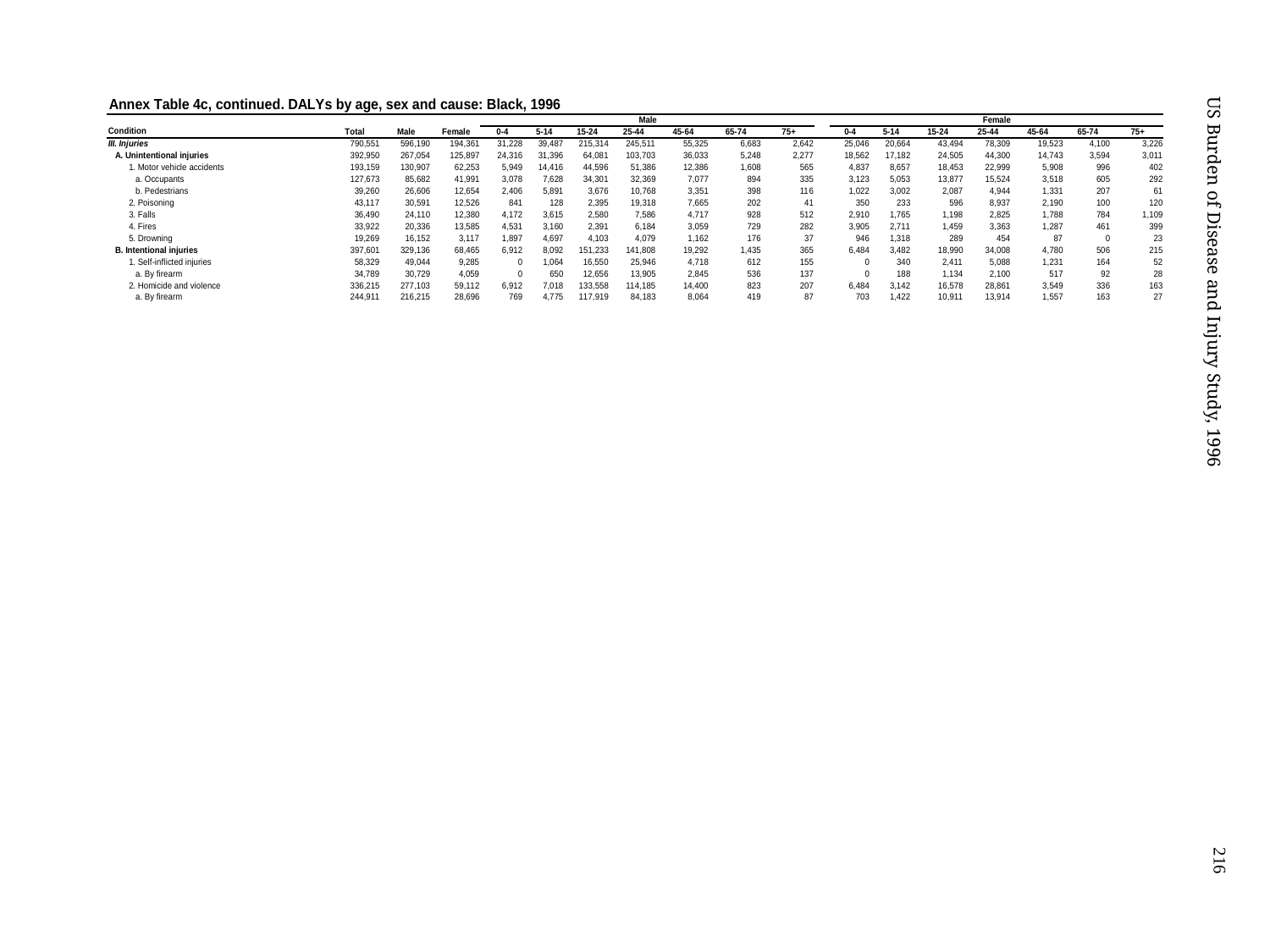

US Burden of Disease and Injury Study, 1996
216

## Slide 55
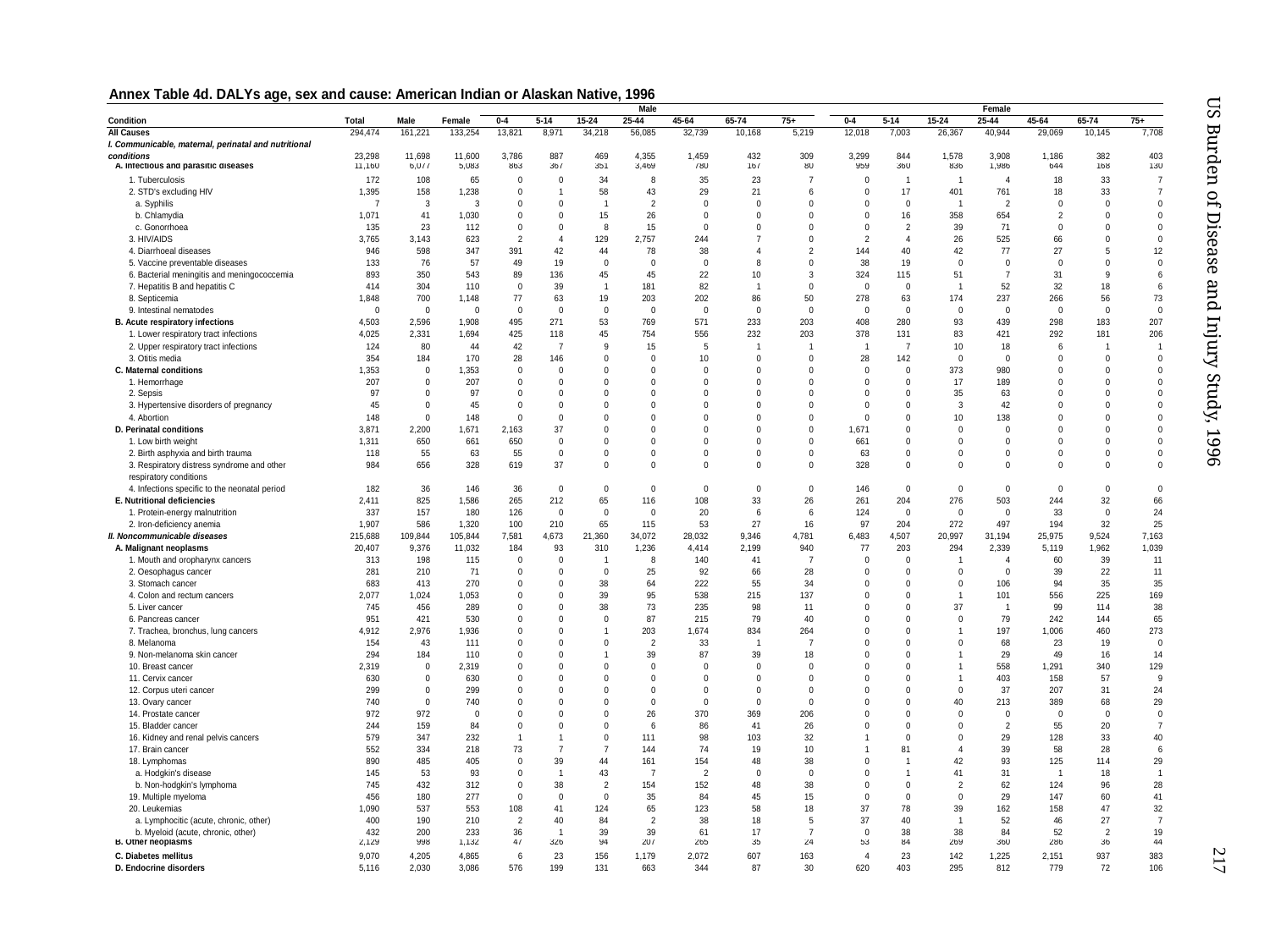

US Burden of Disease and Injury Study, 1996
217

## Slide 56
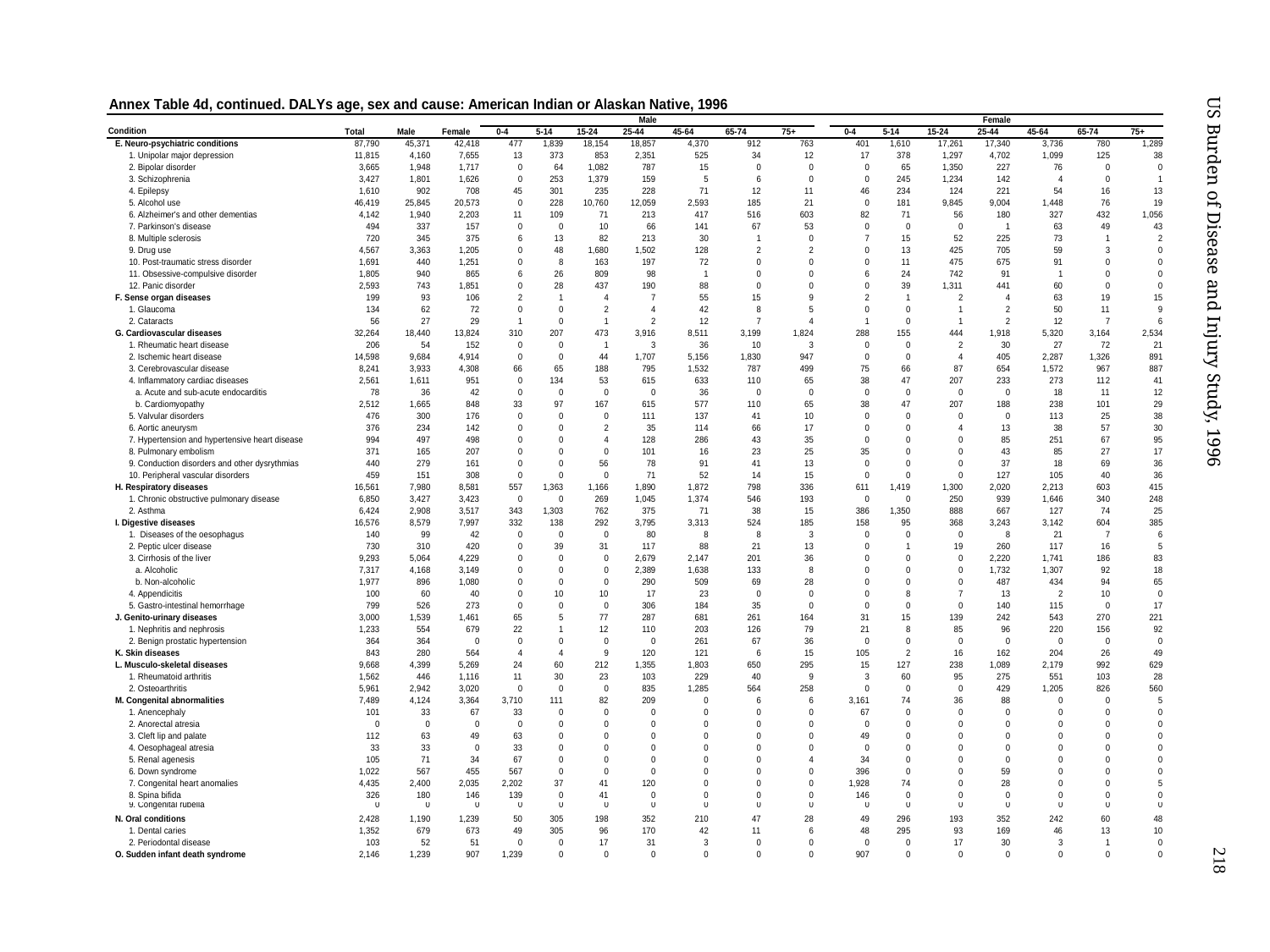

US Burden of Disease and Injury Study, 1996
218

## Slide 57
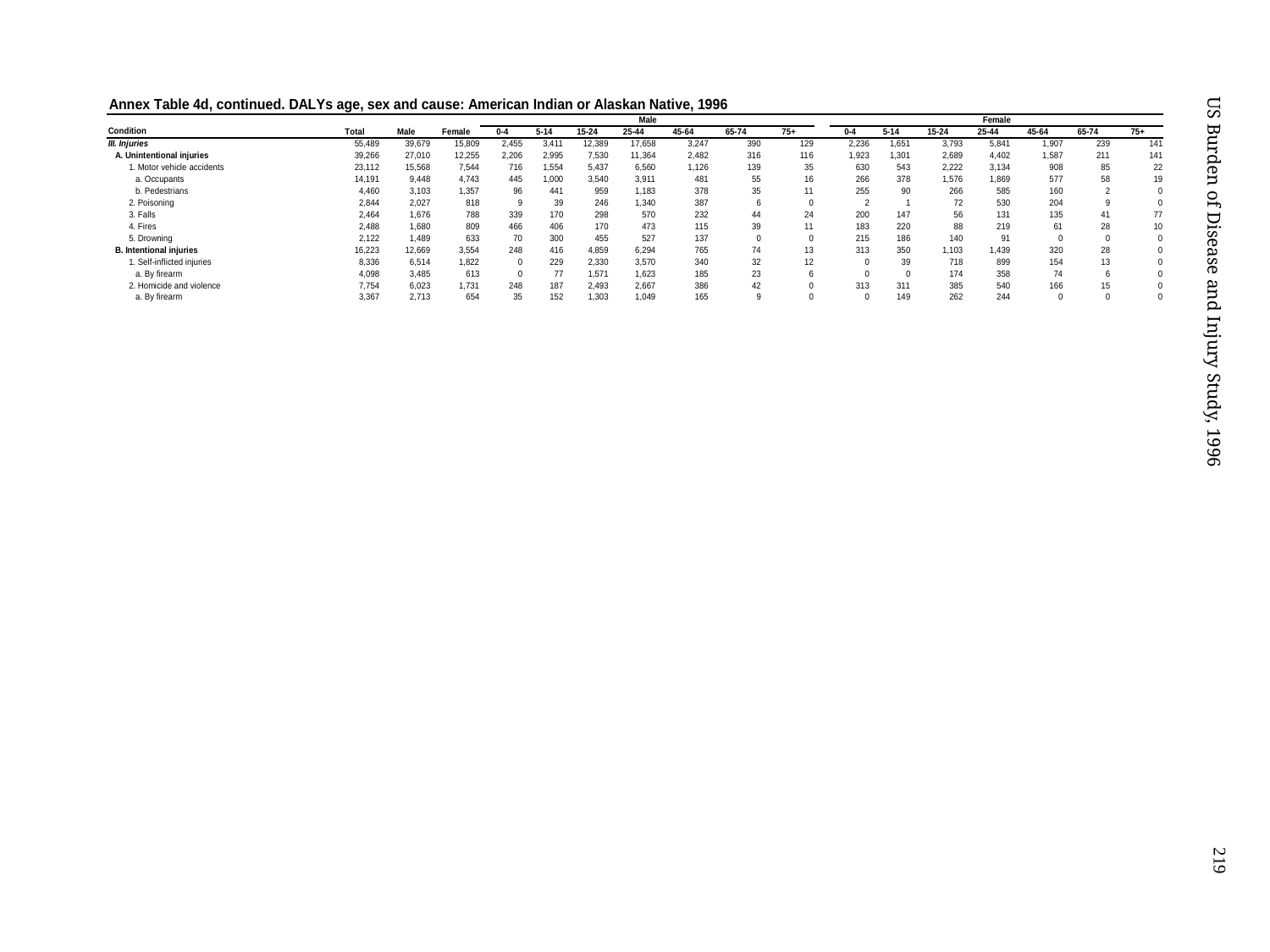

US Burden of Disease and Injury Study, 1996
219

## Slide 58
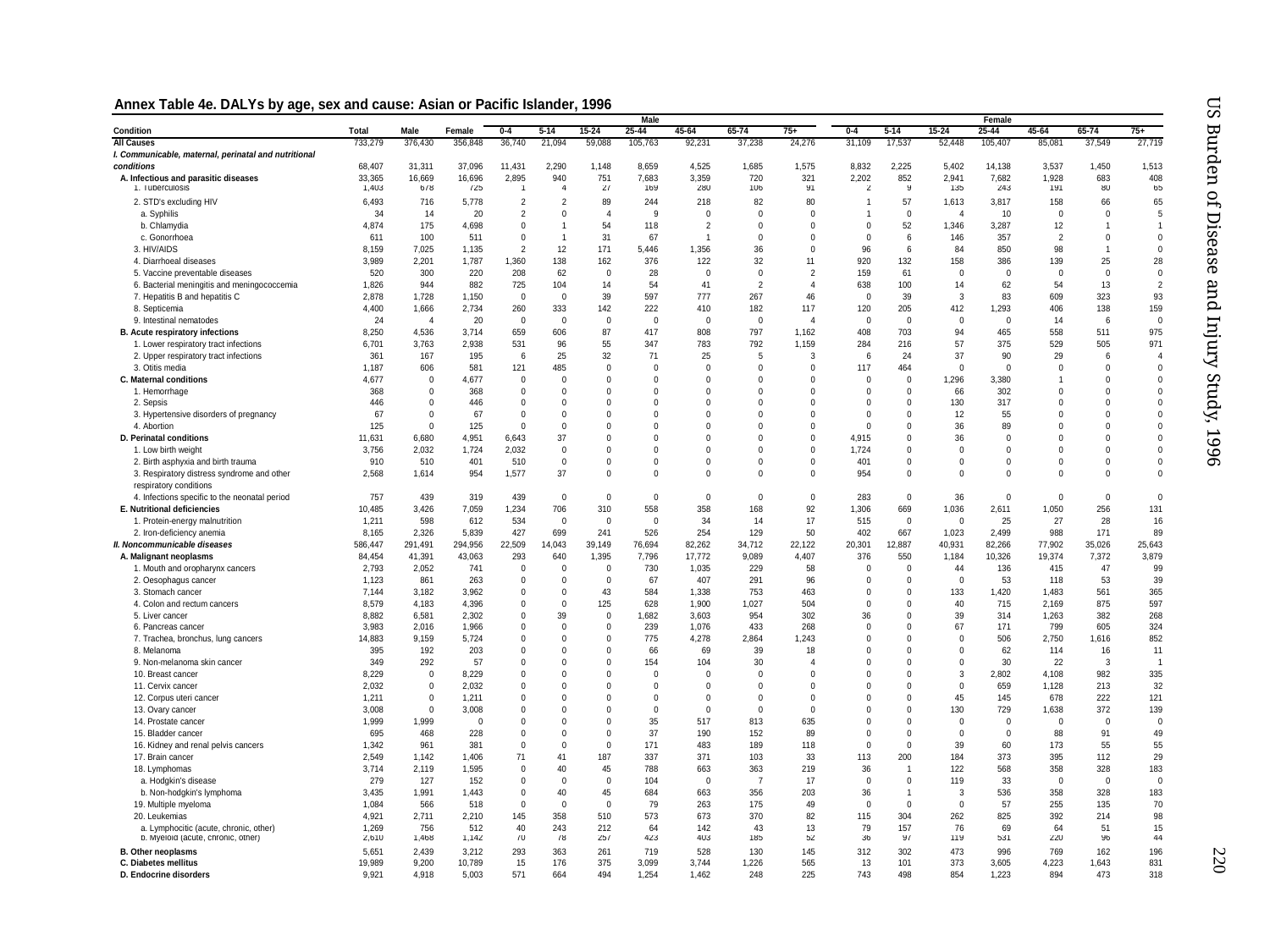

US Burden of Disease and Injury Study, 1996
220

## Slide 59
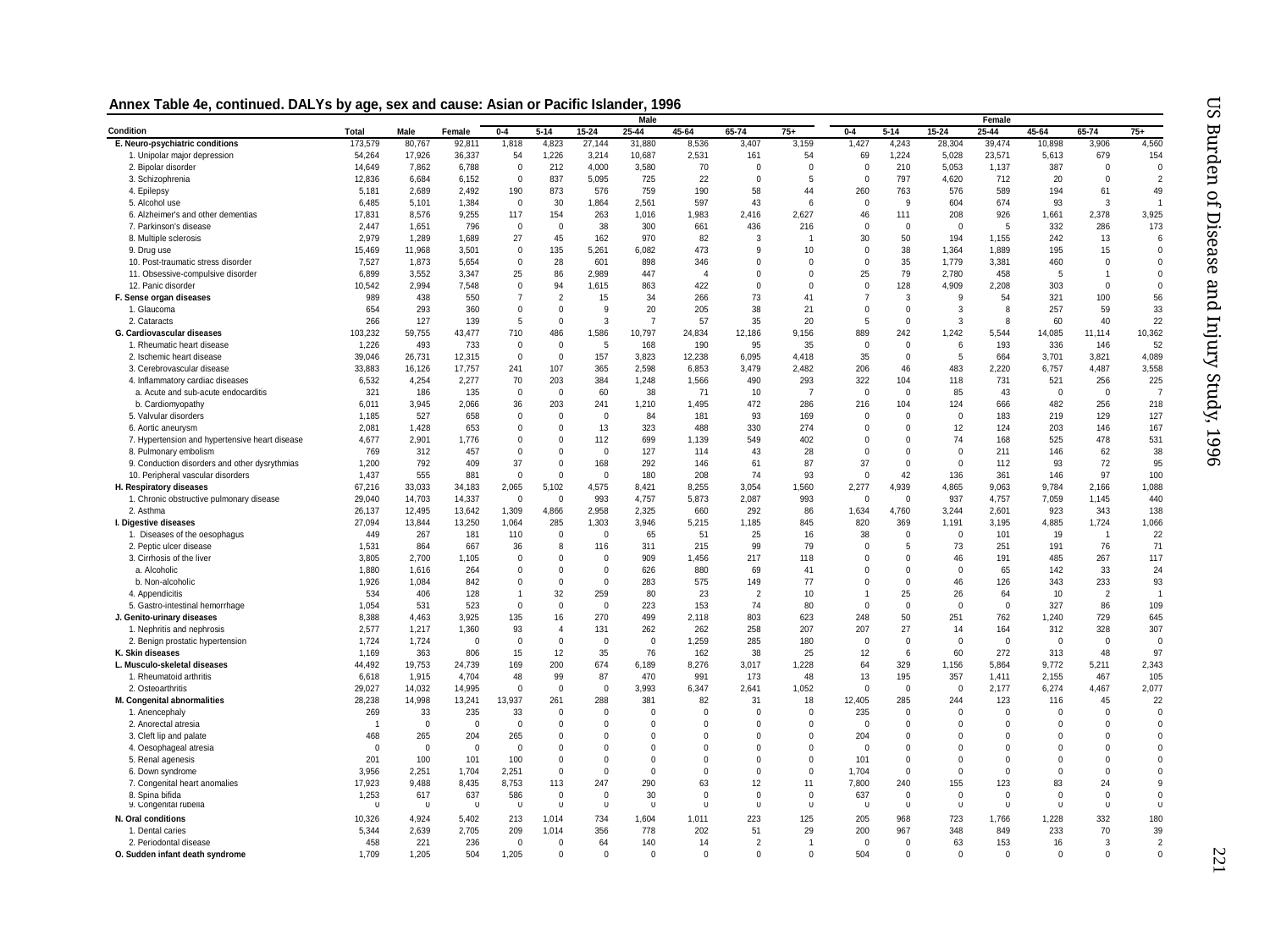

US Burden of Disease and Injury Study, 1996
221

## Slide 60
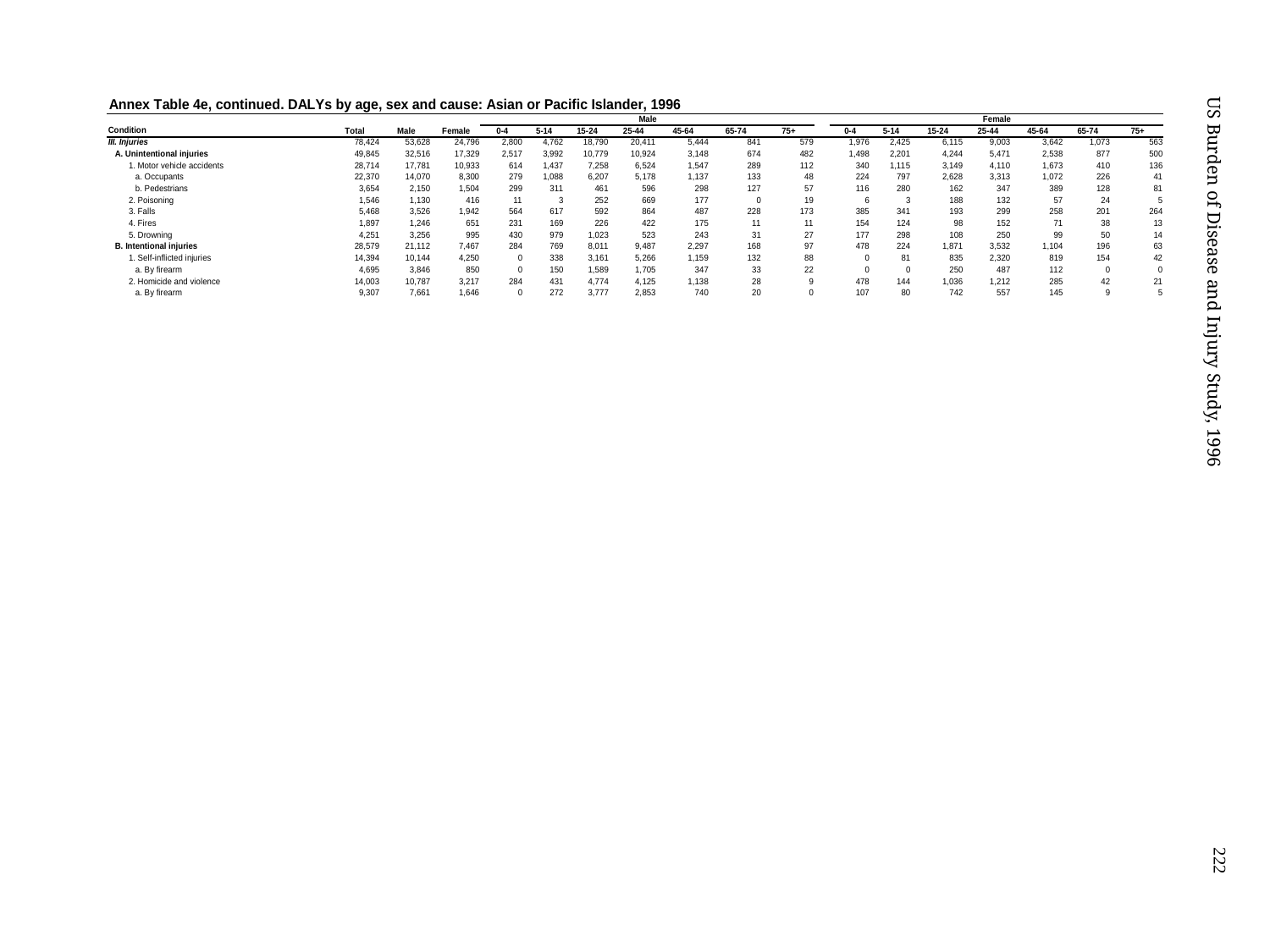

US Burden of Disease and Injury Study, 1996
222
